# Supplementary material for: Investigating the impact of poverty on mental illness in the UK Biobank using Mendelian randomization
Source: Nat Hum Behav. 2024 Jul 10;8(9):1771–83. doi: 10.1038/s41562-024-01919-3 (PMC11420075; doi:10.1038/s41562-024-01919-3)
Supplement: Supplementary file 1 — Supplementary Figs. 1–133, Tables 1–33, Methods and Frequently Asked Questions (FAQ). [file 41562_2024_1919_MOESM1_ESM.pdf]

nature human behaviour

Supplementary information
https://doi.org/10.1038/s41562-024-01919-3

Investigating the impact of poverty on mental illness in the UK Biobank using Mendelian randomization

In the format provided by the authors and unedited

Summary

METHODS DETAILS **6**

HOUSEHOLD INCOME AND SOCIAL DEPRIVATION GWAS 6
COGNITIVE ABILITY GWAS 6
MR-PRESSO 8
CAUSE 8

MULTIVARIABLE GWAS OF THE LATENT POVERTY FACTOR **9**

SUPPLEMENTARY TABLE 1: FACTOR LOADING OF EACH POVERTY INDICATOR USED FOR THE ESTIMATION OF THE LATENT POVERTY FACTOR 9
SUPPLEMENTARY TABLE 2: SUMMARY STATISTICS OF THE COMMON POVERTY FACTOR, HOUSEHOLD INCOME, SOCIAL DEPRIVATION, AND OCCUPATIONAL INCOME 9
SUPPLEMENTARY TABLE 3: GENETIC CORRELATION (RG) BETWEEN THE COMMON FACTOR POVERTY, HOUSEHOLD INCOME, SOCIAL DEPRIVATION, OCCUPATIONAL INCOME, AND COGNITIVE ABILITIES 10
SUPPLEMENTARY TABLE 4: RESULTS OF BIDIRECTIONAL MENDELIAN RANDOMIZATION OF COGNITIVE ABILITIES AGAINST POVERTY 10
PLOTS - FORWARD ANALYSES 12
Supplementary Figure 1: scatterplot of cognitive abilities against common factor poverty *12*
Supplementary Figure 2: leave-one out analysis of cognitive abilities against common factor poverty *13*
PLOTS - BACKWARD ANALYSES 13
Supplementary Figure 3: scatterplot of common factor poverty against cognitive abilities *13*
Supplementary Figure 4: leave-one out analysis of common factor poverty against cognitive abilities *14*

UNIVARIABLE MENDELIAN RANDOMIZATION OF POVERTY AND MENTAL ILLNESSES **15**

SUPPLEMENTARY TABLE 5: RESULTS OF BIDIRECTIONAL MR OF POVERTY AGAINST MENTAL ILLNESS 15
SUPPLEMENTARY TABLE 6: ODDS RATIO OF UNIVARIABLE FORWARD MENDELIAN RANDOMIZATION ANALYSIS OF POVERTY AGAINST MENTAL ILLNESSES 18
PLOTS - FORWARD ANALYSES 19
Supplementary Figure 5: scatterplot of poverty against ADHD *19*
Supplementary Figure 6: scatterplot of poverty against AN *20*
Supplementary Figure 7: scatterplot of poverty against ANX *20*
Supplementary Figure 8: scatterplot of poverty against ASD *21*
Supplementary Figure 9: scatterplot of poverty against BD *21*
Supplementary Figure 10: scatterplot of poverty against MDD *22*
Supplementary Figure 11: scatterplot of poverty against OCD *23*
Supplementary Figure 12: scatterplot of poverty against PTSD *23*
Supplementary Figure 13: scatterplot of poverty against SZ *24*
Supplementary Figure 14: leave-one-out analysis of poverty against ADHD *24*
Supplementary Figure 15: leave-one-out analysis of poverty against AN. *25*
Supplementary Figure 16: leave-one-out analysis of poverty against ANX. *26*
Supplementary Figure 17: leave-one-out analysis of poverty against ASD. *26*
Supplementary Figure 18: leave-one-out analysis of poverty against BD. *27*
Supplementary Figure 19: leave-one-out analysis of poverty against MDD. *27*
Supplementary Figure 20: leave-one-out analysis of poverty against OCD. *28*
Supplementary Figure 21: leave-one-out analysis of poverty against PTSD. *29*
Supplementary Figure 22: leave-one-out analysis of poverty against SZ *29*
PLOTS - BACKWARD ANALYSES 30
Supplementary Figure 23: scatterplot of ADHD against poverty *30*
Supplementary Figure 24: scatterplot of AN against poverty *31*
Supplementary Figure 25: scatterplot of BD against poverty *31*
Supplementary Figure 26: scatterplot of SZ against poverty *32*
Supplementary Figure 27: leave-one-out analysis of ADHD against poverty *33*
Supplementary Figure 28: leave-one-out analysis of BD against poverty *33*
Supplementary Figure 29: leave-one-out analysis of SZ against poverty *34*

SUPPLEMENTARY TABLE 7: CAUSE RESULTS OF THE RELATIONS BETWEEN POVERTY AND MENTAL ILLNESS .. 34
SUPPLEMENTARY TABLE 8: RESULTS OF UNIVARIABLE BIDIRECTIONAL MENDELIAN RANDOMIZATION OF POVERTY AGAINST MENTAL ILLNESSES, AFTER STEIGER FILTERING 37

UNIVARIABLE MENDELIAN RANDOMIZATION OF HOUSEHOLD INCOME AND MENTAL ILLNESSES 38

SUPPLEMENTARY TABLE 9: ODDS RATIO OF UNIVARIABLE FORWARD MENDELIAN RANDOMIZATION ANALYSIS OF HOUSEHOLD INCOME AGAINST MENTAL ILLNESSES 38
SUPPLEMENTARY TABLE 10: RESULTS OF UNIVARIABLE BIDIRECTIONAL MENDELIAN RANDOMIZATION OF HOUSEHOLD INCOME AGAINST MENTAL ILLNESSES 39
PLOTS - FORWARD ANALYSES 42
Supplementary Figure 30: scatterplot of household income against ADHD 42
Supplementary Figure 31: scatterplot of household income against AN 43
Supplementary Figure 32: scatterplot of household income against ANX 43
Supplementary Figure 33: scatterplot of household income against ASD 44
Supplementary Figure 34: scatterplot of household income against BD 45
Supplementary Figure 35: scatterplot of household income against MDD 45
Supplementary Figure 36: scatterplot of household income against OCD 46
Supplementary Figure 37: scatterplot of household income against PTSD 47
Supplementary Figure 38: scatterplot of household income against SZ 47
Supplementary Figure 39: leave-one-out analysis of household income against ADHD 48
Supplementary Figure 40: leave-one-out analysis of household income against AN 49
Supplementary Figure 41: leave-one-out analysis of household income against ANX 49
Supplementary Figure 42: leave-one-out analysis of household income against ASD 50
Supplementary Figure 43: leave-one-out analysis of household income against BD 51
Supplementary Figure 44: leave-one-out analysis of household income against MDD 51
Supplementary Figure 45: leave-one-out analysis of household income against OCD 52
Supplementary Figure 46: leave-one-out analysis of household income against PTSD 53
Supplementary Figure 47: leave-one-out analysis of household income against SZ 53
PLOTS - BACKWARD ANALYSES 54
Supplementary Figure 48: scatterplot of ADHD against household income 54
Supplementary Figure 49: scatterplot of AN against household income 55
Supplementary Figure 50: scatterplot of BD against household income 55
Supplementary Figure 51: scatterplot of SZ against household income 56
Supplementary Figure 52: leave-one-out analysis of ADHD against household income 57
Supplementary Figure 53: leave-one-out analysis of AN against household income 57
Supplementary Figure 54: leave-one-out analysis of BD against household income 58
Supplementary Figure 55: leave-one-out analysis of SZ against household income 59
SUPPLEMENTARY TABLE 11: CAUSE RESULTS OF THE RELATIONS BETWEEN HOUSEHOLD INCOME AND MENTAL ILLNESSES 59
SUPPLEMENTARY TABLE 12: RESULTS OF UNIVARIABLE BIDIRECTIONAL MENDELIAN RANDOMIZATION OF HOUSEHOLD INCOME AGAINST MENTAL ILLNESSES, AFTER STEIGER FILTERING 61

UNIVARIABLE MENDELIAN RANDOMIZATION OF OCCUPATIONAL INCOME AND MENTAL ILLNESSES 62

SUPPLEMENTARY TABLE 13: ODDS RATIO OF UNIVARIABLE FORWARD MENDELIAN RANDOMIZATION ANALYSIS OF OCCUPATIONAL INCOME AGAINST MENTAL ILLNESSES 62
SUPPLEMENTARY TABLE 14: RESULTS OF BIDIRECTIONAL MR OF OCCUPATIONAL INCOME (OI) AGAINST MENTAL HEALTH TRAITS 63
PLOTS - FORWARD ANALYSES 66
Supplementary Figure 56: scatterplot of occupational income against ADHD 66
Supplementary Figure 57: scatterplot of occupational income against AN 67
Supplementary Figure 58: scatterplot of occupational income against ANX 67
Supplementary Figure 59: scatterplot of occupational income against ASD 68
Supplementary Figure 60: scatterplot of occupational income against BD 68
Supplementary Figure 61: scatterplot of occupational income against MDD 69
Supplementary Figure 62: scatterplot of occupational income against OCD 70

Supplementary Figure 63: scatterplot of occupational income against PTSD 70
Supplementary Figure 64: scatterplot of occupational income against SZ....... 71
Supplementary Figure 65: leave-one-out analysis of occupational income against ADHD....... 71
Supplementary Figure 66: leave-one-out analysis of occupational income against AN....... 72
Supplementary Figure 67: leave-one-out analysis of occupational income against ANX 73
Supplementary Figure 68: leave-one-out analysis of occupational income against ASD....... 73
Supplementary Figure 69: leave-one-out analysis of occupational income against BD....... 74
Supplementary Figure 70: leave-one-out analysis of occupational income against MDD....... 75
Supplementary Figure 71: leave-one-out analysis of occupational income against OCD 75
Supplementary Figure 72: leave-one-out analysis of occupational income against PTSD 76
Supplementary Figure 73: leave-one-out analysis of occupational income against SZ....... 77
PLOTS - BACKWARD ANALYSES 77
Supplementary Figure 74: scatterplot of ADHD against occupational income....... 78
Supplementary Figure 75: scatterplot of AN against occupational income....... 78
Supplementary Figure 76: scatterplot of BD against occupational income....... 79
Supplementary Figure 77: scatterplot of SZ against occupational income....... 79
Supplementary Figure 78: leave-one-out analysis of ADHD against occupational income....... 80
Supplementary Figure 79: leave-one-out analysis of AN against occupational income....... 81
Supplementary Figure 80: leave-one-out analysis of BD against occupational income....... 81
Supplementary Figure 81: leave-one-out analysis of SZ against occupational income....... 82
SUPPLEMENTARY TABLE 15: CAUSE RESULTS OF THE RELATIONS BETWEEN OCCUPATIONAL INCOME AND MENTAL ILLNESS 82
SUPPLEMENTARY TABLE 16: RESULTS OF UNIVARIABLE BIDIRECTIONAL MENDELIAN RANDOMIZATION OF OCCUPATIONAL INCOME AGAINST MENTAL ILLNESSES, AFTER STEIGER FILTERING 85
UNIVARIABLE MENDELIAN RANDOMIZATION OF SOCIAL DEPRIVATION AND MENTAL ILLNESSES....... 86
SUPPLEMENTARY TABLE 17: ODDS RATIO OF UNIVARIABLE FORWARD MENDELIAN RANDOMIZATION ANALYSIS OF SOCIAL DEPRIVATION AGAINST MENTAL ILLNESSES 86
SUPPLEMENTARY TABLE 18: RESULTS OF BIDIRECTIONAL MR OF SOCIAL DEPRIVATION (SD) AGAINST MENTAL HEALTH TRAITS....... 87
PLOTS - FORWARD ANALYSES 90
Supplementary Figure 82: scatterplot of social deprivation against ADHD 90
Supplementary Figure 83: scatterplot of social deprivation against AN 91
Supplementary Figure 84: scatterplot of social deprivation against ANX....... 91
Supplementary Figure 85: scatterplot of social deprivation against ASD 92
Supplementary Figure 86: scatterplot of social deprivation against BD 92
Supplementary Figure 87: scatterplot of social deprivation against MDD 93
Supplementary Figure 88: scatterplot of social deprivation against OCD....... 94
Supplementary Figure 89: scatterplot of social deprivation against PTSD....... 94
Supplementary Figure 90: scatterplot of social deprivation against SZ 95
Supplementary Figure 91: leave-one-out analysis of social deprivation against ADHD 95
Supplementary Figure 92: leave-one-out analysis of social deprivation against AN 96
Supplementary Figure 93: leave-one-out analysis of social deprivation against ANX....... 97
Supplementary Figure 94: leave-one-out analysis of social deprivation against ASD 97
Supplementary Figure 95: leave-one-out analysis of social deprivation against BD 98
Supplementary Figure 96: leave-one-out analysis of social deprivation against MDD 99
Supplementary Figure 97: leave-one-out analysis of social deprivation against OCD....... 99
Supplementary Figure 98: leave-one-out analysis of social deprivation against PTSD....... 100
Supplementary Figure 99: leave-one-out analysis of social deprivation against SZ 101
PLOTS - BACKWARD ANALYSES 101
Supplementary Figure 100: scatterplot of ADHD against social deprivation 102
Supplementary Figure 101: scatterplot analysis of AN against social deprivation 102
Supplementary Figure 102: scatterplot analysis of BD against social deprivation....... 103
Supplementary Figure 103: scatterplot of SZ against social deprivation 103
Supplementary Figure 104: leave-one-out analysis of ADHD against social deprivation 104
Supplementary Figure 105: leave-one-out analysis of AN against social deprivation 104

Supplementary Figure 106: leave-one-out analysis of BD against social deprivation 105
Supplementary Figure 107: leave-one-out analysis of SZ against social deprivation 106
Supplementary Table 19: CAUSE results of the relations between social deprivation and mental illness 106
Supplementary Table 20: Results of univariable bidirectional Mendelian Randomization of social deprivation against mental illnesses, after Steiger filtering 109
UNIVARIABLE MENDELIAN RANDOMIZATION OF HOUSEHOLD INCOME LEVELS AND MENTAL ILLNESSES 109
Supplementary Table 21: results of univariable bidirectional Mendelian randomization of Low Household income (LHI) against mental illnesses 109
Supplementary Table 22: results of univariable bidirectional Mendelian randomization of Low-Mid Household income (LMHI) against mental illnesses 112
Supplementary Table 23: results of univariable bidirectional Mendelian randomization of Mid-High Household income (MHHI) against mental illnesses 114
Supplementary Table 24: results of univariable bidirectional Mendelian randomization of High Household income (HHI) against mental illness 116
Supplementary Table 25: Odds Ratio of univariable bidirectional Mendelian randomization analysis of household income levels against mental illnesses 119
Supplementary Table 26: Results of univariable bidirectional Mendelian randomization analysis of household income levels against mental illnesses, after Steiger filtering 122
UNIVARIABLE MENDELIAN RANDOMIZATION OF COGNITIVE ABILITIES AND MENTAL ILLNESSES 124
Supplementary Table 27: Odds Ratio of univariable forward Mendelian randomization analysis of cognitive abilities against mental illnesses 124
Supplementary Table 28: results of bidirectional MR of cognitive abilities against mental illness 125
Plots - Forward analyses 128
Supplementary Figure 108: scatterplot of cognitive abilities against ADHD 128
Supplementary Figure 109: scatterplot of cognitive abilities against AN 129
Supplementary Figure 110: scatterplot of cognitive abilities against ANX 129
Supplementary Figure 111: scatterplot of cognitive abilities against ASD 130
Supplementary Figure 112: scatterplot of cognitive abilities against BD 131
Supplementary Figure 113: scatterplot of cognitive abilities against MDD 131
Supplementary Figure 114: scatterplot of cognitive abilities against OCD 132
Supplementary Figure 115: scatterplot of cognitive abilities against PTSD 132
Supplementary Figure 116: scatterplot of cognitive abilities against SZ 133
Supplementary Figure 117: leave-one out analysis of cognitive abilities against ADHD 134
Supplementary Figure 118: leave-one out analysis of cognitive abilities against AN 134
Supplementary Figure 119: leave-one out analysis of cognitive abilities against ANX 135
Supplementary Figure 120: leave-one out analysis of cognitive abilities against ASD 136
Supplementary Figure 121: leave-one out analysis of cognitive abilities against BD 136
Supplementary Figure 122: leave-one-out analysis of cognitive abilities against MDD 137
Supplementary Figure 123: leave-one out analysis of cognitive abilities against OCD 138
Supplementary Figure 124: leave-one out analysis of cognitive abilities against PTSD 138
Supplementary Figure 125: leave-one-out analysis of cognitive abilities against SZ 139
Plots - Backward analyses 139
Supplementary Figure 126: scatterplot of ADHD against cognitive abilities 140
Supplementary Figure 127: scatterplot of AN against cognitive abilities 140
Supplementary Figure 128: scatterplot of BD against cognitive abilities 141
Supplementary Figure 129: scatterplot of SZ against cognitive abilities 142
Supplementary Figure 130: leave-one-out analysis of ADHD against cognitive abilities 142
Supplementary Figure 131: leave-one-out analysis of AN against cognitive abilities 143
Supplementary Figure 132: leave-one-out analysis of BD against cognitive abilities 143
Supplementary Figure 133: leave-one-out analysis of SZ against cognitive abilities 144
Supplementary Table 29: CAUSE results of the relations between cognitive abilities and mental illnesses 144

SUPPLEMENTARY TABLE 30: RESULTS OF UNIVARIABLE BIDIRECTIONAL MENDELIAN RANDOMIZATION OF COGNITIVE ABILITIES AGAINST MENTAL ILLNESSES, AFTER STEIGER FILTERING 147

MULTIVARIABLE MENDELIAN RANDOMIZATION OF POVERTY INDICATORS AND COGNITIVE ABILITIES AGAINST MENTAL ILLNESS 148

SUPPLEMENTARY TABLE 31: MULTIVARIABLE MENDELIAN RANDOMIZATION RESULTS OF HOUSEHOLD INCOME AND COGNITIVE ABILITIES ON MENTAL ILLNESS 148
SUPPLEMENTARY TABLE 32: MULTIVARIABLE MENDELIAN RANDOMIZATION RESULTS OF OCCUPATIONAL INCOME AND COGNITIVE ABILITIES ON MENTAL ILLNESS 148
SUPPLEMENTARY TABLE 33: MULTIVARIABLE MENDELIAN RANDOMIZATION RESULTS OF SOCIAL DEPRIVATION AND COGNITIVE ABILITIES ON MENTAL ILLNESS 149

FREQUENTLY ASKED QUESTIONS (FAQ) 150

WHAT WAS YOUR GOAL WITH THIS STUDY? 150
IS THE RELATION BETWEEN POVERTY AND MENTAL HEALTH NOT ALREADY OBVIOUS? 150
HOW DO YOU FIND EVIDENCE OF CAUSALITY? 150
WHAT DO YOU MEAN WHEN YOU TALK ABOUT ‘POVERTY’? 151
WHAT DID YOU DO IN THIS STUDY? 152
WHAT IS MEANT BY THE GENETIC ARCHITECTURE OF POVERTY, AND HOW DID YOU IDENTIFY GENETIC VARIATIONS RELATED TO POVERTY? 152
WHAT DID YOU FIND? 153
HOW MAY POVERTY AND MENTAL ILLNESS BE RELATED? 153
WHY IS THIS RELEVANT? 153
TO WHOM DO THE RESULTS APPLY? 153
ARE THE ASSOCIATIONS NOT JUST DUE TO “DYNASTIC EFFECTS”? 154
DOES THIS STUDY IMPLY THAT POVERTY AND MENTAL ILLNESSES ARE DETERMINED AT BIRTH? 154
DO GENES DETERMINE WHETHER YOU BECOME POOR? 154
DOES THIS STUDY IMPLY THAT MENTALLY ILL PEOPLE ARE NOT ABLE TO EARN THE MONEY THEY NEED TO LIVE? 155
WHAT ARE THE PRACTICAL APPLICATIONS OF THIS RESEARCH? 155

Methods details

Household income and social deprivation GWAS

Samples

Among 502,408 UK biobank participants, 440,964 samples of recent European ancestry that have genetic information, have identical reported sex as genotype inferred sex, not have sex chromosome aneuploidy, not have been detected as extreme outliers of heterozygosity and missingness, and have a genotyping rate greater than 0.9 were retained in our analysis. European ancestry was identified as self-reported whites of which each of their first six principal components (PC) was within its corresponding 99.7% confidence intervals (i.e., mean ± 3 standard deviations).

Genotype and imputation data

A total number of 784,256 and 93,095,624 autosomal variants are available in UK biobank genotype and imputation data, respectively. For genotype data, 581,097 variants with MAF > 0.01, call rate > 0.9, and HWE-p value > 10^{-15} in the European subset were kept. For imputation data, 25,590,374 variants with MAF >= 0.0005 and INFO >= 0.3 in the whole population were retained for the GWAS.

Phenotype

Six phenotypes were extracted for study samples. Social deprivation is a continuous trait with N = 440,350. Household income is a five-level category trait with N = 379,598 ('Do not known' and 'Prefer not to answer' removed). Another four binary traits were derived from household income. Low income (coded 1 for level 1 and 0 for the rest), LowMid income (coded 1 for levels 1 and 2 and 0 for the rest), MidHigh income (coded 1 for levels 4 and 5 and 0 for the rest), and High income (coded 1 for level 5 and 0 for the rest).

GWAS using Regenie

GWAS was conducted in Regenie v3.1.3, a two-step GWAS software that accounts for sample relatedness and population structure. In the first step, a whole genome regression model was fit to each trait using 581,097 post-QC genotype variants. In the second step, association test was performed for each of the 25,590,374 post-QC imputed variant using a LOCO (leave-one-chromosome out) scheme. The per-chromosome LOCO genomic predictions produced in the first step were fitted in the second step to account for sample relatedness and population structure. In addition, sex, age at assessment, assessment centers, genotyping array, genotyping bathes, and the first 40 PCs were fitted as covariates in both steps. For binary phenotypes, firth logistic regression test was performed in the second step to account for unbalanced case-control ratio. Afterwards, variants with MAF < 0.0005 and INFO < 0.3 in each subset were removed, resulting in 20,408,331 final variants for household income related phenotypes and 20,413,590 for Townsend score.

Cognitive ability GWAS

Summary statistics on cognitive ability were taken from Hill et al 2019[1]. There are two principal stages to this meta-analysis. First, publicly available GWAS summary statistics from Sniekers et al.[2] were meta-analysed with 120,934 participants from UK Biobank who took the test of verbal numerical reasoning using a sample size weighted meta-analysis[3]. This resulted in a sample size of 199,242. Second, data from these 199,242 participants was meta-analysed with the publicly available GWAS summary statistics on educational attainment from Okbay et al.[4] using multi trait analysis of genome-wide association studies (MTAG[5]). MTAG allows

for the meta-analysis of genetically correlated traits in order to increase the statistical power in any one of those traits. In the case of the Hill et al. data, cognitive ability was meta-analyzed with education to in order to detect associations specific to the trait of cognitive ability.

Table showing the cohorts and cognitive ability tests used in Hill et al.

Cognitive ability (Hill et al.[1])		
Cohorts	N	Phenotype
UK Biobank	120,934	Verbal numerical reasoning
Cognitive ability (Sniekers et al.[2])		
Cohorts	N	Phenotype
UK Biobank	54,119	Verbal numerical reasoning (fluid intelligence using SES as an additional covariate)
LBC1921	464	Moray House Test No.12
LBC1936	947	Moray House Test No.12
Brisbane Adolescent Twin Study	1,752	IQ scores derived using Multi-dimensional Aptitude Battery
Western Australia Pregnancy Cohort	936	g-factor
Twins Early Development	2,825	g-factor
Erasmus Rucphen Family Study	1,076	g-factor
Generation R Study	3,701	Snijders-Ooman non-verbal Intelligence Test
The Harvard/Union Study	389	IQ score
The Minnesota Center for Twin and Family Research Study	3,367	Wechsler Adult Intelligence Scale-Revised and the Wechsler Intelligence Scale for Children Revised
Swedish Twin Registry	3,215	g-factor
ALSPAC Children	5,517	Wechsler Intelligence Scale for Children III

References

1. Hill, W.D. *et al.* A combined analysis of genetically correlated traits identifies 187 loci and a role for neurogenesis and myelination in intelligence. *Molecular Psychiatry* **24**, 169-181 (2019).
2. Sniekers, S. *et al.* Genome-wide association meta-analysis of 78,308 individuals identifies new loci and genes influencing human intelligence. *Nature Genetics* **49**, 1107 (2017).
3. Willer, C.J., Li, Y. & Abecasis, G.R. METAL: fast and efficient meta-analysis of genomewide association scans. *Bioinformatics* **26**, 2190-2191 (2010).
4. Okbay, A. *et al.* Genome-wide association study identifies 74 loci associated with educational attainment. *Nature* **533**, 539 (2016).
5. Turley, P. *et al.* Multi-trait analysis of genome-wide association summary statistics using MTAG. *Nature Genetics* **50**, 229-237 (2018).

MR-PRESSO

The Mendelian randomization pleiotropy residual sum and outlier (MR-PRESSO) test identifies possible bias from horizontal pleiotropy. The test consists of three parts. (1) the MR-PRESSO global test which detects horizontal pleiotropy. (2) the outlier corrected causal estimate which corrects for the detected horizontal pleiotropy and (3) the MR-PRESSO distortion test which estimates if the causal estimate is significantly different (at $p<0.05$) after adjustment for outliers. We conduct all three stages (with the argument NbDistribution=1000. namely using1000 simulation form the null distribution to compute empirical p-values) and present the outlier adjusted causal estimates (OACE) when both global and distortion tests are significant.

CAUSE

In addition to MR-Egger and MR-PRESSO. we accounted for false positive due to horizontal pleiotropy using Causal Analysis Using Summary Effect Estimates (CAUSE).
CAUSE grounds on estimating two models. One under the assumption that the relation between the instrument and the outcome is due to a pleiotropic effect (i.e., the shared model). and the second assuming that the relation is due to causal effect (i.e., the causal model). CAUSE also provides a test that the posteriors estimated under the causal model fit the data significantly better than posteriors estimated under the sharing model. If this is the case, it is possible to conclude that the data are consistent with a causal effect.
CAUSE consists in 4 steps: (1) format the data for use with CAUSE; (2) calculate nuisance parameters; (3) LD pruning; (4) fit CAUSE. We estimated nuisance parameters setting a random subset of 1.000.000 variants and performed LD pruning setting R^2 threshold to 0.01 and p-value threshold to 0.001.

Multivariable GWAS of the latent poverty factor

Supplementary Table 1: factor loading of each poverty indicator used for the estimation of the latent poverty factor

Regression	Unstandardized B (SE)	Standardized B (SE)	p-value
F~HI	0.280 (0.007)	1,00 (0.028)	**5.061×10^{-295}**
F~SD	-0.127 (0.004)	-0.733 (0.025)	**5.230×10^{-198}**
F~OI	0.261 (0.008)	0.862 (0.025)	**6.194×10^{-260}**

Abbreviations: F: common factor; HI: household income; SD: social deprivation; OI: occupational income; B: linear regression coefficient; SE: standard error.

Legend: Linear regression was used for testing the factor loadings of each indicator on the common factor. The p-values are two-sided and not adjusted for multiple testing, therefore p-value<0.05 was considered significant (and reported in bolded text).

Supplementary Table 2: Summary statistics of the common poverty factor, household income, social deprivation, and occupational income

	Mean Chi^2	LDSC intercept (SE) [N SNPs]	Heritability h^2 % (SE)
HI	1.5669	1.0426 (0.0099) [1165506]	7.08% (0.0031) **$p=4.33\times10^{-113}$**
SD	1.3379	1.0423 (0.0081) [1165534]	3.01% (0.0015) **$p=2.15\times10^{-93}$**
OI	1.5145	1.0015 (0.0092) [1177612]	9.14% (0.004) **$p=4.59\times10^{-115}$**
Common factor: P	1.7301	0.9883 (0.0108) [1158117]	8.38% (0.0031) **$p=4.24\times10^{-164}$**

Abbreviations: LDSC: Linkage Disequilibrium Score Regression; SE: standard error; P: poverty; HI: household income; SD: social deprivation; OI: occupational income.

Legend: Mean Chi^2 measures the overall strength of association between genetic variants and the phenotype of interest; a high Mean Chi^2 value indicates that there are many genetic variants that are strongly associated with the trait. The Linkage Disequilibrium Score Regression (LDSC) intercept captures the contribution of factors other than polygenicity (such as population stratification) to inflation in association test statistic. Narrow sense heritability (h^2) is a measure of the proportion of phenotypic variation that is attributable to genetic variation. The p-values are

calculated from two-sided chi^2 test and are not adjusted for multiple testing, therefore p-value<0.05 was considered significant (and reported in bolded text).

Supplementary Table 3: genetic correlation (rg) between the common factor poverty, household income, social deprivation, occupational income, and cognitive abilities

	**P**				
**P**	1.00	**HI**			
**HI**	0.9826 (0.0398)	1.00	**SD**		
**SD**	-0.8007 (0.0344)	-0.7712 (0.0345)	1.00	**OI**	
**OI**	0.9509 (0.0372)	0.9067 (0.0391)	-0.6318 (0.0325)	1.00	**CA**
**CA**	0.7396 (0.0289)	0.7019 (0.0315)	-0.4092 (0.0276)	0.8147 (0.0334)	1.00

Abbreviations: P: poverty; HI: household income; SD: social deprivation; OI: occupational income; CA: cognitive abilities.
Legend: genetic correlations are presented as rg(standard error)

Supplementary Table 4: Results of bidirectional Mendelian Randomization of Cognitive Abilities against Poverty

**MR**	**N SNP**	**IVW, B (95% CI)**	**IVW p-value**	**WM, B (95% CI)**	**WM p-value**	**MR-Egger, B (95% CI)**	**MR-Egger p-value**	**Egger intercept p-value**	**Steiger Test p-value†**	**MR-PRESSO**	**Mean F**
Fw: CA on P	133	-0.390 (-0.408; -0.372)	**$<1^{-1000}$**	-0.352 (-0.387; -0.317)	**1.48×10^{-100}**	-0.458 (-0.591; -0.326)	**3.83×10^{-10}**	0.303	**1.08×10^{-256}**	DT; p=0.851	44.0
Bw: P on CA	78	-0.274 (-0.288; -0.261)	**$<1^{-1000}$**	-0.237 (-0.264; -0.209)	**8.82×10^{-65}**	-0.330 (-0.444; -0.215)	**2.91×10^{-7}**	0.337	0.423	DT; p=0.358	40.0

Abbreviations: Fw: forward analysis; Bw: backward analysis; CA: cognitive abilities; P: common factor poverty; MR: mendelian randomization; SNP: single nucleotide polymorphism; IVW: inverse variance weighted (fixed effect); B: effect estimates are unstandardized regression coefficient; 95% CI: 95% confidence interval; WM: weighted median; DT: distortion test; GT: global test.
Legend:

Legend: Poverty is a latent variable built using household income as unit identification, therefore an increase in the indicator's load stands for increased income, therefore the regression coefficients have been flipped to facilitate interpretation of the effect of poverty.
P-value threshold for SNP selection <5e-8.
[a] The Mendelian randomization pleiotropy residual sum and outlier (MR-PRESSO) test identifies possible bias from horizontal pleiotropy. The test consists of three parts, (1) the MR-PRESSO global test which detects horizontal pleiotropy, (2) the outlier corrected causal estimate which corrects for the detected horizontal pleiotropy and (3) the MR-PRESSO distortion test which estimates if the causal estimate is significantly different (at $p<0.05$) after adjustment for outliers. We conduct all three stages (with the argument NbDistribution=1000, namely using1000 simulation form the null distribution to compute empirical p-values) and present the outlier adjusted causal estimates (OACE) when both global and distortion tests are significant.
[b] We did not run Steiger Test if none of the MR analysis resulted significant (NR: not reported in the cell).
[c] Not enough SNP to perform MR (NR: not reported in the cell).
[†] Based on t-test.
All statistical tests were two-sided. The p-values were not adjusted for multiple testing, therefore p-value<0.05 was considered significant (and reported in bolded text).

<u>Plots - Forward analyses</u>

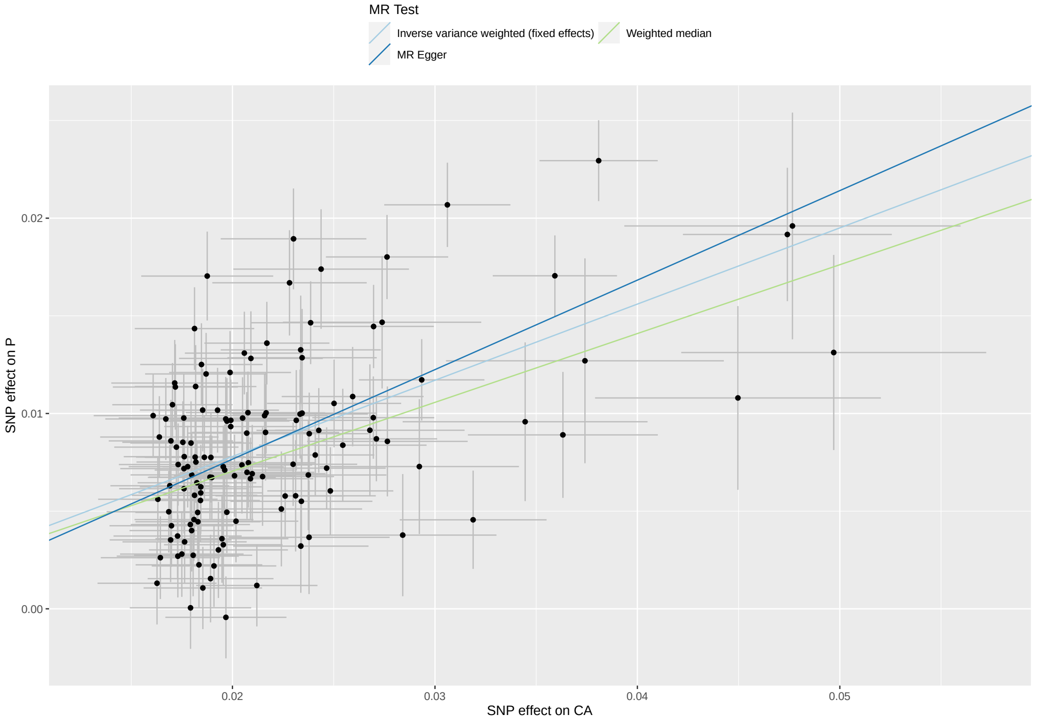

Supplementary Figure 1: scatterplot of cognitive abilities against common factor poverty
Abbreviations: MR: Mendelian randomization; SNP: single nucleotide polymorphism; CA: cognitive abilities; P: common factor poverty.
Legend: n=133 SNPs. Each dot represents a SNP, with the slope of each line corresponding to the estimated MR effect per method.

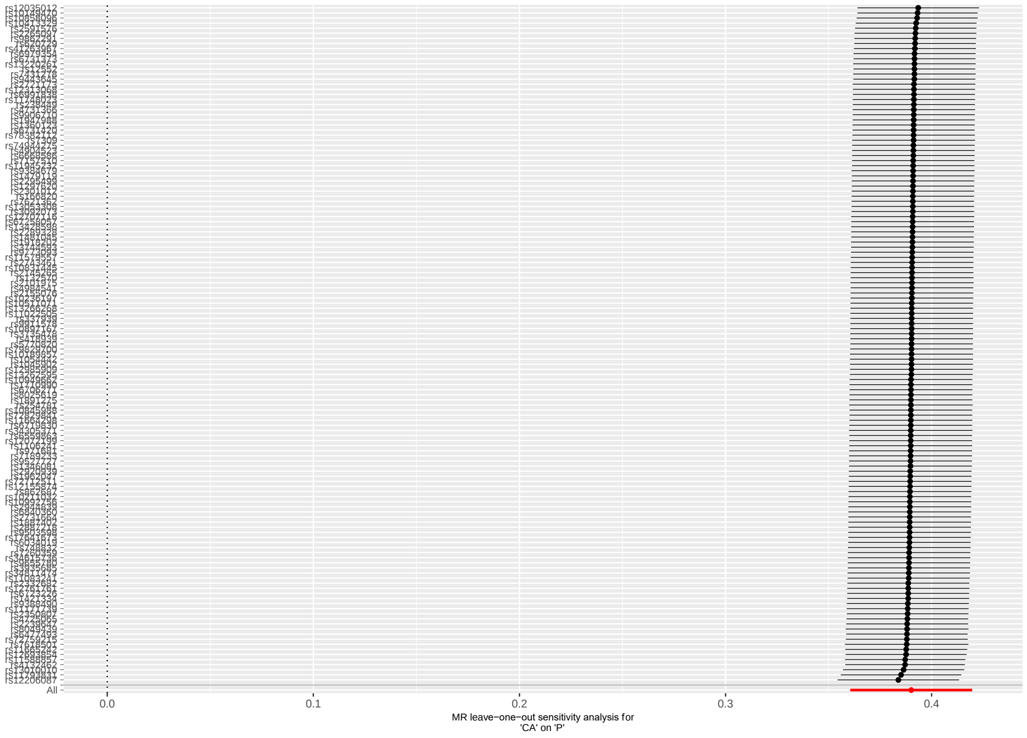

Supplementary Figure 2: leave-one out analysis of cognitive abilities against common factor poverty
Abbreviations: MR: Mendelian randomization; CA: cognitive abilities; P: common factor poverty.
Legend: n=133 SNPs. Forest plot of individual and combined SNP MR estimated effect size. The effect estimates represent the linear effect for mean cognitive ability per one standard deviation (1 SD) increase in the poverty factor, and the error bars represent 95% CIs.

<u>Plots - Backward analyses</u>

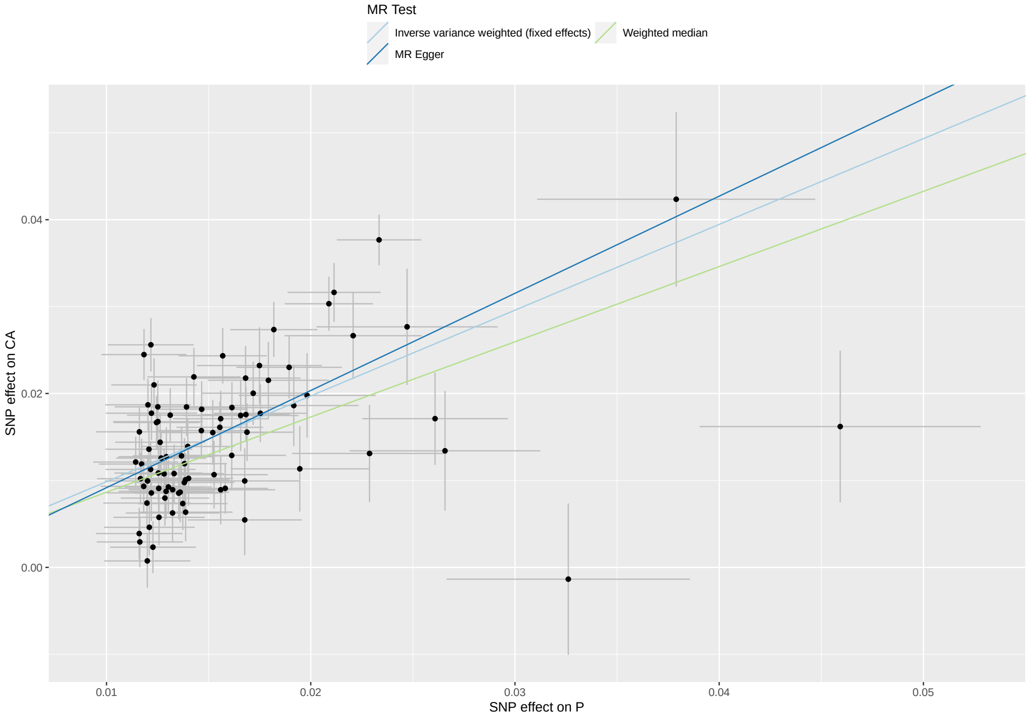

Supplementary Figure 3: scatterplot of common factor poverty against cognitive abilities
Abbreviations: MR: Mendelian randomization; SNP: single nucleotide polymorphism; CA: cognitive abilities; P: common factor poverty.
Legend: n=78 SNPs. Each dot represents a SNP, with the slope of each line corresponding to the estimated MR effect per method.

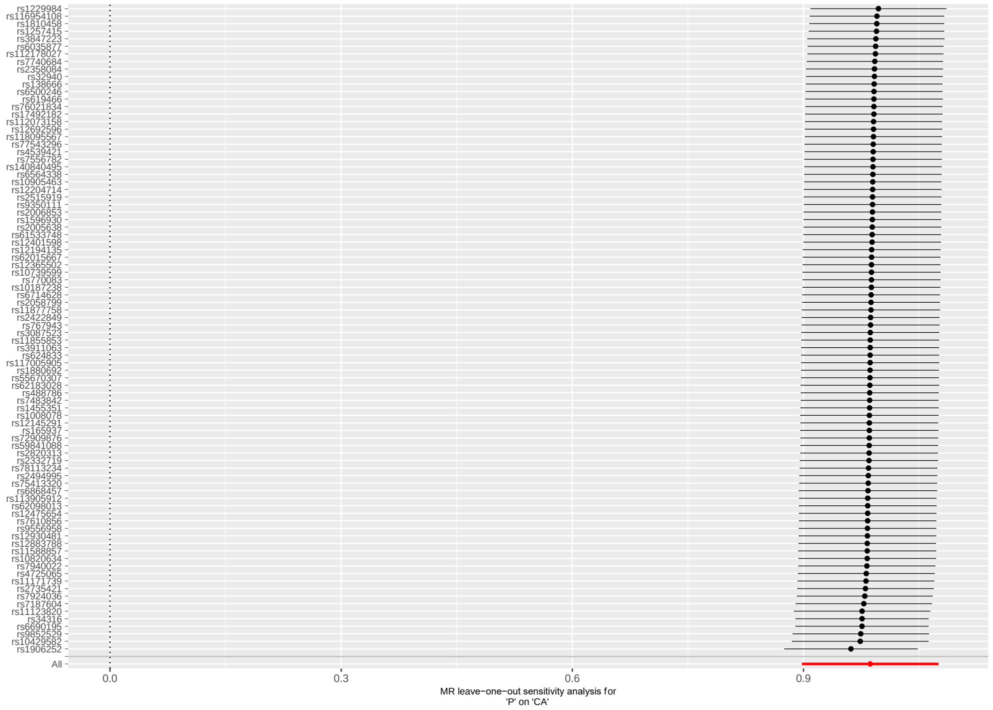

Supplementary Figure 4: leave-one out analysis of common factor poverty against cognitive abilities

Abbreviations: MR: Mendelian randomization; CA: cognitive abilities; P: common factor poverty.

Legend: n=78 SNPs. Forest plot of individual and combined SNP MR estimated effect size. The effect estimates represent the linear effect for the mean poverty factor per one standard deviation (1 SD) increase in the cognitive ability, and the error bars represent 95% CIs.

Univariable Mendelian randomization of poverty and mental illnesses

Supplementary Table 5: results of bidirectional MR of poverty against mental illness

MR	N SNP	IVW, B (95% CI)	IVW p-value	IVW Q(df)	Q p-value[†]	WM, B (95% CI)	WM p-value	MR-Egger, B (95% CI)	MR-Egger p-value	Egger intercept p-value	Steiger Test p-value[‡]	MR-PRESSO	Mean F
Fw: P on ADHD	77	0.330 (0.287; 0.373)	**1.28×10^{-51}**	243 (76)	**2.95×10^{-64}**	0.219 (0.142; 0.295)	**2.28×10^{-8}**	-0.232 (-0.568; 0.104)	0.180	**0.001**	**6.71×10^{-50}**	DT; p=0.418	40.8
Bw: ADHD on P	22	0.402 (0.348; 0.455)	**1.24×10^{-48}**	102 (21)	**1.05×10^{-12}**	0.349 (0.245; 0.453)	**4.48×10^{-11}**	0.143 (-0.569; 0.856)	0.723	0.448	**3.37×10^{-39}**	GT; p=0.853	38.3
Fw: P on AN	77	-0.192 (-0.254; -0.129)	**1.90×10^{-9}**	160 (76)	**5.50×10^{-6}**	-0.191 (-0.290; -0.092)	**1.50×10^{-4}**	-0.420 (-0.858; 0.018)	0.064	0.415	**8.32×10^{-17}**	DT; p=0.786	40.4
Bw: AN on P	2	-0.001 (-0.038; 0.035)	0.947	7 (1)	**0.008**	NR [c]	NR [c]	NR [c]	NR [c]	NR [c]	NR [b]	NR [c]	NR [c]
Fw: P on ANX	79	0.229 (0.101; 357)	**4.59×10^{-4}**	82 (78)	0.362	0.158 (-0.028; 0.344)	0.096	-0.066 (-0.720; 0.587)	0.842	0.369	**3.20×10^{-9}**	GT; p=0.270	40.6
Bw: ANX on P	0	NR [c]	NR [c]	NR [c]	NR [c]	NR [c]	NR [c]	NR [c]	NR [c]	NR [c]	NR [c]	NR [c]	NR [c]
Fw: P on ASD	79	-0.025 (-0.087; 0.038)	0.443	199 (78)	**4.54×10^{-9}**	-0.020 (-0.123; 0.082)	0.698	-0.477 (-0.899; -0.054)	**0.030**	**0.035**	**7.12×10^{-6}**	DT; p=0.981	40.3
Bw: ASD on P	0	NR [c]	NR [c]	NR [c]	NR [c]	NR [c]	NR [c]	NR [c]	NR [c]	NR [c]	NR [c]	NR [c]	NR [c]
Fw: P on BD	79	-0.003 (-0.045; 0.039)	0.889	264 (78)	**9.93×10^{-26}**	-0.033 (-0.045; 0.039)	0.389	-0.247 (-0.631; 0.137)	0.211	0.206	NR [b]	DT; p=0.163	40.3
Bw: BD on P	36	-0.091 (-0.133; -0.049)	**1.93×10^{-5}**	233 (35)	**1.29×10^{-29}**	-0.011 (-0.091; 0.069)	0.783	-0.052 (-0.638; 0.534)	0.863	0.909	**7.24×10^{-224}**	DT; p=0.110	39.2
Fw: P on MDD	78	0.115 (0.074; 0.156)	**3.45×10^{-8}**	150 (77)	**7.91×10^{-6}**	0.089 (0.022; 0.157)	**0.010**	-0.013 (-0.271; 0.246)	0.924	0.325	**3.65×10^{-57}**	DT; p=0.824	40.0

Bw: MDD on P	0	NR [c]	NR [c]	NR [c]	NR [c]	NR [c]	NR [c]	NR [c]	NR [c]	NR [c]	NR [c]	NR [c]	NR [c]
Fw: P on OCD	78	-0.202 (-0.355; -0.049)	**0.010**	96 (78)	0.067	-0.145 (-0.378; 0.088)	0.221	0.296 (-0.443; 1.04)	0.434	0.325	0.051	GT; p=0.141	40.0
Bw: OCD on P	0	NR [c]	NR [c]	NR [c]	NR [c]	NR [c]	NR [c]	NR [c]	NR [c]	NR [c]	NR [c]	NR [c]	NR [c]
Fw: P on PTSD	79	0.140 (0.073; 0.207)	**3.79×10^{-5}**	118 (78)	**0.013**	0.114 (0.010; 0.217)	**0.031**	-0.108 (-0.461; 0.245)	0.511	0.163	**1.52×10^{-94}**	DT; p=0.952	40.3
Bw: PTSD on P	0	NR [c]	NR [c]	NR [c]	NR [c]	NR [c]	NR [c]	NR [c]	NR [c]	NR [c]	NR [c]	NR [c]	NR [c]
Fw: P on SZ	79	0.110 (0.074; 0.145)	**1.36×10^{-9}**	547 (78)	**2.95×10^{-64}**	0.127 (0.056; 0.198)	**4.22×10^{-4}**	0.079 (-0.462; 0.304)	0.689	0.323	**7.79×10^{-77}**	DT; p=0.333	40.3
Bw: SZ on P	176	0.082 (0.061; 0.102)	**1.41×10^{-14}**	706 (175)	**4.84×10^{-63}**	0.050 (0.012; 0.088)	**9.37×10^{-3}**	0.006 (-0.156; 0.169)	0.922	0.352	**$<1^{-1000}$**	DT; p=0.351	45.6

Abbreviations: Fw: forward analysis; Bw: backward analysis; P: poverty; ADHD: attention deficit hyperactivity disorder; AN: anorexia nervosa; ANX: anxiety disorder; ASD: autism spectrum disorder; BD: bipolar disorder; MDD: major depressive disorder; OCD: obsessive-compulsive disorder; PTSD: post-traumatic stress disorder; SZ: schizophrenia; MR: mendelian randomization; SNP: single nucleotide polymorphism; IVW: inverse variance weighted (fixed effect); B: effect estimates are log-odds for binary traits (i.e., for mental illnesses) and unstandardized regression coefficient for continuous traits (i.e., for poverty); 95% CI: 95% confidence interval; Q: Cochran's Q measure of heterogeneity; df: degree of freedom; WM: weighted median; DT: distortion test; GT: global test.

Legend:

P-value threshold for SNP selection <5e-8.

Poverty is a latent variable built using household income as unit identification, therefore an increase in the indicator's load stands for increased income, therefore the regression coefficients have been reversed to facilitate interpretation of the effect of poverty.

[a] The Mendelian randomization pleiotropy residual sum and outlier (MR-PRESSO) test identifies possible bias from horizontal pleiotropy. The test consists of three parts, (1) the MR-PRESSO global test which detects horizontal pleiotropy, (2) the outlier corrected causal estimate which corrects for the detected horizontal pleiotropy and (3) the MR-PRESSO distortion test which estimates if the causal estimate is significantly different (at p<0.05) after adjustment for outliers. We conduct all three stages (with the argument NbDistribution=1000, namely using1000

simulation form the null distribution to compute empirical p-values) and present the outlier adjusted causal estimates (OACE) when both global and distortion tests are significant.

[b] We did not run Steiger Test if none of the MR analysis resulted significant (NR: not reported in the cell).

[c] Not enough SNP to perform MR (NR: not reported in the cell).

[†] Based on chi^2 test.

[‡] Based on t-test.

All statistical tests were two-sided. The p-values were not adjusted for multiple testing, therefore p-value<0.05 was considered significant (and reported in bolded text).

Supplementary Table 6: Odds Ratio of univariable forward Mendelian randomization analysis of poverty against mental illnesses

MR: method	OR (95% CI)	p-value
P → ADHD:		
IVW	1.39 (1.33; 1.45)	**1.28×10^{-51}**
WM	1.24 (1.15; 1.34)	**2.28×10^{-8}**
MR-Egger	0.794 (0.565; 1.10)	0.180
P → AN:		
IVW	0.826 (0.775; 0.877)	**1.90×10^{-9}**
WM	0.826 (0.746; 0.910)	**1.50×10^{-4}**
MR-Egger	0.658 (0.424; 1.02)	0.064
P → ANX:		
IVW	1.26 (1.11; 1.43)	**4.59×10^{-4}**
WM	1.17 (0.971; 1.41)	0.096
MR-Egger	0.935 (0.488; 1.80)	0.842
P → ASD:		
IVW	0.980 (0.917; 1.04)	0.443
WM	0.980 (0.885; 1.09)	0.698
MR-Egger	0.621 (0.406; 0.943)	**0.030**
P → BD:		
IVW	0.997 (0.952; 1.04)	0.889
WM	0.971 (0.901; 1.04)	0.389
MR-Egger	0.775 (0.532; 1.15)	0.211
P → MDD:		
IVW	1.12 (1.08; 1.17)	**3.45×10^{-8}**
WM	1.09 (1.02; 1.17)	**0.010**
MR-Egger	0.990 (0.763; 1.28)	0.924
P → OCD:		
IVW	0.820 (0.699; 0.952)	**0.010**
WM	0.862 (0.685; 1.09)	0.221
MR-Egger	1.34 (0.641; 2.82)	0.434
P → PTSD:		
IVW	1.15 (1.08; 1.23)	**3.79×10^{-5}**
WM	1.12 (1.01; 1.24)	**0.031**
MR-Egger	0.901 (0.630; 1.28)	0.163
P → SZ:		
IVW	1.12 (1.08; 1.16)	**1.41×10^{-14}**
WM	1.14 (1.06; 1.22)	**9.37×10^{-3}**
MR-Egger	0.926 (0.629; 1.36)	0.922

Abbreviations: MR: Mendelian randomization; OR: Odds Ratio; 95% CI: 95% confidence intervals; P: poverty; ADHD: attention deficit hyperactivity disorder; AN: anorexia nervosa; ANX: anxiety disorder; ASD: autism spectrum disorders; BD: bipolar disorder; MDD: major depressive disorder; OCD: obsessive-compulsive disorder; PTSD: post-traumatic stress disorder; SZ: schizophrenia; IVW: inverse variance weighted (fixed effect); WM: weighted median.

Legend: Poverty is a latent variable built using household income as unit identification, therefore an increase in the indicator's load stands for increased income, therefore the ORs have been reversed to facilitate interpretation of the effect of poverty. All statistical tests were two-sided. The p-values were not adjusted for multiple testing, therefore p-value<0.05 was considered significant (and reported in bolded text).

Plots - Forward analyses

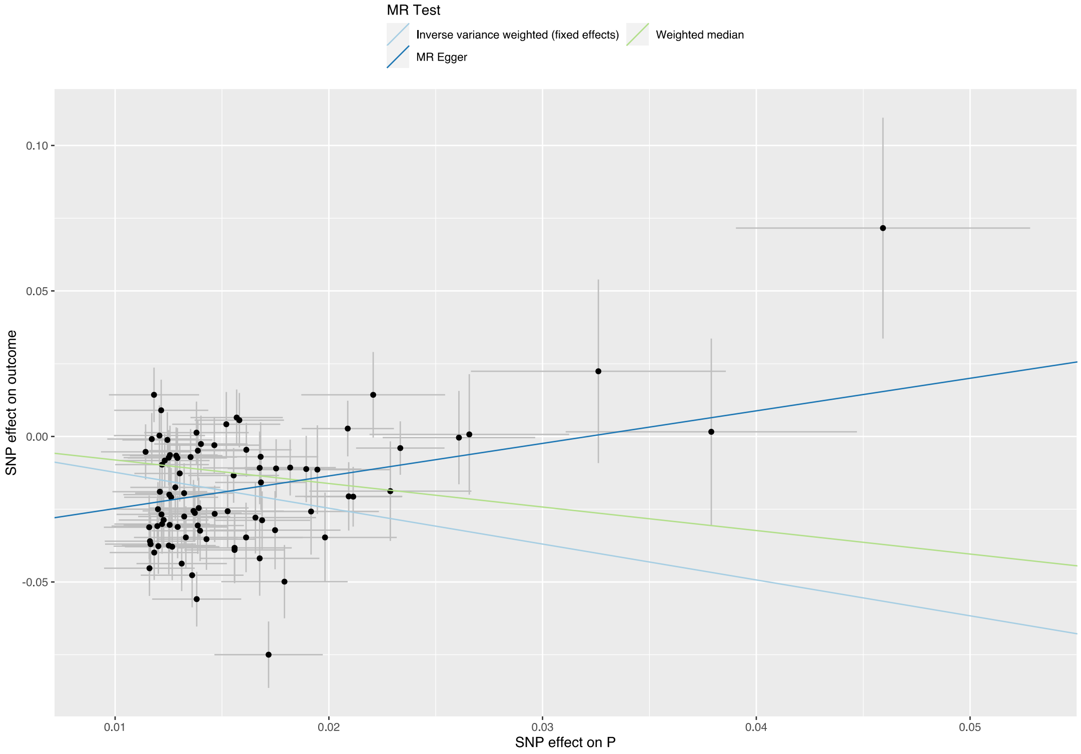

Supplementary Figure 5: scatterplot of poverty against ADHD
Abbreviations: MR: Mendelian randomization; SNP: single nucleotide polymorphism; P: poverty; ADHD: attention deficit hyperactivity disorder.
Legend: n=77 SNPs. Each dot represents a SNP, with the slope of each line corresponding to the estimated MR effect per method.

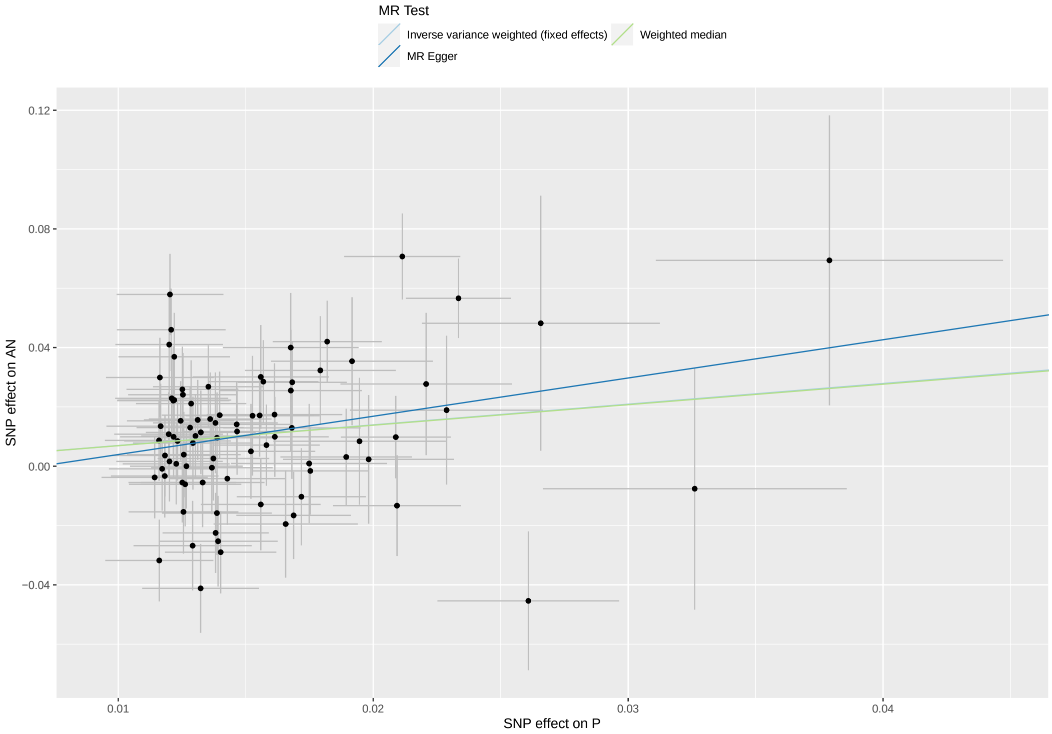

Supplementary Figure 6: scatterplot of poverty against AN
Abbreviations: MR: Mendelian randomization; SNP: single nucleotide polymorphism; P: poverty; AN: anorexia nervosa.
Legend: n=77 SNPs. Each dot represents a SNP, with the slope of each line corresponding to the estimated MR effect per method.

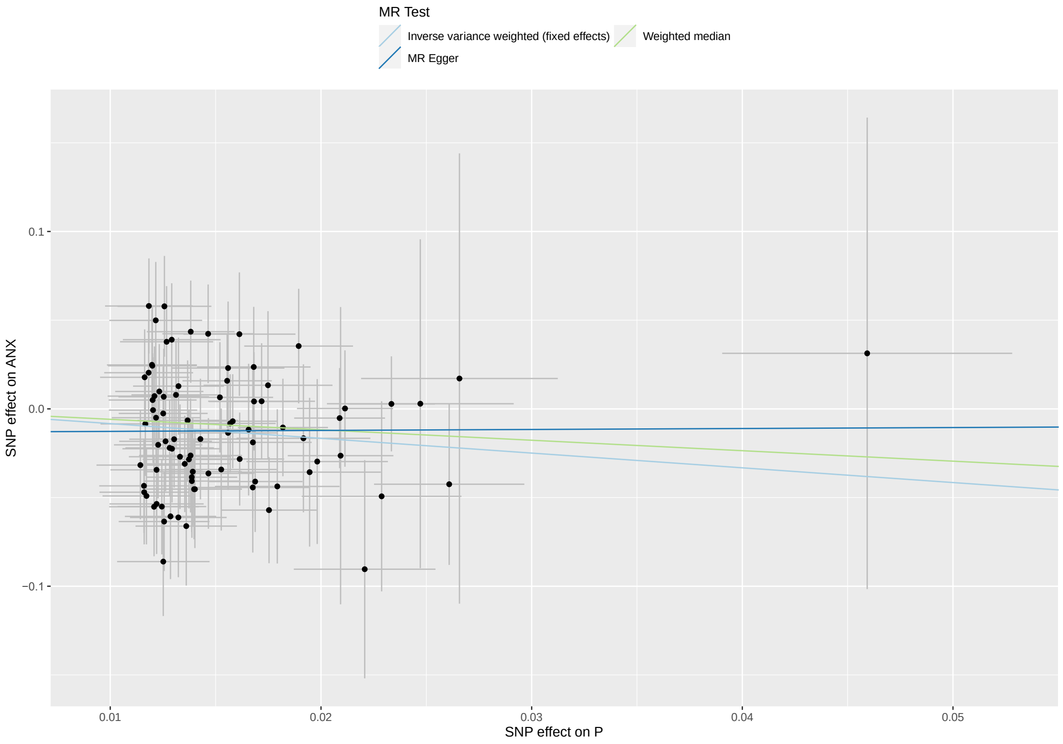

Supplementary Figure 7: scatterplot of poverty against ANX
Abbreviations: MR: Mendelian randomization; SNP: single nucleotide polymorphism; P: poverty; ANX: anxiety disorders.
Legend: n=79 SNPs. Each dot represents a SNP, with the slope of each line corresponding to the estimated MR effect per method.

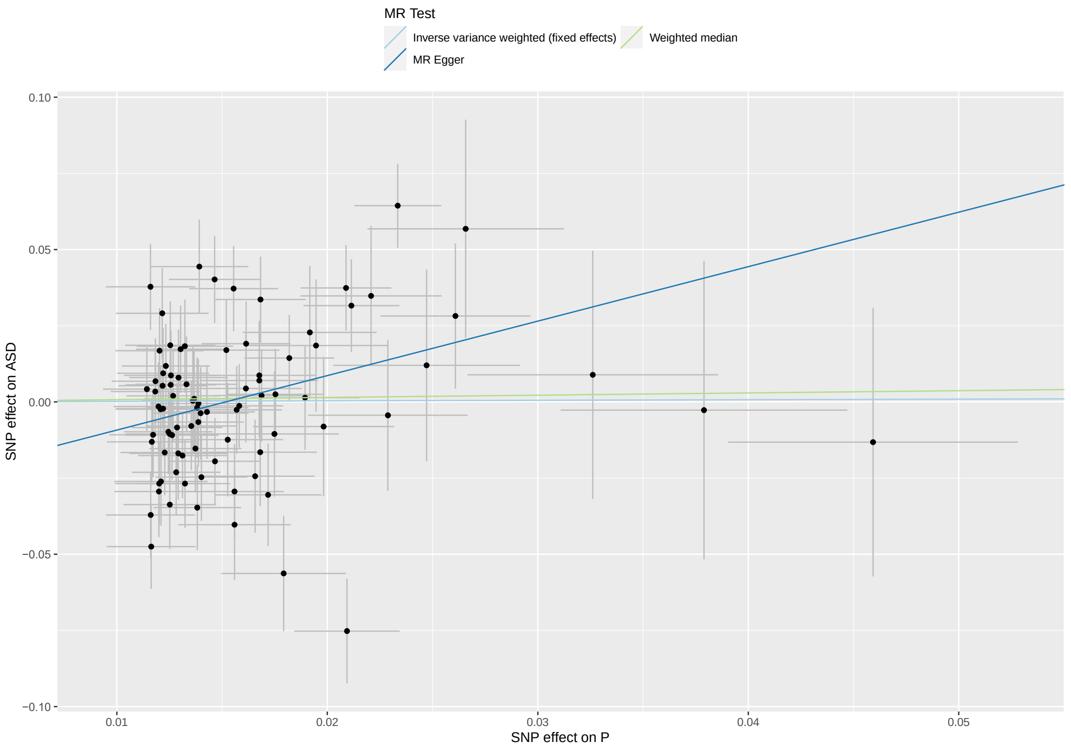

Supplementary Figure 8: scatterplot of poverty against ASD
Abbreviations: MR: Mendelian randomization; SNP: single nucleotide polymorphism; P: poverty; ASD: autism spectrum disorders.
Legend: n=79 SNPs. Each dot represents a SNP, with the slope of each line corresponding to the estimated MR effect per method.

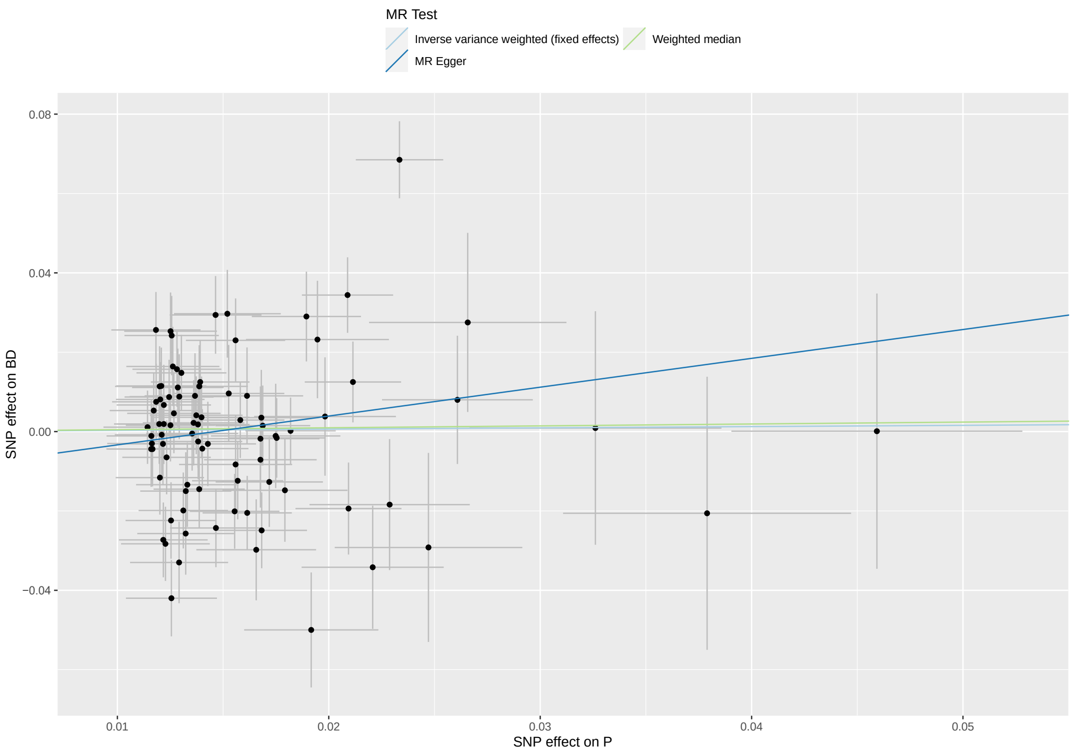

Supplementary Figure 9: scatterplot of poverty against BD
Abbreviations: MR: Mendelian randomization; SNP: single nucleotide polymorphism; P: poverty; BD: bipolar disorder.

Legend: n=79 SNPs. Each dot represents a SNP, with the slope of each line corresponding to the estimated MR effect per method.

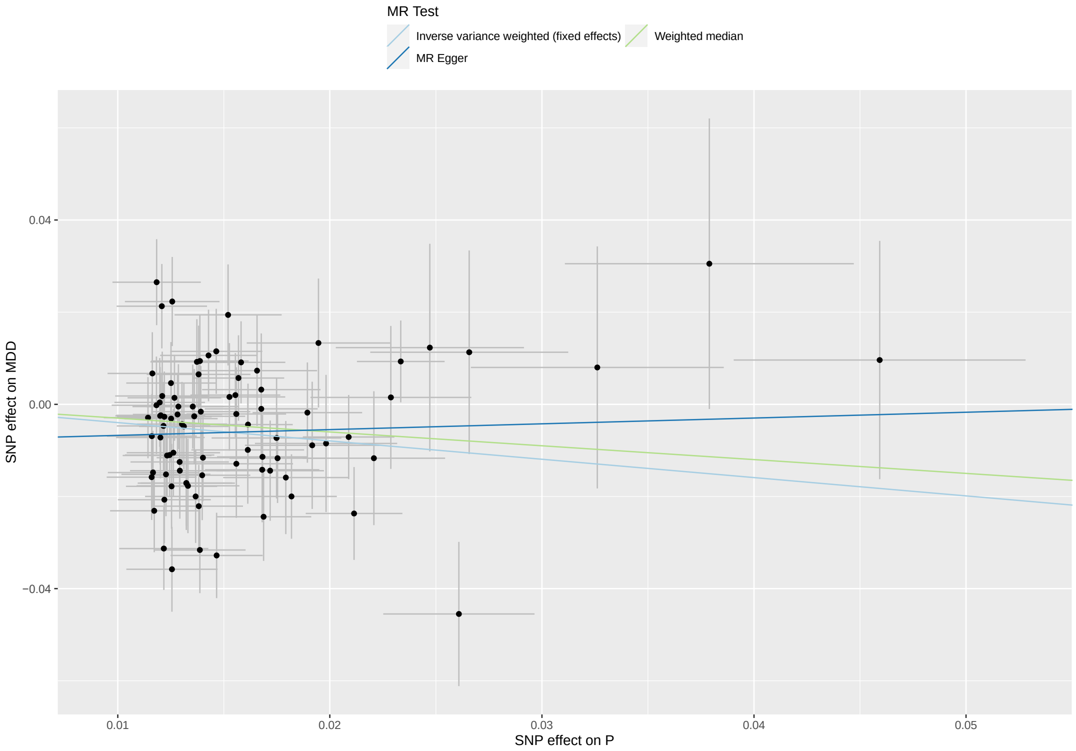

Supplementary Figure 10: scatterplot of poverty against MDD
Abbreviations: MR: Mendelian randomization; SNP: single nucleotide polymorphism; P: poverty; MDD: major depressive disorder.
Legend: n=78 SNPs. Each dot represents a SNP, with the slope of each line corresponding to the estimated MR effect per method.

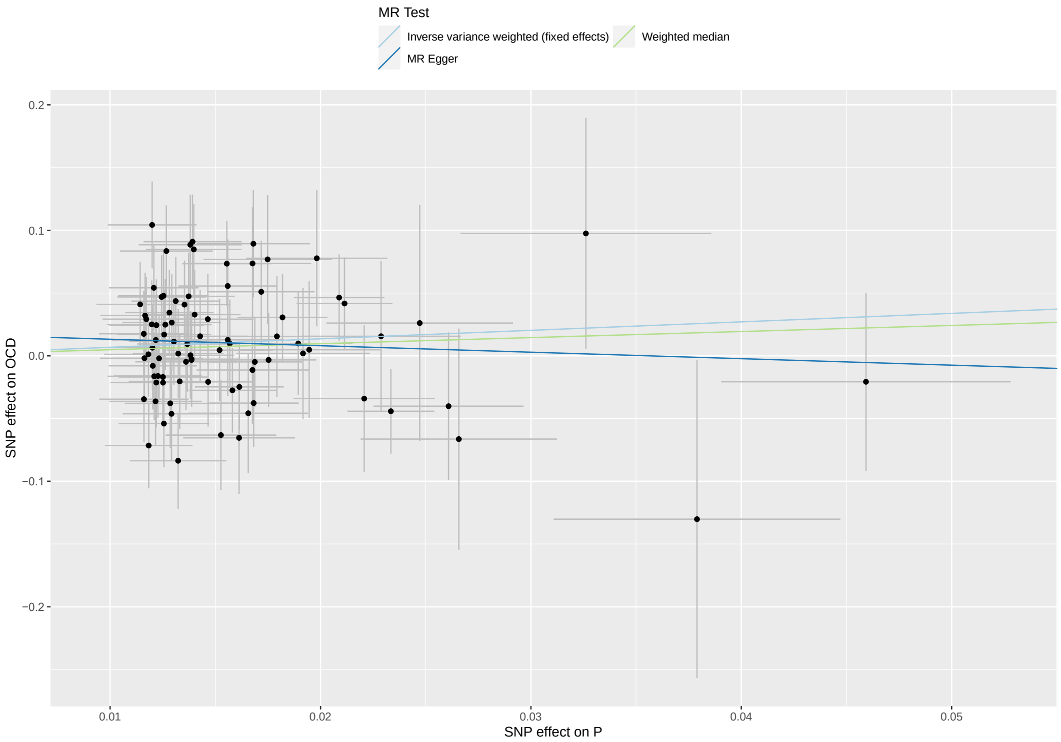

Supplementary Figure 11: scatterplot of poverty against OCD
Abbreviations: MR: Mendelian randomization; SNP: single nucleotide polymorphism; P: poverty; OCD: obsessive-compulsive disorder.
Legend: n=78 SNPs. Each dot represents a SNP, with the slope of each line corresponding to the estimated MR effect per method.

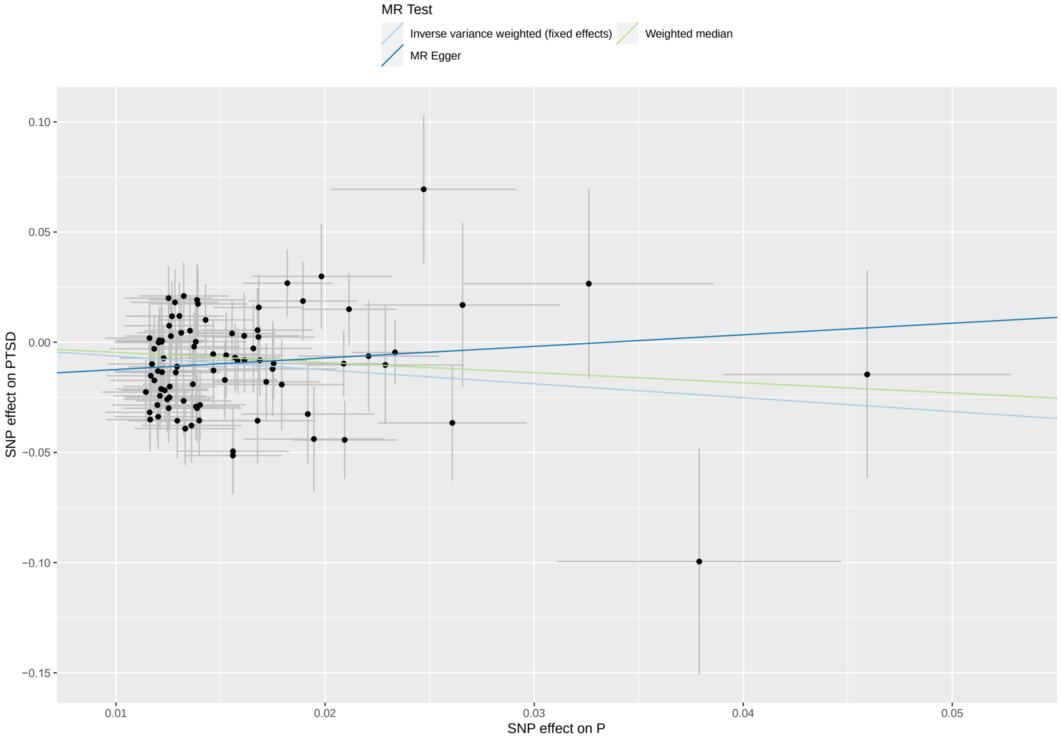

Supplementary Figure 12: scatterplot of poverty against PTSD
Abbreviations: MR: Mendelian randomization; SNP: single nucleotide polymorphism; P: poverty; PTSD: post-traumatic stress disorder.
Legend: n=79 SNPs. Each dot represents a SNP, with the slope of each line corresponding to the estimated MR effect per method.

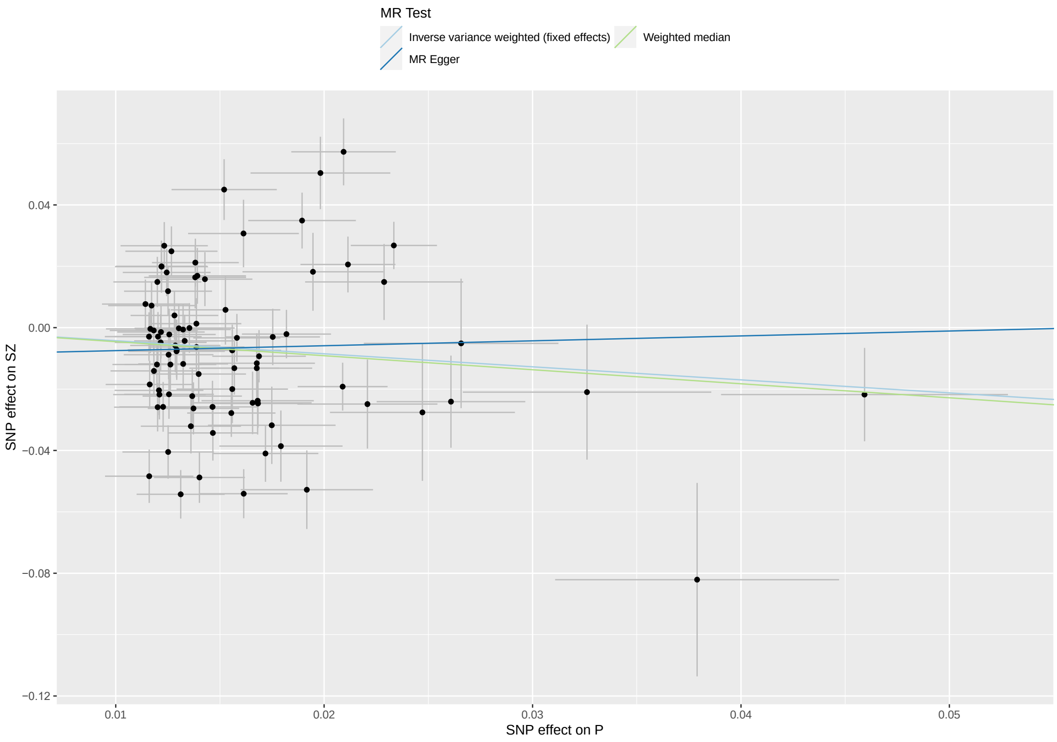

Supplementary Figure 13: scatterplot of poverty against SZ
Abbreviations: MR: Mendelian randomization; SNP: single nucleotide polymorphism; P: poverty; SZ: schizophrenia.
Legend: n=79 SNPs. Each dot represents a SNP, with the slope of each line corresponding to the estimated MR effect per method.

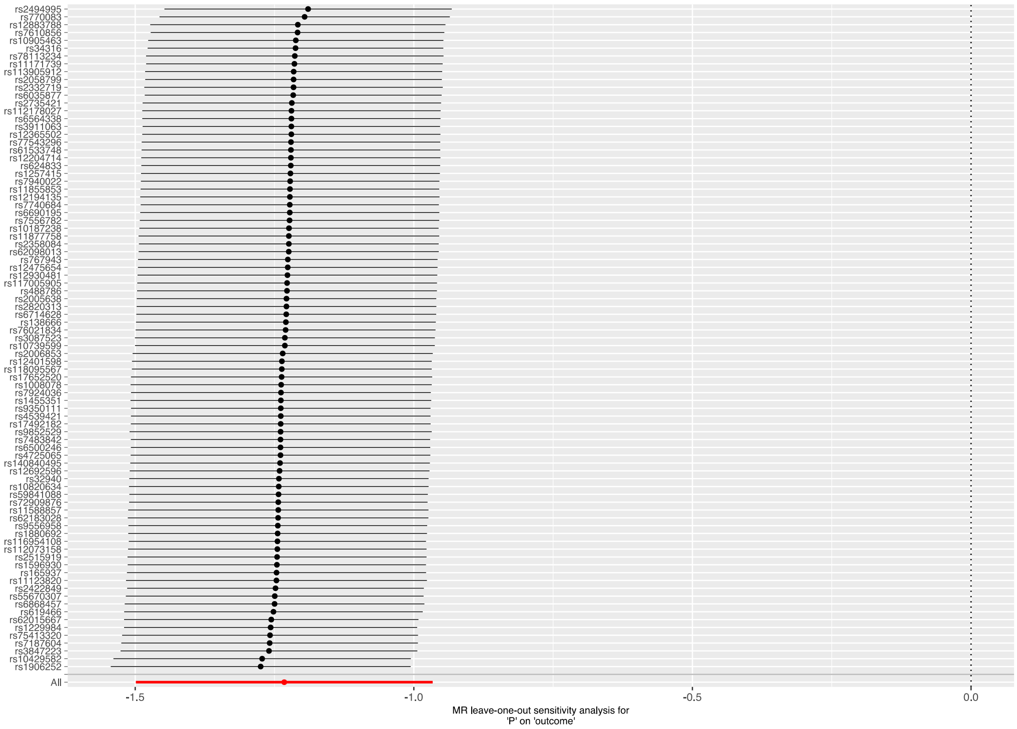

Supplementary Figure 14: leave-one-out analysis of poverty against ADHD
Abbreviations: MR: Mendelian randomization; P: poverty; ADHD: attention deficit hyperactivity disorder.

Legend: n=77 SNPs. Forest plot of individual and combined SNP MR estimated effect size. The effect estimates represent the log-odds for ADHD per one standard deviation (1 SD) increase in the mean poverty factor, and the error bars represent 95% CIs.

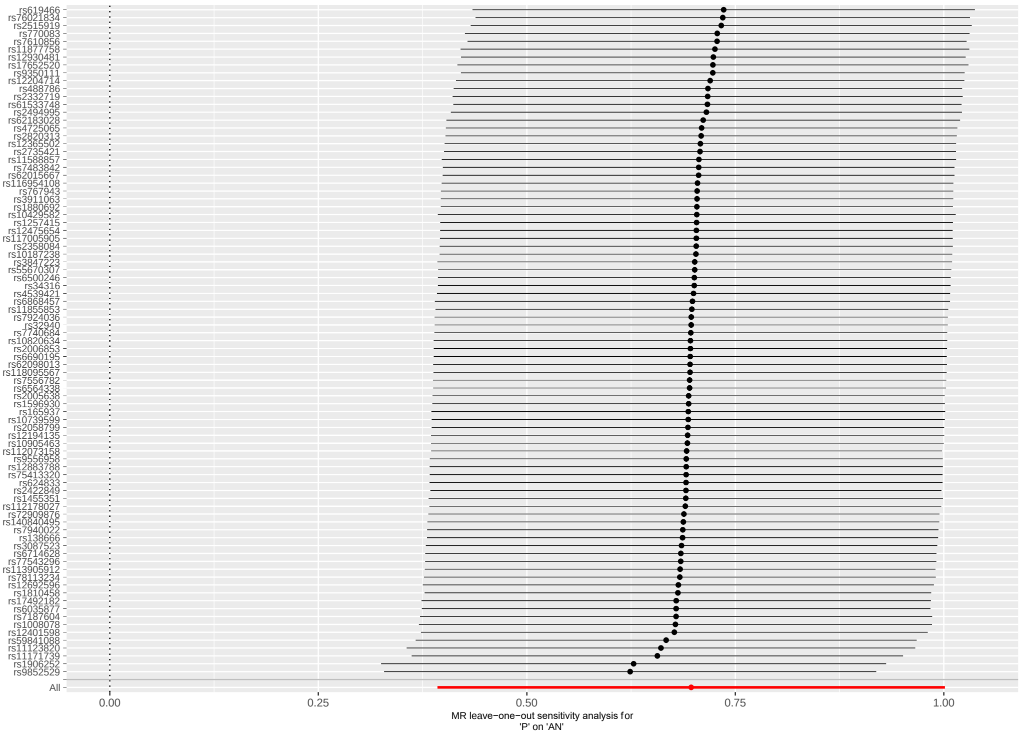

Supplementary Figure 15: leave-one-out analysis of poverty against AN.
Abbreviations: MR: Mendelian randomization; P: poverty; AN: anorexia nervosa.
Legend: n=77 SNPs. Forest plot of individual and combined SNP MR estimated effect size. The effect estimates represent the log-odds for AN per one standard deviation (1 SD) increase in the mean poverty factor, and the error bars represent 95% CIs.

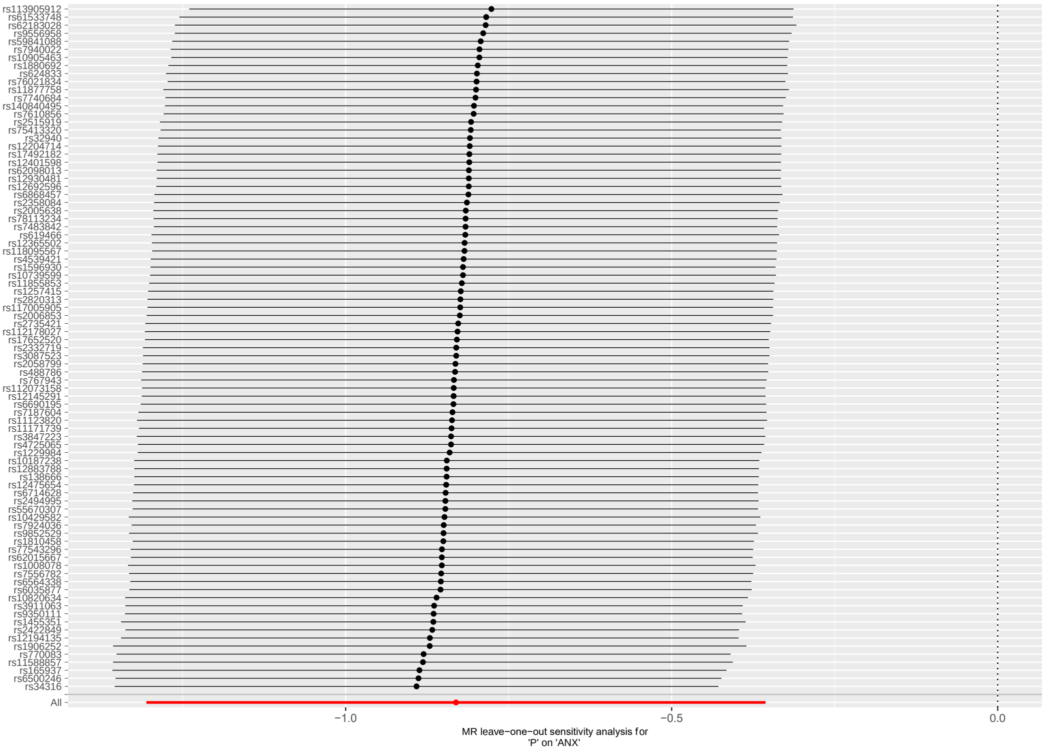

Supplementary Figure 16: leave-one-out analysis of poverty against ANX.
Abbreviations: MR: Mendelian randomization; P: poverty; ANX: anxiety disorders.
Legend: n=79 SNPs. Forest plot of individual and combined SNP MR estimated effect size. The effect estimates represent the log-odds for ANX per one standard deviation (1 SD) increase in the mean poverty factor, and the error bars represent 95% CIs.

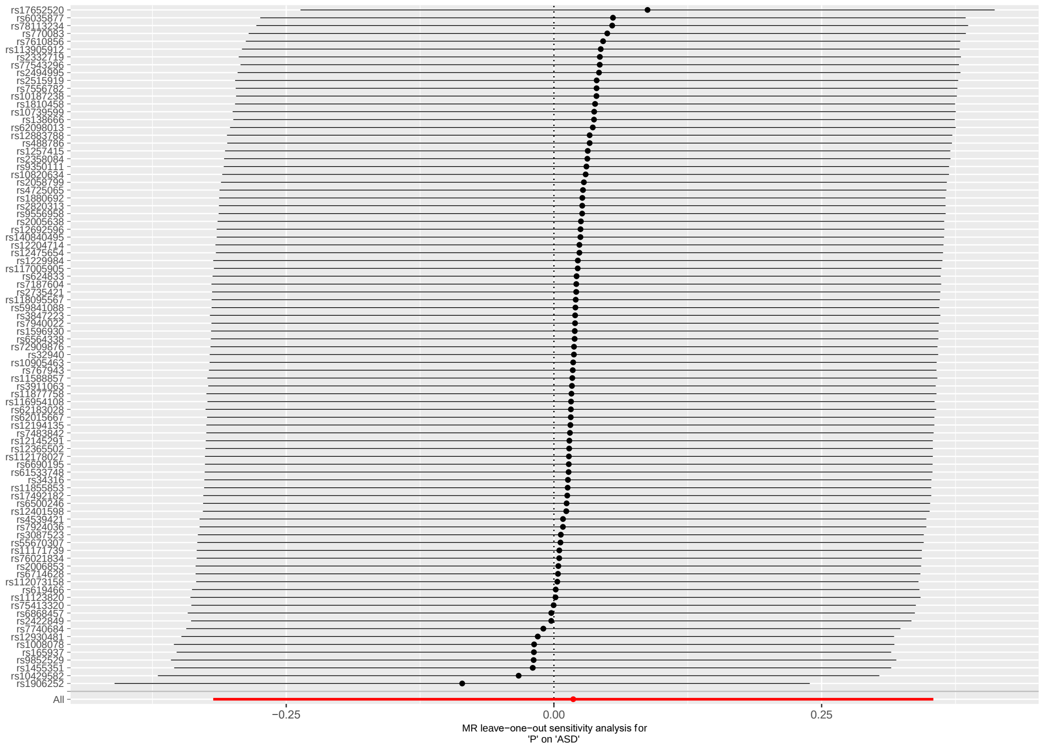

Supplementary Figure 17: leave-one-out analysis of poverty against ASD.
Abbreviations: MR: Mendelian randomization; P: poverty; ASD: autism spectrum disorder.
Legend: n=79 SNPs. Forest plot of individual and combined SNP MR estimated effect size. The effect estimates represent the log-odds for ASD per one standard deviation (1 SD) increase in the mean poverty factor, and the error bars represent 95% CIs.

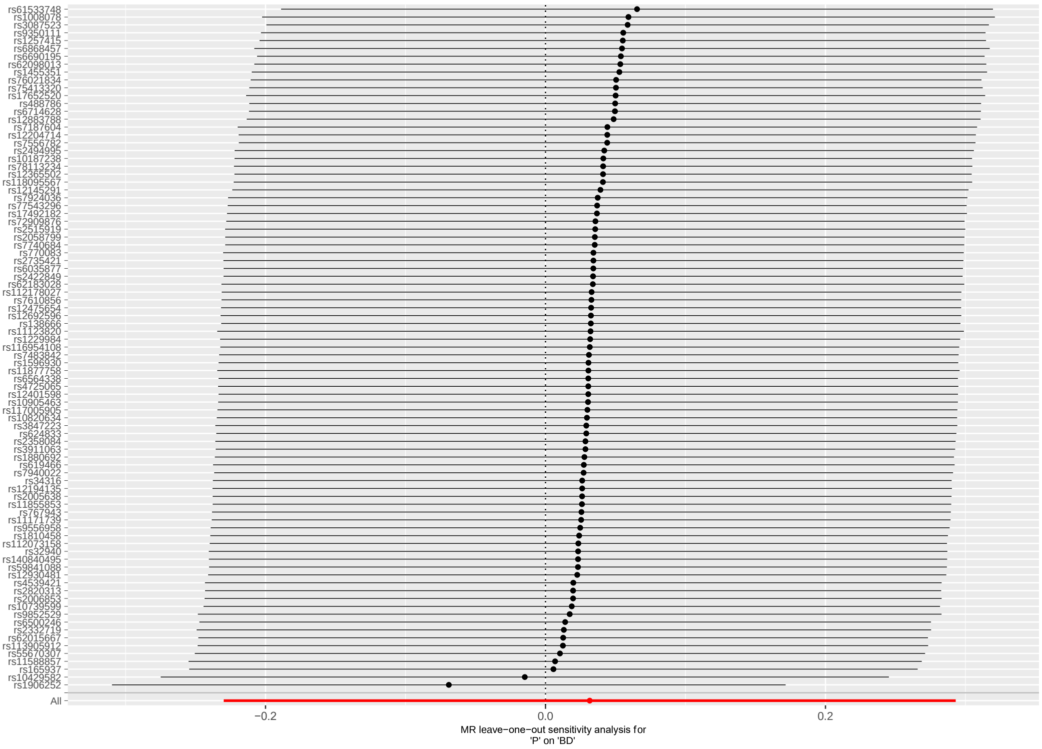

Supplementary Figure 18: leave-one-out analysis of poverty against BD.
Abbreviations: MR: Mendelian randomization; P: poverty; BD: bipolar disorder.
Legend: n=79 SNPs. Forest plot of individual and combined SNP MR estimated effect size. The effect estimates represent the log-odds for BD per one standard deviation (1 SD) increase in the mean poverty factor, and the error bars represent 95% CIs.

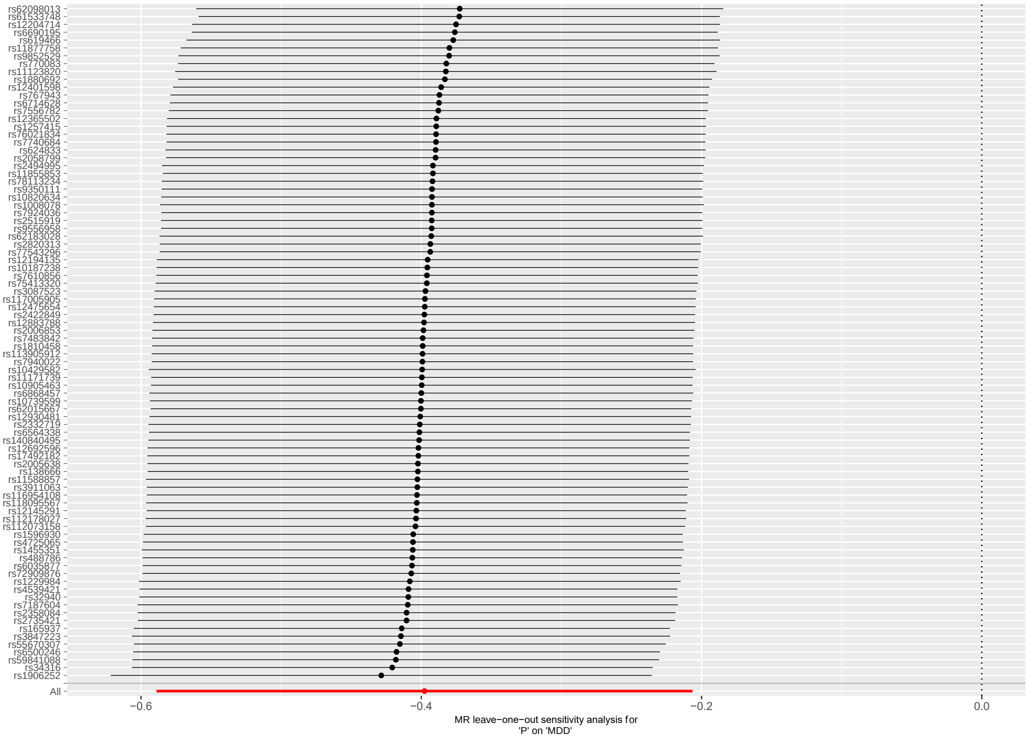

Supplementary Figure 19: leave-one-out analysis of poverty against MDD.
Abbreviations: MR: Mendelian randomization; P: poverty; MDD: major depressive disorder.

Legend: n=78 SNPs. Forest plot of individual and combined SNP MR estimated effect size. The effect estimates represent the log-odds for MDD per one standard deviation (1 SD) increase in the mean poverty factor, and the error bars represent 95% CIs.

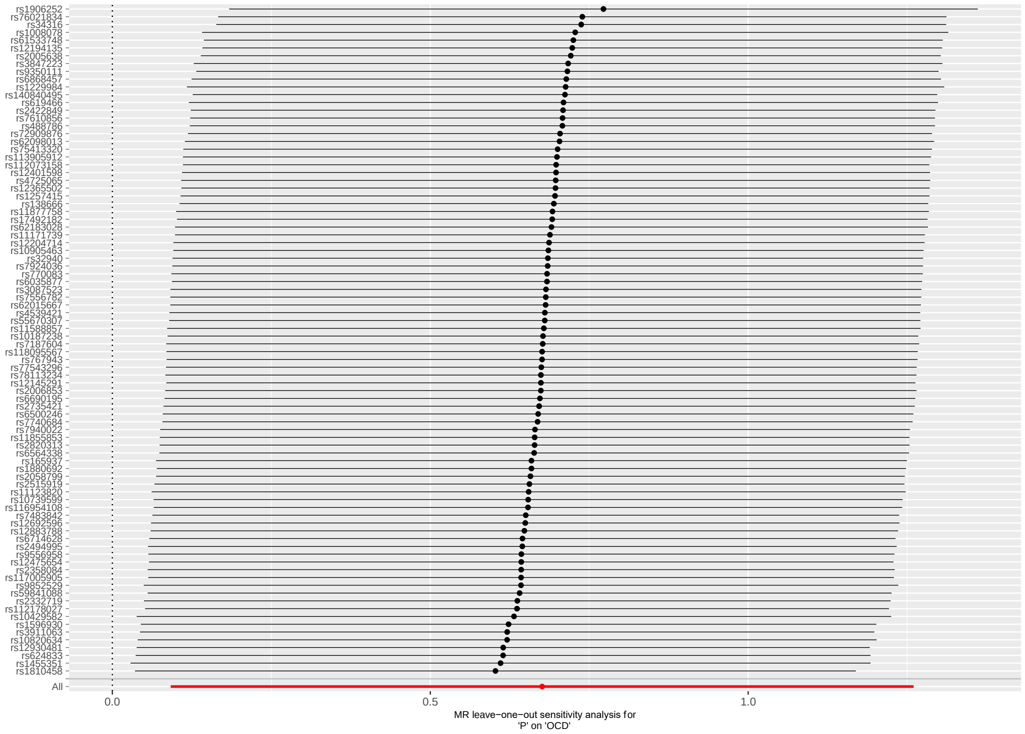

Supplementary Figure 20: leave-one-out analysis of poverty against OCD.
Abbreviations: MR: Mendelian randomization; P: poverty; OCD: obsessive-compulsive disorder.
Legend: n=78 SNPs. Forest plot of individual and combined SNP MR estimated effect size. The effect estimates represent the log-odds for OCD per one standard deviation (1 SD) increase in the mean poverty factor, and the error bars represent 95% CIs.

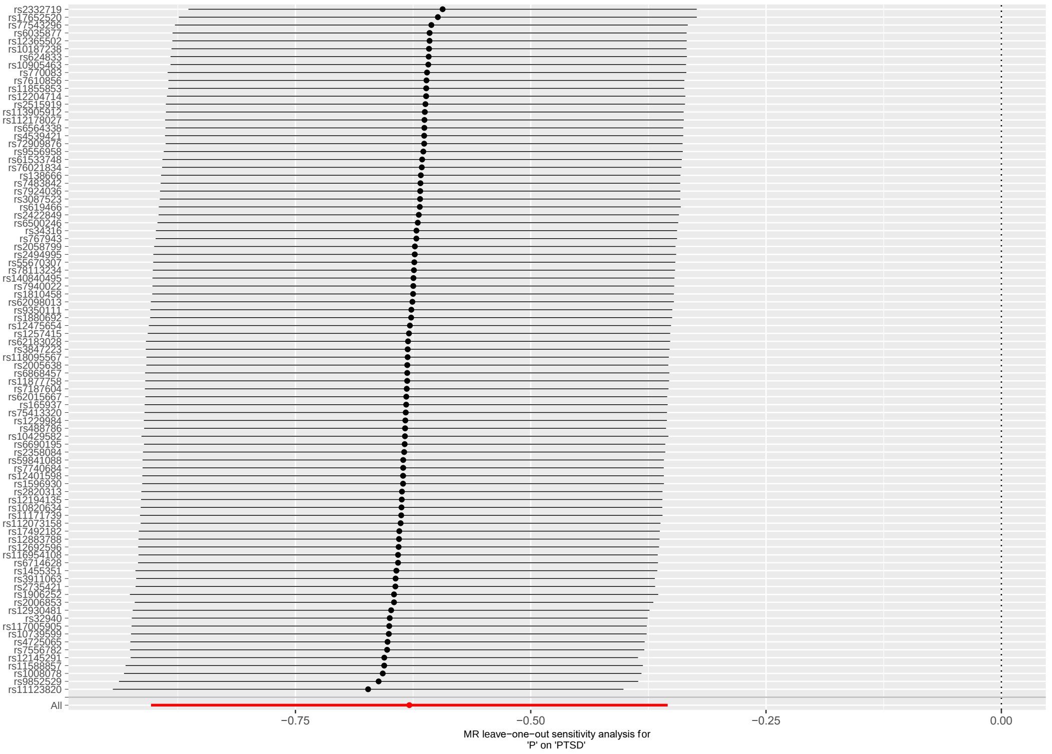

Supplementary Figure 21: leave-one-out analysis of poverty against PTSD.
Abbreviations: MR: Mendelian randomization; P: poverty; PTSD: post-traumatic stress disorder.
Legend: n=79 SNPs. Forest plot of individual and combined SNP MR estimated effect size. The effect estimates represent the log-odds for PTSD per one standard deviation (1 SD) increase in the mean poverty factor, and the error bars represent 95% CIs.

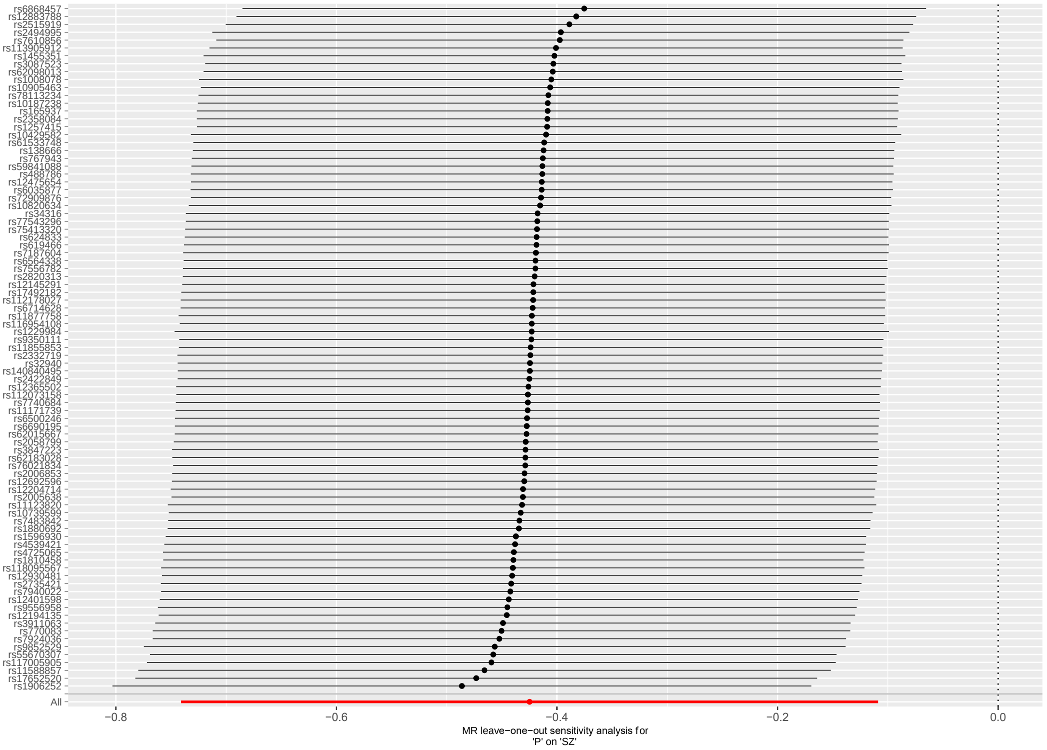

Supplementary Figure 22: leave-one-out analysis of poverty against SZ
Abbreviations: MR: Mendelian randomization; P: poverty; SZ: schizophrenia.

Legend: n=79 SNPs. Forest plot of individual and combined SNP MR estimated effect size. The effect estimates represent the log-odds for SZ per one standard deviation (1 SD) increase in the mean poverty factor, and the error bars represent 95% CIs.

Plots - Backward analyses

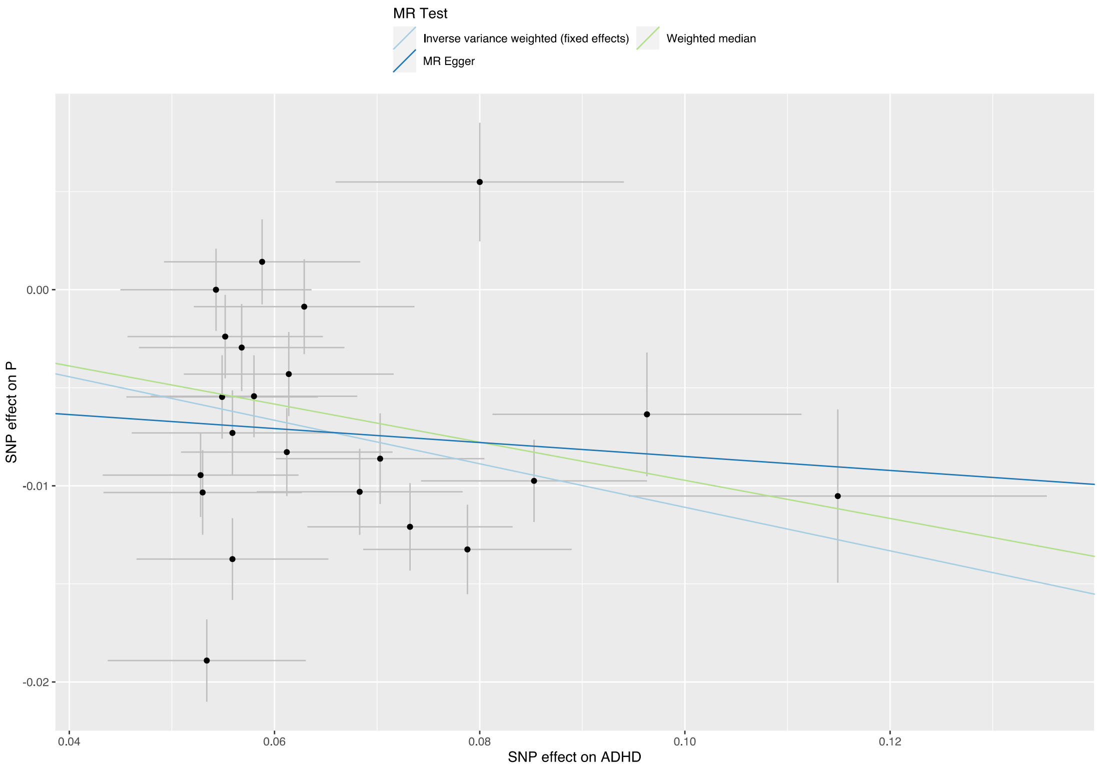

Supplementary Figure 23: scatterplot of ADHD against poverty
Abbreviations: MR: Mendelian randomization; SNP: single nucleotide polymorphism; P: poverty; ADHD: attention deficit hyperactivity disorder.
Legend: n=22 SNPs. Each dot represents a SNP, with the slope of each line corresponding to the estimated MR effect per method.

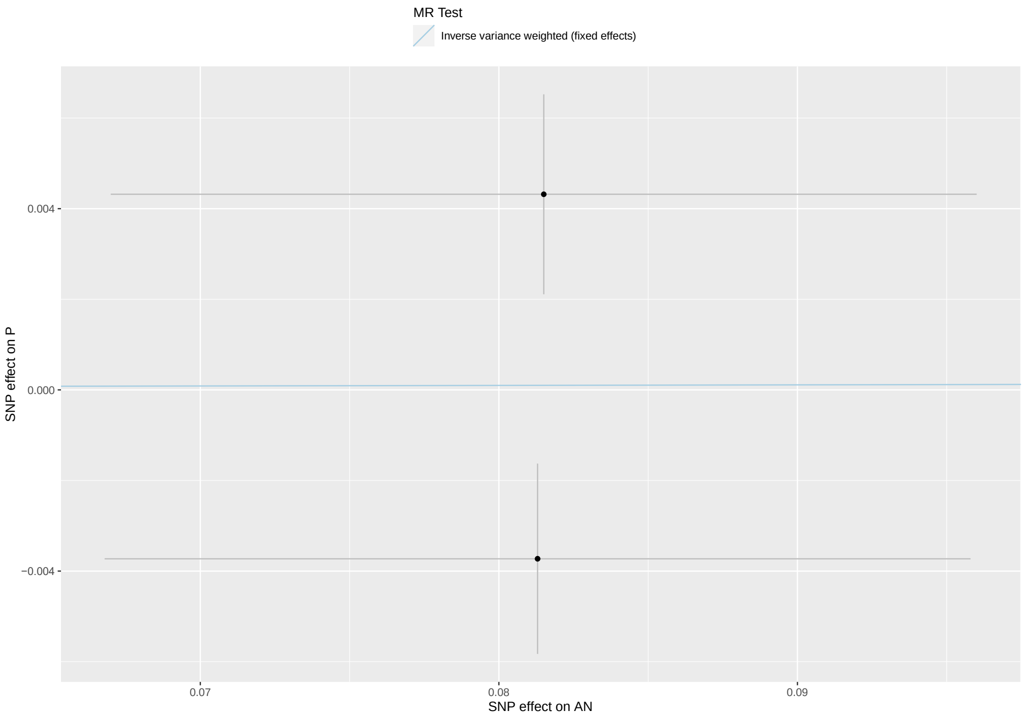

Supplementary Figure 24: scatterplot of AN against poverty
Abbreviations: MR: Mendelian randomization; SNP: single nucleotide polymorphism; P: poverty; AN: anorexia nervosa.
Legend: n=2 SNPs. Each dot represents a SNP, with the slope of each line corresponding to the estimated MR effect per method.

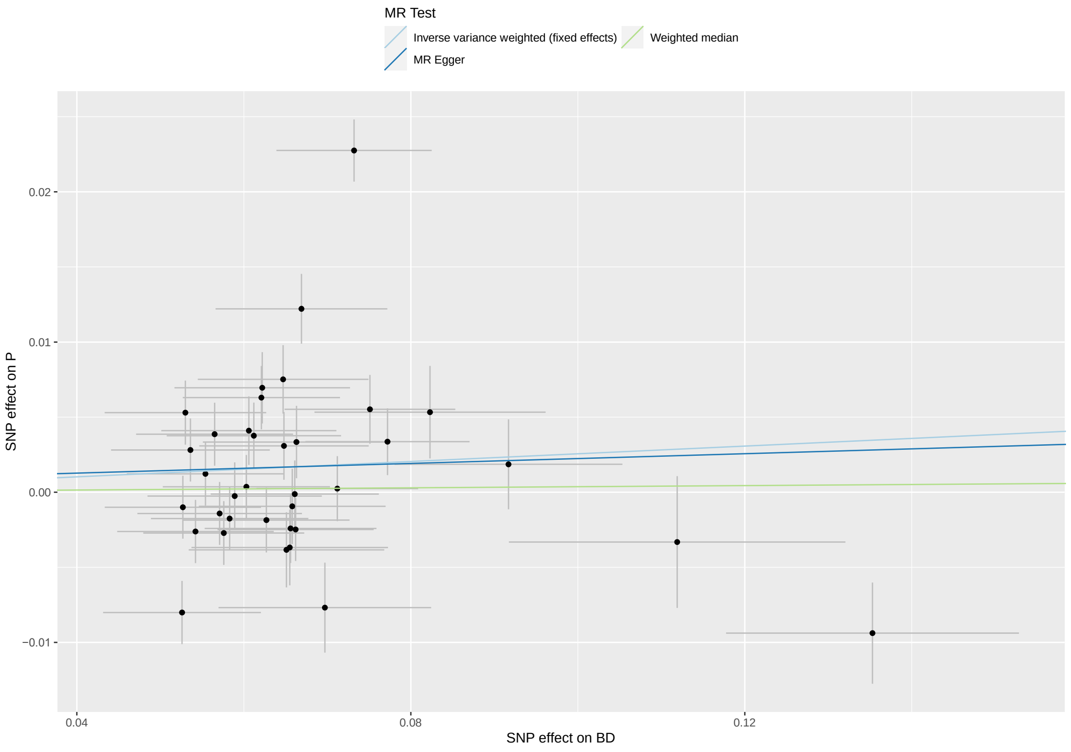

Supplementary Figure 25: scatterplot of BD against poverty
Abbreviations: MR: Mendelian randomization; SNP: single nucleotide polymorphism; P: poverty; BD: bipolar disorder.

Legend: n=36 SNPs. Each dot represents a SNP, with the slope of each line corresponding to the estimated MR effect per method.

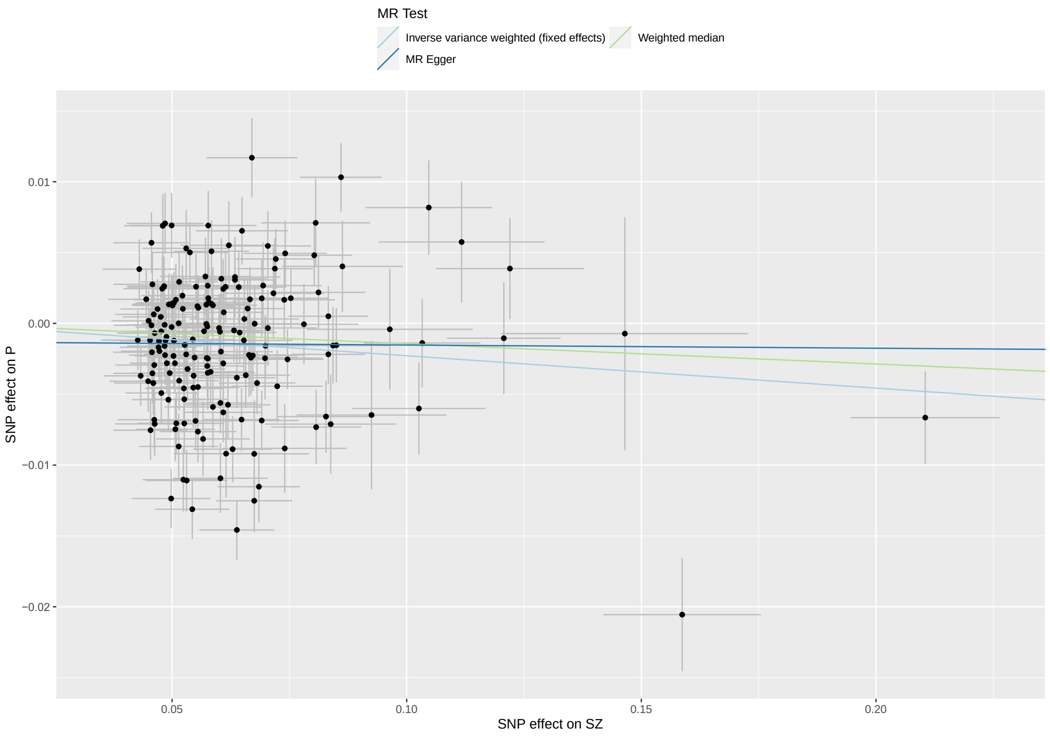

Supplementary Figure 26: scatterplot of SZ against poverty
Abbreviations: MR: Mendelian randomization; SNP: single nucleotide polymorphism; P: poverty; SZ: schizophrenia.
Legend: n=176 SNPs. Each dot represents a SNP, with the slope of each line corresponding to the estimated MR effect per method.

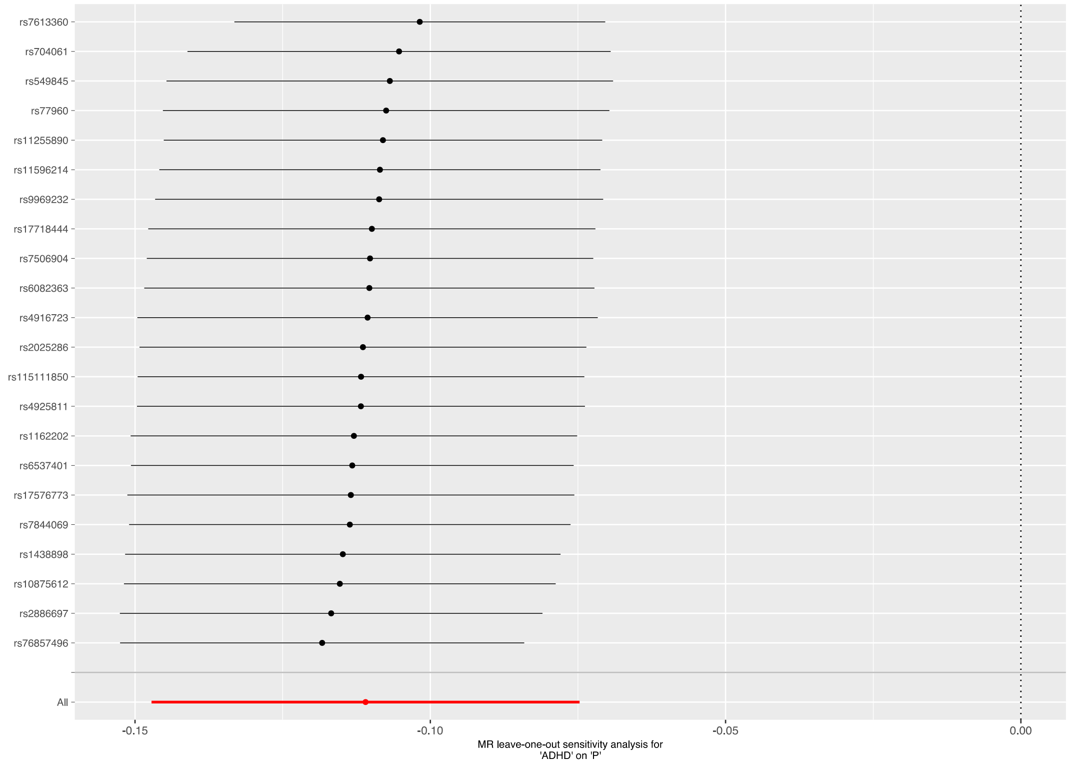

Supplementary Figure 27: leave-one-out analysis of ADHD against poverty
Abbreviations: MR: Mendelian randomization; P: poverty; ADHD: attention deficit hyperactivity disorder.
Legend: n=22 SNPs. Forest plot of individual and combined SNP MR estimated effect size. The effect estimates represent the linear effect for the mean poverty factor per ADHD status, and the error bars represent 95% CIs.

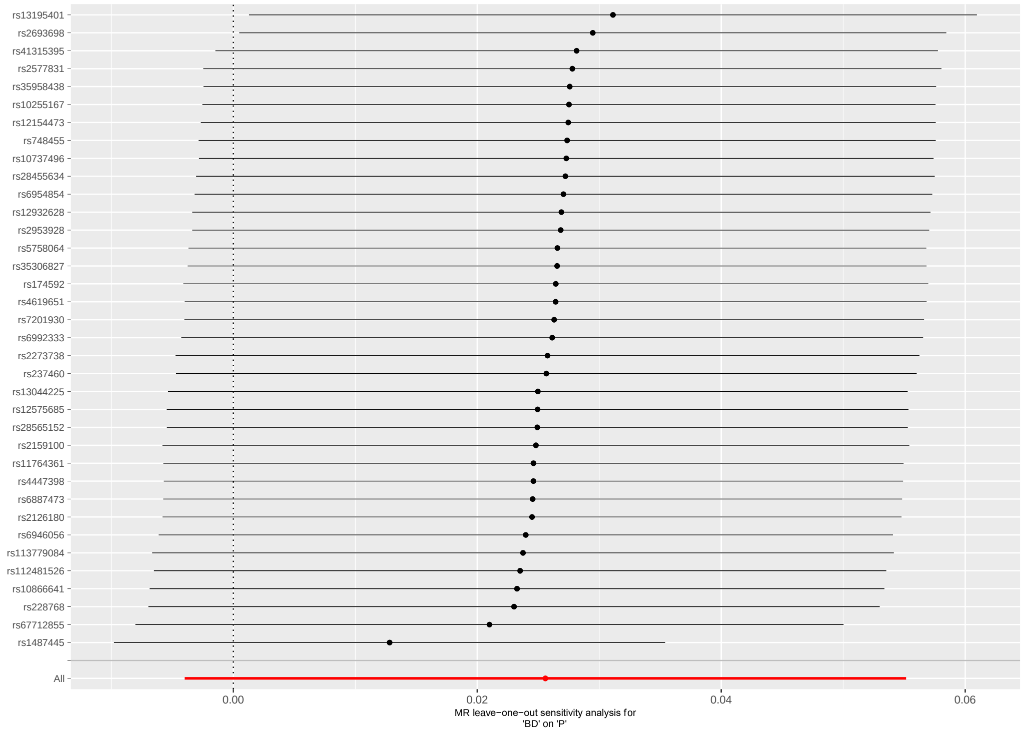

Supplementary Figure 28: leave-one-out analysis of BD against poverty
Abbreviations: MR: Mendelian randomization; P: poverty; BD: bipolar disorder.

Legend: n=36 SNPs. Forest plot of individual and combined SNP MR estimated effect size. The effect estimates represent the linear effect for the mean poverty factor per BD status, and the error bars represent 95% CIs.

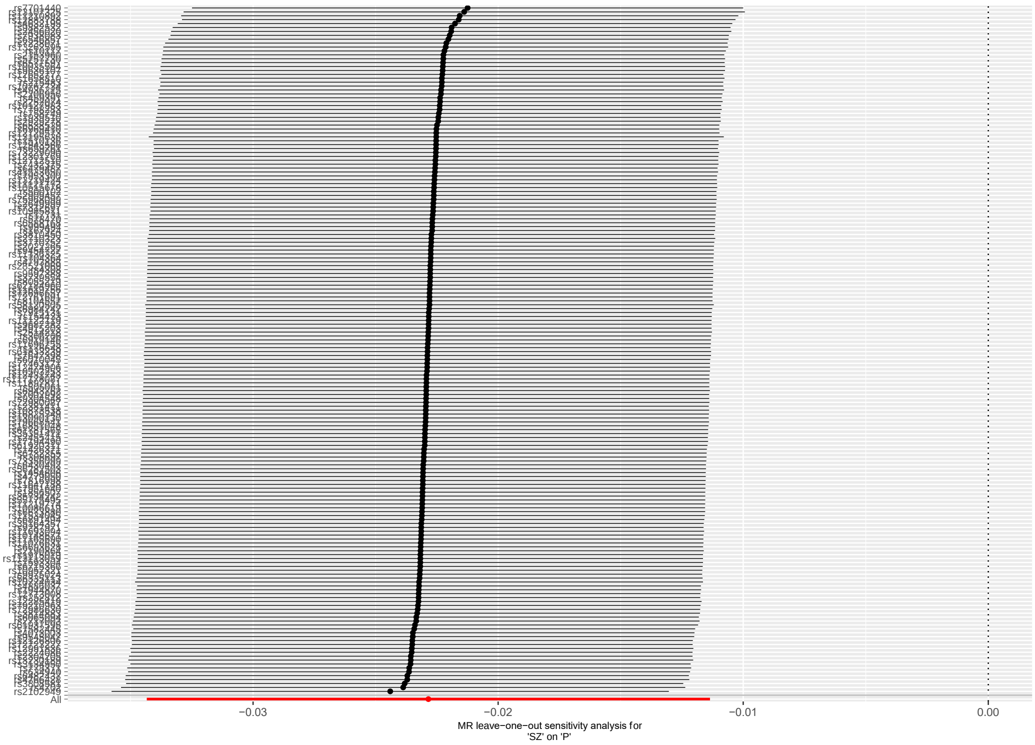

Supplementary Figure 29: leave-one-out analysis of SZ against poverty
Abbreviations: MR: Mendelian randomization; P: poverty; SZ: schizophrenia.
Legend: n=176 SNPs. Forest plot of individual and combined SNP MR estimated effect size. The effect estimates represent the linear effect for the mean poverty factor per ADHD status, and the error bars represent 95% CIs.

Supplementary Table 7: CAUSE results of the relations between poverty and mental illness

**Model 1**	**Model 2**	**Δ ELPD**	**SE Δ ELPD**	**z-score**	**p-value†**
Fw: P on ADHD					
Null	Sharing	-67.54	9.89	-6.83	**8.49×10^{-12}**
Null	Causal	-74.39	10.94	-6.80	**1.46×10^{-11}**
Sharing	Causal	-6.85	1.34	-5.12	**3.06×10^{-7}**
Bw: ADHD on P					
Null	Sharing	-4.26	1.78	-2.40	**0.016**
Null	Causal	-9.60	3.77	-2.55	**0.011**
Sharing	Causal	-5.34	2.01	-2.65	**0.008**
Fw: P on AN					
Null	Sharing	-9.37	3.49	-2.69	**0.007**
Null	Causal	-13.61	5.00	-2.72	**0.006**
Sharing	Causal	-4.24	1.67	-2.54	**0.011**
Bw: AN on P					
Null	Sharing	0.39	0.15	2.62	**0.009**
Null	Causal	0.60	0.98	0.61	0.540
Sharing	Causal	0.21	0.84	0.25	0.803
Fw: P on ANX					

Null<br>Null<br>Sharing	Sharing<br>Causal<br>Causal	-0.41<br>-0.64<br>-0.24	1.11<br>2.02<br>1.03	-0.36<br>-0.32<br>-0.23	0.715<br>0.750<br>0.818
Bw: ANX on P					
Null<br>Null<br>Sharing	Sharing<br>Causal<br>Causal	0.17<br>0.81<br>0.65	0.04<br>0.22<br>0.19	4.09<br>3.70<br>3.41	**4.31×10^{-5}**<br>**2.16×10^{-4}**<br>**0.001**
Fw: P on ASD					
Null<br>Null<br>Sharing	Sharing<br>Causal<br>Causal	0.52<br>1.38<br>0.86	0.07<br>0.11<br>0.03	7.04<br>12.99<br>25.23	**1.92×10^{-12}**<br>**1.39×10^{-38}**<br>**1.88×10^{-140}**
Bw: ASD on P					
Null<br>Null<br>Sharing	Sharing<br>Causal<br>Causal	0.48<br>1.26<br>0.78	0.04<br>0.06<br>0.04	10.65<br>21.68<br>21.17	**1.74×10^{-26}**<br>**3.17×10^{-104}**<br>**1.81×10^{-99}**
Fw: P on BD					
Null<br>Null<br>Sharing	Sharing<br>Causal<br>Causal	0.47<br>1.27<br>0.81	0.09<br>0.50<br>0.43	5.20<br>2.53<br>1.89	**1.99×10^{-7}**<br>**0.011**<br>0.059
Bw: BD on P					
Null<br>Null<br>Sharing	Sharing<br>Causal<br>Causal	0.44<br>1.28<br>0.84	0.04<br>0.05<br>0.02	12.51<br>26.16<br>41.95	**6.58×10^{-36}**<br>**7.58×10^{-151}**<br>**$<1^{-1000}$**
Fw: P on MDD					
Null<br>Null<br>Sharing	Sharing<br>Causal<br>Causal	-9.90<br>-13.68<br>-3.78	3.72<br>5.09<br>1.51	-2.66<br>-2.69<br>-2.50	**0.008**<br>**0.007**<br>**0.013**
Bw: MDD on P					
Null<br>Null<br>Sharing	Sharing<br>Causal<br>Causal	0.01<br>-1.16<br>-1.18	0.36<br>1.81<br>1.45	0.04<br>-0.64<br>-0.81	0.968<br>0.521<br>0.418
Fw: P on OCD					
Null<br>Null<br>Sharing	Sharing<br>Causal<br>Causal	-0.36<br>-1.11<br>-0.75	0.95<br>2.19<br>1.29	-0.38<br>-0.51<br>-0.59	0.707<br>0.612<br>0.558
Bw: OCD on P					
Null<br>Null<br>Sharing	Sharing<br>Causal<br>Causal	0.29<br>0.97<br>0.67	0.05<br>0.36<br>0.32	5.83<br>2.70<br>2.08	**5.54×10^{-9}**<br>**0.007**<br>**0.037**
Fw: P on PTSD					
Null<br>Null<br>Sharing	Sharing<br>Causal<br>Causal	-12.72<br>-16.75<br>-4.03	4.22<br>5.58<br>1.51	-3.01<br>-3.00<br>-2.68	**0.003**<br>**0.003**<br>**0.007**
Bw: PTSD on P					
Null<br>Null<br>Sharing	Sharing<br>Causal<br>Causal	0.19<br>0.65<br>0.46	0.06<br>0.58<br>0.52	3.39<br>1.12<br>0.87	**0.001**<br>0.264<br>0.384
Fw: P on SZ					
Null	Sharing	-1.08	0.96	-1.12	0.261

Null	Causal	-4.24	2.87	-1.48	0.140
Sharing	Causal	-3.16	1.92	-1.65	0.100
Bw: SZ on P					
Null	Sharing	-1.78	1.30	-1.36	0.173
Null	Causal	-5.03	3.24	-1.55	0.121
Sharing	Causal	-3.25	1.95	-1.67	0.095

Abbreviations: MR: mendelian randomization; ELPD: expected log pointwise posterior density; 95%CI: 95% confidence interval; SE: standard error; Fwd: forward MR; Bwd: backward MR; P: latent factor poverty; ADHD: attention deficit hyperactivity disorder; AN: anorexia nervosa; ANX: anxiety disorder; ASD: autism spectrum disorder; BD: bipolar disorder; MDD: major depressive disorder; OCD: obsessive-compulsive disorder; PTSD: post-traumatic stress disorder; SZ: schizophrenia.
Legend: † Based on t-test. The p-values are two-sided and not adjusted for multiple testing, therefore p-value<0.05 was considered significant (and reported in bolded text).

Supplementary Table 8: Results of univariable bidirectional Mendelian Randomization of poverty against mental illnesses, after Steiger filtering

MR	N SNP	IVW, B (95% CI)	IVW p-value	WM, B (95% CI)	WM p-value	MR-Egger, B (95% CI)	MR-Egger p-value	Egger intercept p-value	Mean F
P on ADHD	72	0.283 (0.239; 0.327)	**3.62×10^{-36}**	0.181 (0.105; 0.256)	**2.92×10^{-6}**	-0.210 (-0.497; 0.078)	0.157	**1.91×10^{-5}**	40.8
P on AN	71	-0.147 (-0.212; -0.082)	**9.34×10^{-6}**	-0.170 (-0.267; -0.073)	**6.27×10^{-4}**	-0.420 (-0.799; -0.041)	**0.034**	0.147	40.6
P on ANX	68	0.158 (0.022; 0.294)	**0.023**	0.135 (-0.070; 0.339)	0.197	0.226 (-0.448; 0.900)	0.514	0.997	42.1
P on ASD	67	-0.025 (-0.094; 0.044)	0.473	-0.020 (-0.124; 0.084)	0.703	-0.446 (-0.774; -0.117)	**0.010**	**0.010**	39.6
P on BD	58	-0.014 (-0.064; 0.036)	0.577	-0.036 (-0.109; 0.038)	0.344	0.165 (-0.081; 0.410)	0.194	0.074	39.1
P on MDD	76	0.101 (0.060; 0.143)	**1.49×10^{-6}**	0.082 (0.018; 0.146)	0.012	0.043 (-0.204; 0.289)	0.735	0.549	40.1
P on OCD	49	-0.013 (-0.199; 0.173)	0.893	-0.010 (-0.263; 0.244)	0.941	-0.211 (-0.509; 0.931)	0.569	0.581	43.2
P on PTSD	79	0.140 (0.073; 0.207)	**3.79×10^{-5}**	0.114 (0.012; 0.215)	**0.028**	-0.108 (-0.461; 0.245)	0.551	**0.020**	40.3
P on SZ	76	0.071 (0.035; 0.108)	**1.18×10^{-4}**	0.125 (0.055; 0.194)	**4.24×10^{-4}**	-0.057 (-0.407; 0.294)	0.752	**0.003**	40.2

Abbreviations: P: poverty; ADHD: attention deficit hyperactivity disorder; AN: anorexia nervosa; ANX: anxiety disorder; ASD: autism spectrum disorders; BD: bipolar disorder; MDD: major depressive disorder; OCD: obsessive-compulsive disorder; PTSD: post-traumatic stress disorder; SZ: schizophrenia; MR: mendelian randomization; SNP: single nucleotide polymorphism; IVW: inverse variance weighted (fixed effect); B: effect estimates are log-odds; 95% CI: 95% confidence interval; WM: weighted median; NR: not reported because not enough SNP to perform MR.

Legend: Poverty is a latent variable built using household income as unit identification, therefore an increase in the indicator's load stands for increased income, therefore the regression coefficients have been reversed to facilitate interpretation of the effect of poverty. All statistical tests were two-sided. The p-values were not adjusted for multiple testing, therefore p-value<0.05 was considered significant (and reported in bolded text).

Univariable Mendelian randomization of household income and mental illnesses

Supplementary Table 9: Odds Ratio of univariable forward Mendelian randomization analysis of household income against mental illnesses

MR: method	OR (95% CI)	p-value
HI → ADHD:		
IVW	0.436 (0.363; 0.524)	**5.91×10^{-19}**
WM	0.571 (0.422; 0.774)	**2.91×10^{-4}**
MR-Egger	1.83 (0.504; 6.67)	0.362
HI → AN:		
IVW	1.56 (1.21; 2.02)	**0.001**
WM	1.45 (0.978; 2.14)	0.078
MR-Egger	2.69 (0.492; 14.7)	0.259
HI → ANX:		
IVW	0.418 (0.243; 0.718)	**0.002**
WM	0.608 (0.271; 1.37)	0.229
MR-Egger	0.913 (0.050; 16.7)	0.951
HI → ASD:		
IVW	0.933 (0.729; 1.19)	0.583
WM	0.910 (0.600; 1.38)	0.654
MR-Egger	2.23 (0.428; 11.6)	0.346
HI → BD:		
IVW	1.15 (0.964; 1.38)	0.120
WM	1.07 (0.790; 1.44)	0.685
MR-Egger	3.71 (0.707; 19.5)	0.128
HI → MDD:		
IVW	0.656 (0.555; 0.775)	**7.37×10^{-7}**
WM	0.743 (0.572; 0.966)	0.027
MR-Egger	0.647 (0.274; 1.53)	0.325
HI → OCD:		
IVW	1.52 (0.814; 2.85)	0.189
WM	1.35 (0.553; 3.30)	0.528
MR-Egger	1.08 (0.089; 13.2)	0.951
HI → PTSD:		
IVW	0.652 (0.500; 0.851)	**0.002**
WM	0.736 (0.497; 1.09)	0.135
MR-Egger	0.728 (0.205; 2.59)	0.626
HI → SZ:		
IVW	0.660 (0.569; 0.767)	**5.81×10^{-8}**
WM	0.649 (0.487; 0.865)	**0.005**
MR-Egger	0.864 (0.171; 4.38)	0.861

Abbreviations: MR: Mendelian randomization; OR: Odds Ratio; 95% CI: 95% confidence intervals; HI: household income; ADHD: attention deficit hyperactivity disorder; AN: anorexia nervosa; ANX: anxiety disorder; ASD: autism spectrum disorders; BD: bipolar disorder; MDD: major depressive disorder; OCD: obsessive-compulsive disorder; PTSD: post-traumatic stress disorder; SZ: schizophrenia; IVW: inverse variance weighted (fixed effect); WM: weighted median; NR: not reported because not enough SNP to perform MR. Legend: All statistical tests were two-sided. The p-values were not adjusted for multiple testing, therefore p-value<0.05 was considered significant (and reported in bolded text).

Supplementary Table 10: Results of univariable bidirectional Mendelian Randomization of household income against mental illnesses

MR	N SNP	IVW, B (95% CI)	IVW p-value	IVW Q(df)	Q p-value[†]	WM, B (95% CI)	WM p-value	MR-Egger, B (95% CI)	MR-Egger p-value	Egger intercept p-value	Steiger Test p-value[‡]	MR-PRESSO	Mean F
Fw: HI on ADHD	46	-0.830 (-1.01; -0.647)	**5.91×10^{-19}**	125 (45)	**1.92×10^{-9}**	-0.560 (-0.863; -0.256)	**2.91×10^{-4}**	0.606 (-0.684; 1.90)	0.362	**0.030**	**5.62×10^{-53}**	DT; p=0.215	36.7
Bw: ADHD on HI	23	-0.103 (-0.120; -0.086)	**4.28×10^{-33}**	75.2 (22)	**9.84×10^{-9}**	-0.079 (-0.108; -0.049)	**2.37×10^{-7}**	0.019 (-0.159; 0.197)	0.836	0.185	**2.10×10^{-53}**	DT; p=0.572	39.2
Fw: HI on AN	50	0.448 (0.191; 0.704)	**0.001**	100 (49)	**2.50×10^{-5}**	0.370 (-0.041; 0.781)	0.078	0.990 (-0.710; 2.69)	0.259	0.524	**3.67×10^{-16}**	DT; p=0.149	37.2
Bw: AN on HI	4	-0.012 (-0.044; 0.019)	0.444	9 (3)	**0.027**	-0.005 (-0.043; 0.034)	0.817	0.268 (-0.187; 0.722)	0.367	0.348	NR [b]	GT; p=0.093	31.9
Fw: HI on ANX	48	-0.872 (-1.41; -0.331)	**0.002**	48 (47)	0.434	-0.497 (-1.31; 0.312)	0.229	-0.091 (-3.00; 2.82)	0.951	0.594	**5.61×10^{-9}**	GT; p=0.427	37.3
Bw: ANX on HI	0	NR [c]	NR [c]	NR [c]	NR [c]	NR [c]	NR [c]	NR [c]	NR [c]	NR [c]	NR [c]	NR [c]	NR [c]
Fw: HI on ASD	54	-0.069 (-0.316; 0.178)	0.583	134 (53)	**6.80×10^{-9}**	-0.095 (-0.508; 0.319)	0.654	0.801 (-0.850; 2.45)	0.346	0.292	NR [b]	DT; p=0.909	37.5
Bw: ASD on HI	0	NR [c]	NR [c]	NR [c]	NR [c]	NR [c]	NR [c]	NR [c]	NR [c]	NR [c]	NR [c]	NR [c]	NR [c]
Fw: HI on BD	47	0.143 (-0.037; 0.322)	0.120	207 (46)	**3.64×10^{-22}**	0.063 (-0.242; 0.368)	0.685	1.31 (-0.347; 2.97)	0.128	0.163	NR [b]	DT; p=0.093	37.2
Bw: BD on HI	36	0.016 (0.002; 0.030)	**0.020**	189 (35)	**5.82×10^{-23}**	0.018 (-0.005; 0.042)	0.122	-0.015 (-0.188; 0.158)	0.869	0.726	**1.58×10^{-260}**	DT; p=0.098	39.2
Fw: HI on MDD	50	-0.422 (-0.589; -0.255)	**7.37×10^{-7}**	72 (49)	**0.017**	-0.297 (-0.561; -0.032)	**0.027**	-0.435 (-1.29; 0.423)	0.325	0.975	**4.71×10^{-54}**	DT; p=0.725	36.9
Bw: MDD on HI	0	NR [c]	NR [c]	NR [c]	NR [c]	NR [c]	NR [c]	NR [c]	NR [c]	NR [c]	NR [c]	NR [c]	NR [c]

Fw: HI on OCD	50	0.420 (-0.206; 1.05)	0.189	48 (49)	0.499	0.301 (-0.633; 1.23)	0.528	0.079 (-2.42; 2.58)	0.951	0.784	NR [b]	GT; p=0.514	36.9
Bw: OCD on HI	0	NR [c]	NR [c]	NR [c]	NR [c]	NR [c]	NR [c]	NR [c]	NR [c]	NR [c]	NR [c]	NR [c]	NR [c]
Fw: HI on PTSD	54	-0.427 (-0.693; -0.161)	**0.002**	66 (53)	0.113	-0.306 (-0.708; 0.096)	0.135	-0.317 (-1.59; 0.952)	0.626	0.861	**1.66×10^{-84}**	GT; p=0.112	37.5
Bw: PTSD on HI	0	NR [c]	NR [c]	NR [c]	NR [c]	NR [c]	NR [c]	NR [c]	NR [c]	NR [c]	NR [c]	NR [c]	NR [c]
Fw: HI on SZ	47	-0.415 (-0.565; -0.265)	**5.81×10^{-8}**	338 (46)	**5.41×10^{-45}**	-0.432 (-0.733; -0.130)	**0.005**	-0.146 (-1.77; 1.48)	0.861	0.739	**3.44×10^{-56}**	DT; p=0.431	37.2
Bw: SZ on HI	176	-0.031 (-0.037; -0.024)	**7.19×10^{-19}**	603 (175)	**1.91×10^{-47}**	-0.025 (-0.037; -0.012)	**0.001**	-0.018 (-0.067; 0.031)	0.477	0.597	**$<1^{-1000}$**	DT; p=0.240	45.6

Abbreviations: Fw: forward analysis; Bw: backward analysis; HI: household income; ADHD: attention deficit hyperactivity disorder; AN: anorexia nervosa; ANX: anxiety disorder; ASD: autism spectrum disorders; BD: bipolar disorder; MDD: major depressive disorder; OCD: obsessive-compulsive disorder; PTSD: post-traumatic stress disorder; SZ: schizophrenia; MR: mendelian randomization; SNP: single nucleotide polymorphism; IVW: inverse variance weighted (fixed effect); B: effect estimates are log-odds for binary traits (i.e., for mental illnesses) and unstandardized regression coefficient for continuous traits (i.e., for household income); 95% CI: 95% confidence interval; Q: Cochran's Q measure of heterogeneity; df: degree of freedom; WM: weighted median; DT: distortion test; GT: global test.

Legend:

P-value threshold for SNP selection <5e-8.

[a] The Mendelian randomization pleiotropy residual sum and outlier (MR-PRESSO) test identifies possible bias from horizontal pleiotropy. The test consists of three parts. (1) the MR-PRESSO global test which detects horizontal pleiotropy. (2) the outlier corrected causal estimate which corrects for the detected horizontal pleiotropy and (3) the MR-PRESSO distortion test which estimates if the causal estimate is significantly different (at p<0.05) after adjustment for outliers. We conduct all three stages (with the argument NbDistribution=1000. namely using1000 simulation form the null distribution to compute empirical p-values) and present the outlier adjusted causal estimates (OACE) when both global and distortion tests are significant.

[b] We did not run Steiger Test if none of the MR analysis resulted significant (NR: not reported in the cell).

[c] Not enough SNP to perform MR (NR: not reported in the cell).

† Based on chi2 test.

‡ Based on t-test.

All statistical tests were two-sided. The p-values were not adjusted for multiple testing, therefore p-value<0.05 was considered significant (and reported in bolded text).

Plots - Forward analyses

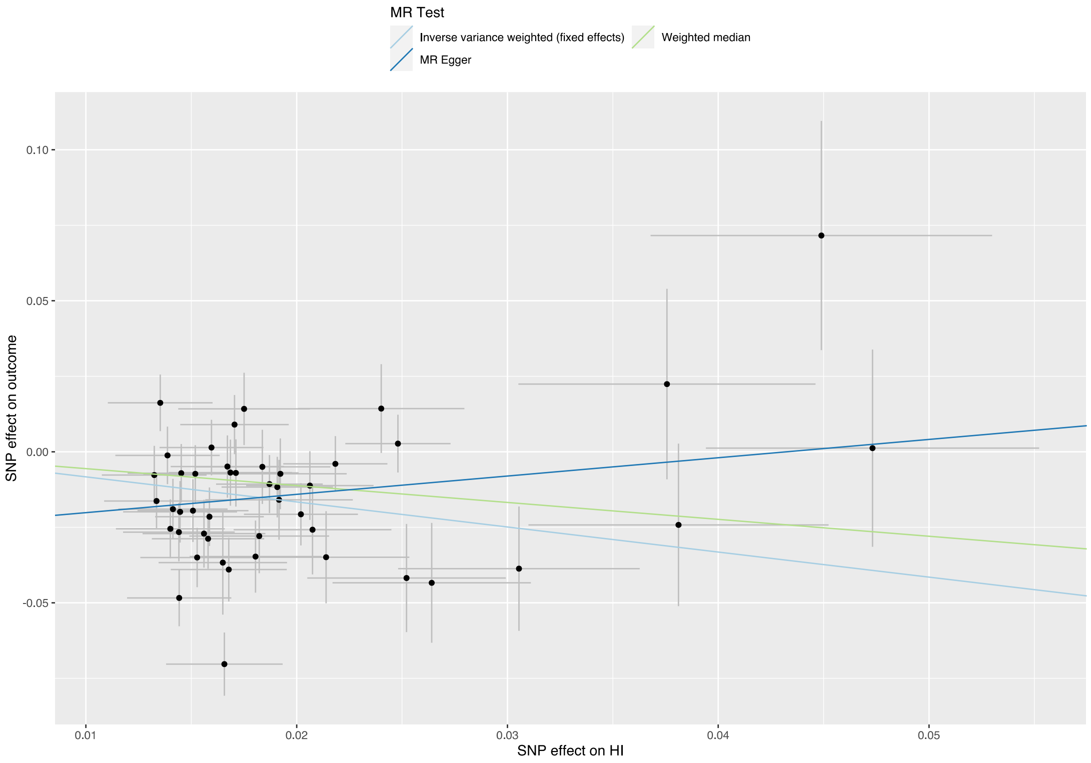

Supplementary Figure 30: scatterplot of household income against ADHD
Abbreviations: MR: Mendelian randomization; SNP: single nucleotide polymorphism; HI: household income; ADHD: attention deficit hyperactivity disorder.
Legend: n=46 SNPs. Each dot represents a SNP, with the slope of each line corresponding to the estimated MR effect per method.

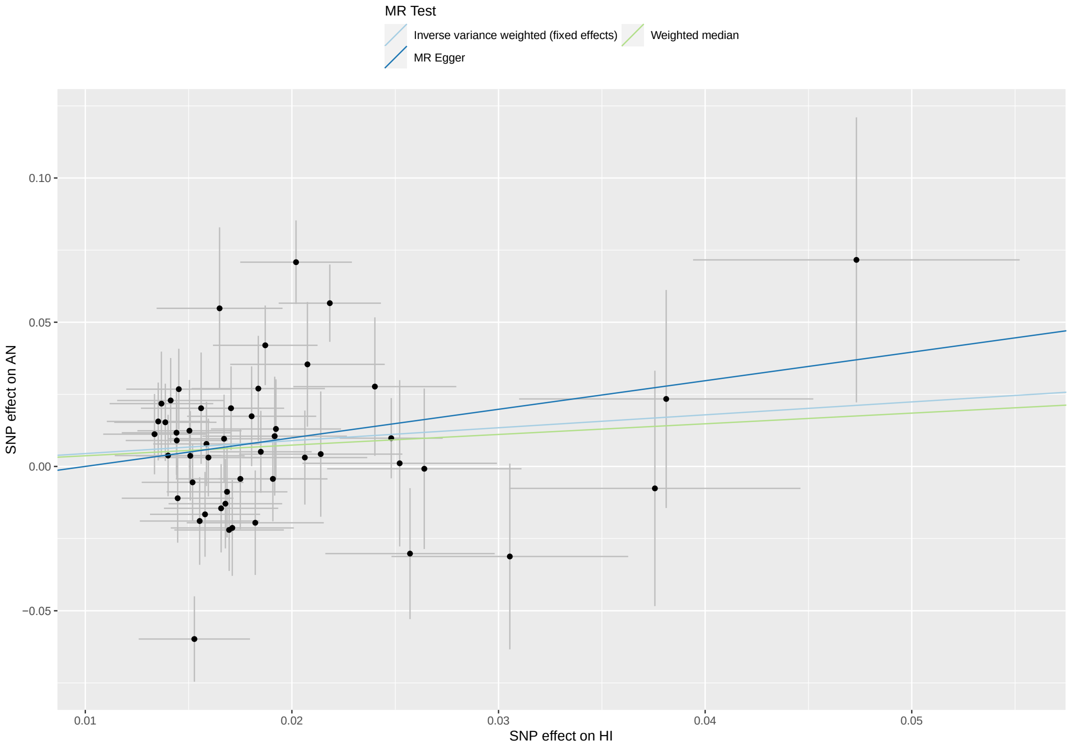

Supplementary Figure 31: scatterplot of household income against AN
Abbreviations: MR: Mendelian randomization; SNP: single nucleotide polymorphism; HI: household income; AN: anorexia nervosa.
Legend: n=50 SNPs. Each dot represents a SNP, with the slope of each line corresponding to the estimated MR effect per method.

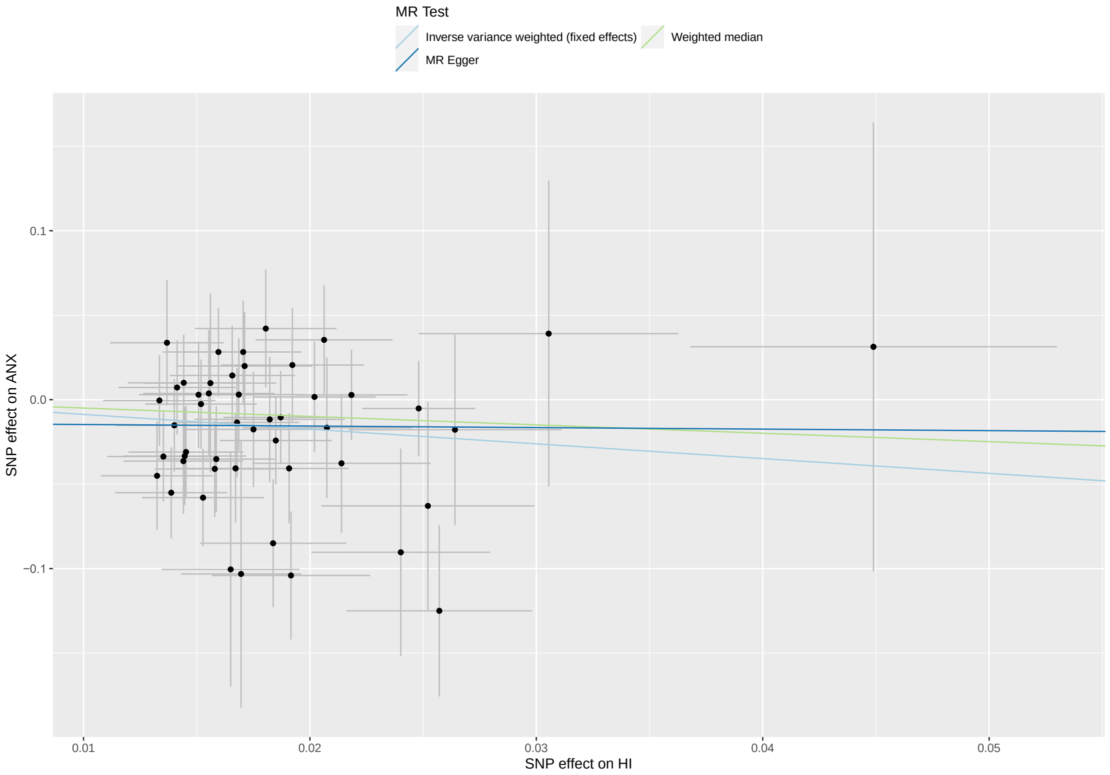

Supplementary Figure 32: scatterplot of household income against ANX
Abbreviations: MR: Mendelian randomization; SNP: single nucleotide polymorphism; HI: household income; ANX: anxiety disorder.
Legend: n=48 SNPs. Each dot represents a SNP, with the slope of each line corresponding to the estimated MR effect per method.

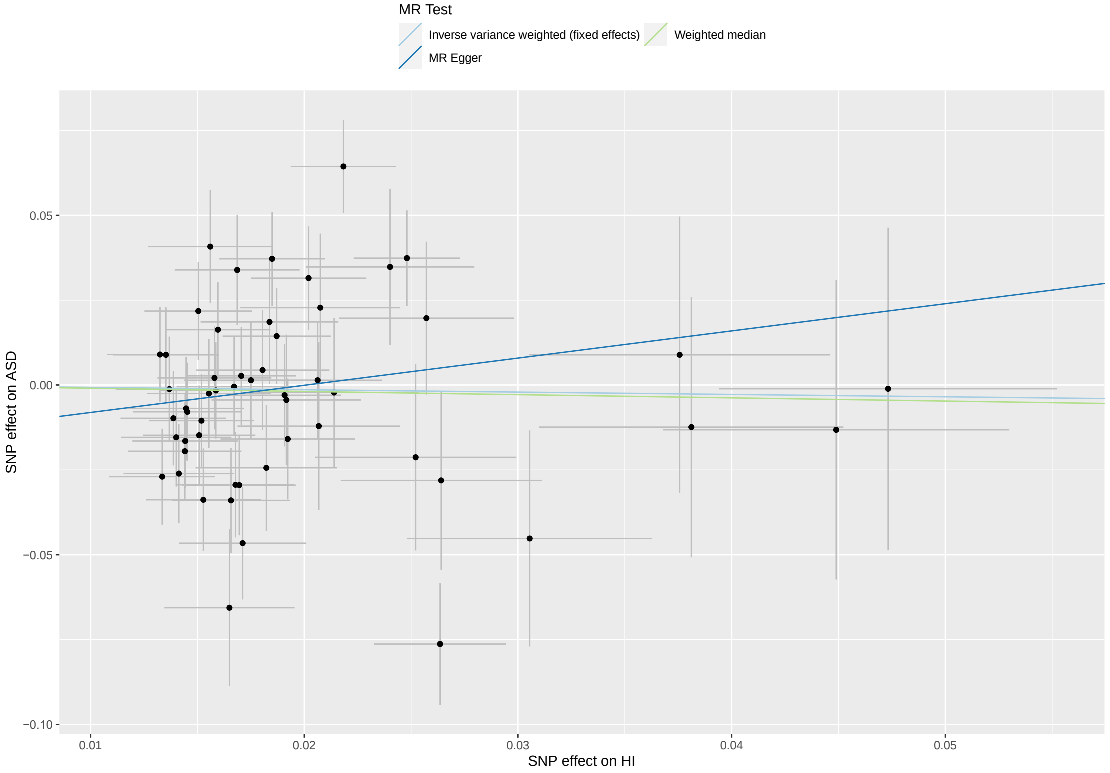

Supplementary Figure 33: scatterplot of household income against ASD
Abbreviations: MR: Mendelian randomization; SNP: single nucleotide polymorphism; HI: household income; ASD: autism spectrum disorder.
Legend: n=54 SNPs. Each dot represents a SNP, with the slope of each line corresponding to the estimated MR effect per method.

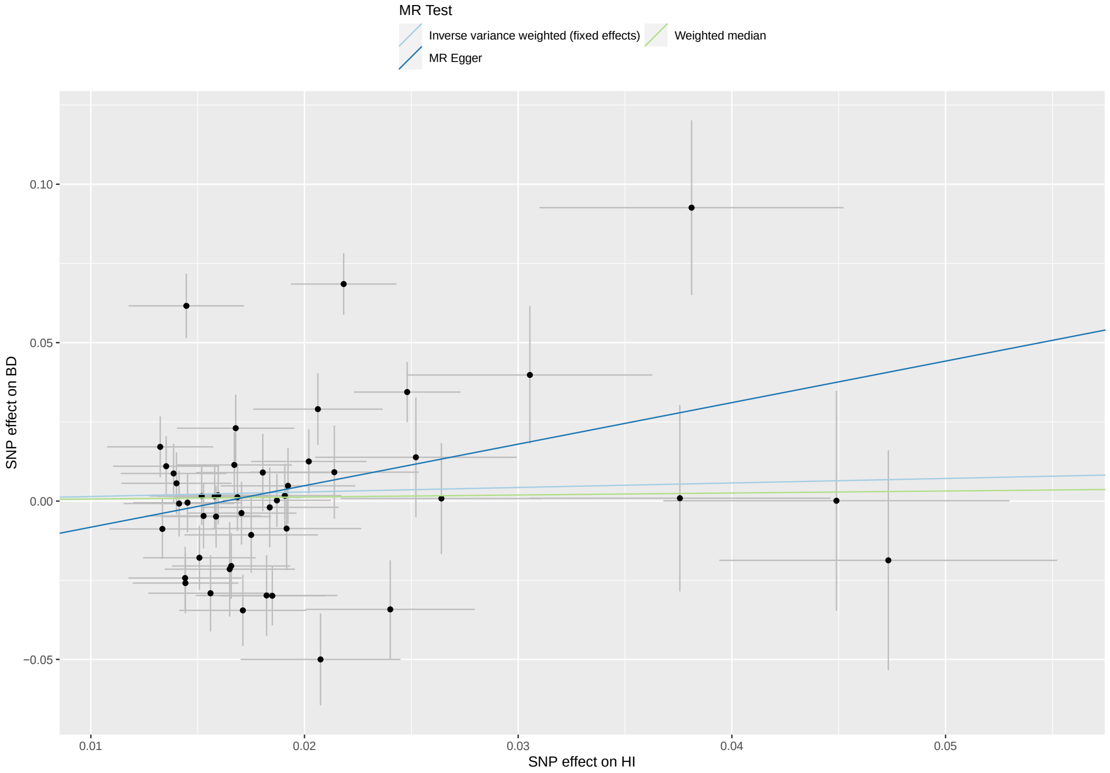

Supplementary Figure 34: scatterplot of household income against BD
Abbreviations: MR: Mendelian randomization; SNP: single nucleotide polymorphism; HI: household income; BD: bipolar disorder.
Legend: n=47 SNPs. Each dot represents a SNP, with the slope of each line corresponding to the estimated MR effect per method.

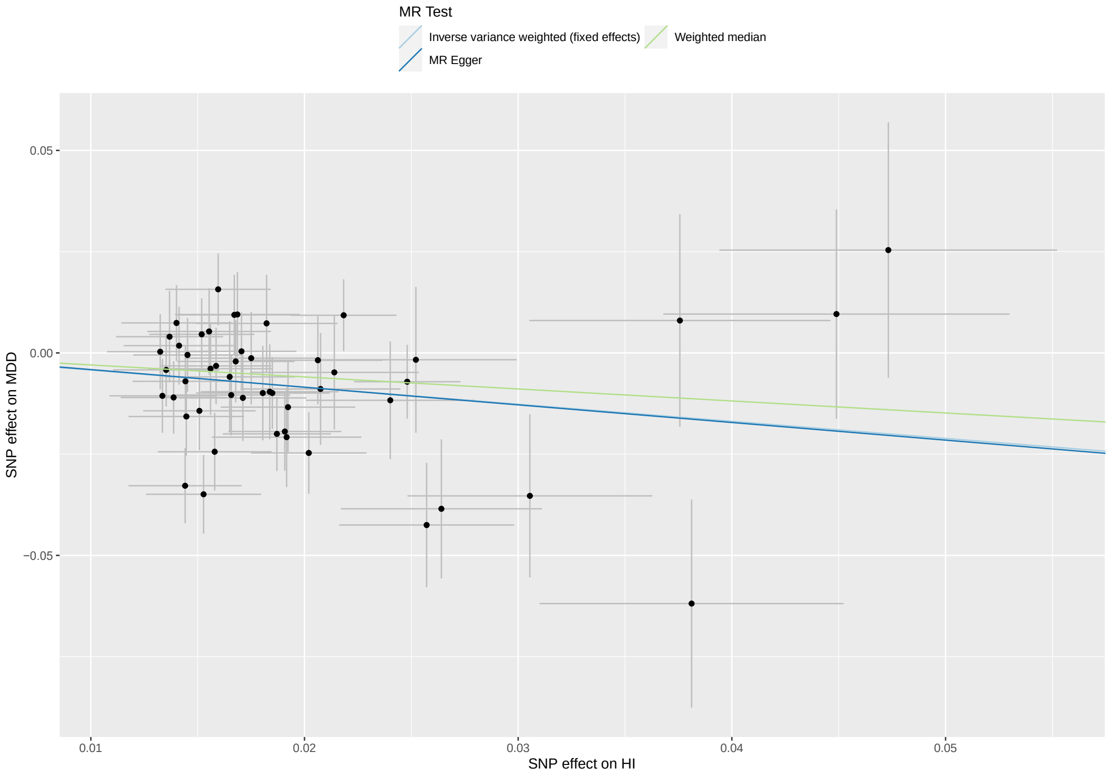

Supplementary Figure 35: scatterplot of household income against MDD
Abbreviations: MR: Mendelian randomization; SNP: single nucleotide polymorphism; HI: household income; MDD: major depressive disorder.
Legend: n=50 SNPs. Each dot represents a SNP, with the slope of each line corresponding to the estimated MR effect per method.

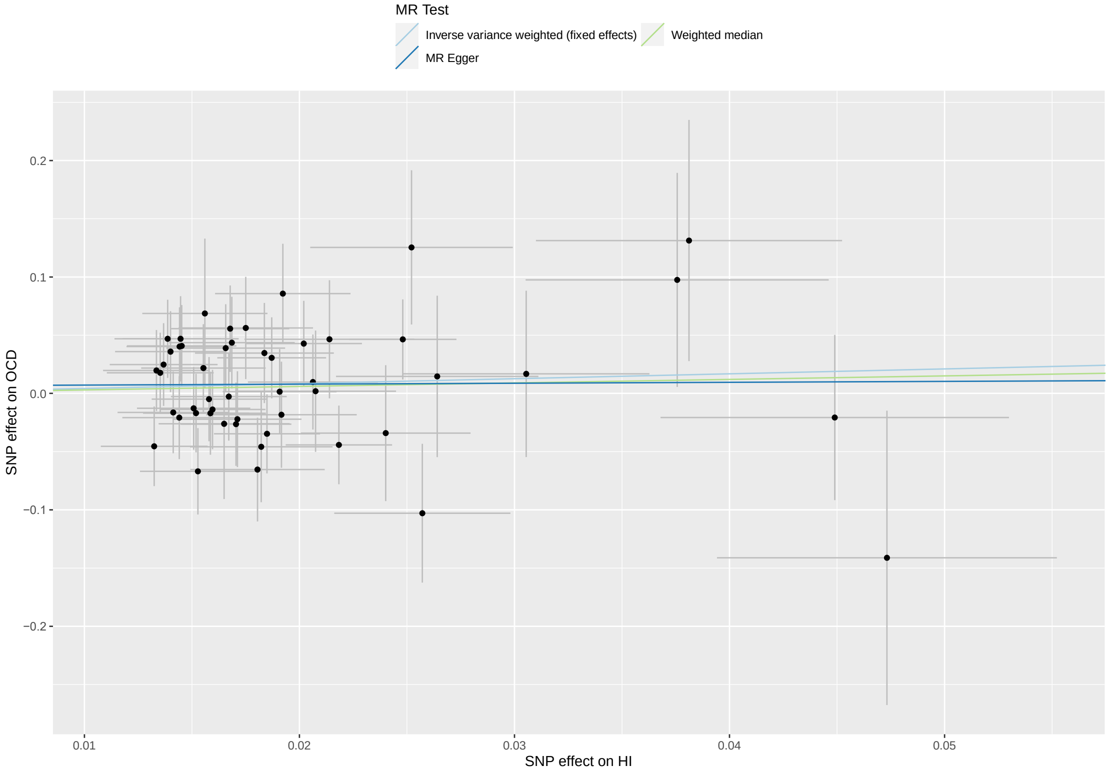

Supplementary Figure 36: scatterplot of household income against OCD
Abbreviations: MR: Mendelian randomization; SNP: single nucleotide polymorphism; HI: household income; OCD: obsessive compulsive disorder.
Legend: n=50 SNPs. Each dot represents a SNP, with the slope of each line corresponding to the estimated MR effect per method.

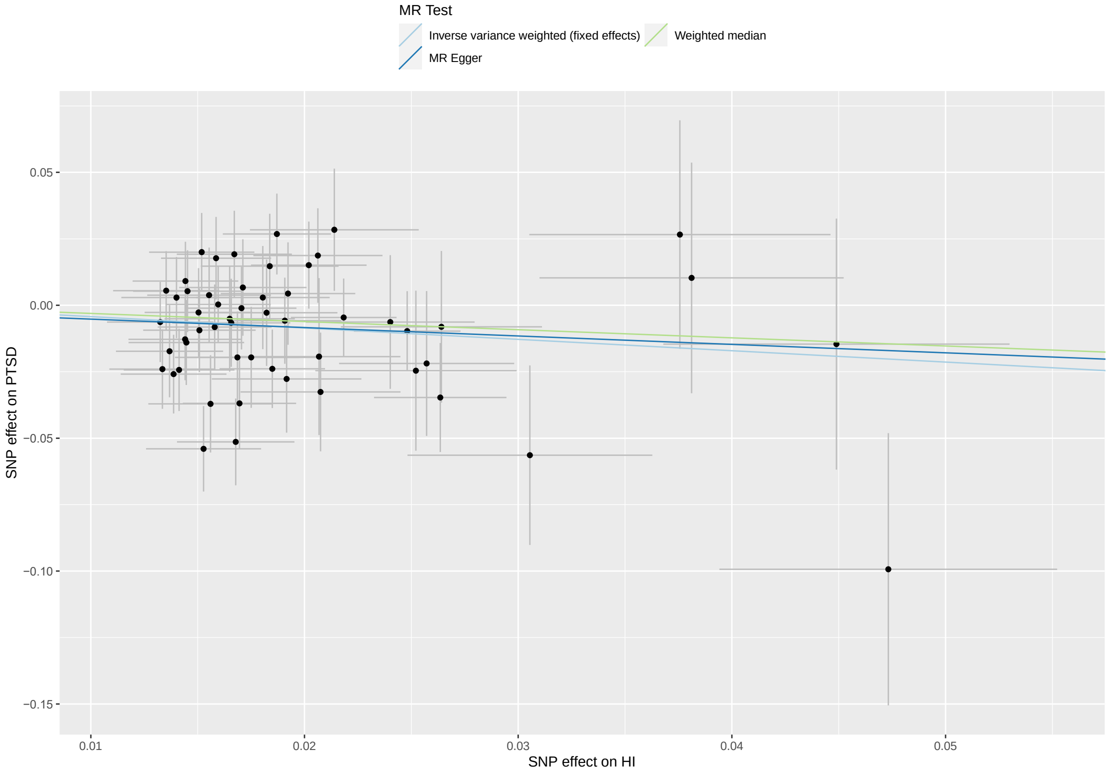

Supplementary Figure 37: scatterplot of household income against PTSD
Abbreviations: MR: Mendelian randomization; SNP: single nucleotide polymorphism; HI: household income; PTSD: post-traumatic stress disorder.
Legend: n=54 SNPs. Each dot represents a SNP, with the slope of each line corresponding to the estimated MR effect per method.

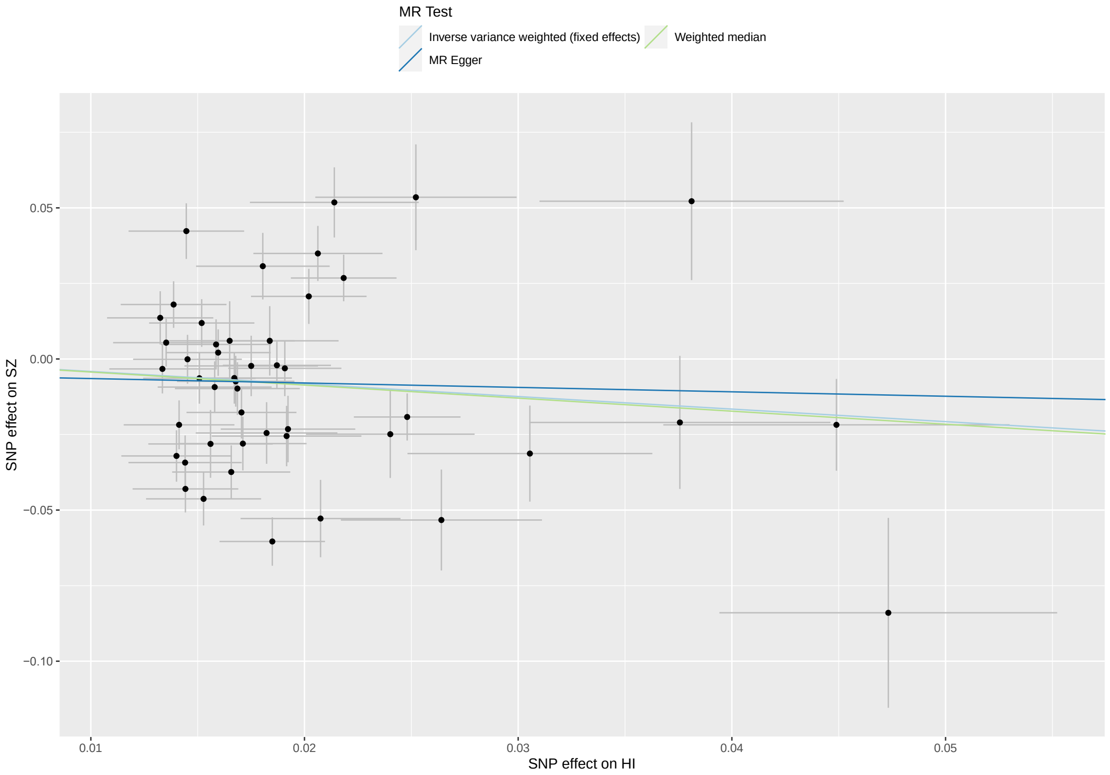

Supplementary Figure 38: scatterplot of household income against SZ
Abbreviations: MR: Mendelian randomization; SNP: single nucleotide polymorphism; HI: household income; SZ: schizophrenia.
Legend: n=47 SNPs. Each dot represents a SNP, with the slope of each line corresponding to the estimated MR effect per method.

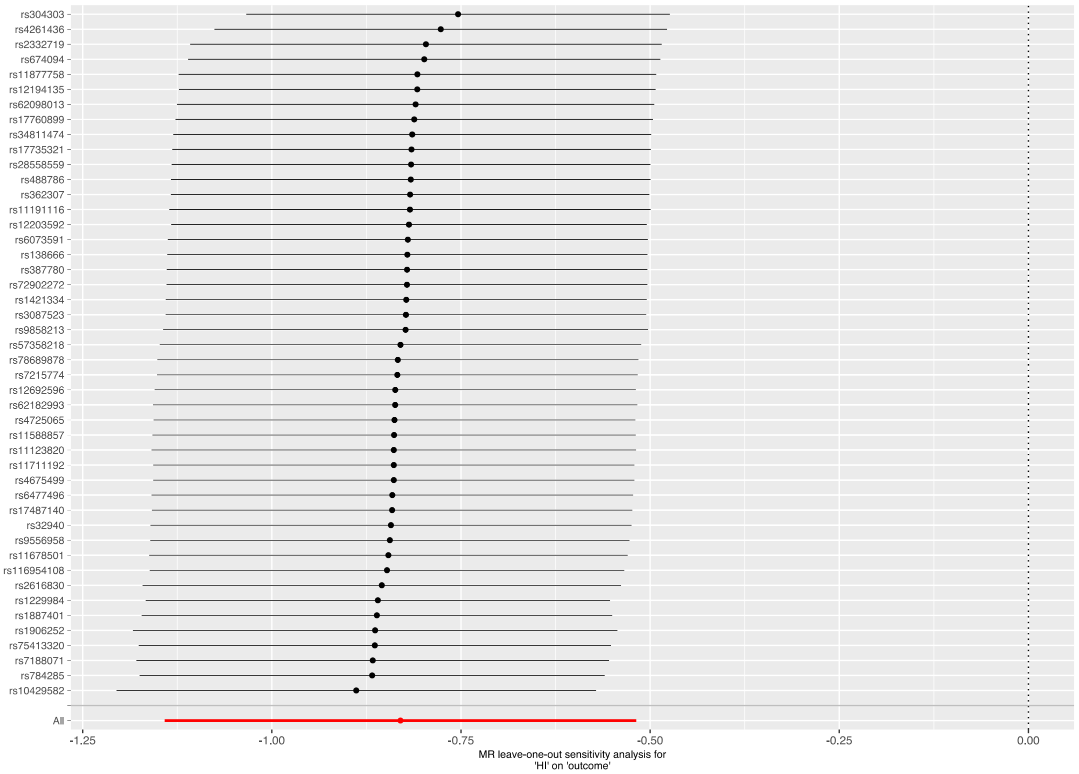

Supplementary Figure 39: leave-one-out analysis of household income against ADHD
Abbreviations: MR: Mendelian randomization; HI: household income; ADHD: attention deficit hyperactivity disorder.
Legend: n=46 SNPs. Forest plot of individual and combined SNP MR estimated effect size. The effect estimates represent the log-odds for ADHD per one standard deviation (1 SD) increase in the mean household income, and the error bars represent 95% CIs.

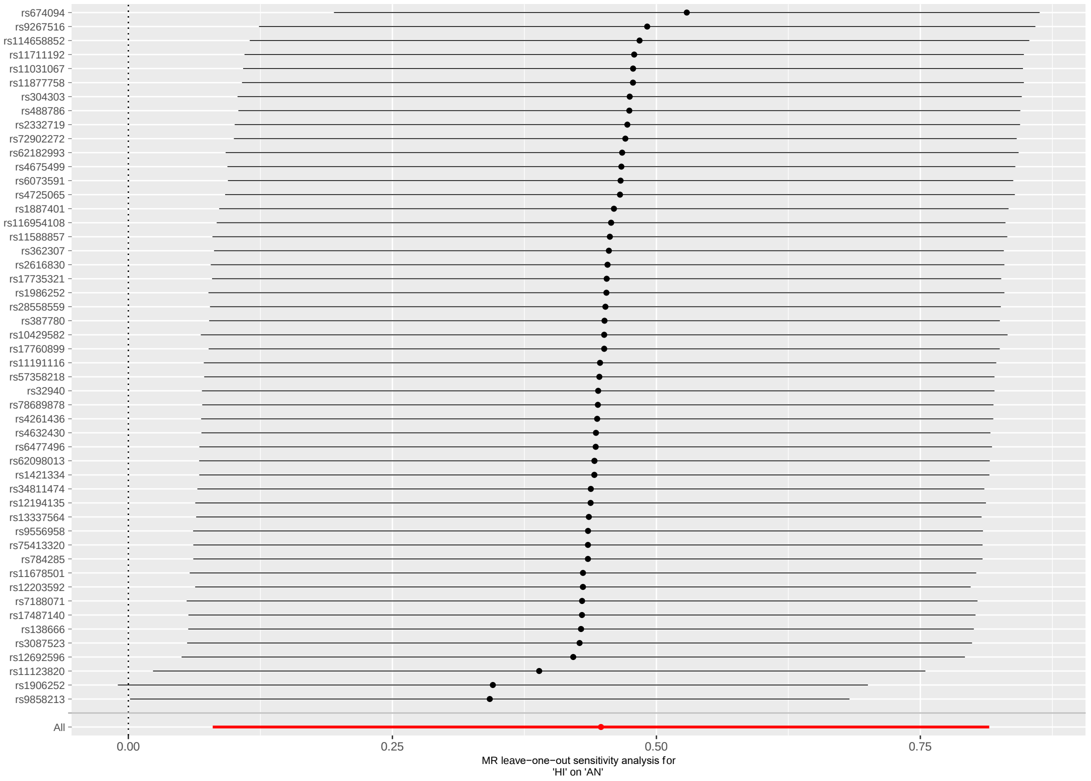

Supplementary Figure 40: leave-one-out analysis of household income against AN
Abbreviations: MR: Mendelian randomization; HI: household income; AN: anorexia nervosa.
Legend: n=50 SNPs. Forest plot of individual and combined SNP MR estimated effect size. The effect estimates represent the log-odds for AN per one standard deviation (1 SD) increase in the mean household income, and the error bars represent 95% CIs.

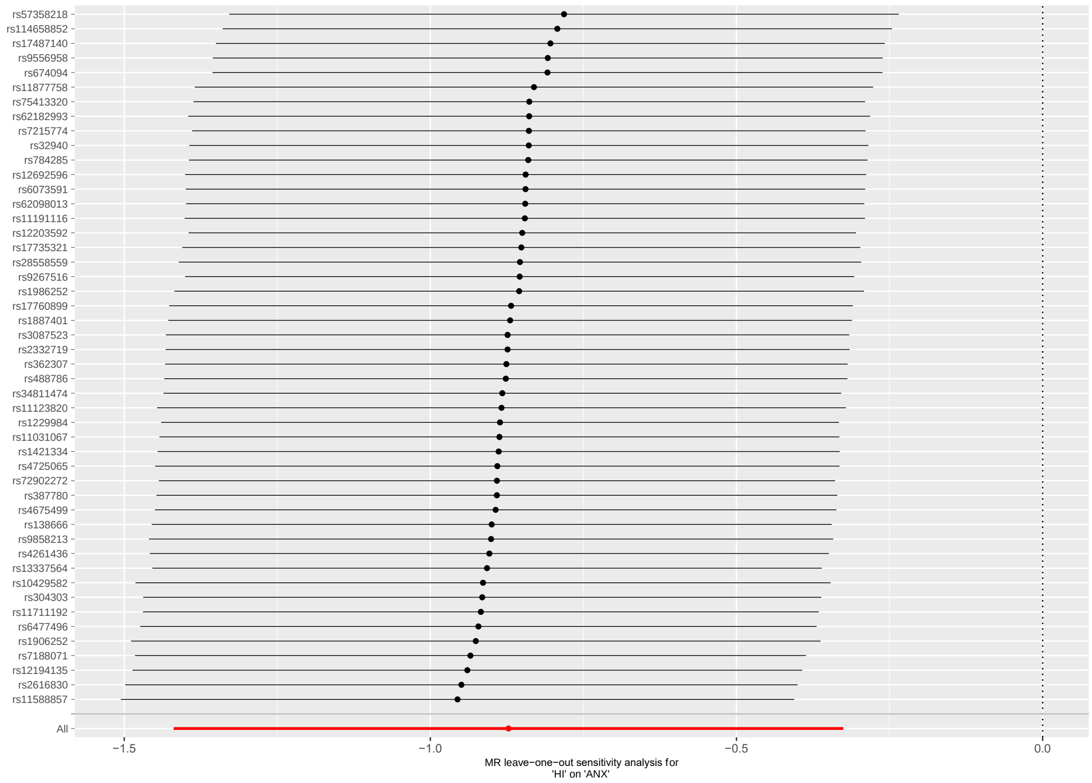

Supplementary Figure 41: leave-one-out analysis of household income against ANX
Abbreviations: MR: Mendelian randomization; HI: household income; ANX: anxiety disorder.
Legend: n=48 SNPs. Forest plot of individual and combined SNP MR estimated effect size. The effect estimates represent the log-odds for ANX per one standard deviation (1 SD) increase in the mean household income, and the error bars represent 95% CIs.

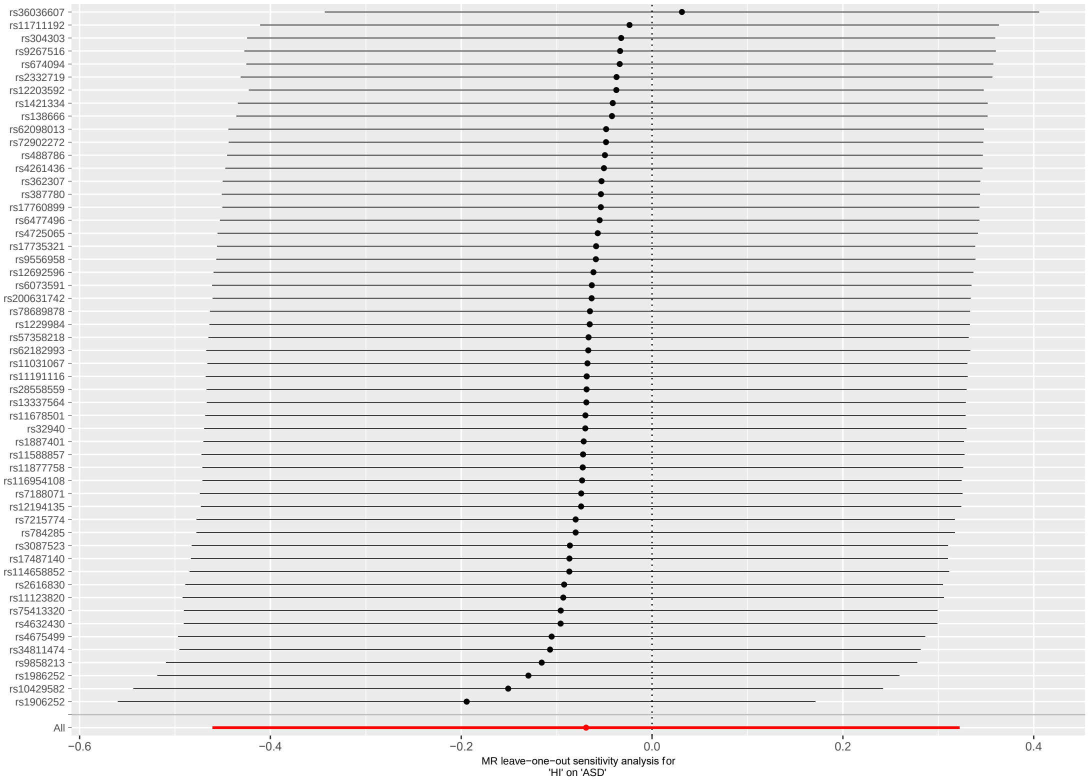

Supplementary Figure 42: leave-one-out analysis of household income against ASD
Abbreviations: MR: Mendelian randomization; HI: household income; ASD: autism spectrum disorders.
Legend: n=54 SNPs. Forest plot of individual and combined SNP MR estimated effect size. The effect estimates represent the log-odds for ASD per one standard deviation (1 SD) increase in the mean household income, and the error bars represent 95% CIs.

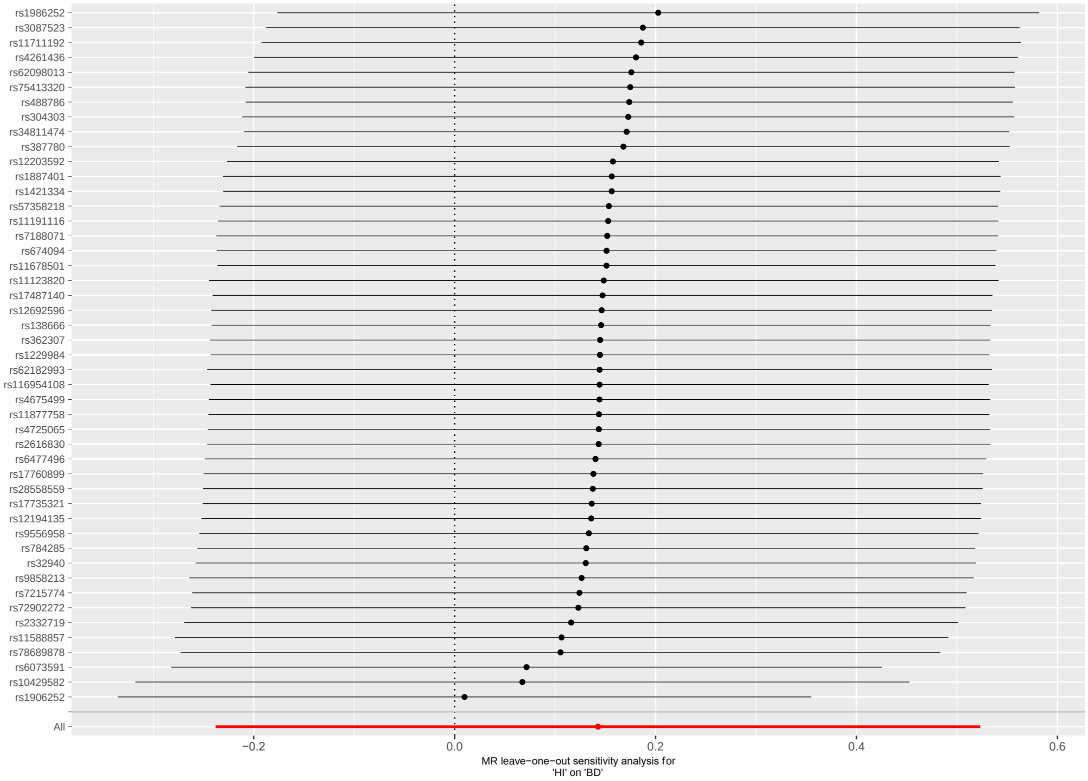

Supplementary Figure 43: leave-one-out analysis of household income against BD
Abbreviations: MR: Mendelian randomization; HI: household income; BD: bipolar disorder.
Legend: n=47 SNPs. Forest plot of individual and combined SNP MR estimated effect size. The effect estimates represent the log-odds for BD per one standard deviation (1 SD) increase in the mean household income, and the error bars represent 95% CIs.

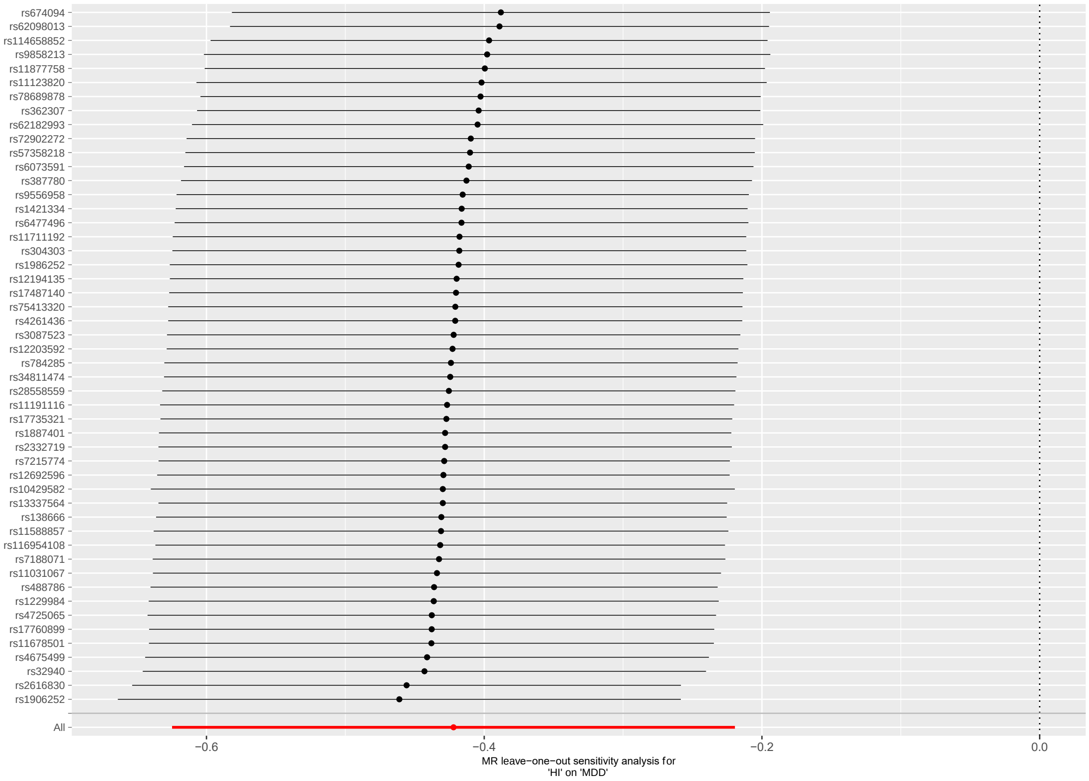

Supplementary Figure 44: leave-one-out analysis of household income against MDD
Abbreviations: MR: Mendelian randomization; HI: household income; MDD: major depressive disorder.
Legend: n=50 SNPs. Forest plot of individual and combined SNP MR estimated effect size. The effect estimates represent the log-odds for MDD per one standard deviation (1 SD) increase in the mean household income, and the error bars represent 95% CIs.

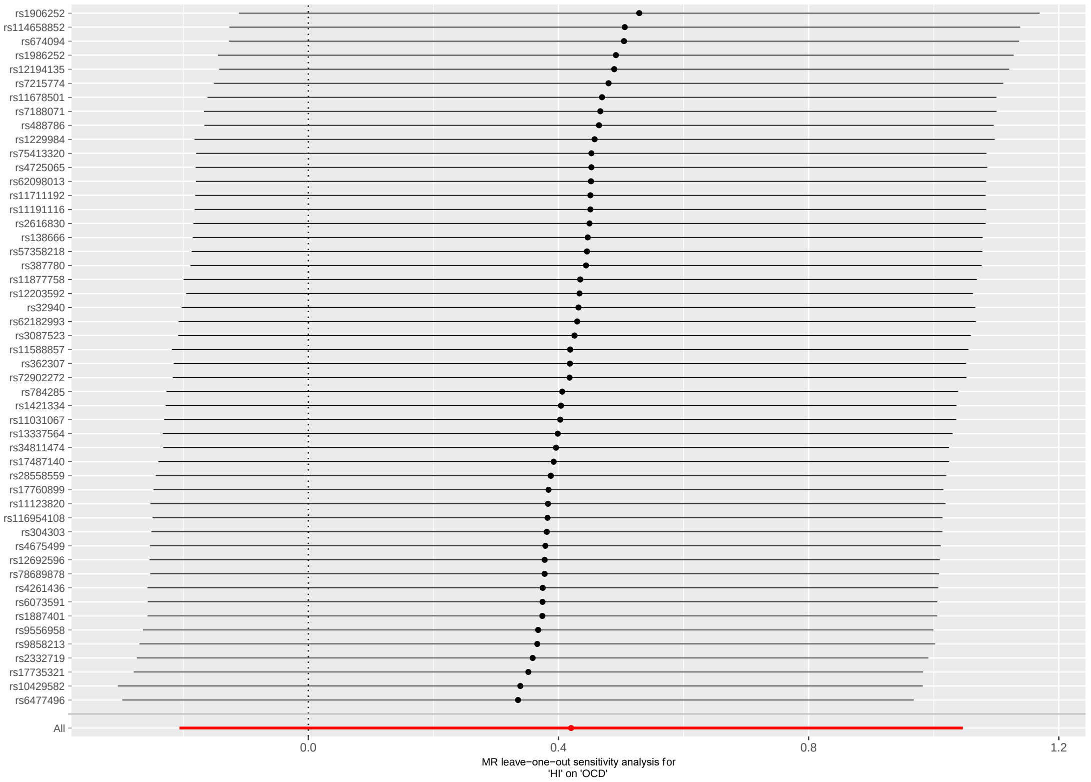

Supplementary Figure 45: leave-one-out analysis of household income against OCD
Abbreviations: MR: Mendelian randomization; HI: household income; OCD: obsessive compulsive disorder.
Legend: n=50 SNPs. Forest plot of individual and combined SNP MR estimated effect size. The effect estimates represent the log-odds for OCD per one standard deviation (1 SD) increase in the mean household income, and the error bars represent 95% CIs.

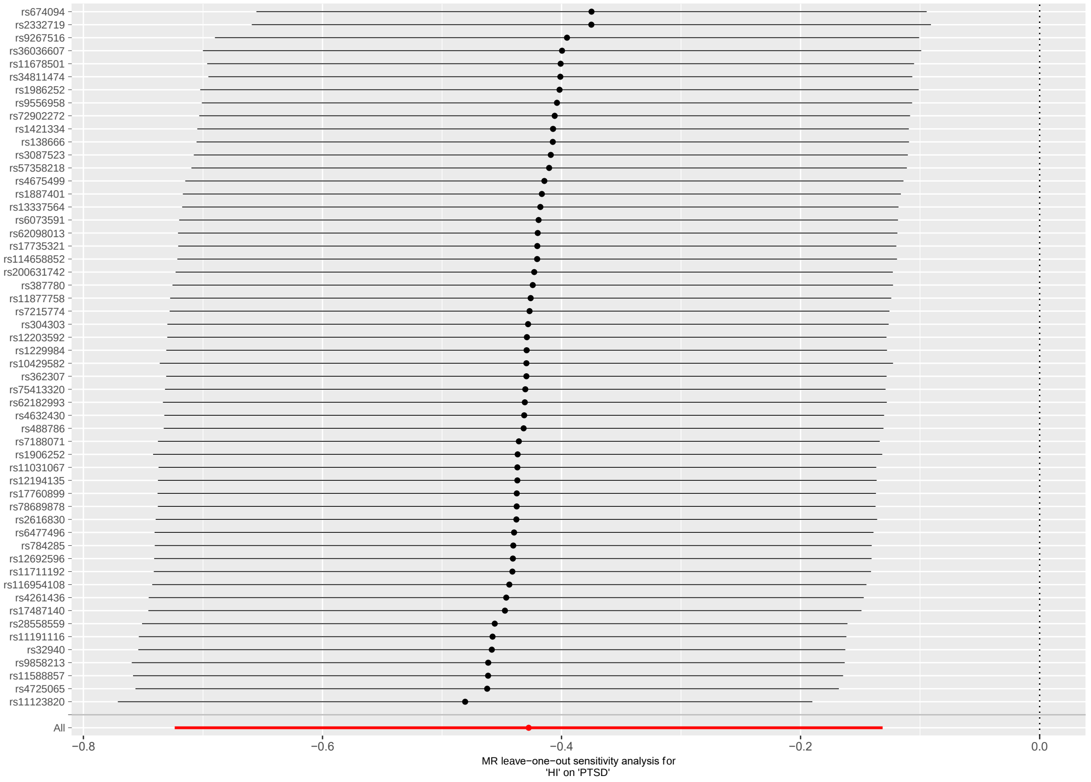

Supplementary Figure 46: leave-one-out analysis of household income against PTSD
Abbreviations: MR: Mendelian randomization; HI: household income; PTSD: post-traumatic stress disorder.
Legend: n=54 SNPs. Forest plot of individual and combined SNP MR estimated effect size. The effect estimates represent the log-odds for PTSD per one standard deviation (1 SD) increase in the mean household income, and the error bars represent 95% CIs.

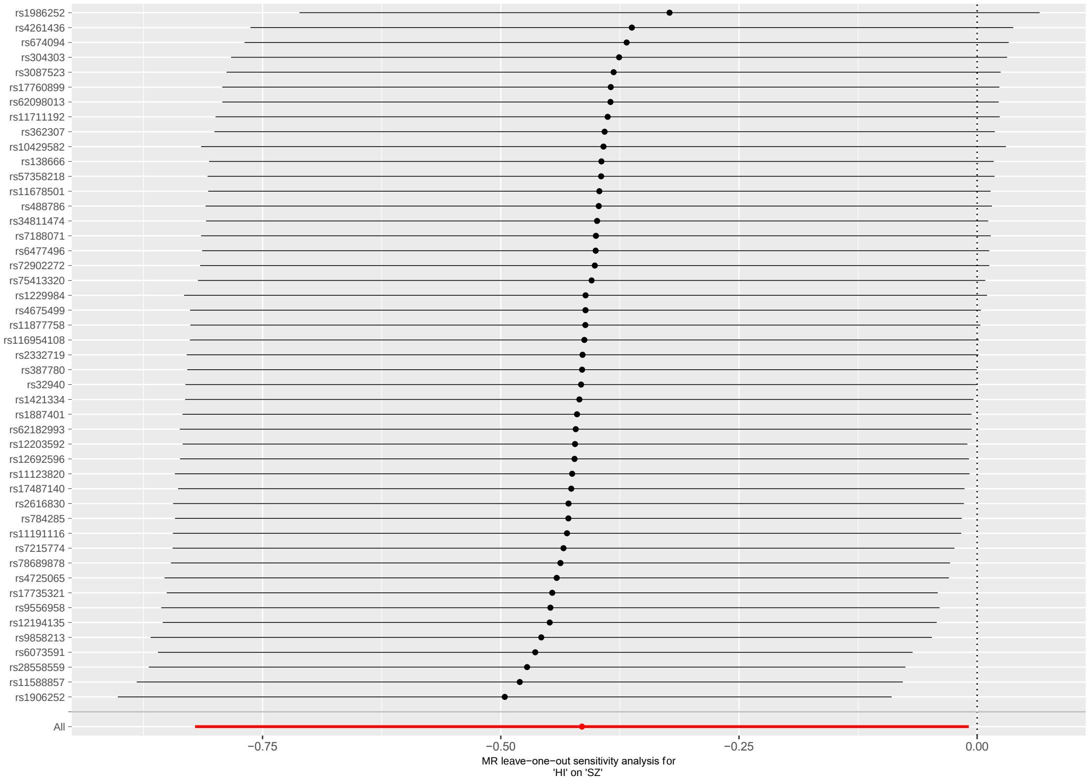

Supplementary Figure 47: leave-one-out analysis of household income against SZ
Abbreviations: MR: Mendelian randomization; HI: household income; SZ: schizophrenia.
Legend: n=47 SNPs. Forest plot of individual and combined SNP MR estimated effect size. The effect estimates represent the log-odds for SZ per one standard deviation (1 SD) increase in the mean household income, and the error bars represent 95% CIs.

Plots - Backward analyses

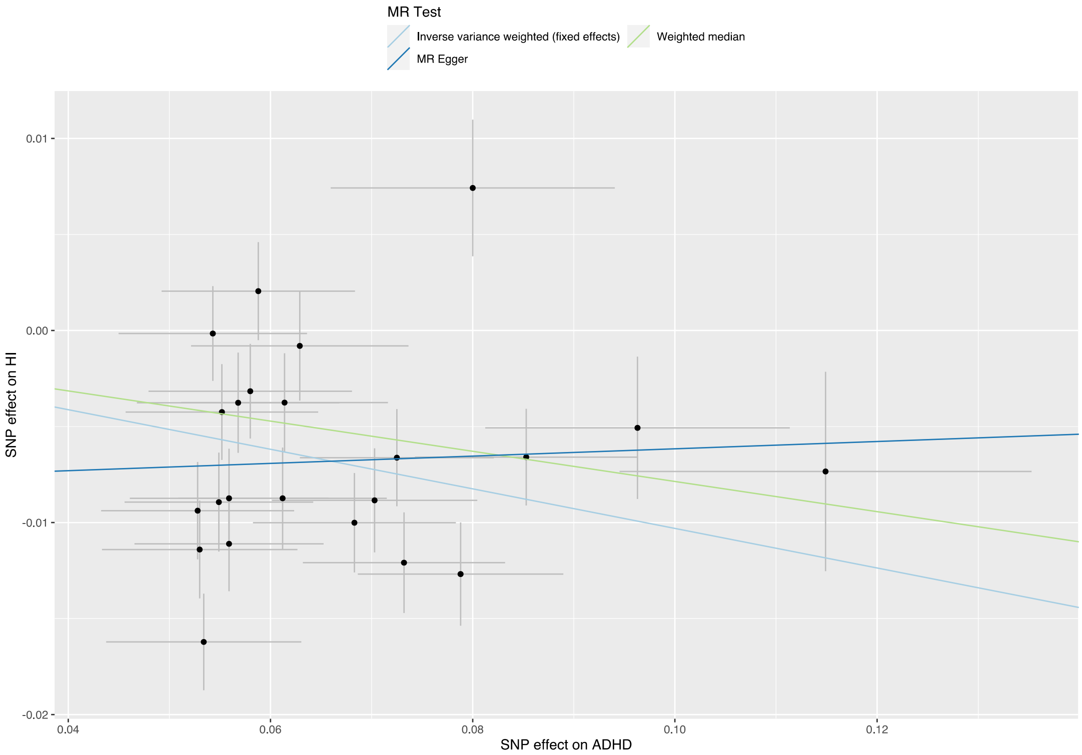

Supplementary Figure 48: scatterplot of ADHD against household income
Abbreviations: MR: Mendelian randomization; SNP: single nucleotide polymorphism; HI: household income; ADHD: attention deficit hyperactivity disorder.
Legend: n=23 SNPs. Each dot represents a SNP, with the slope of each line corresponding to the estimated MR effect per method.

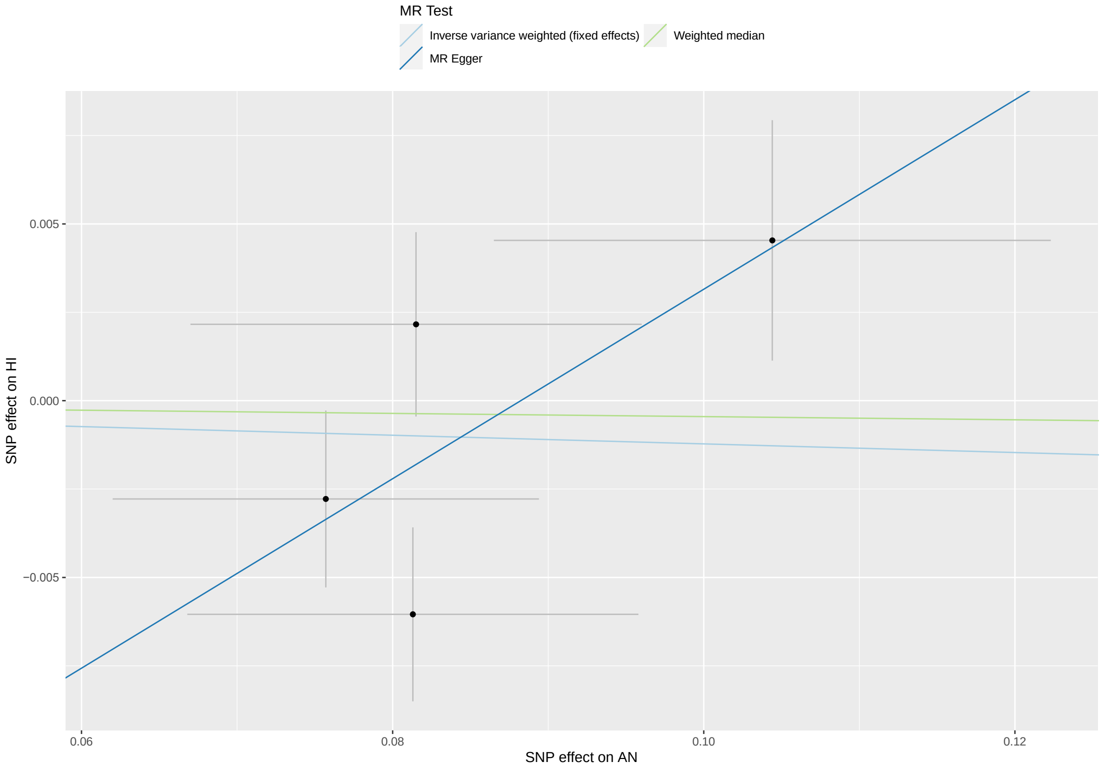

Supplementary Figure 49: scatterplot of AN against household income
Abbreviations: MR: Mendelian randomization; SNP: single nucleotide polymorphism; HI: household income; AN: anorexia nervosa.
Legend: n=4 SNPs. Each dot represents a SNP, with the slope of each line corresponding to the estimated MR effect per method.

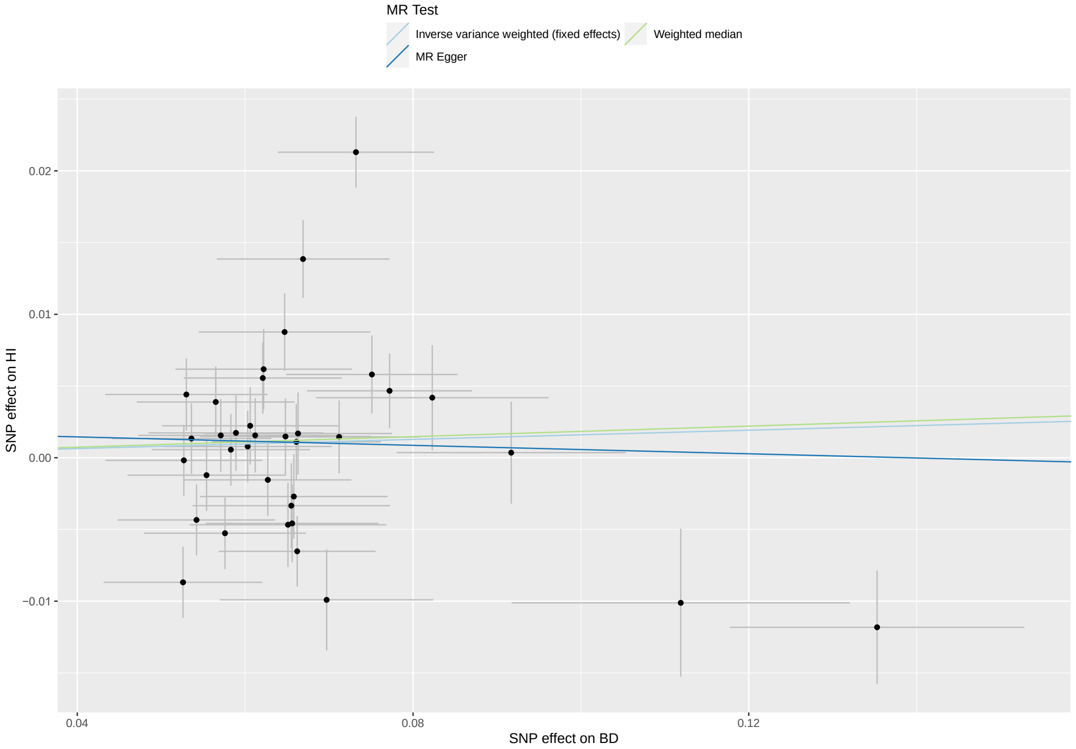

Supplementary Figure 50: scatterplot of BD against household income
Abbreviations: MR: Mendelian randomization; SNP: single nucleotide polymorphism; HI: household income; BD: bipolar disorder.
Legend: n=36 SNPs. Each dot represents a SNP, with the slope of each line corresponding to the estimated MR effect per method.

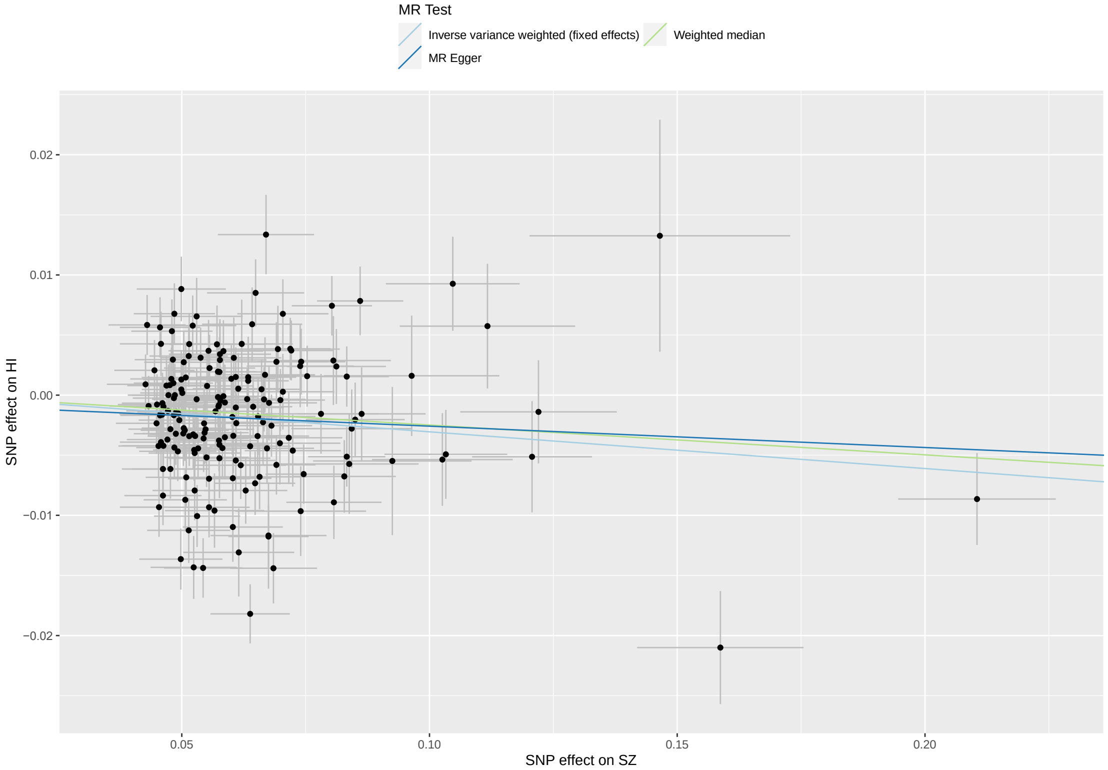

Supplementary Figure 51: scatterplot of SZ against household income
Abbreviations: MR: Mendelian randomization; SNP: single nucleotide polymorphism; HI: household income; SZ: schizophrenia.
Legend: n=176 SNPs. Each dot represents a SNP, with the slope of each line corresponding to the estimated MR effect per method.

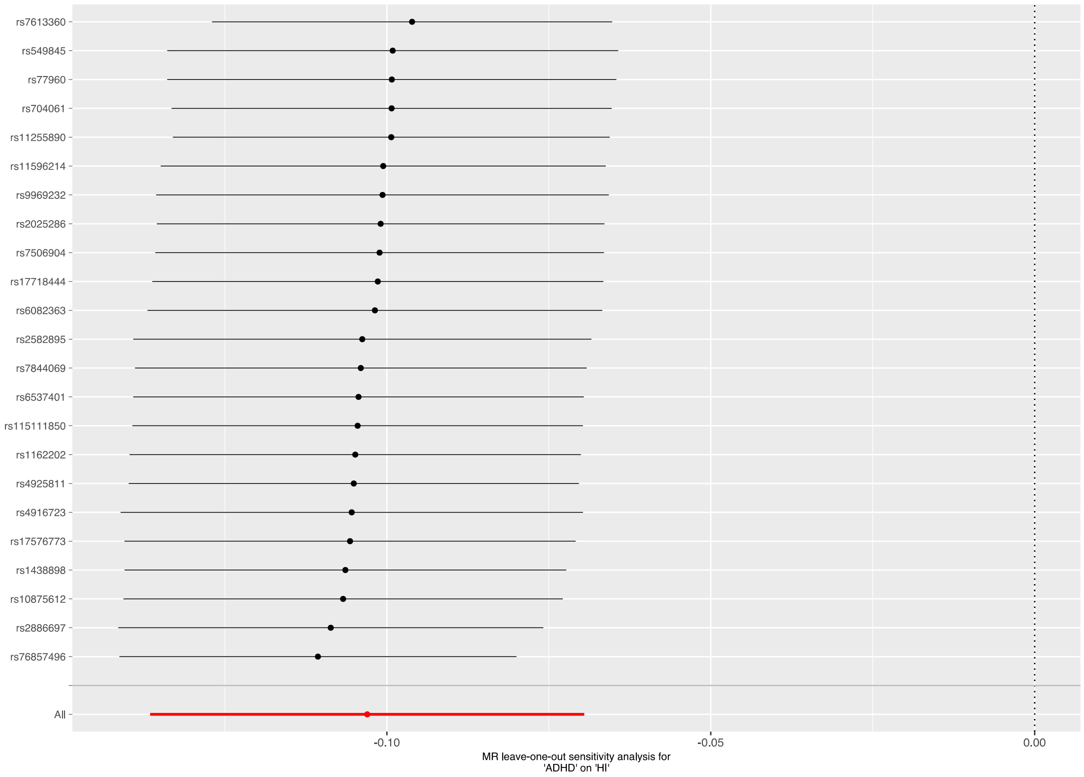

Supplementary Figure 52: leave-one-out analysis of ADHD against household income
Abbreviations: MR: Mendelian randomization; HI: household income; ADHD: attention deficit hyperactivity disorder.
Legend: n=23 SNPs. Forest plot of individual and combined SNP MR estimated effect size. The effect estimates represent the linear effect for the mean household income per ADHD status, and the error bars represent 95% CIs.

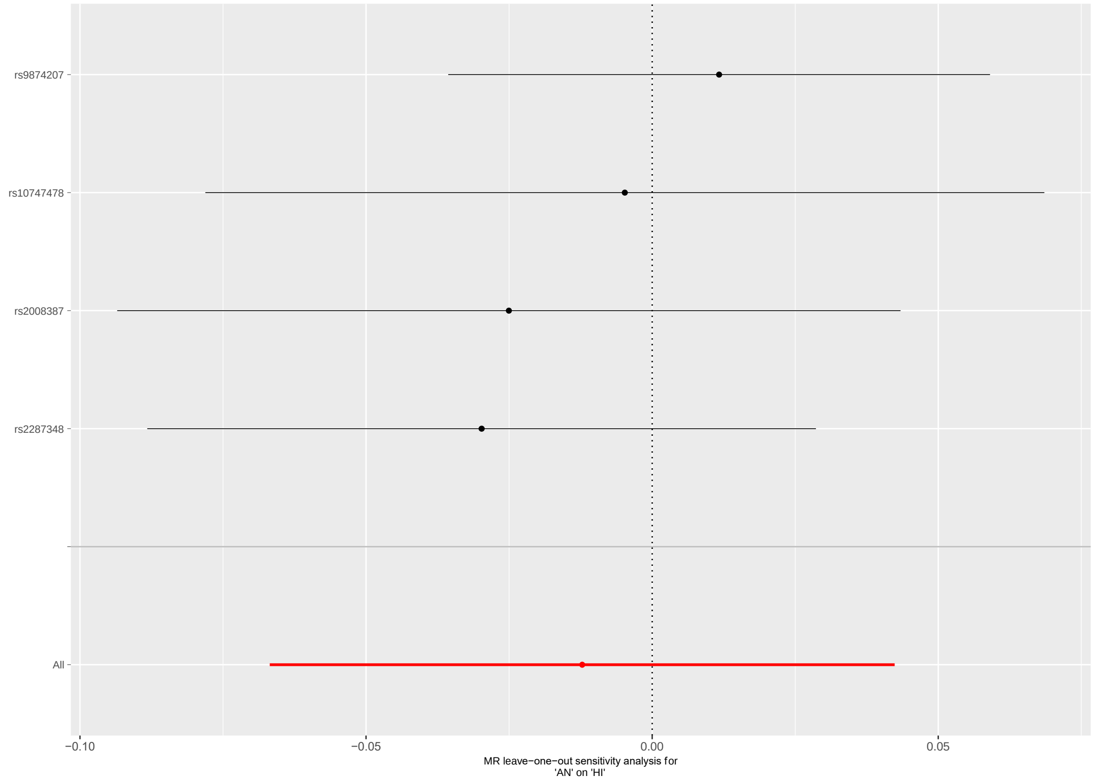

Supplementary Figure 53: leave-one-out analysis of AN against household income
Abbreviations: MR: Mendelian randomization; HI: household income; AN: anorexia nervosa.
Legend: n=4 SNPs. Forest plot of individual and combined SNP MR estimated effect size. The effect estimates represent the linear effect for the mean household income per AN status, and the error bars represent 95% CIs.

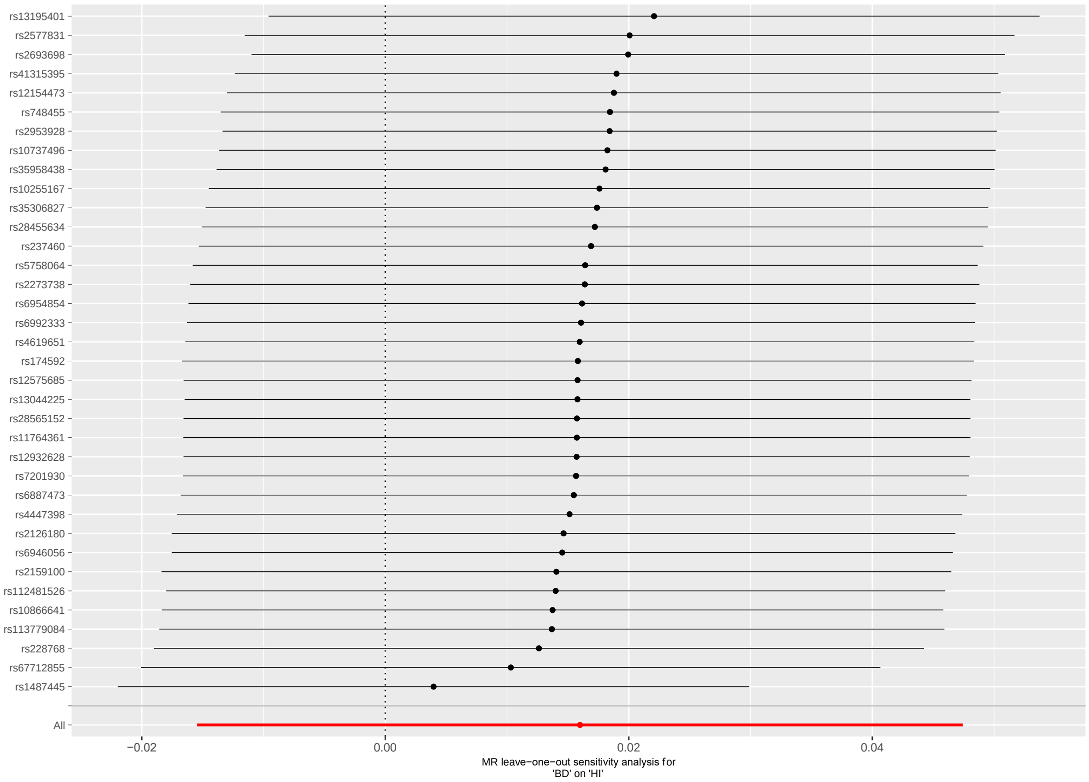

Supplementary Figure 54: leave-one-out analysis of BD against household income
Abbreviations: MR: Mendelian randomization; HI: household income; BD: bipolar disorder.
Legend: n=36 SNPs. Forest plot of individual and combined SNP MR estimated effect size. The effect estimates represent the linear effect for the mean household income per BD status, and the error bars represent 95% CIs.

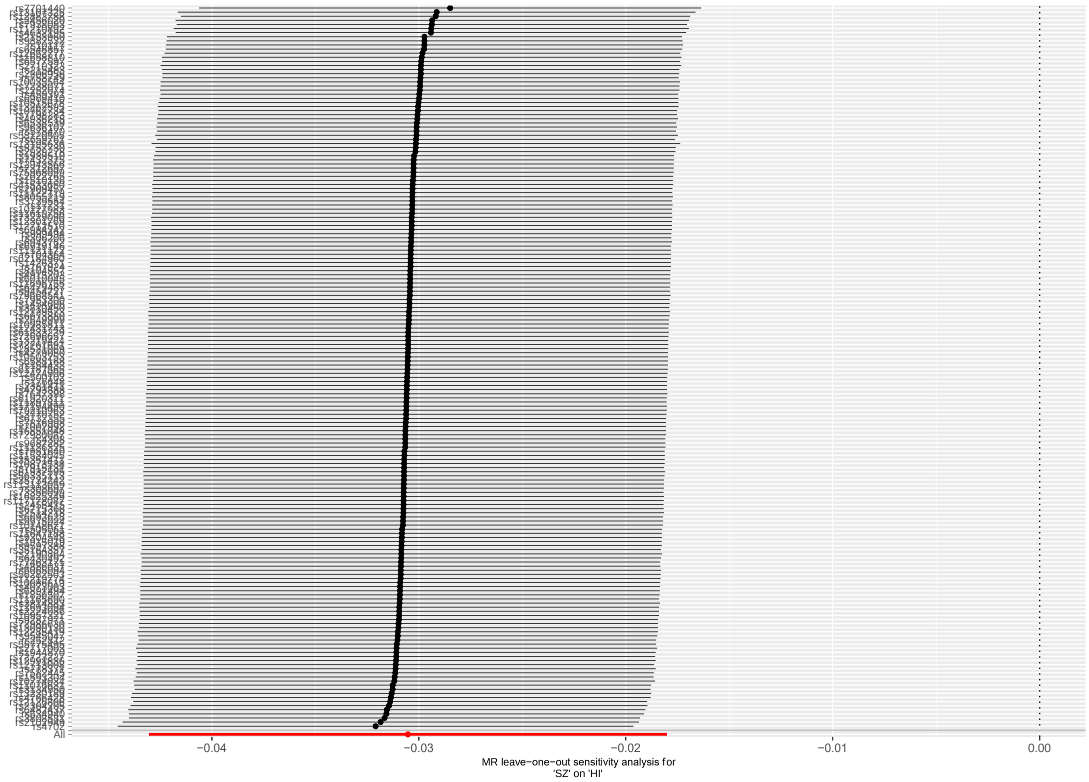

Supplementary Figure 55: leave-one-out analysis of SZ against household income
Abbreviations: MR: Mendelian randomization; HI: household income; SZ: schizophrenia.
Legend: n=176 SNPs. Forest plot of individual and combined SNP MR estimated effect size. The effect estimates represent the linear effect for the mean household income per SZ status, and the error bars represent 95% CIs.

Supplementary Table 11: CAUSE results of the relations between household income and mental illnesses

**Model 1**	**Model 2**	**Δ ELPD**	**SE Δ ELPD**	**z-score**	**p-value†**
		Fw: HI on ADHD			
Null	Sharing	-26.00	5.93	-4.39	**1.13×10^{-5}**
Null	Causal	-31.83	7.26	-4.38	**1.19×10^{-5}**
Sharing	Causal	-5.83	1.53	-3.80	**1.45×10^{-4}**
		Bw: ADHD on HI			
Null	Sharing	-17.77	4.47	-3.98	**6.89×10^{-5}**
Null	Causal	-23.53	6.00	-3.92	**8.85×10^{-5}**
Sharing	Causal	-5.76	1.60	-3.61	**3.06×10^{-4}**
		Fw: HI on AN			
Null	Sharing	-4.18	2.04	-2.05	**0.040**
Null	Causal	-8.41	3.89	-2.17	**0.030**
Sharing	Causal	-4.24	1.90	-2.24	**0.025**
		Bw: AN on HI			
Null	Sharing	0.45	0.06	7.08	**1.44×10^{-12}**
Null	Causal	1.15	0.54	2.15	**0.032**
Sharing	Causal	0.70	0.48	1.44	0.150
		Fw: HI on ANX			
Null	Sharing	-1.54	1.86	-0.83	0.407
Null	Causal	-2.04	2.70	-0.75	0.453
Sharing	Causal	-0.50	1.21	-0.41	0.682
		Bw: ANX on HI			
Null	Sharing	0.09	0.04	2.12	**0.034**
Null	Causal	0.62	0.36	1.70	0.089
Sharing	Causal	0.53	0.33	1.57	0.116
		Fw: HI on ASD			
Null	Sharing	0.49	0.07	7.16	**8.07×10^{-13}**
Null	Causal	1.39	0.26	5.32	**1.04×10^{-7}**
Sharing	Causal	0.90	0.21	4.20	**2.67×10^{-5}**
		Bw: ASD on HI			
Null	Sharing	-0.51	0.62	-0.83	0.407
Null	Causal	-3.19	2.44	-1.31	0.190
Sharing	Causal	-2.68	1.84	-1.46	0.144
		Fw: HI on BD			
Null	Sharing	0.44	0.11	4.12	**0.001**
Null	Causal	1.03	0.70	1.48	0.069
Sharing	Causal	0.59	0.61	0.98	0.327
		Bw: BD on HI			
Null	Sharing	0.43	0.03	13.18	**1.14×10^{-39}**
Null	Causal	1.35	0.11	11.76	**6.27×10^{-32}**
Sharing	Causal	0.92	0.11	8.19	

					**2.61×10^{-16}**
Fw: HI on MDD					
Null	Sharing	-11.26	4.00	-2.82	**0.005**
Null	Causal	-15.12	5.31	-2.85	**0.004**
Sharing	Causal	-3.86	1.54	-2.50	**0.012**
Bw: MDD on HI					
Null	Sharing	-0.51	0.62	-0.83	0.407
Null	Causal	-3.19	2.44	-1.31	0.190
Sharing	Causal	-2.68	1.84	-1.46	0.144
Fw: HI on OCD					
Null	Sharing	-0.10	0.72	-0.14	0.889
Null	Causal	-0.55	1.94	-0.28	0.779
Sharing	Causal	-0.45	1.25	-0.36	0.719
Bw: OCD on HI					
Null	Sharing	0.27	0.08	3.30	**0.001**
Null	Causal	0.92	0.52	1.77	0.077
Sharing	Causal	0.65	0.45	1.44	0.150
Fw: HI on PTSD					
Null	Sharing	-6.47	3.09	-2.09	**0.037**
Null	Causal	-8.97	4.47	-2.00	**0.046**
Sharing	Causal	-2.50	1.53	-1.64	0.101
Bw: PTSD on HI					
Null	Sharing	0.18	0.10	1.74	0.082
Null	Causal	0.24	0.91	0.26	0.795
Sharing	Causal	0.06	0.81	0.07	0.944
Fw: HI on SZ					
Null	Sharing	-1.55	0.91	-1.70	0.089
Null	Causal	-6.27	3.00	-2.09	**0.037**
Sharing	Causal	-4.73	2.10	-2.25	**0.024**
Bw: SZ on HI					
Null	Sharing	-2.78	1.61	-1.73	0.084
Null	Causal	-6.76	3.53	-1.91	0.056
Sharing	Causal	-3.98	1.94	-2.05	**0.040**

Abbreviations: MR: mendelian randomization; ELPD: expected log pointwise posterior density; 95%CI: 95% confidence interval; SE: standard error; Fwd: forward MR; Bwd: backward MR; HI: household income; ADHD: attention deficit hyperactivity disorder; AN: anorexia nervosa; ANX: anxiety disorder; ASD: autism spectrum disorder; BD: bipolar disorder; MDD: major depressive disorder; OCD: obsessive-compulsive disorder; PTSD: post-traumatic stress disorder; SZ: schizophrenia.

Legend: † Based on t-test. The p-values are two-sided and not adjusted for multiple testing, therefore p-value<0.05 was considered significant (and reported in bolded text).

Supplementary Table 12: Results of univariable bidirectional Mendelian Randomization of household income against mental illnesses, after Steiger filtering

**MR**	**N SNP**	**IVW, B (95% CI)**	**IVW p-value**	**WM, B (95% CI)**	**WM p-value**	**MR-Egger, B (95% CI)**	**MR-Egger p-value**	**Egger intercept p-value**	**Mean F**
HI on ADHD	44	-0.698 (-0.885; -0.511)	**2.54×10^{-13}**	-0.549 (-0.856; 0.243)	**5.53×10^{-4}**	0.366 (-0.716; 1.45)	0.511	**0.022**	36.8
HI on AN	47	0.315 (0.045; 0.585)	**0.022**	0.301 (-0.101; 0.702)	0.142	-0.018 (-1.29; 1.25)	0.978	0.315	36.1
HI on ANX	43	-0.473 (-1.04; 0.097)	0.104	-0.206 (-1.01; 0.596)	0.615	0.619 (-2.41; 3.65)	0.690	0.075	37.8
HI on ASD	44	-0.044 (-0.321; 0.233)	0.754	-0.092 (-0.500; 0.317)	0.660	0.791 (-0.362; 1.94)	0.186	**4.88×10^{-4}**	36.2
HI on BD	36	0.148 (-0.058; 0.354)	0.160	0.083 (-0.219; 0.386)	0.589	0.547 (-0.476; 1.57)	0.302	**0.021**	36.7
HI on MDD	48	-0.354 (-0.524; -0.183)	**4.63×10^{-5}**	-0.288 (-0.549; -0.027)	**0.031**	-0.650 (-1.42; 0.116)	0.103	0.930	37.1
HI on OCD	29	0.043 (-0.820; 0.734)	0.913	-0.284 (-1.35; 0.785)	0.602	0.322 (-2.91; 3.56)	0.847	0.578	40.3
HI on PTSD	54	-0.427 (-0.693; -0.161)	**0.002**	-0.306 (-0.708; 0.096)	0.135	-0.317 (-1.59; 0.952)	0.626	0.861	37.5
HI on SZ	44	-0.214 (-0.370; -0.059)	**0.007**	-0.405 (-0.695; -0.114)	**0.006**	-0.374 (-1.82; 1.07)	0.615	0.207	36.9

Abbreviations: HI: household income; ADHD: attention deficit hyperactivity disorder; AN: anorexia nervosa; ANX: anxiety disorder; ASD: autism spectrum disorders; BD: bipolar disorder; MDD: major depressive disorder; OCD: obsessive-compulsive disorder; PTSD: post-traumatic stress disorder; SZ: schizophrenia; MR: mendelian randomization; SNP: single nucleotide polymorphism; IVW: inverse variance weighted (fixed effect); B: effect estimates are log-odds; 95% CI: 95% confidence interval; WM: weighted median; NR: not reported because not enough SNP to perform MR.

Legend: All statistical tests were two-sided. The p-values were not adjusted for multiple testing, therefore p-value<0.05 was considered significant (and reported in bolded text).

Univariable Mendelian randomization of occupational income and mental illnesses

Supplementary Table 13: Odds Ratio of univariable forward Mendelian randomization analysis of occupational income against mental illnesses

**MR: method**	**OR (95% CI)**	**p-value**
OI → ADHD:		
IVW	0.424 (0.350; 0.512)	**7.75×10^{-19}**
WM	0.564 (0.410; 0.776)	**5.61×10^{-4}**
MR-Egger	1.14 (0.215; 6.05)	0.878
OI→ AN:		
IVW	1.84 (1.40; 2.41)	**1.10×10^{-5}**
WM	1.55 (0.980; 2.45)	0.054
MR-Egger	12.3 (1.89; 80.3)	**0.013**
OI → ANX:		
IVW	0.896 (0.510; 1.57)	0.702
WM	0.879 (0.385; 2.00)	0.749
MR-Egger	2.15 (0.095; 49.0)	0.634
OI → ASD:		
IVW	1.60 (1.21; 2.12)	**0.001**
WM	1.40 (0.872; 2.26)	0.167
MR-Egger	10.6 (1.32; 85.1)	**0.034**
OI → BD:		
IVW	1.22 (1.02; 1.47)	**0.033**
WM	1.15 (0.828; 1.59)	0.407
MR-Egger	6.58 (1.14; 37.9)	**0.043**
OI → MDD:		
IVW	0.724 (0.605; 0.867)	**4.37×10^{-4}**
WM	0.883 (0.655; 1.19)	0.408
MR-Egger	1.36 (0.333; 5.57)	0.671
OI → OCD:		
IVW	1.82 (0.925; 3.59)	0.083
WM	1.70 (0.620; 4.66)	0.290
MR-Egger	0.116 (0.004; 3.09)	0.208
OI → PTSD:		
IVW	0.950 (0.707; 1.28)	0.736
WM	0.763 (0.497; 1.17)	0.235
MR-Egger	0.665 (0.105; 4.23)	0.669
OI → SZ:		
IVW	0.830 (0.709; 0.971)	**0.020**
WM	0.839 (0.607; 1.16)	0.272
MR-Egger	1.22 (0.126; 11.9)	0.863

Abbreviations: MR: Mendelian randomization; OR: Odds Ratio; 95% CI: 95% confidence intervals; OI: occupational income; ADHD: attention deficit hyperactivity disorder; AN: anorexia nervosa; ANX: anxiety disorder; ASD: autism spectrum disorders; BD: bipolar disorder; MDD: major depressive disorder; OCD: obsessive-compulsive disorder; PTSD: post-traumatic stress disorder; SZ: schizophrenia; IVW: inverse variance weighted (fixed effect); WM: weighted median; NR: not reported because not enough SNP to perform MR. Legend: All statistical tests were two-sided. The p-values were not adjusted for multiple testing, therefore p-value<0.05 was considered significant (and reported in bolded text).

Supplementary Table 14: results of bidirectional MR of Occupational Income (OI) against mental health traits

**MR**	**N SNP**	**IVW, B (95% CI)**	**IVW p-value**	**IVW Q(df)**	**Q p-value**[†]	**WM, B (95% CI)**	**WM p-value**	**MR-Egger, B (95% CI)**	**MR-Egger p-value**	**Egger intercept p-value**	**Steiger Test p-value**[‡]	**MR-PRESSO**	**Mean F**
Fw: OI on ADHD	33	-0.859 (-1.05; -0.669)	**7.75×10^{-19}**	100 (32)	**6.68×10^{-9}**	-0.572 (-0.891; -0.253)	**5.61×10^{-4}**	0.132 (-1.54; 1.80)	0.878	0.243	**1.18×10^{-45}**	DT; p=0.560	40.3
Bw: ADHD on OI	23	-0.102 (-0.120; -0.083)	**2.93×10^{-27}**	113 (22)	**2.92×10^{-14}**	-0.102 (-0.135; -0.069)	**3.30×10^{-11}**	-0.052 (-0.297; 0.192)	0.679	0.692	**3.69×10^{-33}**	DT; p=0.598	39.2
Fw: OI on AN	34	0.606 (0.337; 0.879)	**1.10×10^{-5}**	72 (33)	**1.50×10^{-4}**	0.437 (-0.007; 0.882)	0.054	2.51 (0.635; 4.39)	**0.013**	0.051	**1.50×10^{-16}**	DT; p=0.392	40.4
Bw: AN on OI	4	0.045 (0.011; 0.080)	**0.009**	5 (3)	0.204	0.048 (0.002; 0.094)	**0.042**	-0.056 (-0.500; 0.388)	0.827	0.696	**1.05×10^{-17}**	GT; p=0.299	31.9
Fw: OI on ANX	32	-0.110 (-0.672; 0.453)	0.702	39 (31)	0.163	-0.129 (-0.920; 0.661)	0.749	0.767 (-2.36; 3.89)	0.634	0.578	NR [b]	GT; p=0.164	40.6
Bw: ANX on OI	0	NR [c]	NR [c]	NR [c]	NR [c]	NR [c]	NR [c]	NR [c]	NR [c]	NR [c]	NR [c]	NR [c]	NR [c]
Fw: OI on ASD	33	0.471 (0.192; 0.750)	**0.001**	82 (32)	**3.22×10^{-6}**	0.340 (-0.142; 0.822)	0.167	2.36 (0.281; 4.44)	**0.034**	0.078	**5.50×10^{-6}**	DT; p=0.200	40.3
Bw: ASD on OI	0	NR [c]	NR [c]	NR [c]	NR [c]	NR [c]	NR [c]	NR [c]	NR [c]	NR [c]	NR [c]	NR [c]	NR [c]
Fw: OI on BD	34	0.201 (0.016; 0.386)	**0.033**	136 (33)	**2.12×10^{-14}**	0.137 (-0.187; 0.461)	0.407	1.88 (0.135; 3.63)	**0.043**	0.063	**3.43×10^{-4}**	DT; p=0.096	40.4
Bw: BD on OI	36	0.042 (0.028; 0.057)	**1.90×10^{-8}**	147 (35)	**3.26×10^{-15}**	0.020 (-0.005; 0.046)	0.116	0.068 (-0.099; 0.235)	0.431	0.763	**7.03×10^{-206}**	DT; p=0.174	39.2
Fw: OI on MDD	33	-0.322 (-0.502; -0.143)	**4.37×10^{-4}**	83 (32)	**3.05×10^{-6}**	-0.125 (-0.420; 0.171)	0.408	0.309 (-1.10; 1.72)	0.671	0.376	**9.81×10^{-40}**	DT; p=0.849	40.3
Bw: MDD on OI	0	NR [c]	NR [c]	NR [c]	NR [c]	NR [c]	NR [c]	NR [c]	NR [c]	NR [c]	NR [c]	NR [c]	NR [c]

Fw: OI on OCD	33	0.600 (-0.078; 1.28)	0.083	34 (32)	0.362	0.530 (-0.453; 1.51)	0.290	-2.15 (-5.44; 1.13)	0.208	0.103	NR [b]	GT; p=0.361	40.3
Bw: OCD on OI	0	NR [c]	NR [c]	NR [c]	NR [c]	NR [c]	NR [c]	NR [c]	NR [c]	NR [c]	NR [c]	NR [c]	NR [c]
Fw: OI on PTSD	33	-0.051 (-0.347; 0.245)	0.736	53 (32)	**0.012**	-0.271 (-0.717; 0.176)	0.235	-0.407 (-2.26; 1.44)	0.669	0.702	NR [b]	DT; p=0.934	40.3
Bw: PTSD on OI	0	NR [c]	NR [c]	NR [c]	NR [c]	NR [c]	NR [c]	NR [c]	NR [c]	NR [c]	NR [c]	NR [c]	NR [c]
Fw: OI on SZ	34	-0.187 (-0.344; -0.030)	**0.020**	278 (33)	**2.06×10^{-40}**	-0.176 (-0.489; 0.138)	0.272	0.202 (-2.07; 2.48)	0.863	0.734	**3.65×10^{-54}**	DT; p=0.818	40.4
Bw: SZ on OI	176	-0.010 (-0.017; -0.003)	**0.008**	512 (175)	**9.74×10^{-35}**	-0.008 (-0.021; 0.005)	0.228	0.005 (-0.044; 0.055)	0.830	0.526	**$<1^{-1000}$**	DT; p=0.065	45.6

Abbreviations: Fw: forward analysis; Bw: backward analysis; OI: occupational income; ADHD: attention deficit hyperactivity disorder; AN: anorexia nervosa; ANX: anxiety disorder; ASD: autism spectrum disorder; BD: bipolar disorder; MDD: major depressive disorder; OCD: obsessive-compulsive disorder; PTSD: post-traumatic stress disorder; SZ: schizophrenia; MR: mendelian randomization; SNP: single nucleotide polymorphism; IVW: inverse variance weighted (fixed effect); B: effect estimates are log-odds for binary traits (i.e., for mental illnesses) and unstandardized regression coefficient for continuous traits (i.e., for occupational income); 95% CI: 95% confidence interval; Q: Cochran's Q measure of heterogeneity; df: degree of freedom; WM: weighted median; DT: distortion test; GT: global test.

Legend:

P-value threshold for SNP selection <5e-8.

[a] The Mendelian randomization pleiotropy residual sum and outlier (MR-PRESSO) test identifies possible bias from horizontal pleiotropy. The test consists of three parts, (1) the MR-PRESSO global test which detects horizontal pleiotropy, (2) the outlier corrected causal estimate which corrects for the detected horizontal pleiotropy and (3) the MR-PRESSO distortion test which estimates if the causal estimate is significantly different (at p<0.05) after adjustment for outliers. We conduct all three stages (with the argument NbDistribution=1000, namely using1000 simulation form the null distribution to compute empirical p-values) and present the outlier adjusted causal estimates (OACE) when both global and distortion tests are significant.

[b] We did not run Steiger Test if none of the MR analysis resulted significant (NR: not reported in the cell).

[c] Not enough SNP to perform MR (NR: not reported in the cell).

† Based on chi^2 test.

‡ Based on t-test.

All statistical tests were two-sided. The p-values were not adjusted for multiple testing, therefore p-value<0.05 was considered significant (and reported in bolded text).

Plots - Forward analyses

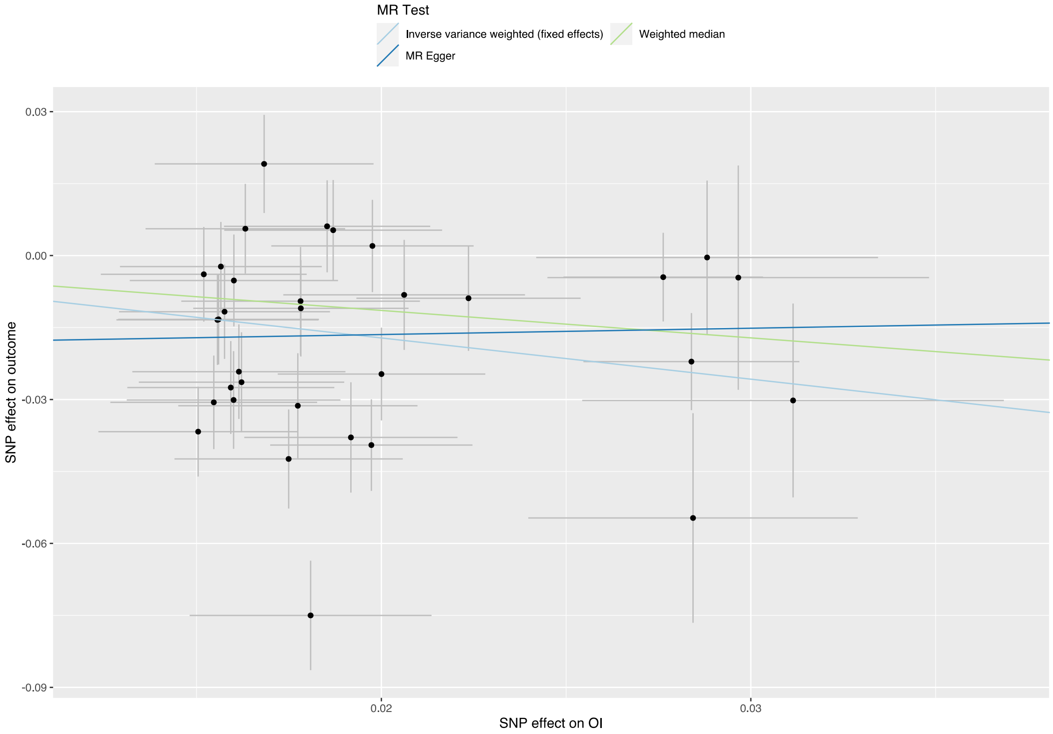

Supplementary Figure 56: scatterplot of occupational income against ADHD
Abbreviations: MR: Mendelian randomization; SNP: single nucleotide polymorphism; OI: occupational income; ADHD: attention deficit hyperactivity disorder.
Legend: n=33 SNPs. Each dot represents a SNP, with the slope of each line corresponding to the estimated MR effect per method.

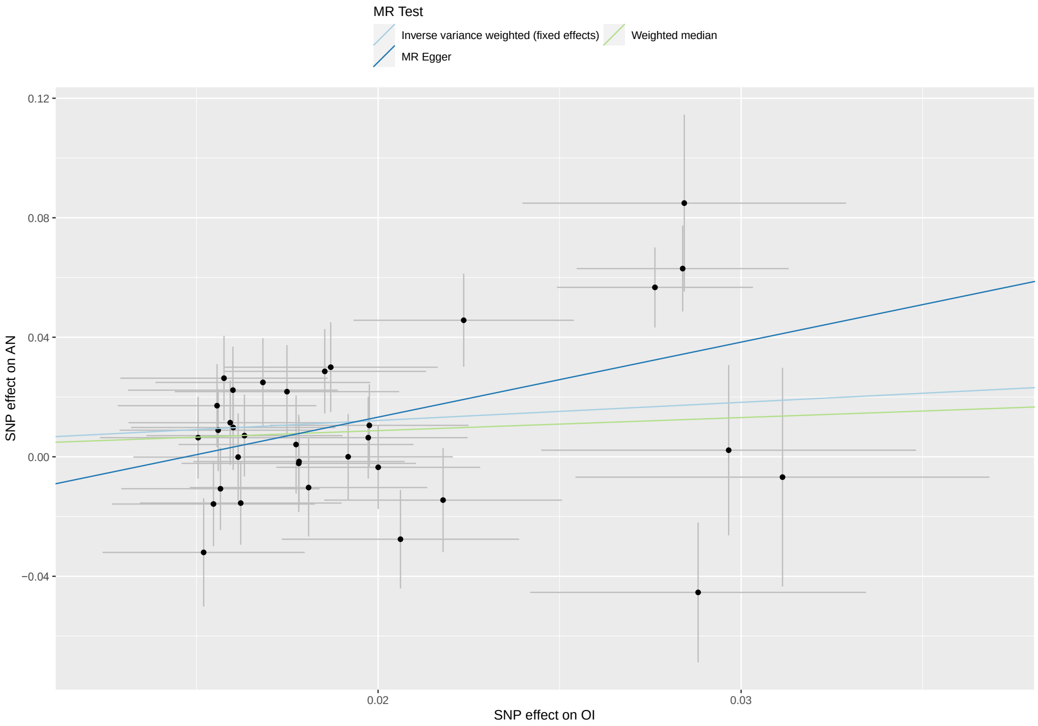

Supplementary Figure 57: scatterplot of occupational income against AN
Abbreviations: MR: Mendelian randomization; SNP: single nucleotide polymorphism; OI: occupational income; AN: anorexia nervosa.
Legend: n=34 SNPs. Each dot represents a SNP, with the slope of each line corresponding to the estimated MR effect per method.

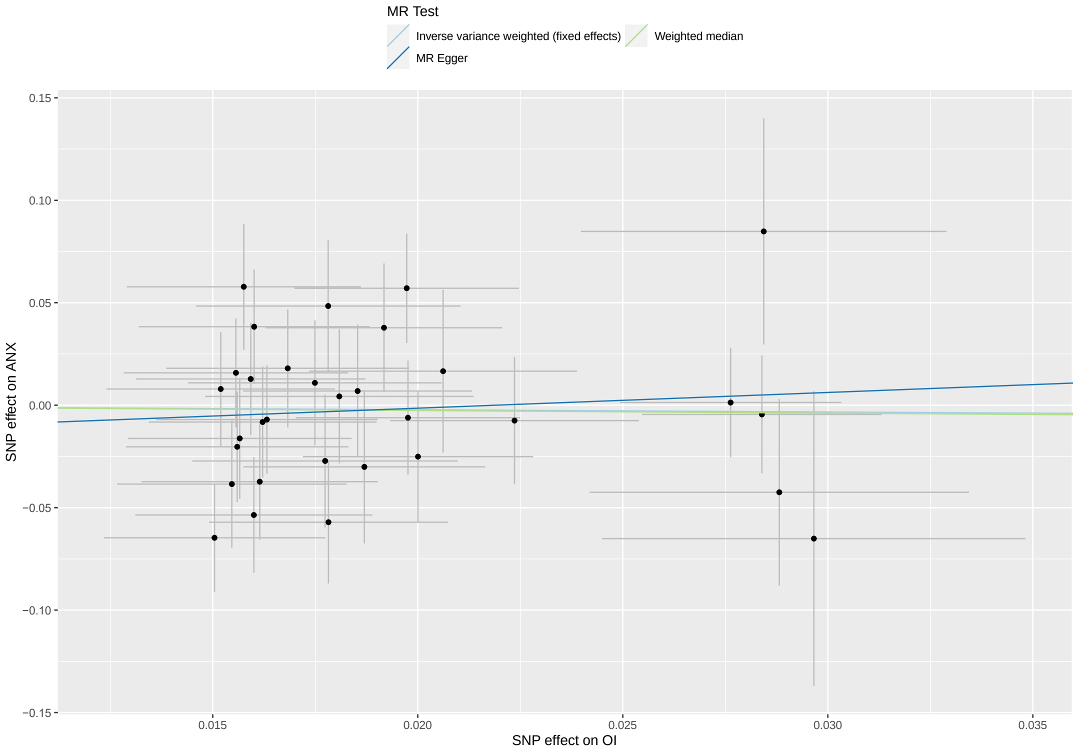

Supplementary Figure 58: scatterplot of occupational income against ANX
Abbreviations: MR: Mendelian randomization; SNP: single nucleotide polymorphism; OI: occupational income; ANX: anxiety disorders.
Legend: n=32 SNPs. Each dot represents a SNP, with the slope of each line corresponding to the estimated MR effect per method.

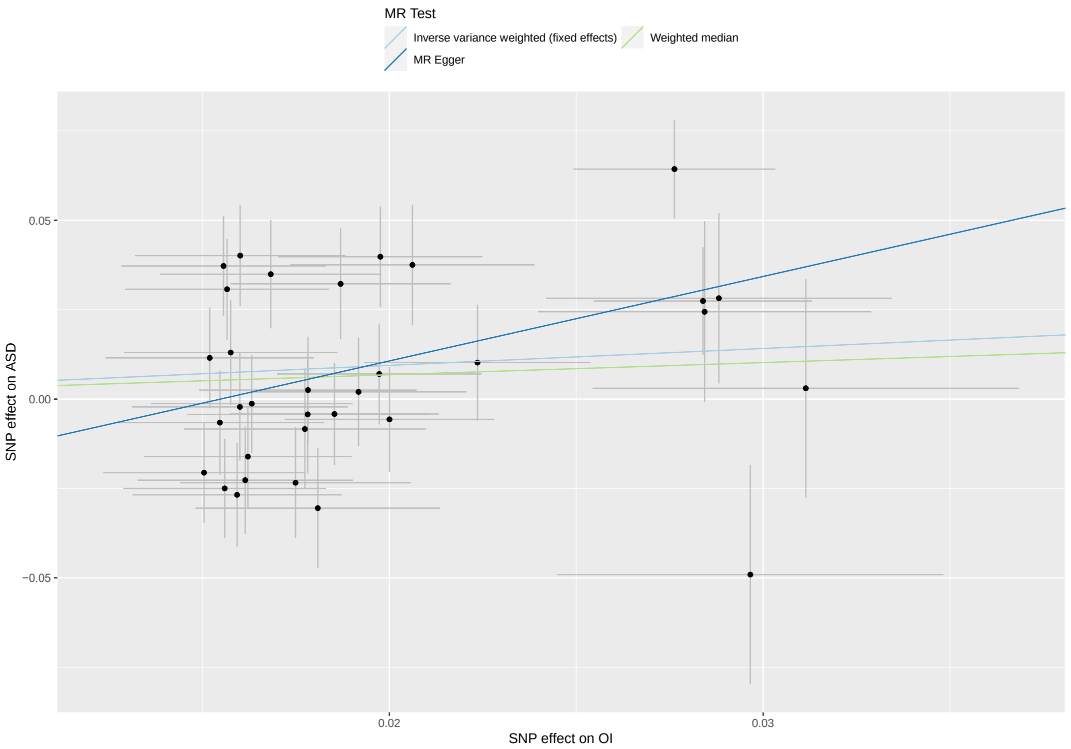

Supplementary Figure 59: scatterplot of occupational income against ASD
Abbreviations: MR: Mendelian randomization; SNP: single nucleotide polymorphism; OI: occupational income; ASD: autism spectrum disorders.
Legend: n=33 SNPs. Each dot represents a SNP, with the slope of each line corresponding to the estimated MR effect per method.

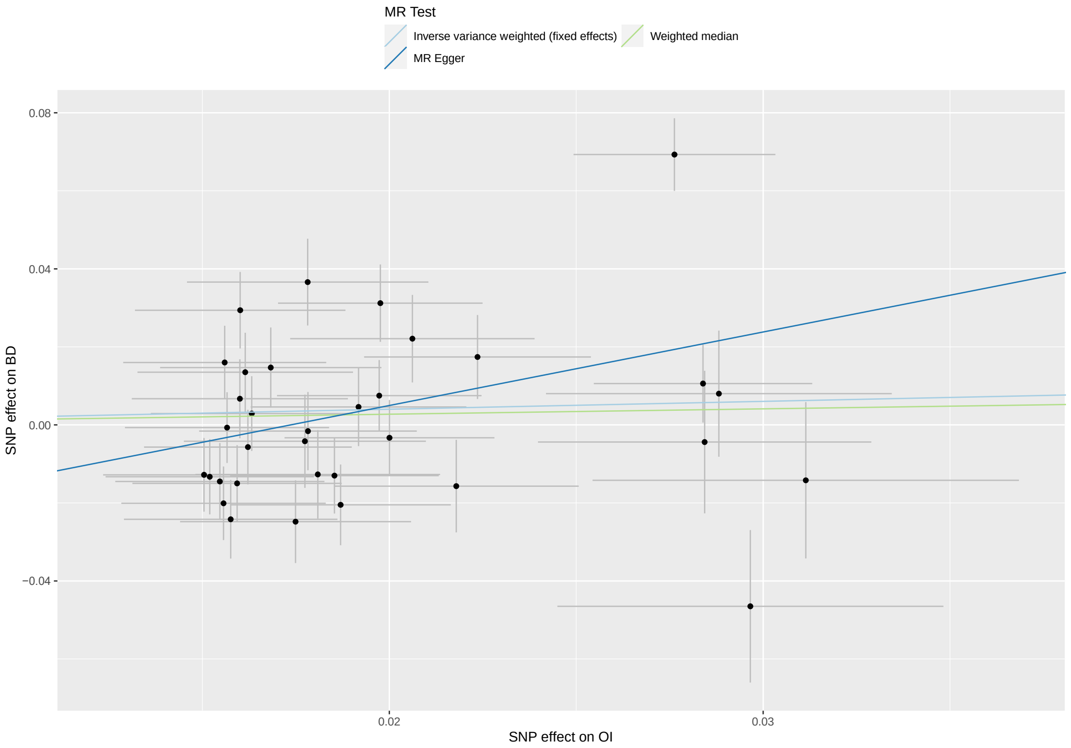

Supplementary Figure 60: scatterplot of occupational income against BD
Abbreviations: MR: Mendelian randomization; SNP: single nucleotide polymorphism; OI: occupational income; BD: bipolar disorder.

Legend: n=34 SNPs. Each dot represents a SNP, with the slope of each line corresponding to the estimated MR effect per method.

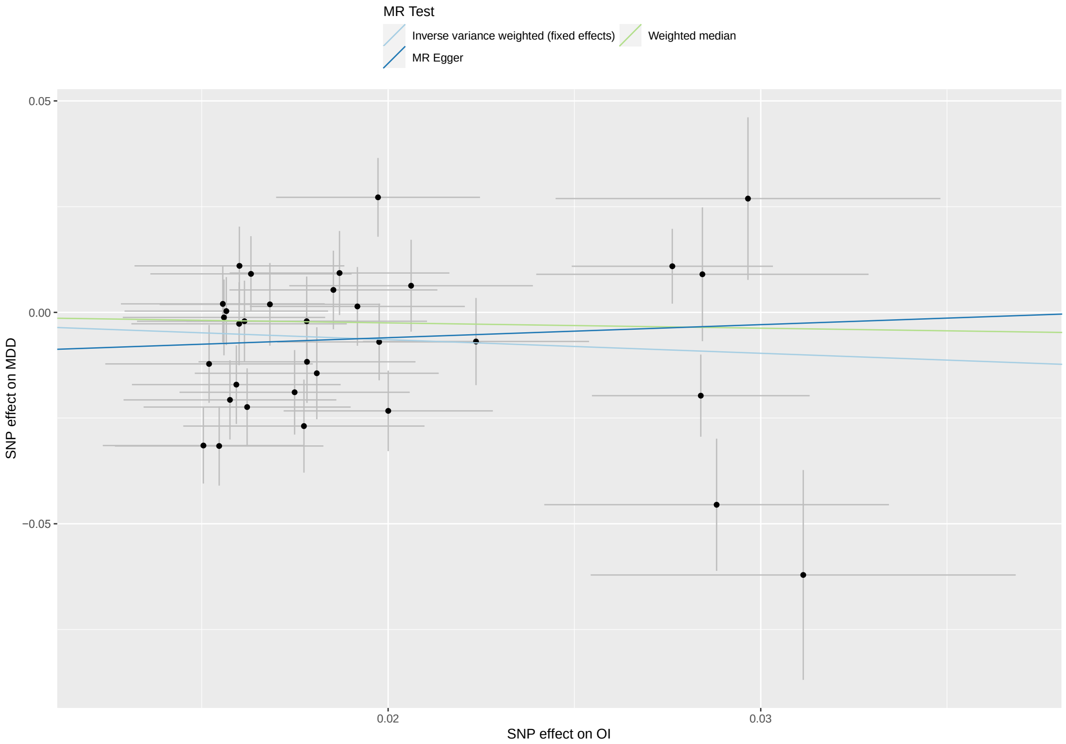

Supplementary Figure 61: scatterplot of occupational income against MDD
Abbreviations: MR: Mendelian randomization; SNP: single nucleotide polymorphism; OI: occupational income; MDD: major depressive disorder.
Legend: n=33 SNPs. Each dot represents a SNP, with the slope of each line corresponding to the estimated MR effect per method.

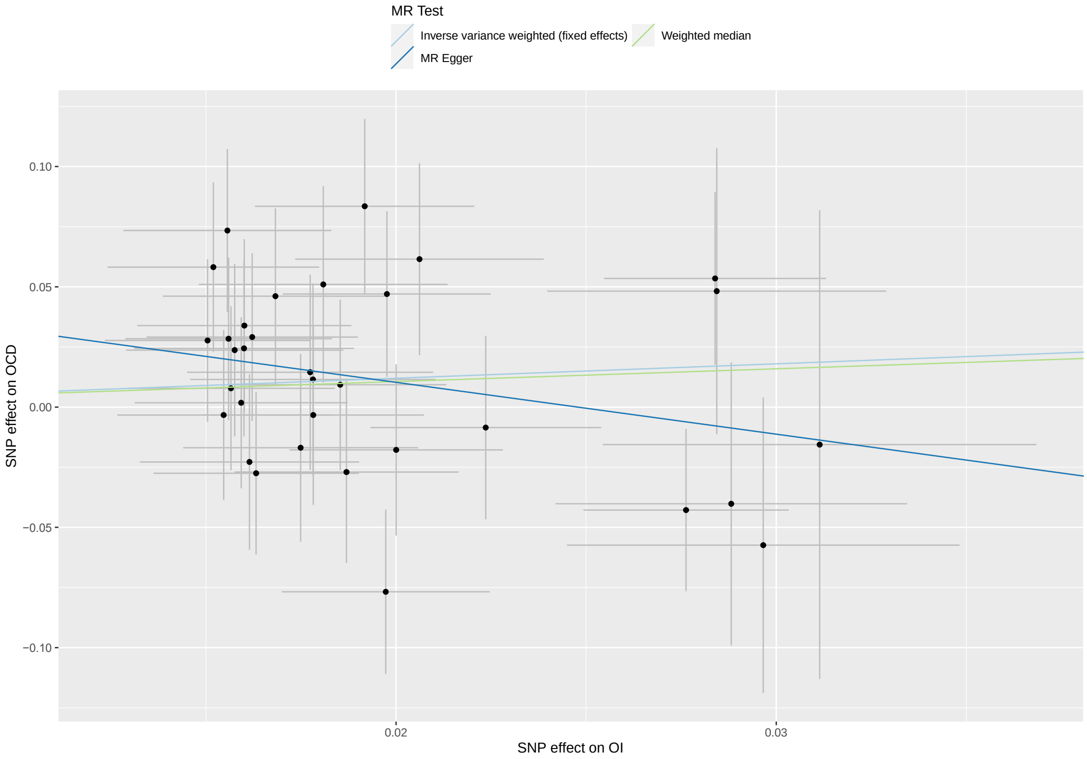

Supplementary Figure 62: scatterplot of occupational income against OCD
Abbreviations: MR: Mendelian randomization; SNP: single nucleotide polymorphism; OI: occupational income; OCD: obsessive-compulsive disorder.
Legend: n=33 SNPs. Each dot represents a SNP, with the slope of each line corresponding to the estimated MR effect per method.

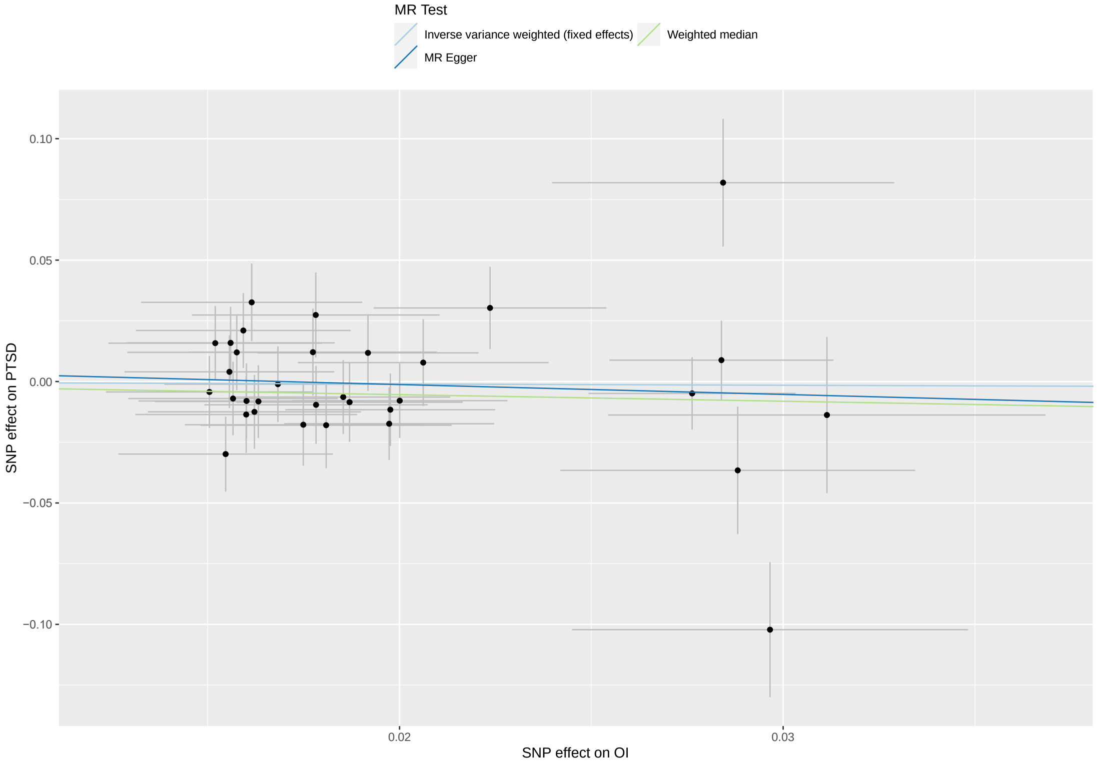

Supplementary Figure 63: scatterplot of occupational income against PTSD
Abbreviations: MR: Mendelian randomization; SNP: single nucleotide polymorphism; OI: occupational income; PTSD: post-traumatic stress disorder.
Legend: n=33 SNPs. Each dot represents a SNP, with the slope of each line corresponding to the estimated MR effect per method.

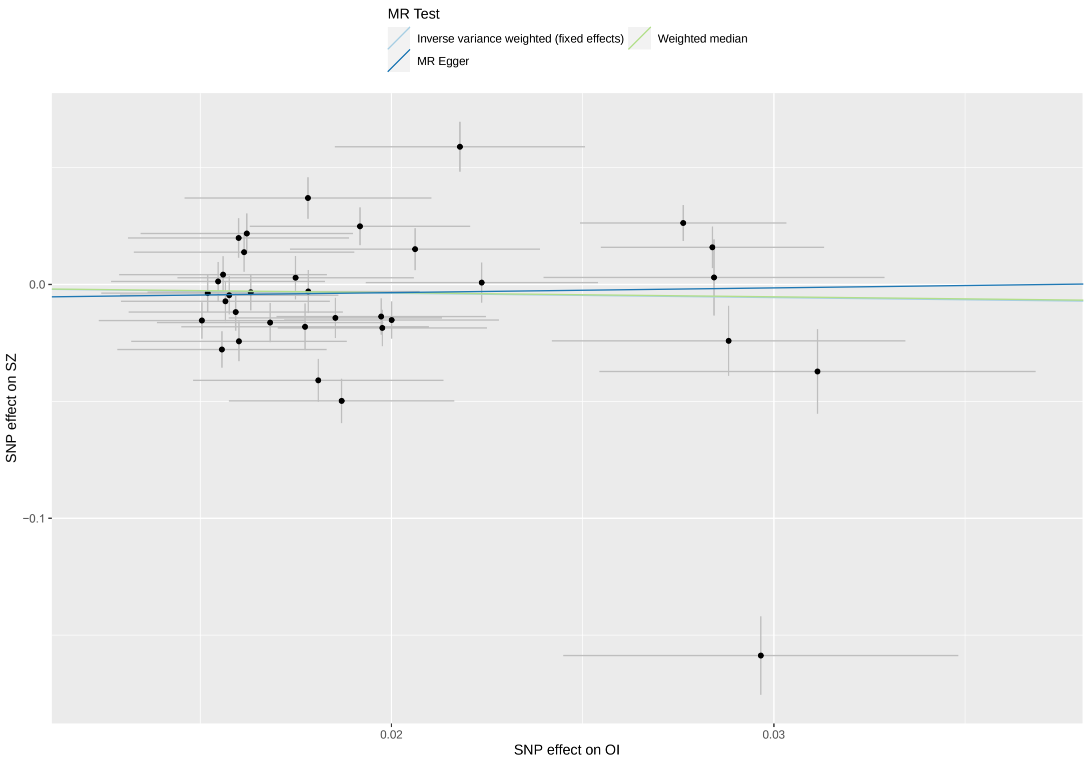

Supplementary Figure 64: scatterplot of occupational income against SZ
Abbreviations: MR: Mendelian randomization; SNP: single nucleotide polymorphism; OI: occupational income; SZ: schizophrenia.
Legend: n=34 SNPs. Each dot represents a SNP, with the slope of each line corresponding to the estimated MR effect per method.

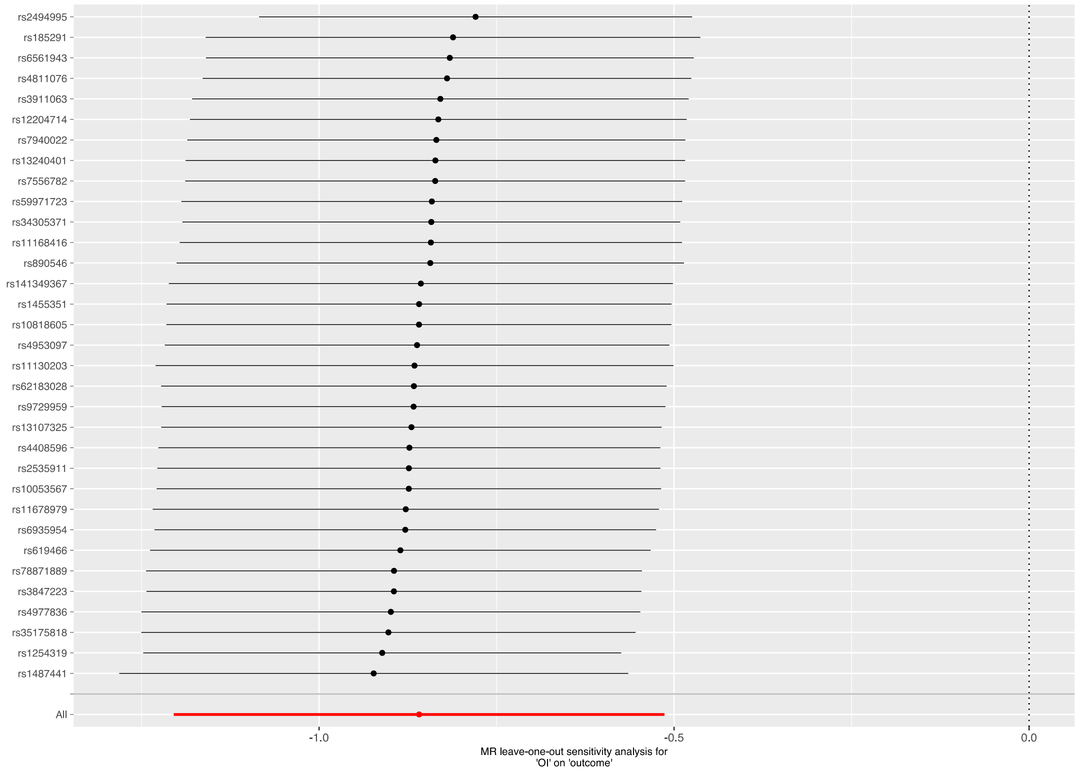

Supplementary Figure 65: leave-one-out analysis of occupational income against ADHD

Abbreviations: MR: Mendelian randomization; OI: occupational income; ADHD: attention deficit hyperactivity disorder.
Legend: n=33 SNPs. Forest plot of individual and combined SNP MR estimated effect size. The effect estimates represent the log-odds for ADHD per one standard deviation (1 SD) increase in the mean occupational income, and the error bars represent 95% CIs.

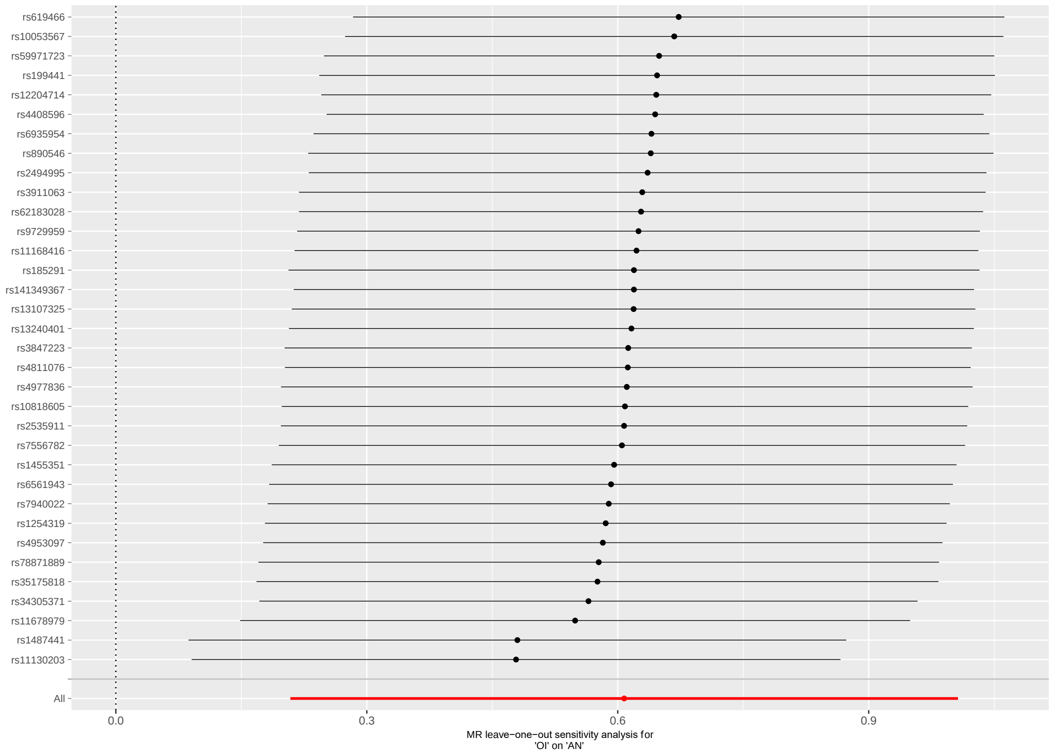

Supplementary Figure 66: leave-one-out analysis of occupational income against AN
Abbreviations: MR: Mendelian randomization; OI: occupational income; AN: anorexia nervosa.
Legend: n=34 SNPs. Forest plot of individual and combined SNP MR estimated effect size. The effect estimates represent the log-odds for AN per one standard deviation (1 SD) increase in the mean occupational income, and the error bars represent 95% CIs.

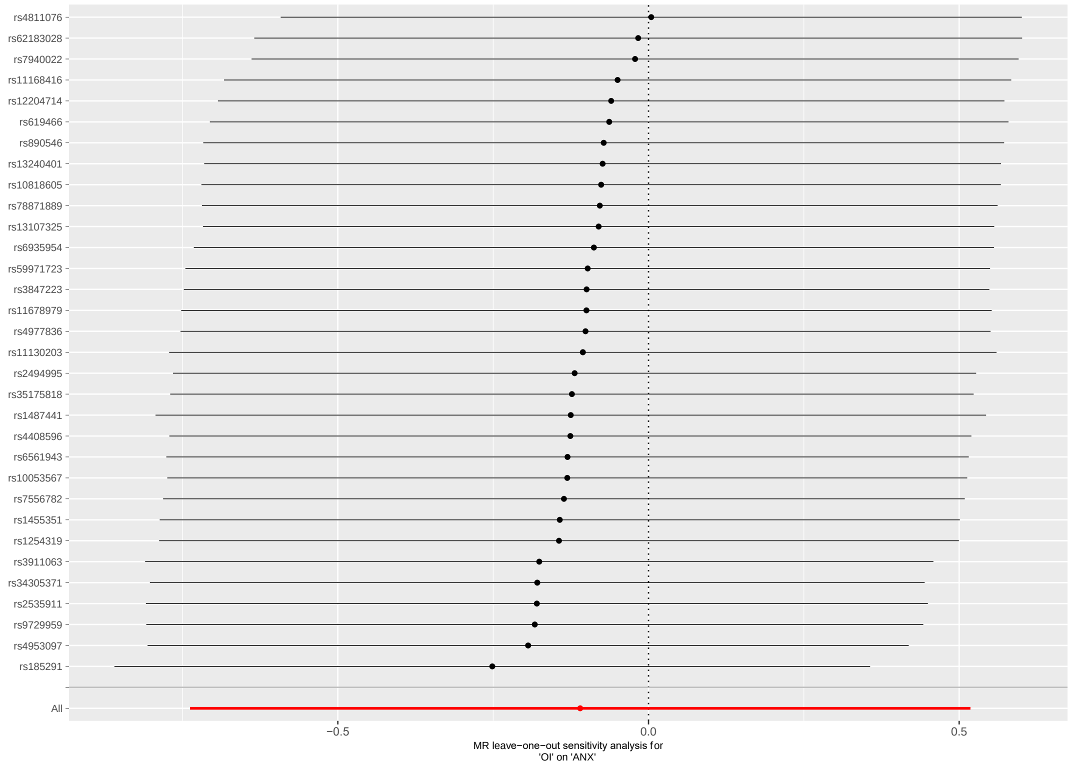

Supplementary Figure 67: leave-one-out analysis of occupational income against ANX
Abbreviations: MR: Mendelian randomization; OI: occupational income; ANX: anxiety disorders.
Legend: n=32 SNPs. Forest plot of individual and combined SNP MR estimated effect size. The effect estimates represent the log-odds for ANX per one standard deviation (1 SD) increase in the mean occupational income, and the error bars represent 95% CIs.

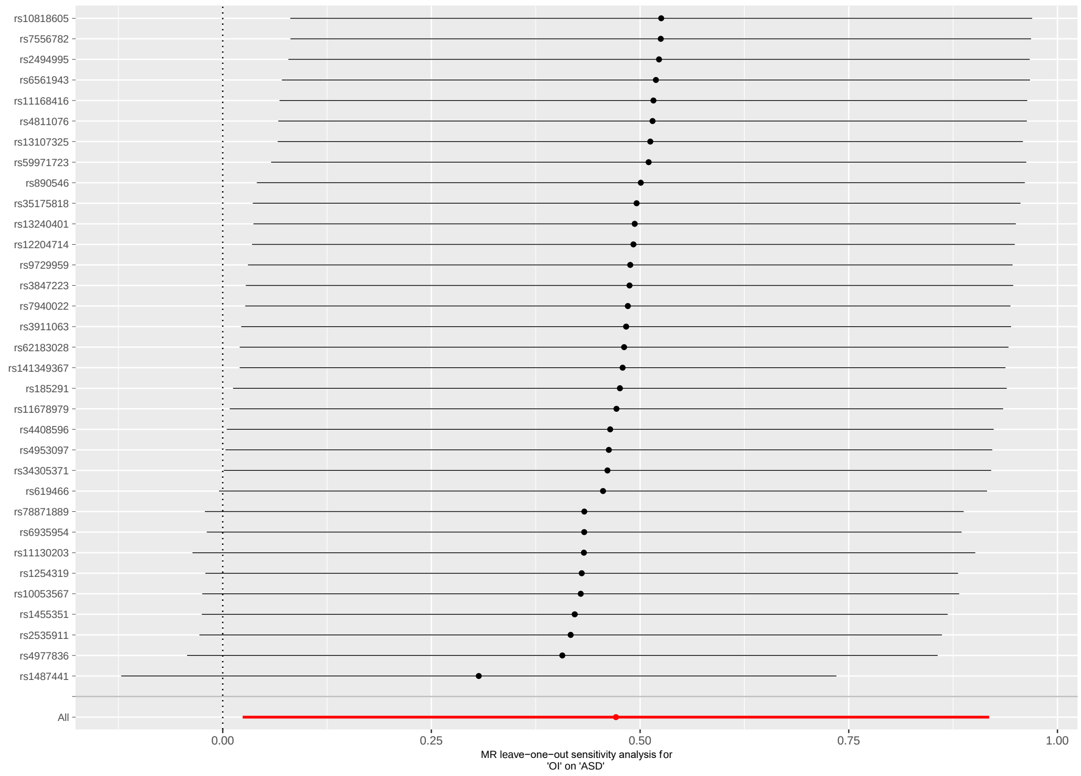

Supplementary Figure 68: leave-one-out analysis of occupational income against ASD

Abbreviations: MR: Mendelian randomization; OI: occupational income; ASD: autism spectrum disorders.
Legend: n=33 SNPs. Forest plot of individual and combined SNP MR estimated effect size. The effect estimates represent the log-odds for ASD per one standard deviation (1 SD) increase in the mean occupational income, and the error bars represent 95% CIs.

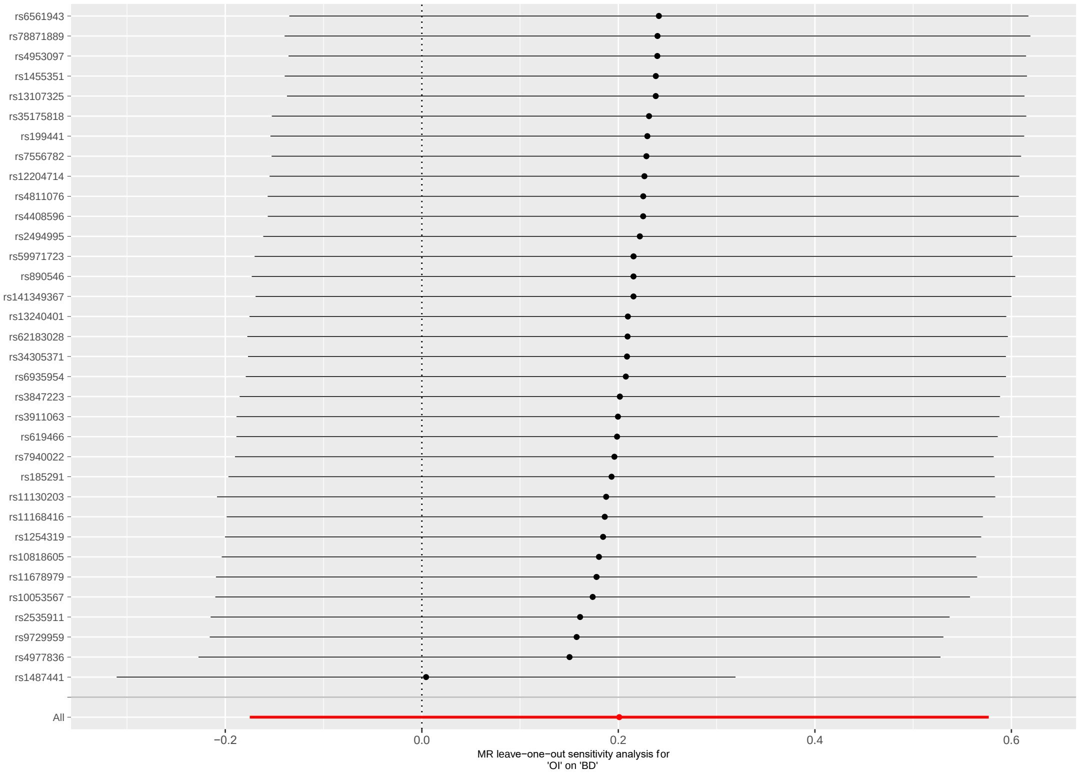

Supplementary Figure 69: leave-one-out analysis of occupational income against BD
Abbreviations: MR: Mendelian randomization; OI: occupational income; BD: bipolar disorder.
Legend: n=34 SNPs. Forest plot of individual and combined SNP MR estimated effect size. The effect estimates represent the log-odds for BD per one standard deviation (1 SD) increase in the mean occupational income, and the error bars represent 95% CIs.

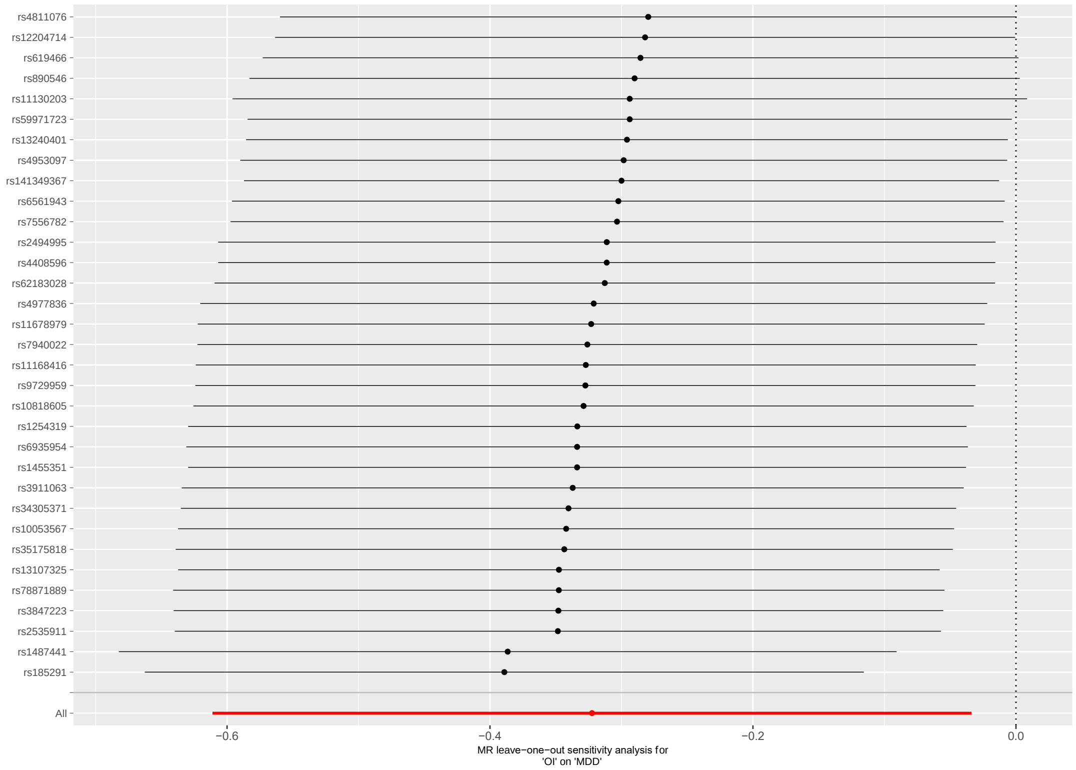

Supplementary Figure 70: leave-one-out analysis of occupational income against MDD
Abbreviations: MR: Mendelian randomization; OI: occupational income; MDD: major depressive disorder.
Legend: n=33 SNPs. Forest plot of individual and combined SNP MR estimated effect size. The effect estimates represent the log-odds for MDD per one standard deviation (1 SD) increase in the mean occupational income, and the error bars represent 95% CIs.

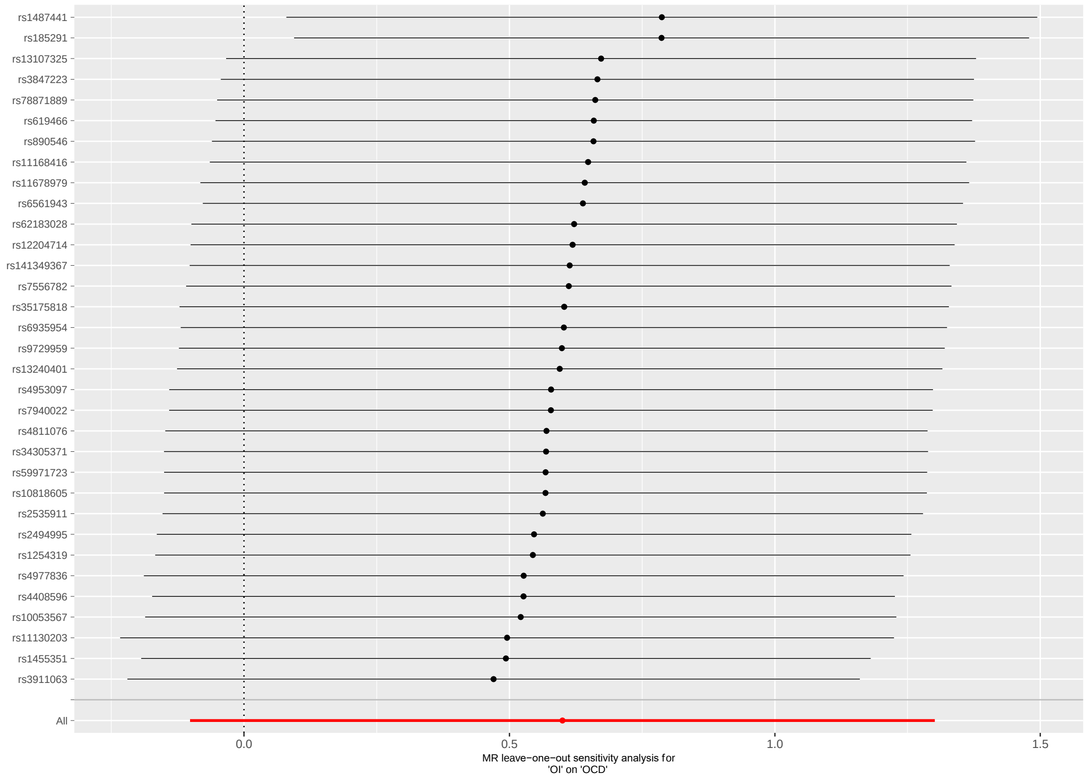

Supplementary Figure 71: leave-one-out analysis of occupational income against OCD

Abbreviations: MR: Mendelian randomization; OI: occupational income; OCD: obsessive-compulsive disorder.
Legend: n=33 SNPs. Forest plot of individual and combined SNP MR estimated effect size. The effect estimates represent the log-odds for OCD per one standard deviation (1 SD) increase in the mean occupational income, and the error bars represent 95% CIs.

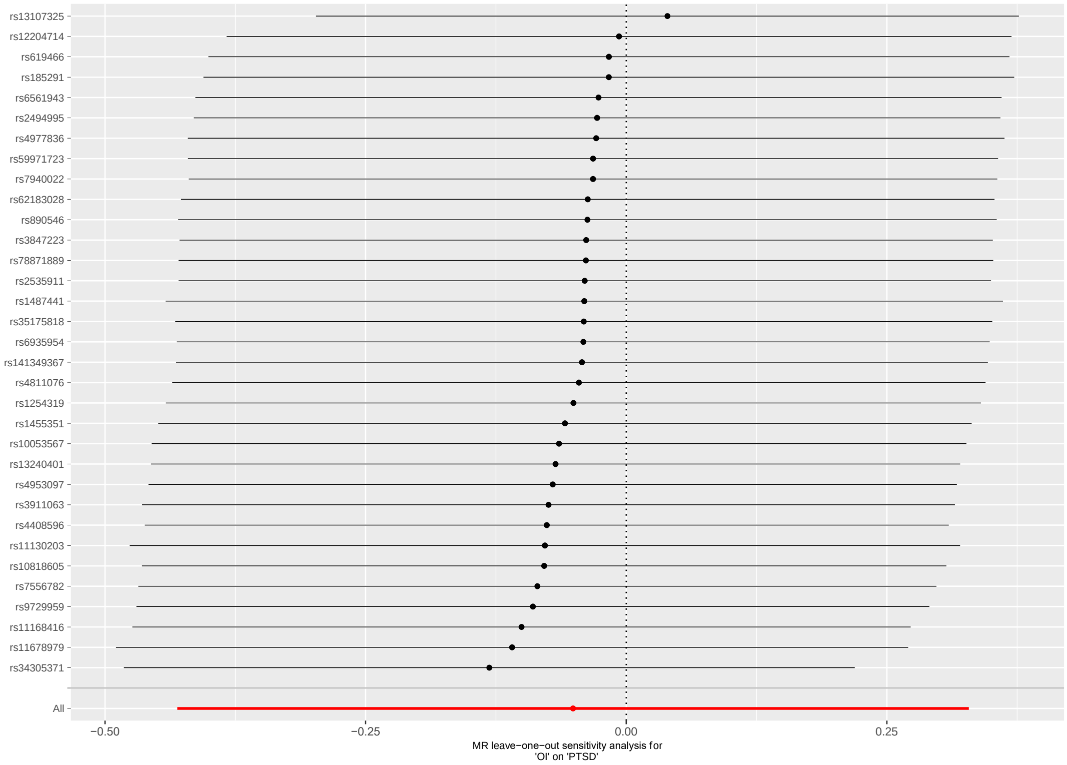

Supplementary Figure 72: leave-one-out analysis of occupational income against PTSD
Abbreviations: MR: Mendelian randomization; OI: occupational income; PTSD: post-traumatic stress disorder.
Legend: n=33 SNPs. Forest plot of individual and combined SNP MR estimated effect size. The effect estimates represent the log-odds for PTSD per one standard deviation (1 SD) increase in the mean occupational income, and the error bars represent 95% CIs.

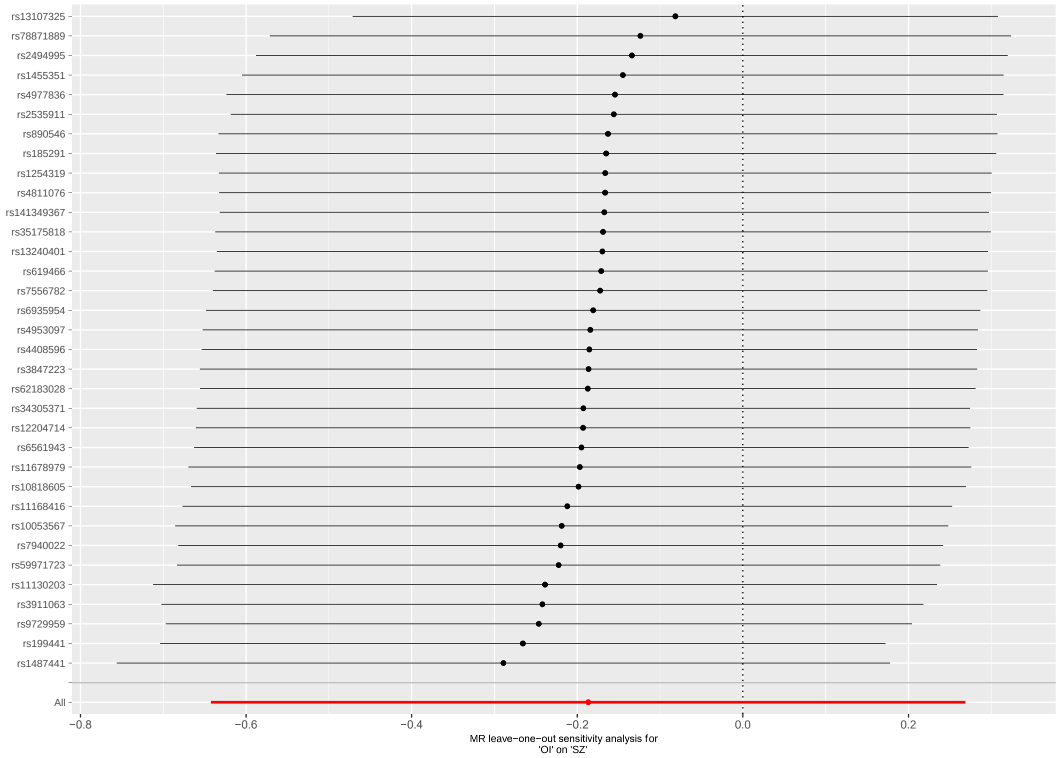

Supplementary Figure 73: leave-one-out analysis of occupational income against SZ
Abbreviations: MR: Mendelian randomization; OI: occupational income; SZ: schizophrenia.
Legend: n=34 SNPs. Forest plot of individual and combined SNP MR estimated effect size. The effect estimates represent the log-odds for SZ per one standard deviation (1 SD) increase in the mean occupational income, and the error bars represent 95% CIs.

Plots - Backward analyses

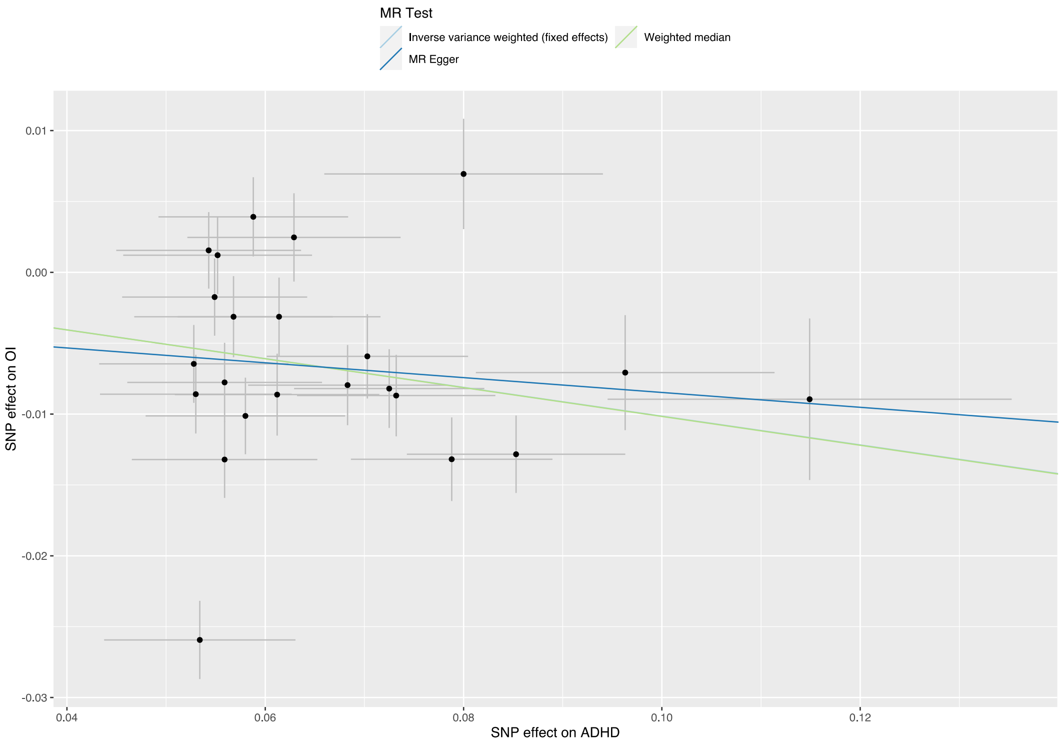

Supplementary Figure 74: scatterplot of ADHD against occupational income
Abbreviations: MR: Mendelian randomization; OI: occupational income; ADHD: attention deficit hyperactivity disorder.
Legend: n=23 SNPs. Each dot represents a SNP, with the slope of each line corresponding to the estimated MR effect per method.

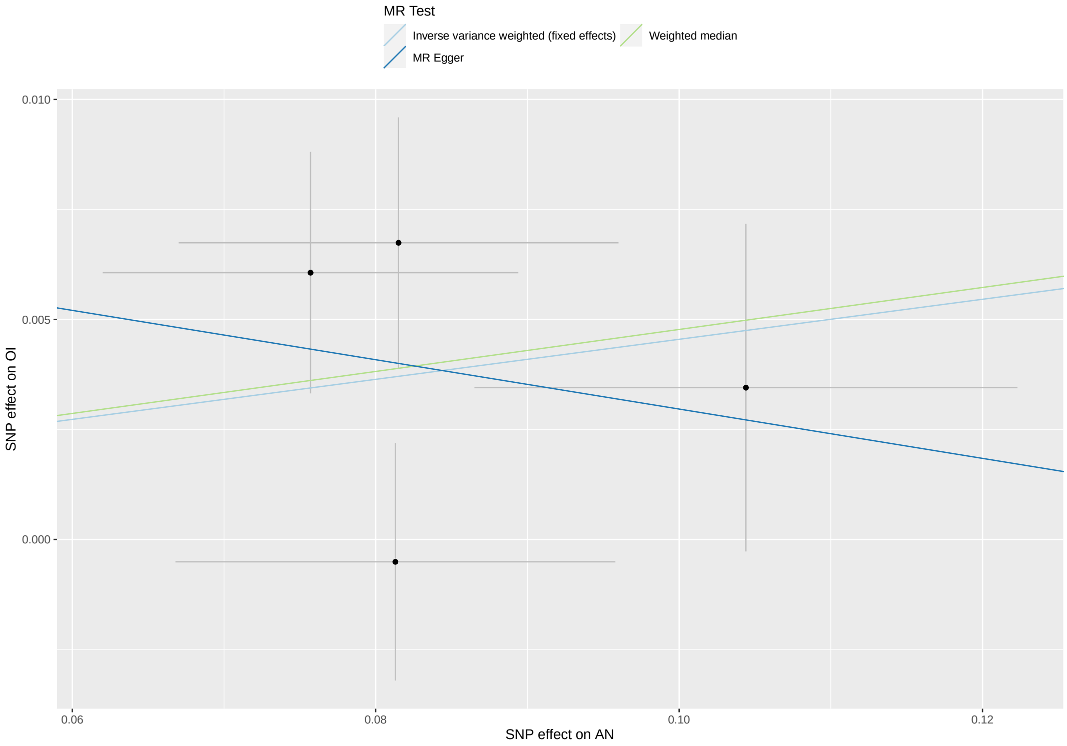

Supplementary Figure 75: scatterplot of AN against occupational income
Abbreviations: MR: Mendelian randomization; OI: occupational income; AN: anorexia nervosa.
Legend: n=4 SNPs. Each dot represents a SNP, with the slope of each line corresponding to the estimated MR effect per method.

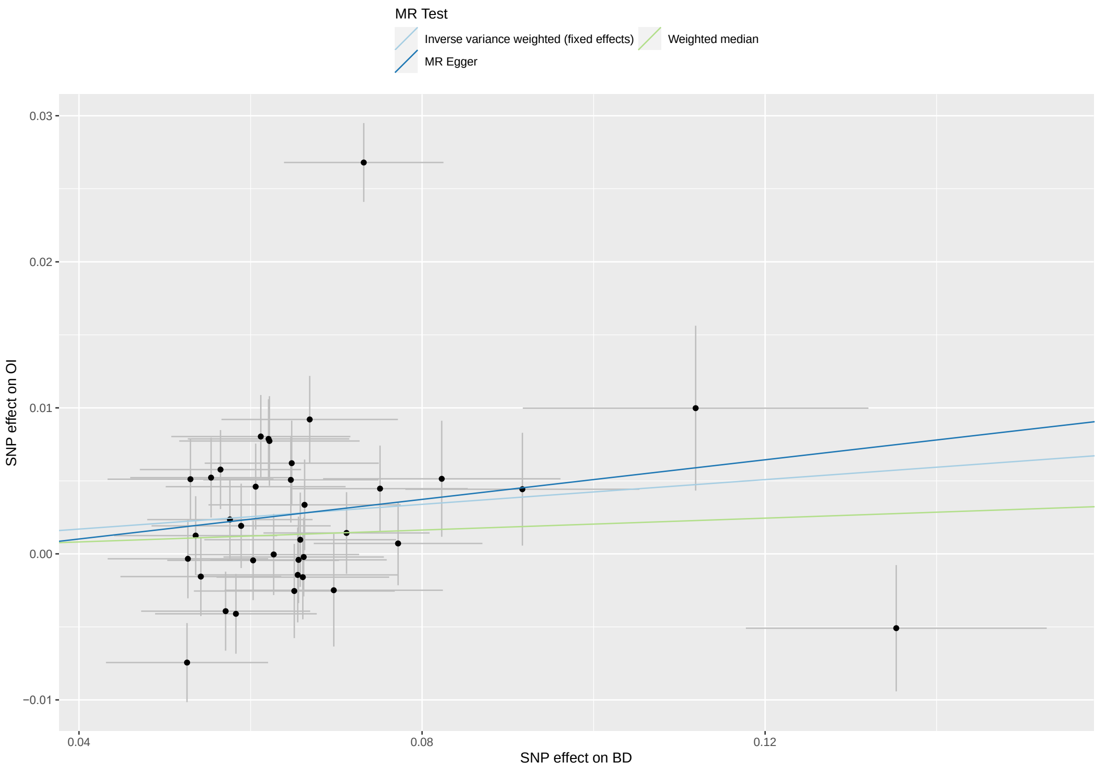

Supplementary Figure 76: scatterplot of BD against occupational income
Abbreviations: MR: Mendelian randomization; OI: occupational income; BD: bipolar disorder.
Legend: n=36 SNPs. Each dot represents a SNP, with the slope of each line corresponding to the estimated MR effect per method.

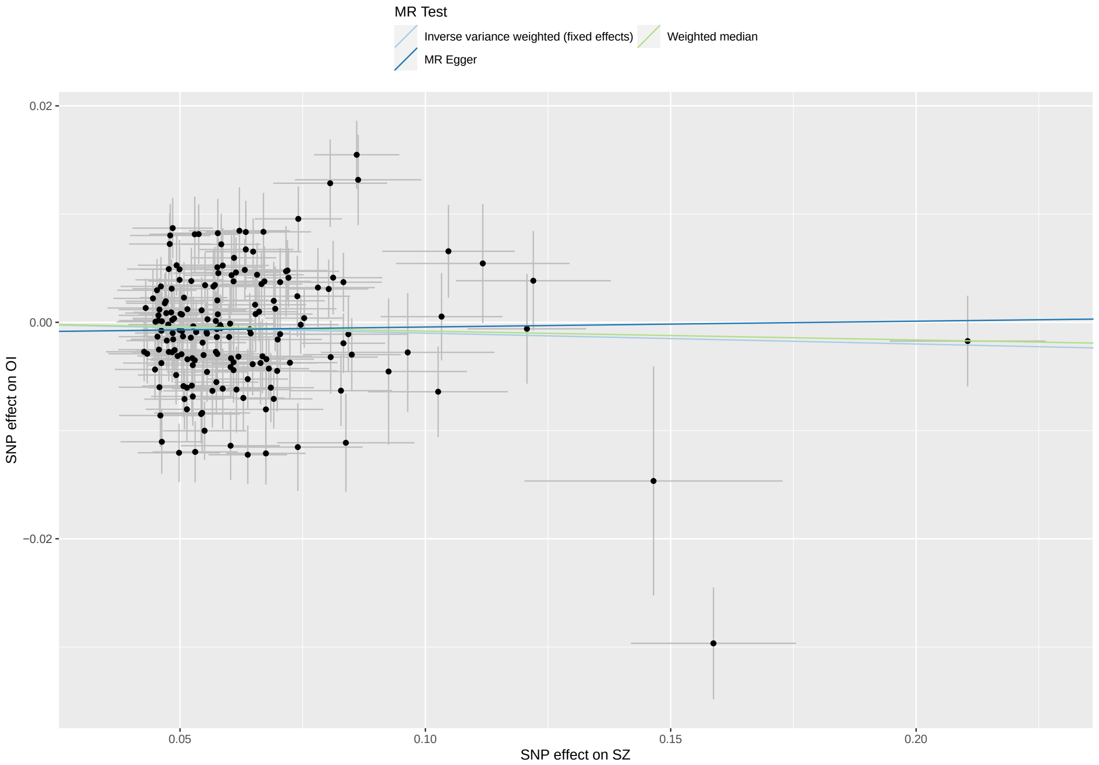

Supplementary Figure 77: scatterplot of SZ against occupational income
Abbreviations: MR: Mendelian randomization; OI: occupational income; SZ: schizophrenia.

Legend: n=176 SNPs. Each dot represents a SNP, with the slope of each line corresponding to the estimated MR effect per method.

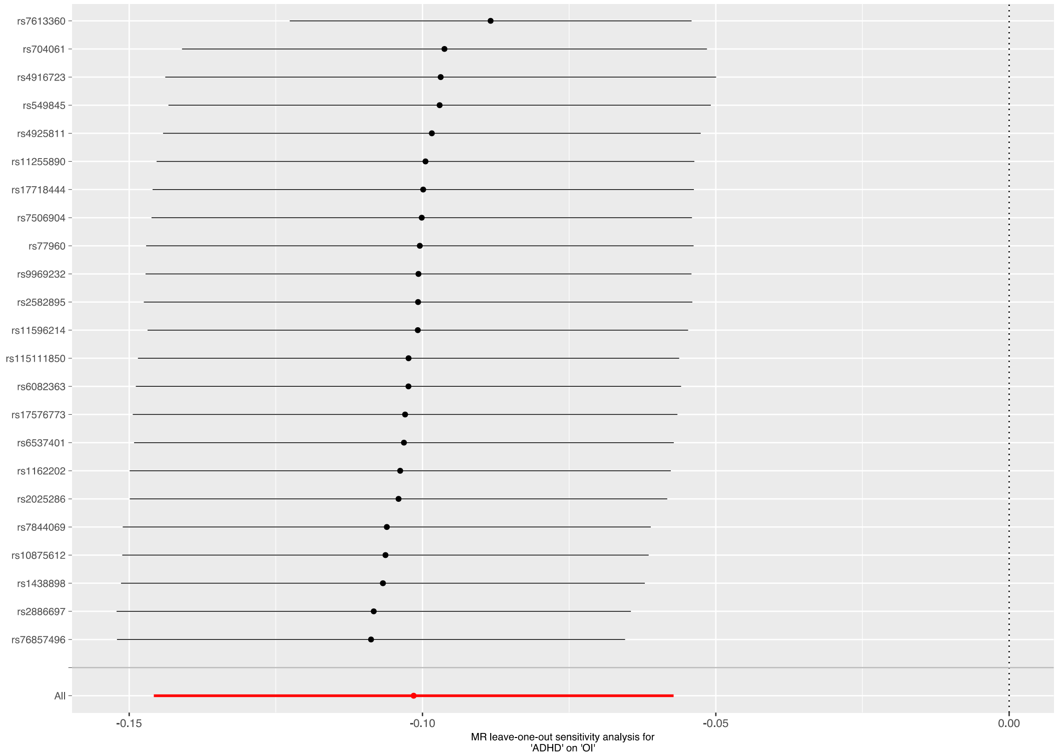

Supplementary Figure 78: leave-one-out analysis of ADHD against occupational income
Abbreviations: MR: Mendelian randomization; OI: occupational income; ADHD: attention deficit hyperactivity disorder.
Legend: n=23 SNPs. Forest plot of individual and combined SNP MR estimated effect size. The effect estimates represent the linear effect for the mean occupational income per ADHD status, and the error bars represent 95% CIs.

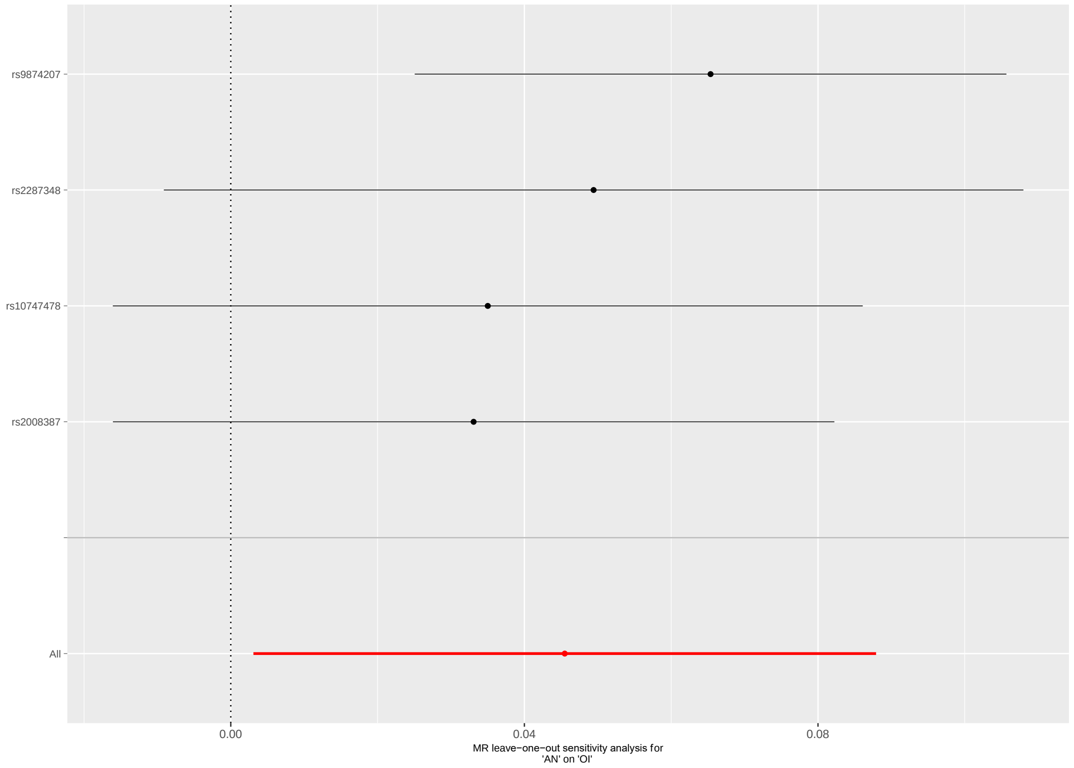

Supplementary Figure 79: leave-one-out analysis of AN against occupational income
Abbreviations: MR: Mendelian randomization; OI: occupational income; AN: anorexia nervosa.
Legend: n=4 SNPs. Forest plot of individual and combined SNP MR estimated effect size. The effect estimates represent the linear effect for the mean occupational income per AN status, and the error bars represent 95% CIs.

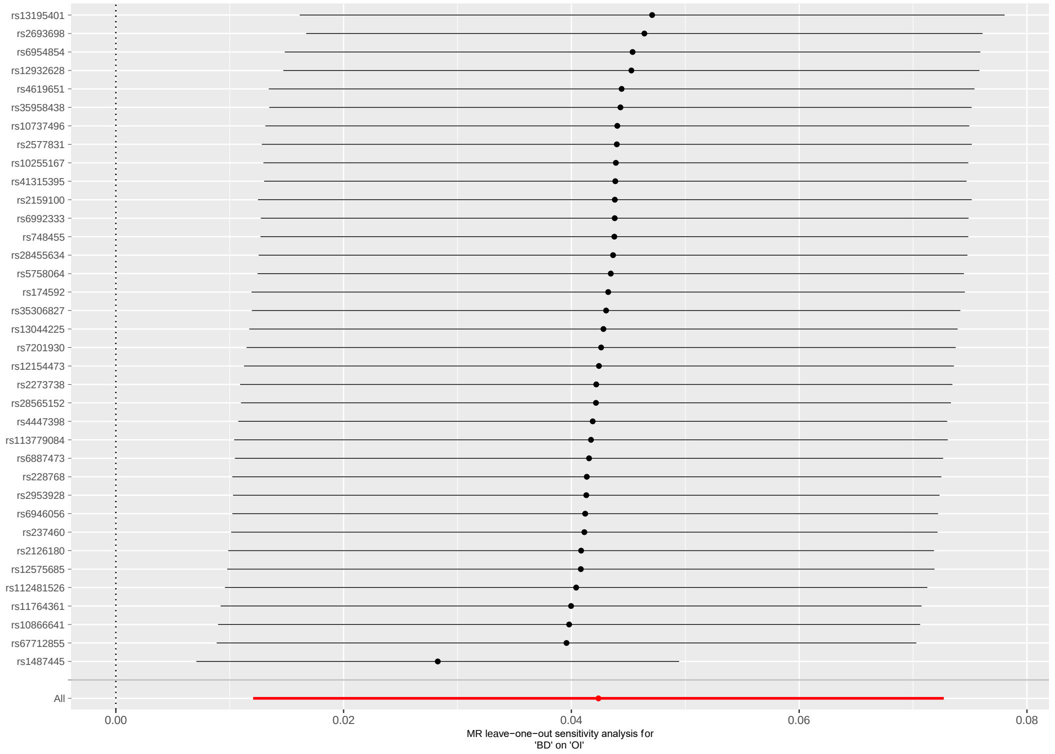

Supplementary Figure 80: leave-one-out analysis of BD against occupational income

Abbreviations: MR: Mendelian randomization; OI: occupational income; BD: bipolar disorder.
Legend: n=36 SNPs. Forest plot of individual and combined SNP MR estimated effect size. The effect estimates represent the linear effect for the mean occupational income per BD status, and the error bars represent 95% CIs.

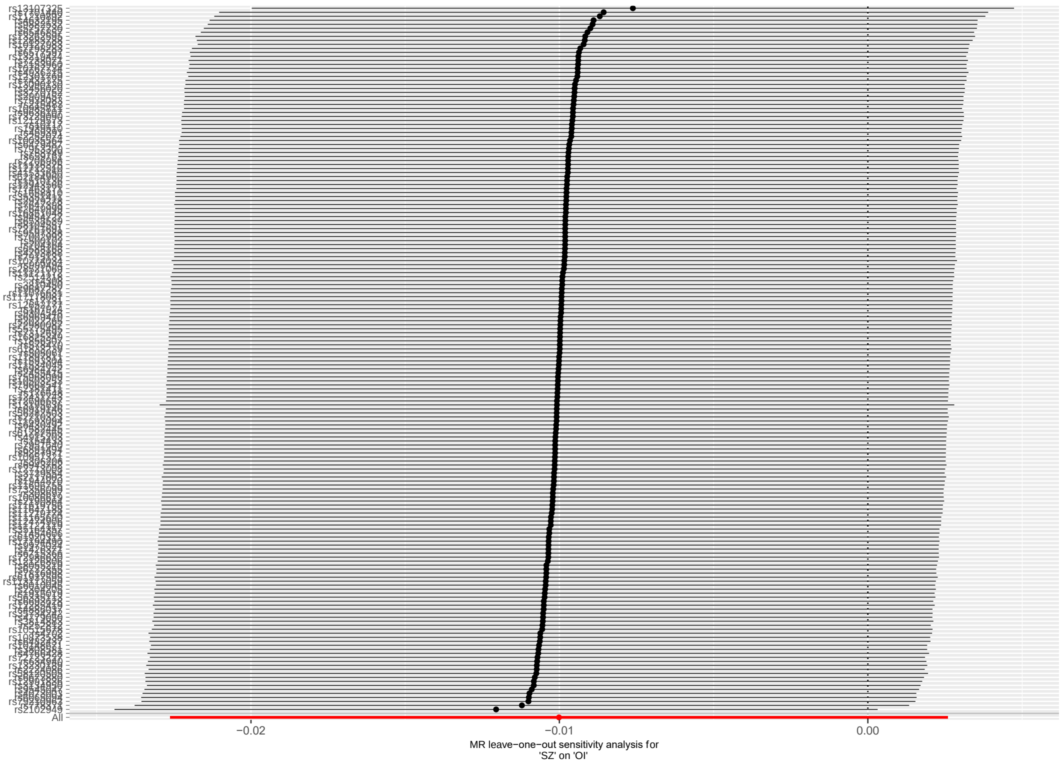

Supplementary Figure 81: leave-one-out analysis of SZ against occupational income
Abbreviations: MR: Mendelian randomization; OI: occupational income; SZ: schizophrenia.
Legend: n=176 SNPs. Forest plot of individual and combined SNP MR estimated effect size. The effect estimates represent the linear effect for the mean occupational income per SZ status, and the error bars represent 95% CIs.

Supplementary Table 15: CAUSE results of the relations between occupational income and mental illness

**Model 1**	**Model 2**	**Δ ELPD**	**SE Δ ELPD**	**z-score**	**p-value†**
		Fw: OI on ADHD			
Null	Sharing	-15.39	4.06	-3.79	**1.51×10^{-4}**
Null	Causal	-21.30	5.72	-3.72	**1.99×10^{-4}**
Sharing	Causal	-5.91	1.72	-3.44	**5.82×10^{-4}**
		Bw: ADHD on OI			
Null	Sharing	-11.42	3.31	-3.45	**5.61×10^{-4}**
Null	Causal	-17.32	5.04	-3.44	**5.82×10^{-4}**
Sharing	Causal	-5.91	1.76	-3.36	**7.79×10^{-4}**
		Fw: OI on AN			
Null	Sharing	-7.83	3.41	-2.30	**0.021**
Null	Causal	-10.22	4.62	-2.21	**0.027**
Sharing	Causal	-2.39	1.47	-1.62	0.105
		Bw: AN on OI			

Null	Sharing	0.29	0.24	1.24	0.215
Null	Causal	0.17	1.26	0.14	0.889
Sharing	Causal	-0.12	1.04	-0.12	0.904
Fw: OI on ANX					
Null	Sharing	-2.19	2.13	-1.03	0.303
Null	Causal	-2.86	2.97	-0.96	0.337
Sharing	Causal	-0.66	1.26	-0.53	0.596
Bw: ANX on OI					
Null	Sharing	0.09	0.10	0.86	0.390
Null	Causal	0.82	0.42	1.96	0.050
Sharing	Causal	0.73	0.34	2.16	**0.031**
Fw: OI on ASD					
Null	Sharing	0.38	0.21	1.83	0.067
Null	Causal	0.63	1.07	0.59	0.555
Sharing	Causal	0.24	0.87	0.28	0.779
Bw: ASD on OI					
Null	Sharing	0.34	0.14	2.39	**0.017**
Null	Causal	0.58	0.90	0.65	0.516
Sharing	Causal	0.24	0.77	0.32	0.749
Fw: OI on BD					
Null	Sharing	0.46	0.07	6.39	**1.66×10^{-10}**
Null	Causal	1.38	0.09	16.01	**1.09×10^{-57}**
Sharing	Causal	0.91	0.02	36.68	**1.52×10^{-294}**
Bw: BD on OI					
Null	Sharing	0.08	0.59	0.13	0.447
Null	Causal	-0.13	1.69	-0.08	0.469
Sharing	Causal	-0.21	1.13	-0.19	0.427
Fw: OI on MDD					
Null	Sharing	-0.69	0.95	-0.73	0.465
Null	Causal	-2.62	2.63	-1.00	0.317
Sharing	Causal	-1.93	1.71	-1.13	0.258
Bw: MDD on OI					
Null	Sharing	0.24	0.21	1.14	0.254
Null	Causal	0.06	1.25	0.05	0.960
Sharing	Causal	-0.18	1.04	-0.17	0.865
Fw: OI on OCD					
Null	Sharing	0.39	0.24	1.61	0.107
Null	Causal	0.72	1.07	0.67	0.503
Sharing	Causal	0.33	0.84	0.39	0.697
Bw: OCD on OI					
Null	Sharing	0.24	0.05	4.39	**1.13×10^{-5}**
Null	Causal	0.80	0.41	1.92	0.055
Sharing	Causal	0.56	0.37	1.52	0.129
Fw: OI on PTSD					
Null	Sharing	-1.71	1.63	-1.05	0.294
Null	Causal	-3.60	3.04	-1.18	0.238
Sharing	Causal	-1.89	1.56	-1.21	0.226
Bw: PTSD on OI					
Null	Sharing	0.27	0.03	8.54	**1.34×10^{-17}**

Null	Causal	1.05	0.13	7.92	**2.38×10^{-15}**
Sharing	Causal	0.78	0.12	6.75	**1.48×10^{-11}**
Fw: OI on SZ					
Null	Sharing	0.467	0.03	16.1	**2.55×10^{-58}**
Null	Causal	1.32	0.05	25.5	**1.97×10^{-143}**
Sharing	Causal	0.849	0.04	19.4	**7.72×10^{-84}**
Bw: SZ on OI					
Null	Sharing	-0.88	2.61	-0.34	0.734
Null	Causal	0.03	2.38	0.01	0.992
Sharing	Causal	0.92	0.55	1.68	0.093

Abbreviations: MR: mendelian randomization; ELPD: expected log pointwise posterior density; 95%CI: 95% confidence interval; SE: standard error; Fwd: forward MR; Bwd: backward MR; OI: occupational income; ADHD: attention deficit hyperactivity disorder; AN: anorexia nervosa; ANX: anxiety disorder; ASD: autism spectrum disorder; BD: bipolar disorder; MDD: major depressive disorder; OCD: obsessive-compulsive disorder; PTSD: post-traumatic stress disorder; SZ: schizophrenia.
Legend: † Based on t-test. The p-values are two-sided and not adjusted for multiple testing, therefore p-value<0.05 was considered significant (and reported in bolded text).

Supplementary Table 16: Results of univariable bidirectional Mendelian Randomization of occupational income against mental illnesses, after Steiger filtering

MR	N SNP	IVW, B (95% CI)	IVW p-value	WM, B (95% CI)	WM p-value	MR-Egger, B (95% CI)	MR-Egger p-value	Egger intercept p-value	Mean F
OI on ADHD	32	-0.780 (-0.972; -0.587)	**1.91×10^{-15}**	-0.556 (-0.890; 0.222)	**0.001**	0.094 (-1.36; 1.55)	0.900	**0.033**	40.6
OI on AN	34	0.608 (0.337; 0.879)	**1.10×10^{-5}**	0.437 (-0.007; 0.882)	0.054	2.51 (0.635; 4.39)	**0.013**	**0.001**	40.4
OI on ANX	31	0.004 (-0.566; 0.575)	0.988	-0.102 (-0.961; 0.757)	0.816	0.164 (-2.83; 3.16)	0.915	0.713	40.9
OI on ASD	29	0.125 (-0.179; 0.428)	0.421	0.104 (-0.363; 0.571)	0.664	2.04 (-0.054; 4.03)	0.054	**0.010**	38.9
OI on BD	29	-0.134 (-0.338; 0.071)	0.201	-0.091 (-0.397; 0.215)	0.561	0.445 (-0.905; 1.79)	0.524	**0.024**	38.7
OI on MDD	33	-0.322 (-0.502; -0.143)	**4.37×10^{-4}**	-0.125 (-0.420; 0.171)	0.408	0.309 (-1.10; 1.72)	0.671	0.376	40.3
OI on OCD	25	0.107 (-0.672; 0.885)	0.788	-0.075 (-1.18; 1.03)	0.894	-1.37 (-4.75; 2.02)	0.437	0.590	40.6
OI on PTSD	33	-0.051 (-0.347; 0.245)	0.736	-0.271 (-0.717; 0.176)	0.235	-0.407 (-2.26; 1.44)	0.669	0.702	40.3
OI on SZ	33	0.082 (-0.240; 0.077)	0.313	-0.172 (-0.497; 0.153)	0.299	1.24 (-0.700; 3.19)	0.219	**0.001**	40.6

Abbreviations: OI: occupational income; ADHD: attention deficit hyperactivity disorder; AN: anorexia nervosa; ANX: anxiety disorder; ASD: autism spectrum disorders; BD: bipolar disorder; MDD: major depressive disorder; OCD: obsessive-compulsive disorder; PTSD: post-traumatic stress disorder; SZ: schizophrenia; MR: mendelian randomization; SNP: single nucleotide polymorphism; IVW: inverse variance weighted (fixed effect); B: effect estimates are log-odds; 95% CI: 95% confidence interval; WM: weighted median; NR: not reported because not enough SNP to perform MR.

Legend: All statistical tests were two-sided. The p-values were not adjusted for multiple testing, therefore p-value<0.05 was considered significant (and reported in bolded text).

Univariable Mendelian randomization of social deprivation and mental illnesses

Supplementary Table 17: Odds Ratio of univariable forward Mendelian randomization analysis of social deprivation against mental illnesses

MR: method	OR (95% CI)	p-value
SD → ADHD:		
IVW	2.04 (1.66; 2.51)	**2.20×10^{-11}**
WM	1.88 (1.35; 2.61)	**2.12×10^{-4}**
MR-Egger	0.855 (0.039; 18.9)	0.925
SD→ AN:		
IVW	0.703 (0.539; 0.917)	**0.009**
WM	0.746 (0.504; 1.11)	0.144
MR-Egger	1.35 (0.055; 33.4)	0.858
SD → ANX:		
IVW	1.18 (0.671; 2.07)	0.566
WM	1.30 (0.615; 2.74)	0.494
MR-Egger	2.47 (0.027; 229.9)	0.708
SD → ASD:		
IVW	0.990 (0.756; 1.29)	0.939
WM	1.24 (0.829; 1.86)	0.292
MR-Egger	0.627 (0.013; 30.5)	0.821
SD → BD:		
IVW	0.949 (0.777; 1.16)	0.607
WM	0.877 (0.656; 1.17)	0.375
MR-Egger	2.78 (0.048; 162.5)	0.639
SD → MDD:		
IVW	1.16 (0.955; 1.39)	0.138
WM	1.22 (0.939; 1.59)	0.137
MR-Egger	1.45 (0.166; 12.6)	0.749
SD → OCD:		
IVW	1.12 (0.553; 2.25)	0.761
WM	1.22 (0.451; 3.31)	0.692
MR-Egger	0.306 (0.001; 549.4)	0.767
SD → PTSD:		
IVW	1.18 (0.881; 1.58)	0.268
WM	1.21 (0.811; 1.80)	0.351
MR-Egger	3.96 (0.255; 61.4)	0.358
SD → SZ:		
IVW	1.24 (1.05; 1.46)	**0.012**
WM	1.37 (1.02; 1.88)	**0.040**
MR-Egger	7.61 (0.371; 156.3)	0.236

Abbreviations: MR: Mendelian randomization; OR: Odds Ratio; 95% CI: 95% confidence intervals; SD: social deprivation; ADHD: attention deficit hyperactivity disorder; AN: anorexia nervosa; ANX: anxiety disorder; ASD: autism spectrum disorders; BD: bipolar disorder; MDD: major depressive disorder; OCD: obsessive-compulsive disorder; PTSD: post-traumatic stress disorder; SZ: schizophrenia; IVW: inverse variance weighted (fixed effect); WM: weighted median; NR: not reported because not enough SNP to perform MR. Legend: All statistical tests were two-sided. The p-values were not adjusted for multiple testing, therefore p-value<0.05 was considered significant (and reported in bolded text).

Supplementary Table 18: results of bidirectional MR of Social Deprivation (SD) against mental health traits

**MR**	**N SNP**	**IVW, B (95% CI)**	**IVW p-value**	**IVW Q(df)**	**Q p-value**[†]	**WM, B (95% CI)**	**WM p-value**	**MR-Egger, B (95% CI)**	**MR-Egger p-value**	**Egger intercept p-value**	**Steiger Test p-value**[‡]	**MR-PRESSO**	**Mean F**
Fw: SD on ADHD	7	0.713 (0.504; 0.922)	**2.20×10^{-11}**	17 (6)	**0.001**	0.629 (0.298; 0.960)	**2.12×10^{-4}**	-0.157 (-3.25; 2.94)	0.925	0.603	0.079	DT; p=0.083	32.1
Bw: ADHD on SD	23	0.222 (0.181; 0.262)	**2.93×10^{-27}**	41 (22)	**0.009**	0.193 (0.129; 0.257)	**5.67×10^{-9}**	0.259 (-0.061; 0.579)	0.128	0.819	**5.95×10^{-78}**	DT; p=0.464	39.2
Fw: SD on AN	10	-0.352 (-0.618; -0.087)	**0.009**	21 (9)	**0.014**	-0.293 (-0.658; 0.072)	0.115	0.302 (-2.90; 3.51)	0.858	0.697	0.170	DT; p=0.100	32.8
Bw: AN on SD	4	-0.038 (-0.113; 0.036)	0.314	4 (3)	0.303	-0.052 (-0.146; 0.042)	0.277	-0.403 (-1.15; 0.342)	0.400	0.436	NR [b]	GT; p=0.352	31.9
Fw: SD on ANX	9	0.165 (-0.399; 0.730)	0.566	5 (8)	0.731	0.261 (-0.471; 0.992)	0.485	0.904 (-3.63; 5.44)	0.708	0.757	NR [b]	GT; p=0.709	33.0
Bw: ANX on SD	0	NR [c]	NR [c]	NR [c]	NR [c]	NR [c]	NR [c]	NR [c]	NR [c]	NR [c]	NR [c]	NR [c]	NR [c]
Fw: SD on ASD	9	-0.010 (-0.279; 0.258)	0.939	21 (8)	**0.009**	0.218 (-0.200; 0.635)	0.308	-0.466 (-4.35; 3.42)	0.821	0.823	NR [b]	DT; p=0.290	33.1
Bw: ASD on SD	0	NR [c]	NR [c]	NR [c]	NR [c]	NR [c]	NR [c]	NR [c]	NR [c]	NR [c]	NR [c]	NR [c]	NR [c]
Fw: SD on BD	8	-0.052 (-0.252; 0.148)	0.607	67 (7)	**5.18×10^{-12}**	-0.131 (-0.427; 0.164)	0.384	1.02 (-3.04; 5.09)	0.639	0.618	NR [b]	DT; p=0.756	31.8
Bw: BD on SD	36	-0.019 (-0.051; 0.013)	0.244	105 (35)	**6.99×10^{-9}**	-0.004 (-0.060; 0.052)	0.884	0.023 (-0.284; 0.330)	0.885	0.787	NR [b]	DT; p=0.096	39.2
Fw: SD on MDD	8	0.141 (-0.046; 0.328)	0.138	12 (7)	0.103	1.00 (-0.075; 0.475)	0.155	0.370 (-1.80; 2.54)	0.749	0.842	NR [b]	GT; p=0.125	33.4
Bw: MDD on SD	0	NR [c]	NR [c]	NR [c]	NR [c]	NR [c]	NR [c]	NR [c]	NR [c]	NR [c]	NR [c]	NR [c]	NR [c]

Fw: SD on OCD	8	0.109 (-0.593; 0.811)	0.761	10 (7)	0.177	0.201 (-0.773; 1.18)	0.685	-1.19 (-8.68; 6.31)	0.767	0.744	NR [b]	GT; p=0.206	33.4
Bw: OCD on SD	0	NR [c]	NR [c]	NR [c]	NR [c]	NR [c]	NR [c]	NR [c]	NR [c]	NR [c]	NR [c]	NR [c]	NR [c]
Fw: SD on PTSD	9	0.165 (-0.127; 0.458)	0.268	10 (8)	0.273	0.190 (-0.228; 0.609)	0.373	1.38 (-1.37; 4.12)	0.358	0.412	NR [b]	GT; p=0.287	33.1
Bw: PTSD on SD	0	NR [c]	NR [c]	NR [c]	NR [c]	NR [c]	NR [c]	NR [c]	NR [c]	NR [c]	NR [c]	NR [c]	NR [c]
Fw: SD on SZ	8	0.213 (0.046; 0.380)	**0.012**	62 (7)	**1.10×10^{-10}**	0.314 (0.015; 0.614)	**0.040**	2.03 (-0.993; 5.05)	0.236	0.278	0.277	DT; p=0.999	31.8
Bw: SZ on SD	176	0.042 (0.026; 0.058)	**3.57×10^{-7}**	402 (175)	**8.73×10^{-20}**	0.020 (-0.007; 0.048)	0.147	-0.043 (-0.137; 0.052)	0.376	0.071	**$<1^{-1000}$**	DT; p=0.836	45.6

Abbreviations: Fw: forward analysis; Bw: backward analysis; SD: social deprivation; ADHD: attention deficit hyperactivity disorder; AN: anorexia nervosa; ANX: anxiety disorder; ASD: autism spectrum disorder; BD: bipolar disorder; MDD: major depressive disorder; OCD: obsessive-compulsive disorder; PTSD: post-traumatic stress disorder; SZ: schizophrenia; MR: mendelian randomization; SNP: single nucleotide polymorphism; IVW: inverse variance weighted (fixed effect); B: effect estimates are log-odds for binary traits (i.e., for mental illnesses) and unstandardized regression coefficient for continuous traits (i.e., for social deprivation); 95% CI: 95% confidence interval; Q: Cochran's Q measure of heterogeneity; df: degree of freedom; WM: weighted median; DT: distortion test; GT: global test.

Legend:

P-value threshold for SNP selection <5e-8.

[a] The Mendelian randomization pleiotropy residual sum and outlier (MR-PRESSO) test identifies possible bias from horizontal pleiotropy. The test consists of three parts, (1) the MR-PRESSO global test which detects horizontal pleiotropy, (2) the outlier corrected causal estimate which corrects for the detected horizontal pleiotropy and (3) the MR-PRESSO distortion test which estimates if the causal estimate is significantly different (at p<0.05) after adjustment for outliers. We conduct all three stages (with the argument NbDistribution=1000, namely using1000 simulation form the null distribution to compute empirical p-values) and present the outlier adjusted causal estimates (OACE) when both global and distortion tests are significant.

[b] We did not run Steiger Test if none of the MR analysis resulted significant (NR: not reported in the cell).

[c] Not enough SNP to perform MR (NR: not reported in the cell).

† Based on chi^2 test.

‡ Based on t-test.

All statistical tests were two-sided. The p-values were not adjusted for multiple testing, therefore p-value<0.05 was considered significant (and reported in bolded text).

Plots - Forward analyses

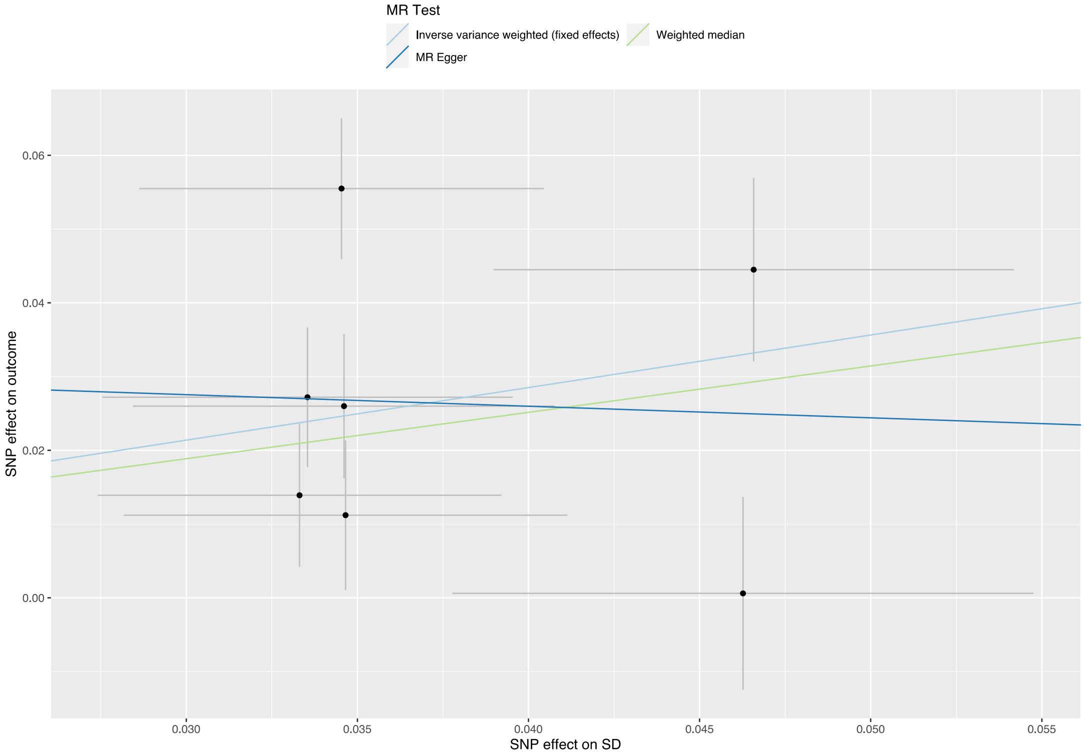

Supplementary Figure 82: scatterplot of social deprivation against ADHD
Abbreviations: MR: Mendelian randomization; SNP: single nucleotide polymorphism; SD: social deprivation; ADHD: attention deficit hyperactivity disorder.
Legend: n=7 SNPs. Each dot represents a SNP, with the slope of each line corresponding to the estimated MR effect per method.

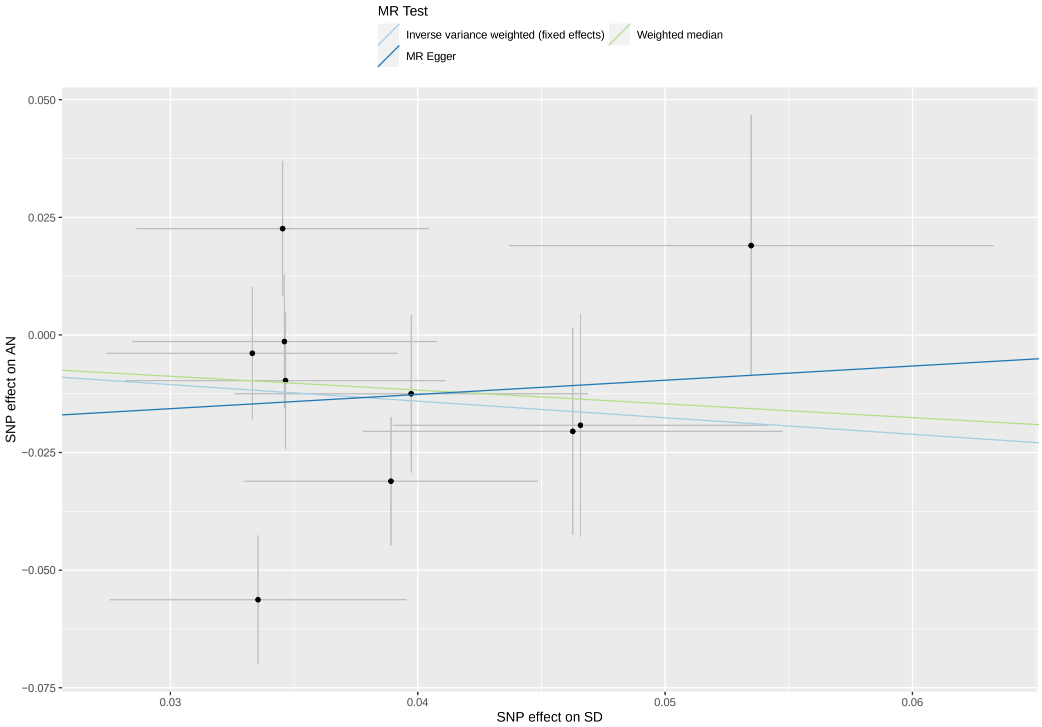

Supplementary Figure 83: scatterplot of social deprivation against AN
Abbreviations: MR: Mendelian randomization; SNP: single nucleotide polymorphism; SD: social deprivation; AN: anorexia nervosa.
Legend: n=10 SNPs. Each dot represents a SNP, with the slope of each line corresponding to the estimated MR effect per method.

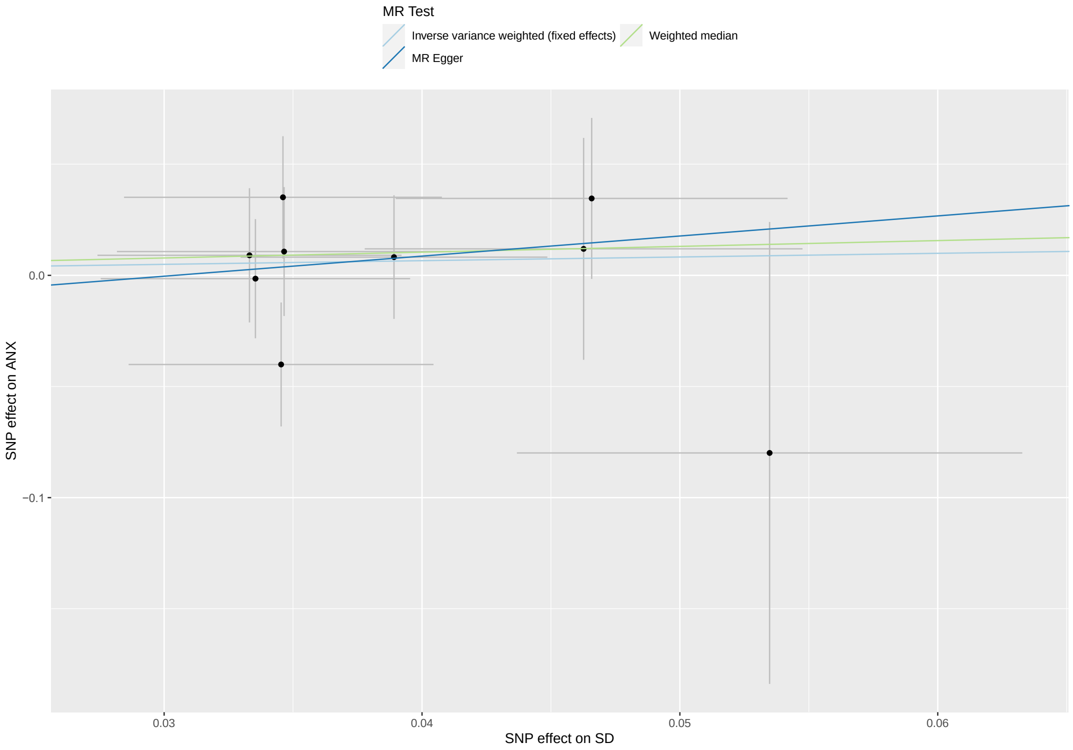

Supplementary Figure 84: scatterplot of social deprivation against ANX
Abbreviations: MR: Mendelian randomization; SNP: single nucleotide polymorphism; SD: social deprivation; ANX: anxiety disorders.
Legend: n=9 SNPs. Each dot represents a SNP, with the slope of each line corresponding to the estimated MR effect per method.

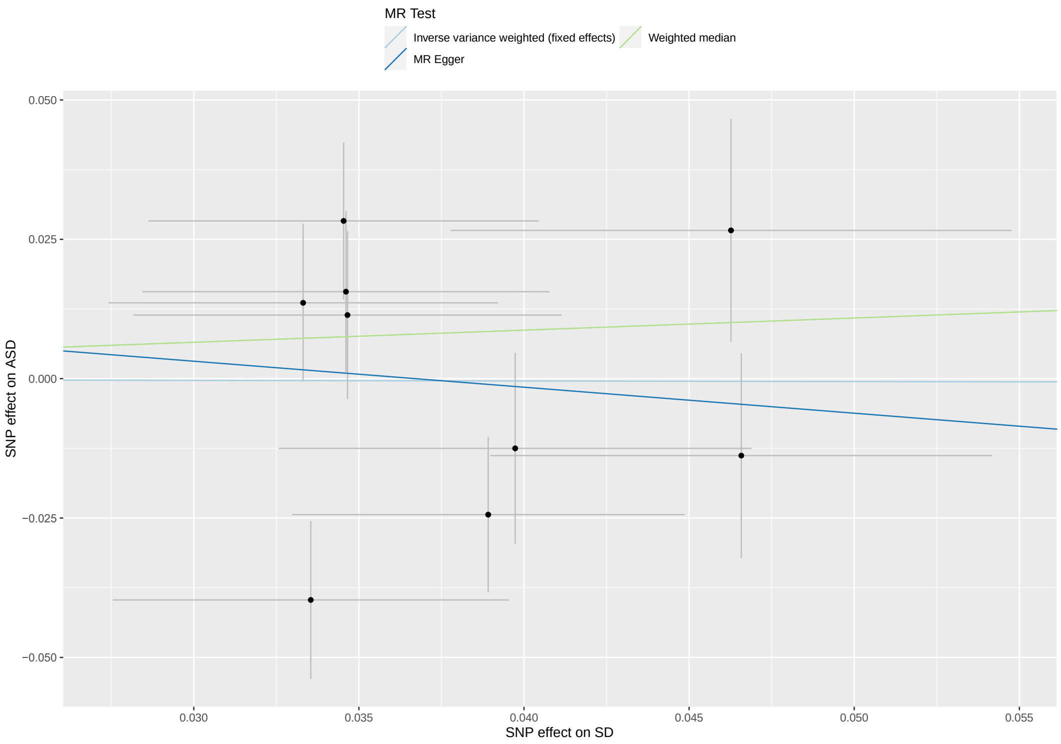

Supplementary Figure 85: scatterplot of social deprivation against ASD
Abbreviations: MR: Mendelian randomization; SNP: single nucleotide polymorphism; SD: social deprivation; ASD: autism spectrum disorders.
Legend: n=9 SNPs. Each dot represents a SNP, with the slope of each line corresponding to the estimated MR effect per method.

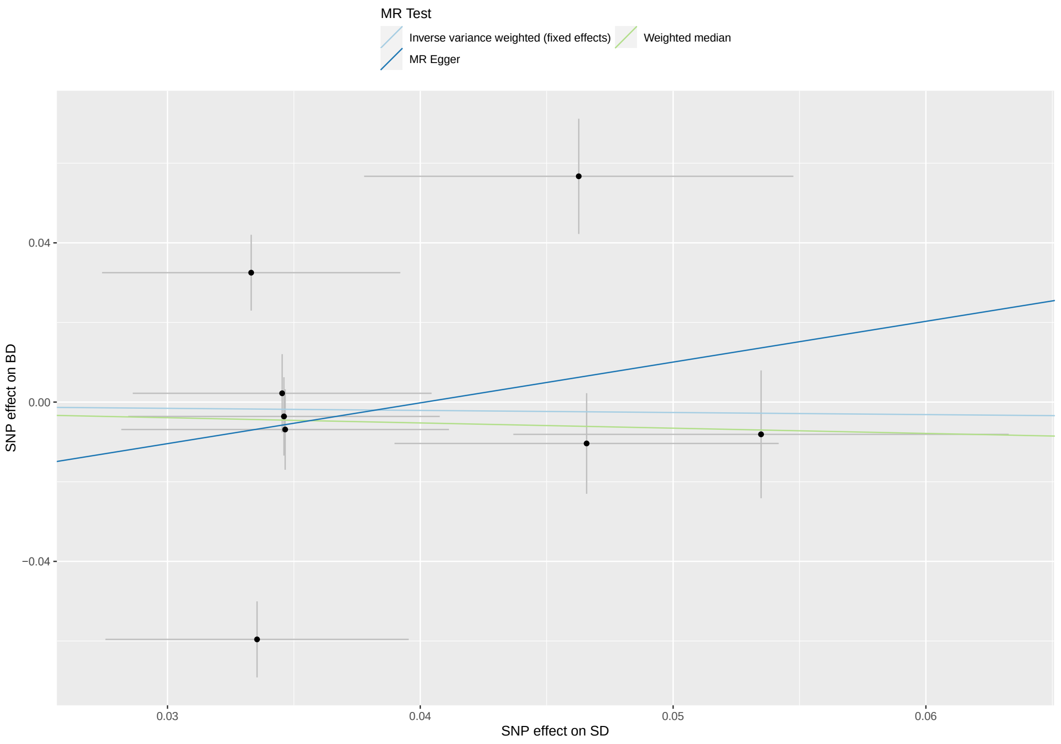

Supplementary Figure 86: scatterplot of social deprivation against BD
Abbreviations: MR: Mendelian randomization; SNP: single nucleotide polymorphism; SD: social deprivation; BD: bipolar disorder.

Legend: n=8 SNPs. Each dot represents a SNP, with the slope of each line corresponding to the estimated MR effect per method.

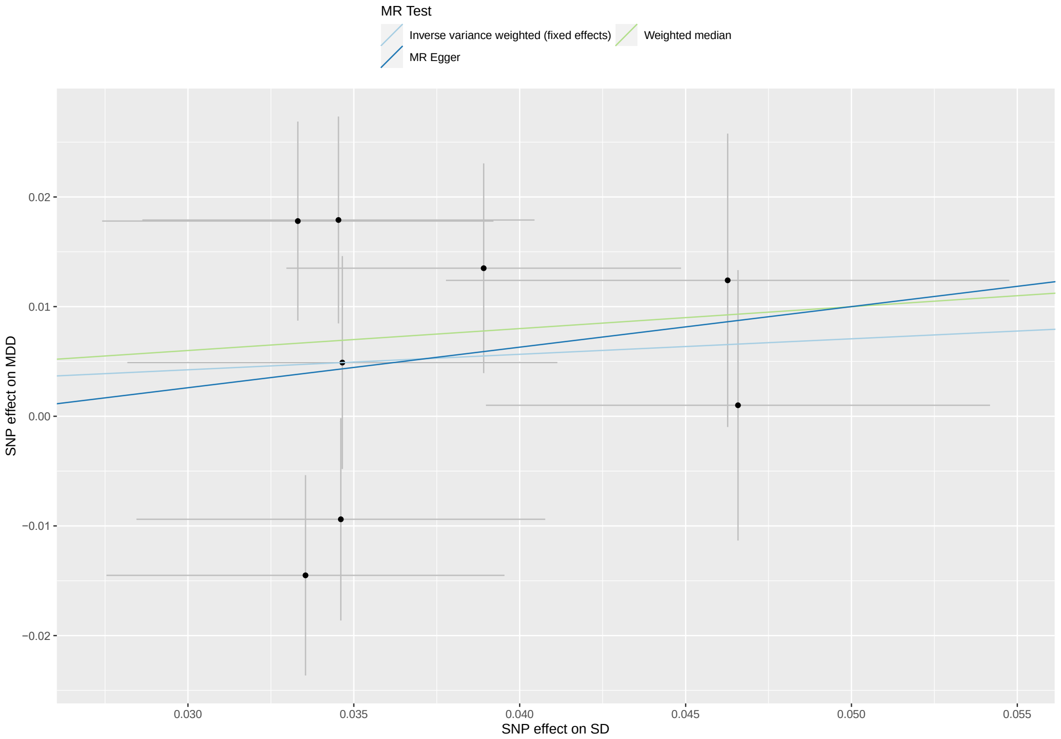

Supplementary Figure 87: scatterplot of social deprivation against MDD
Abbreviations: MR: Mendelian randomization; SNP: single nucleotide polymorphism; SD: social deprivation; MDD: major depressive disorder.
Legend: n=8 SNPs. Each dot represents a SNP, with the slope of each line corresponding to the estimated MR effect per method.

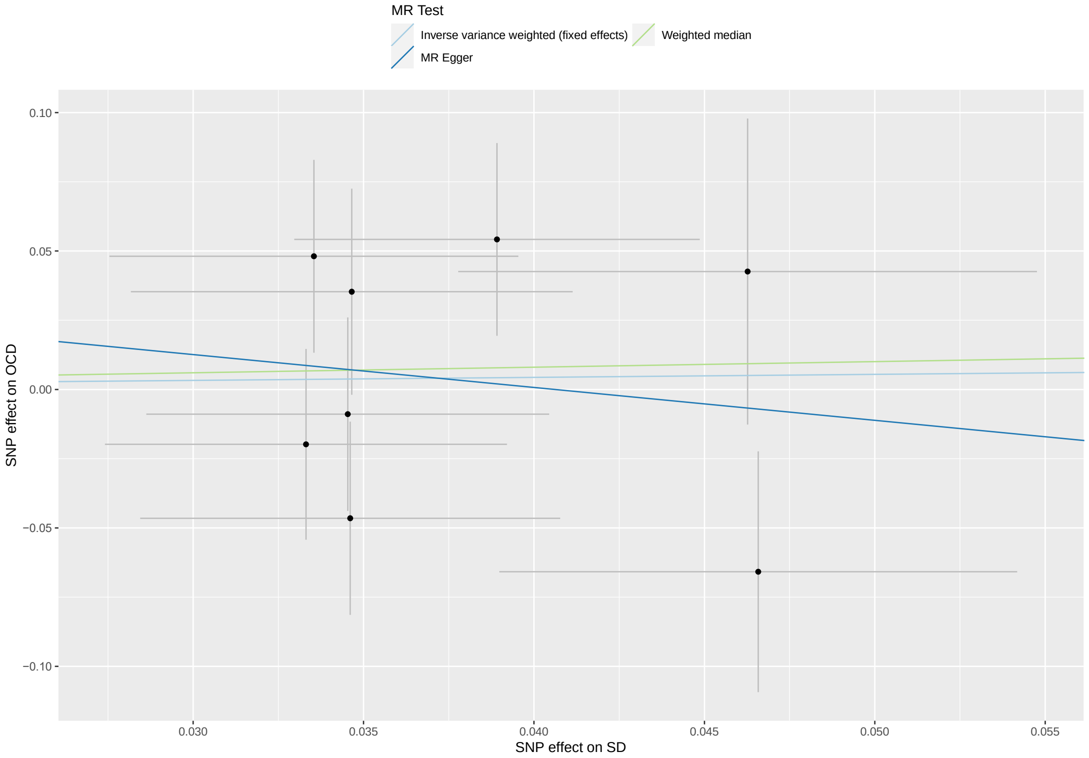

Supplementary Figure 88: scatterplot of social deprivation against OCD
Abbreviations: MR: Mendelian randomization; SNP: single nucleotide polymorphism; SD: social deprivation; OCD: obsessive-compulsive disorder.
Legend: n=8 SNPs. Each dot represents a SNP, with the slope of each line corresponding to the estimated MR effect per method.

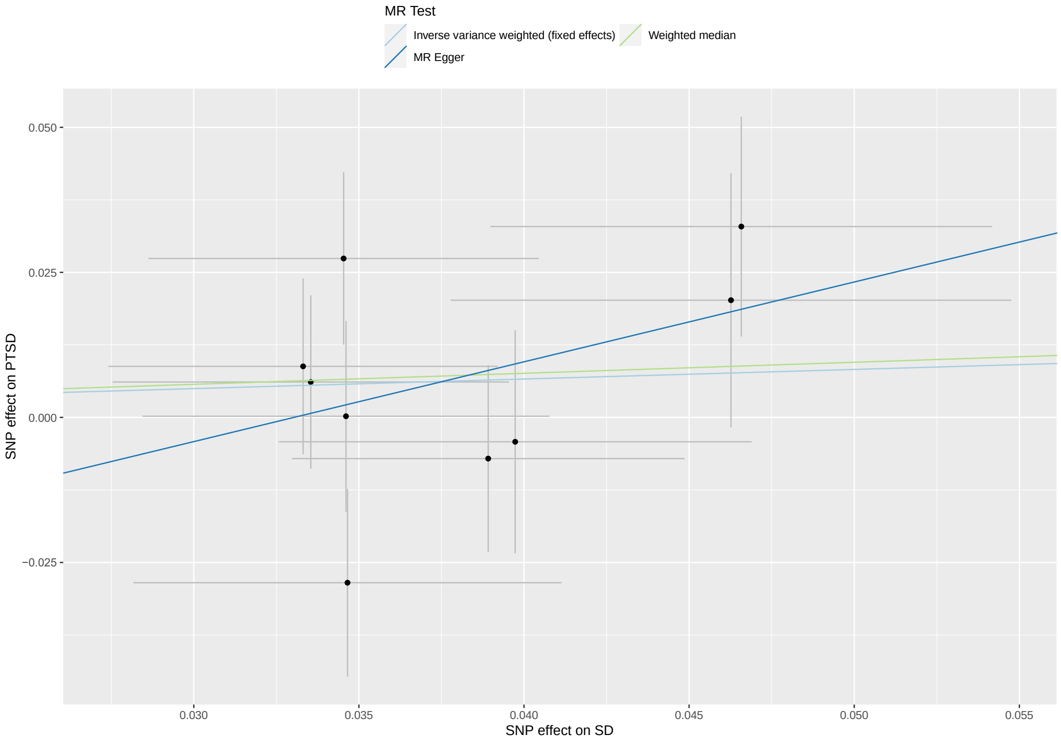

Supplementary Figure 89: scatterplot of social deprivation against PTSD
Abbreviations: MR: Mendelian randomization; SNP: single nucleotide polymorphism; SD: social deprivation; PTSD: post-traumatic stress disorder.
Legend: n=9 SNPs. Each dot represents a SNP, with the slope of each line corresponding to the estimated MR effect per method.

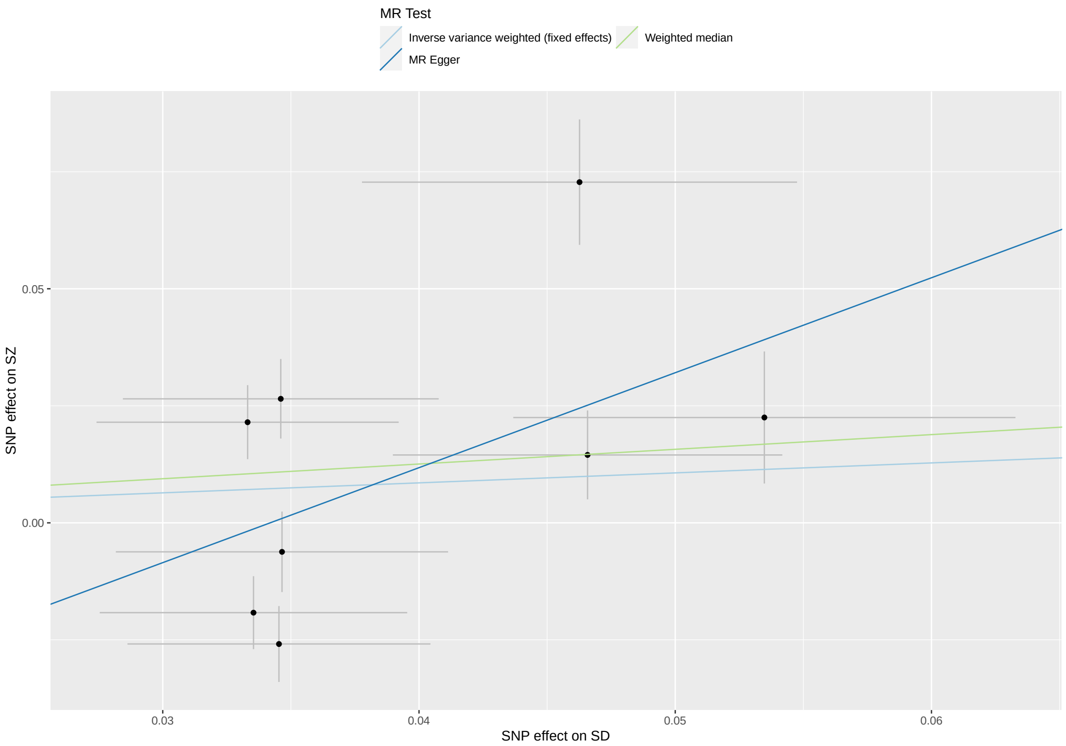

Supplementary Figure 90: scatterplot of social deprivation against SZ
Abbreviations: MR: Mendelian randomization; SNP: single nucleotide polymorphism; SD: social deprivation; SZ: schizophrenia.
Legend: n=8 SNPs. Each dot represents a SNP, with the slope of each line corresponding to the estimated MR effect per method.

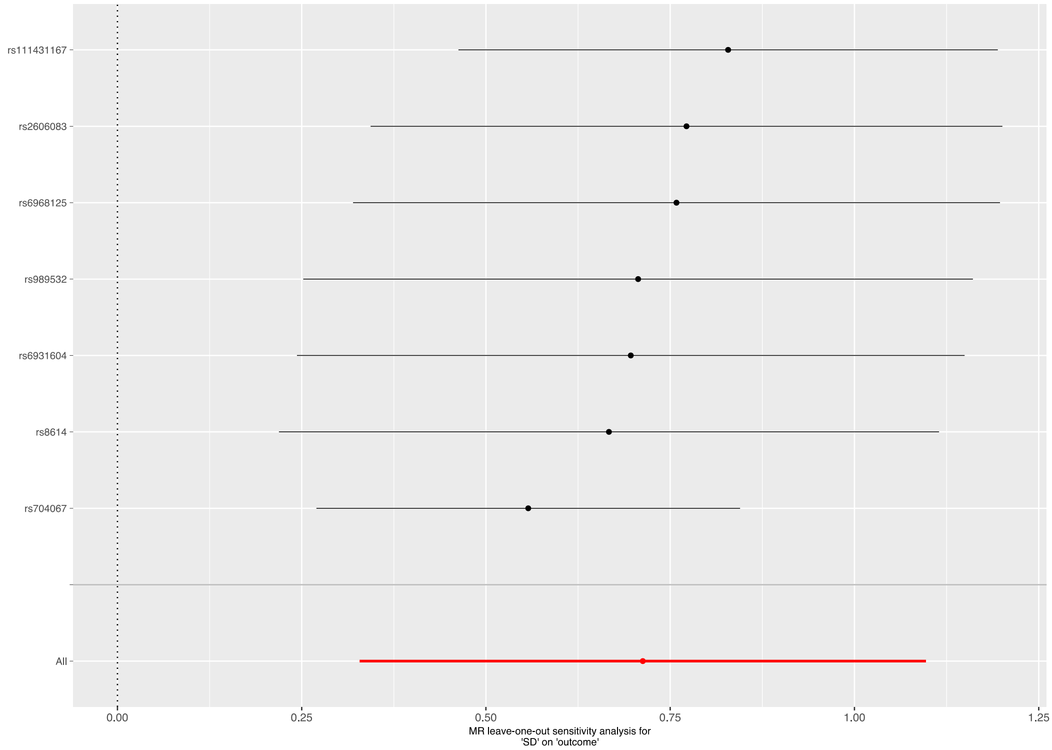

Supplementary Figure 91: leave-one-out analysis of social deprivation against ADHD
Abbreviations: MR: Mendelian randomization; SD: social deprivation; ADHD: attention deficit hyperactivity disorder.

Legend: n=7 SNPs. Forest plot of individual and combined SNP MR estimated effect size. The effect estimates represent the log-odds for ADHD per one standard deviation (1 SD) increase in the mean Towsend deprivation index, and the error bars represent 95% CIs.

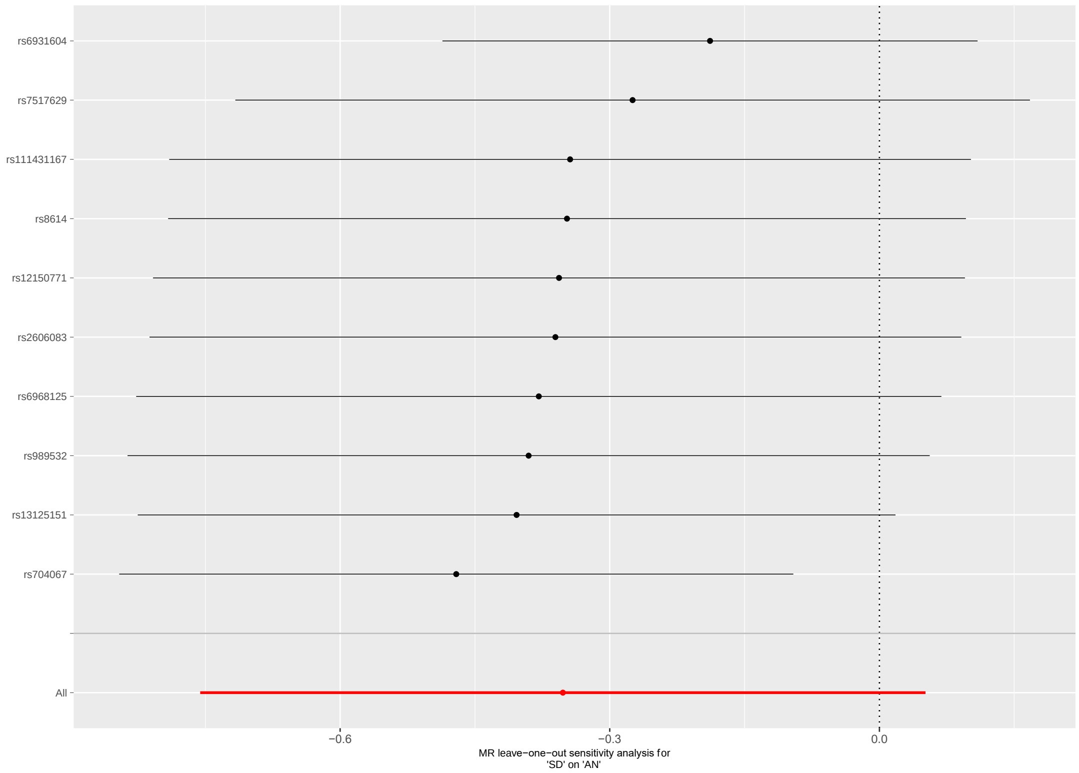

Supplementary Figure 92: leave-one-out analysis of social deprivation against AN
Abbreviations: MR: Mendelian randomization; SD: social deprivation; AN: anorexia nervosa.
Legend: n=10 SNPs. Forest plot of individual and combined SNP MR estimated effect size. The effect estimates represent the log-odds for AN per one standard deviation (1 SD) increase in the mean Towsend deprivation index, and the error bars represent 95% CIs.

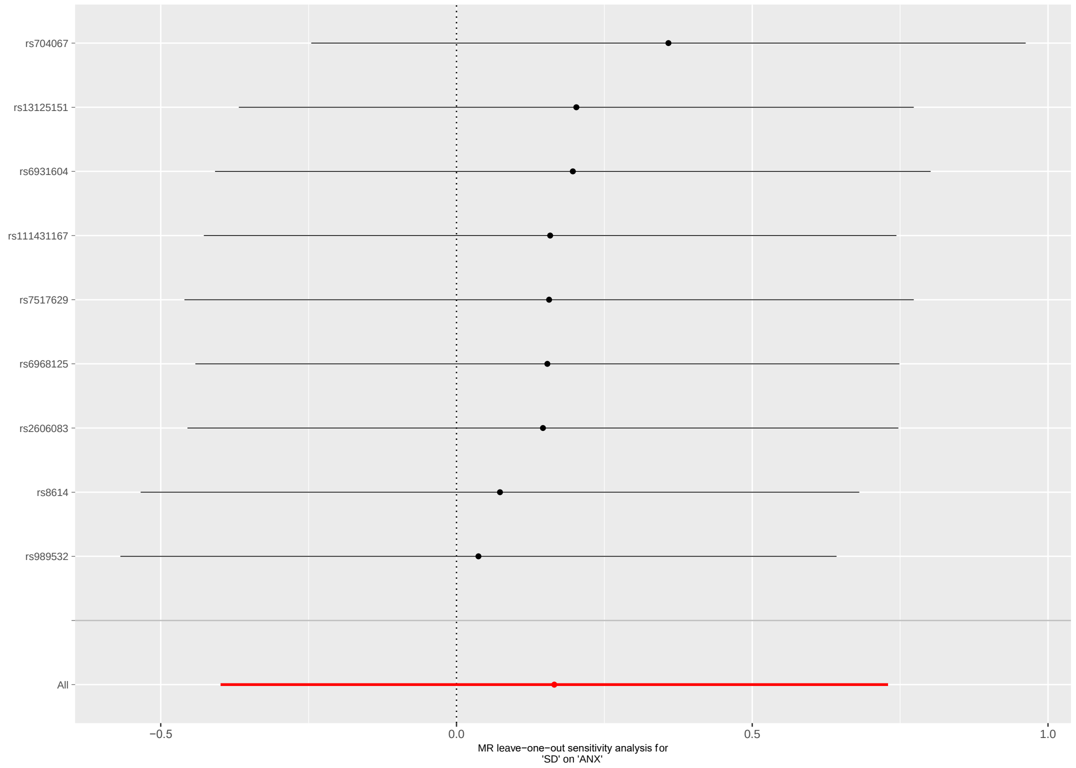

Supplementary Figure 93: leave-one-out analysis of social deprivation against ANX
Abbreviations: MR: Mendelian randomization; SD: social deprivation; ANX: anxiety disorders.
Legend: n=9 SNPs. Forest plot of individual and combined SNP MR estimated effect size. The effect estimates represent the log-odds for ANX per one standard deviation (1 SD) increase in the mean Towsend deprivation index, and the error bars represent 95% CIs.

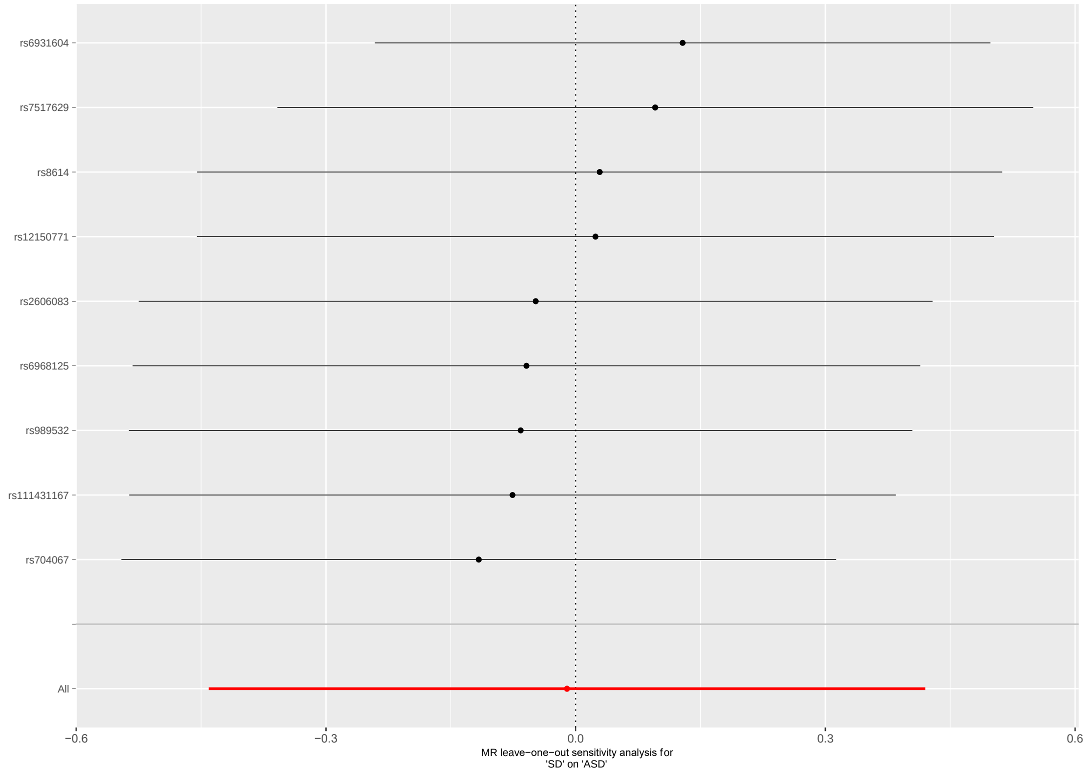

Supplementary Figure 94: leave-one-out analysis of social deprivation against ASD

Abbreviations: MR: Mendelian randomization; SD: social deprivation; ASD: autism spectrum disorders.
Legend: n=9 SNPs. Forest plot of individual and combined SNP MR estimated effect size. The effect estimates represent the log-odds for ASD per one standard deviation (1 SD) increase in the mean Towsend deprivation index, and the error bars represent 95% CIs.

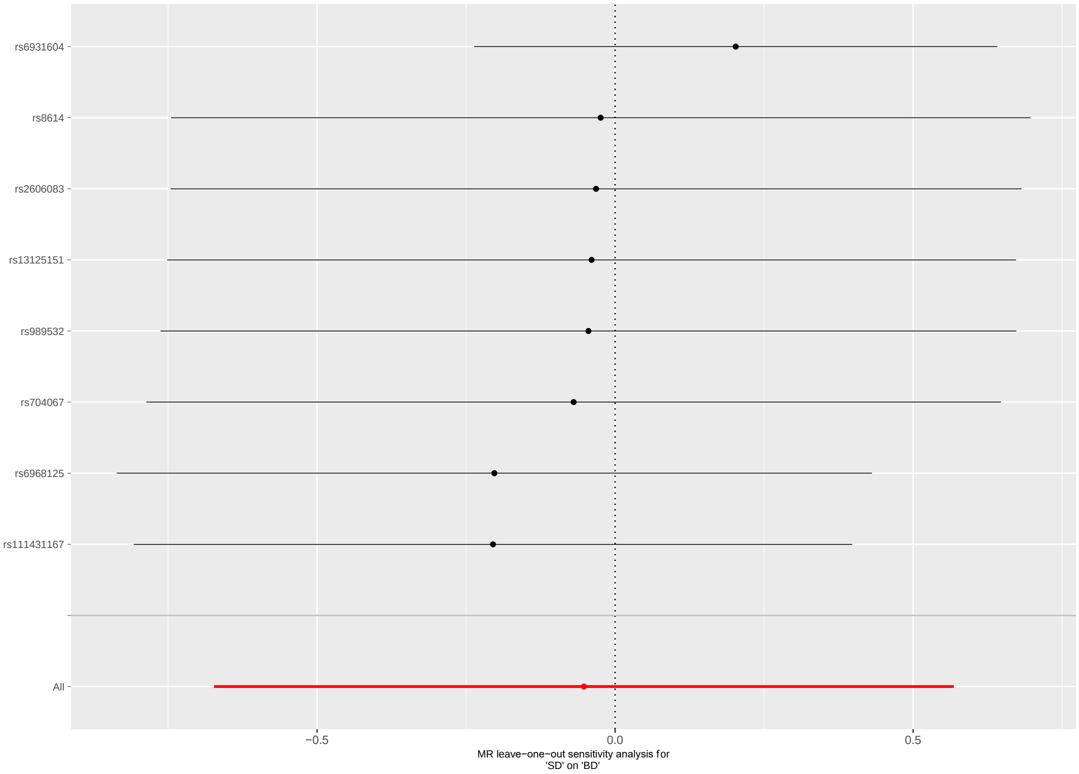

Supplementary Figure 95: leave-one-out analysis of social deprivation against BD
Abbreviations: MR: Mendelian randomization; SD: social deprivation; BD: bipolar disorder.
Legend: n=8 SNPs. Forest plot of individual and combined SNP MR estimated effect size. The effect estimates represent the log-odds for BD per one standard deviation (1 SD) increase in the mean Towsend deprivation index, and the error bars represent 95% CIs.

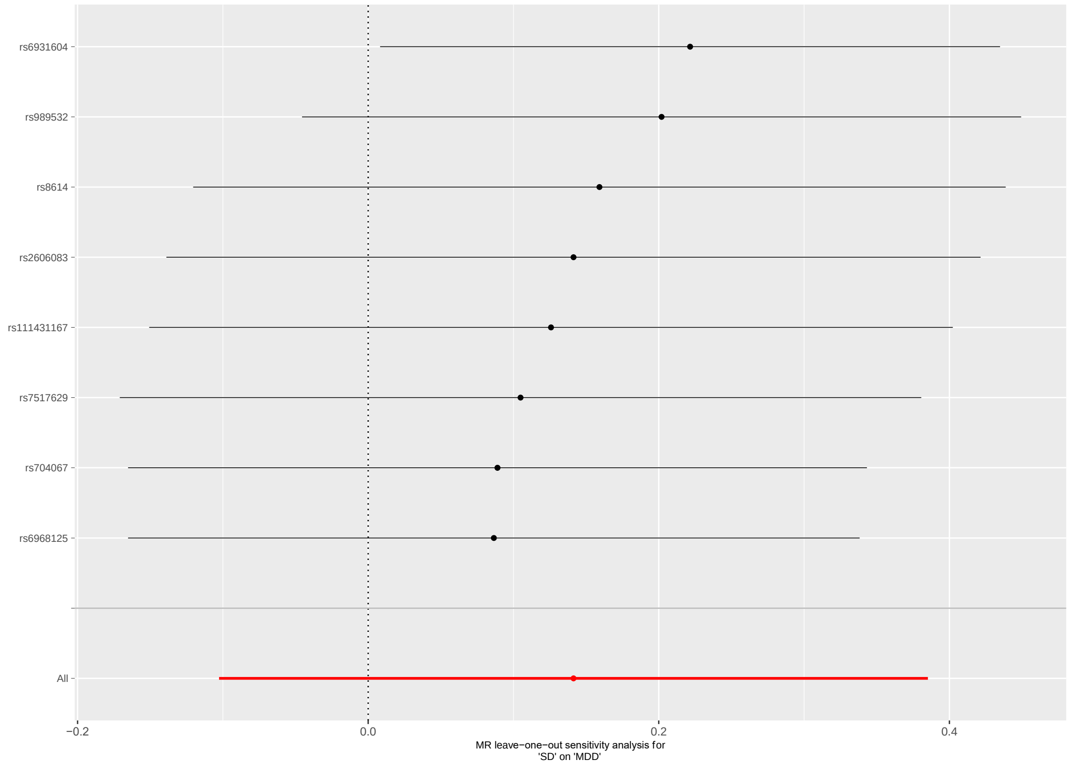

Supplementary Figure 96: leave-one-out analysis of social deprivation against MDD
Abbreviations: MR: Mendelian randomization; SD: social deprivation; MDD: major depressive disorder.
Legend: n=8 SNPs. Forest plot of individual and combined SNP MR estimated effect size. The effect estimates represent the log-odds for MDD per one standard deviation (1 SD) increase in the mean Towsend deprivation index, and the error bars represent 95% CIs.

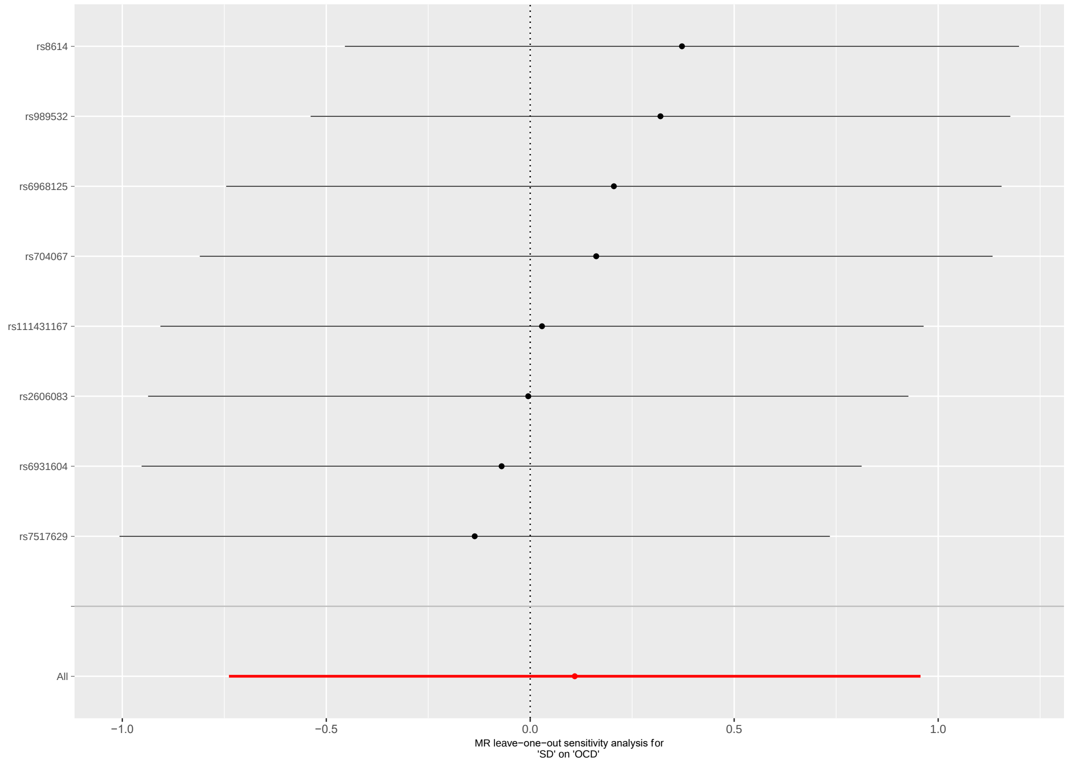

Supplementary Figure 97: leave-one-out analysis of social deprivation against OCD

Abbreviations: MR: Mendelian randomization; SD: social deprivation; OCD: obsessive-compulsive disorder.
Legend: n=8 SNPs. Forest plot of individual and combined SNP MR estimated effect size. The effect estimates represent the log-odds for OCD per one standard deviation (1 SD) increase in the mean Towsend deprivation index, and the error bars represent 95% CIs.

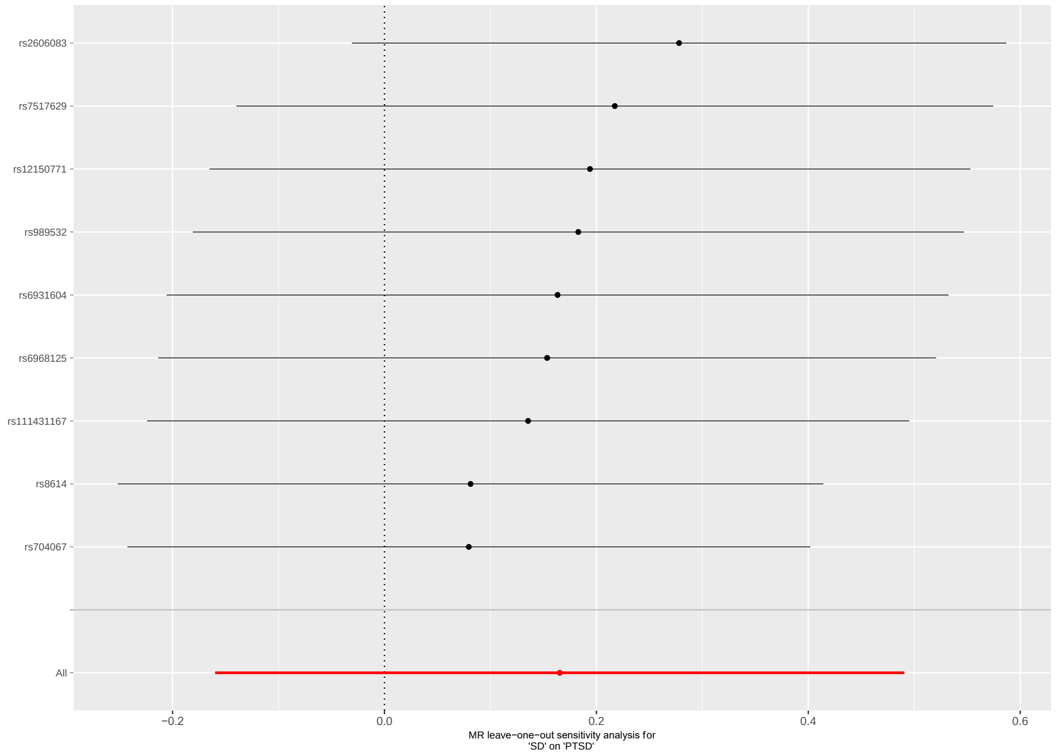

Supplementary Figure 98: leave-one-out analysis of social deprivation against PTSD
Abbreviations: MR: Mendelian randomization; SD: social deprivation; PTSD: post-traumatic stress disorder.
Legend: n=9 SNPs. Forest plot of individual and combined SNP MR estimated effect size. The effect estimates represent the log-odds for PTSD per one standard deviation (1 SD) increase in the mean Towsend deprivation index, and the error bars represent 95% CIs.

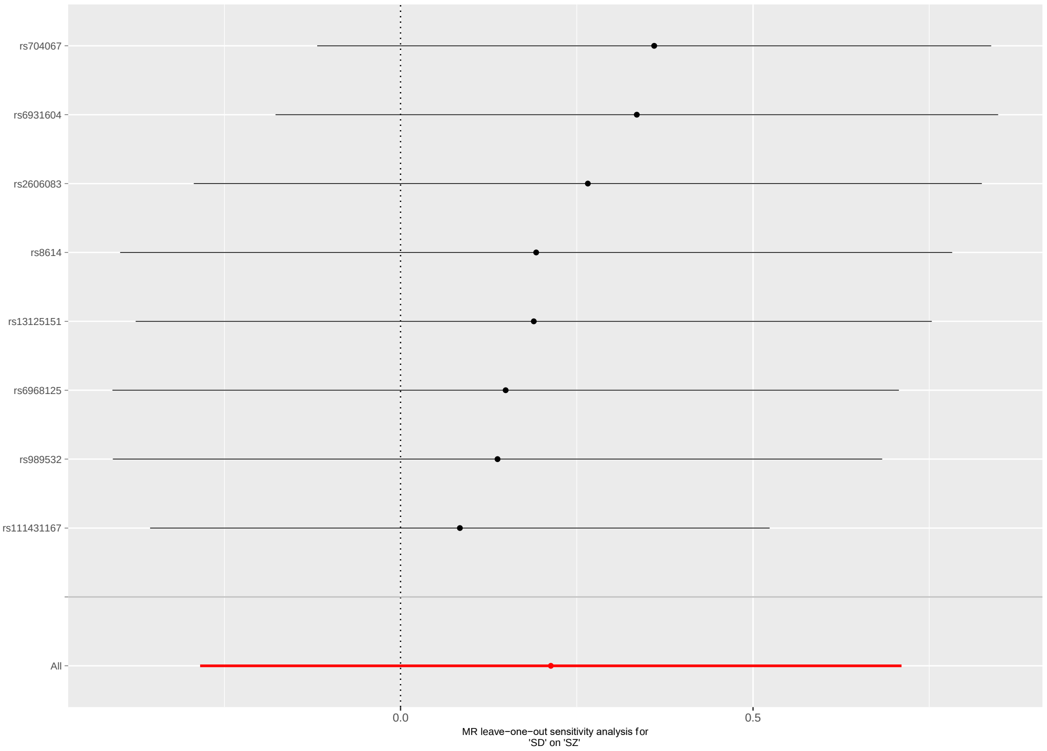

Supplementary Figure 99: leave-one-out analysis of social deprivation against SZ
Abbreviations: MR: Mendelian randomization; SD: social deprivation; SZ: schizophrenia.
Legend: n=8 SNPs. Forest plot of individual and combined SNP MR estimated effect size.
The effect estimates represent the log-odds for SZ per one standard deviation (1 SD) increase in the mean Towsend deprivation index, and the error bars represent 95% CIs.

Plots - Backward analyses

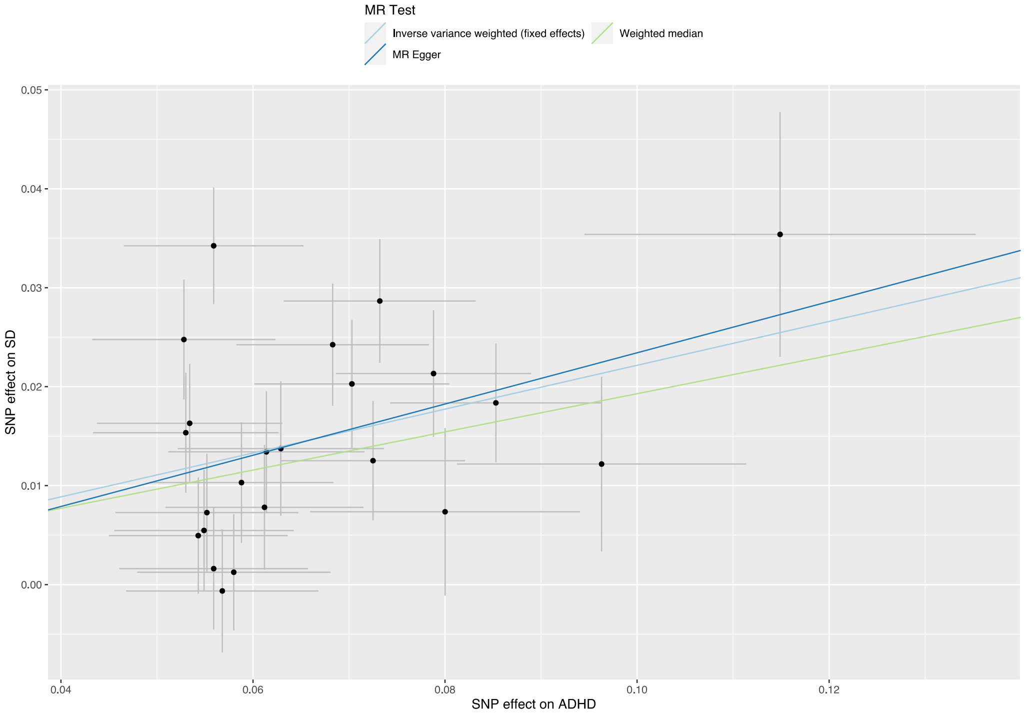

Supplementary Figure 100: scatterplot of ADHD against social deprivation
Abbreviations: MR: Mendelian randomization; SD: social deprivation; ADHD: attention deficit hyperactivity disorder.
Legend: n=23 SNPs. Each dot represents a SNP, with the slope of each line corresponding to the estimated MR effect per method.

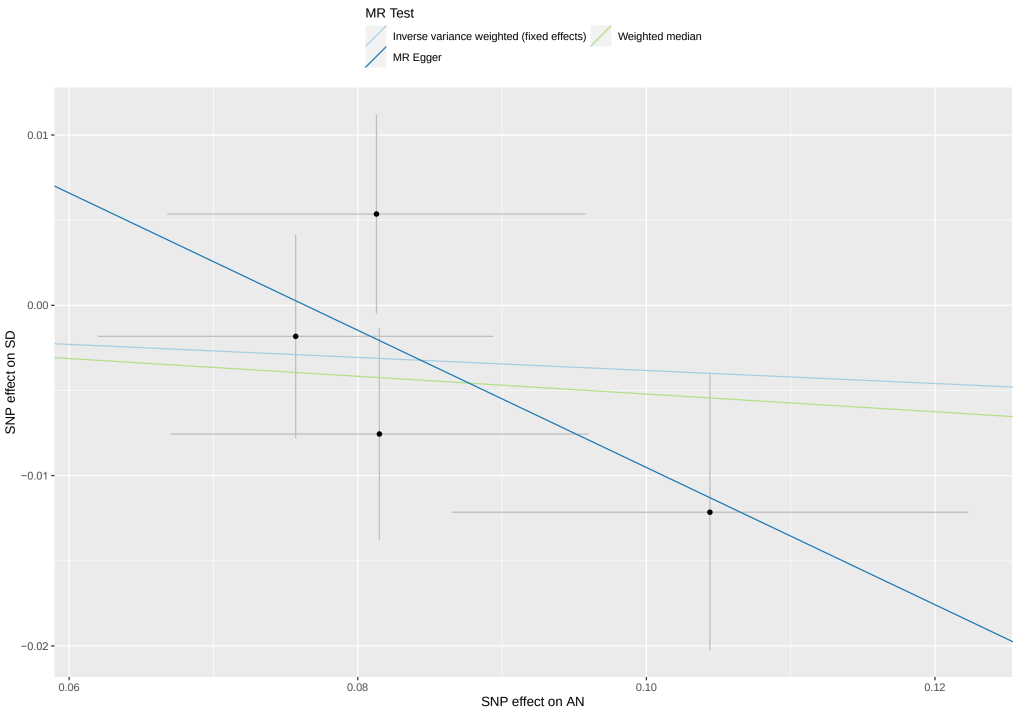

Supplementary Figure 101: scatterplot analysis of AN against social deprivation
Abbreviations: MR: Mendelian randomization; SD: social deprivation; AN: anorexia nervosa.
Legend: n=4 SNPs. Each dot represents a SNP, with the slope of each line corresponding to the estimated MR effect per method.

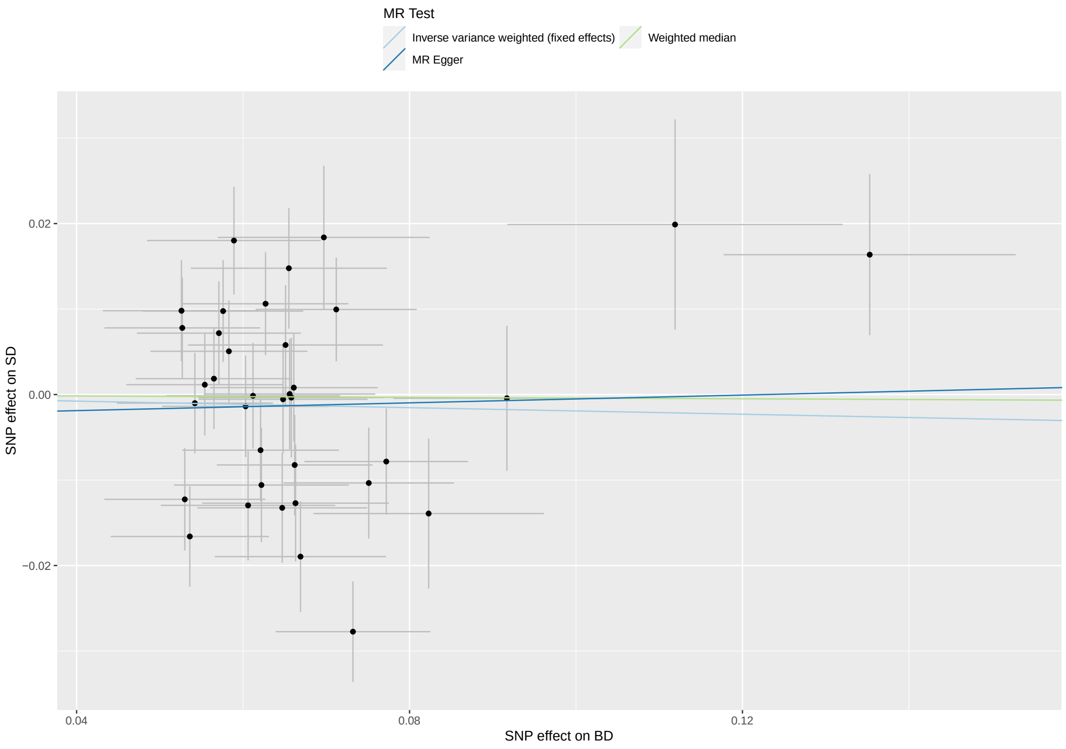

Supplementary Figure 102: scatterplot analysis of BD against social deprivation
Abbreviations: MR: Mendelian randomization; SD: social deprivation; BD: bipolar disorder.
Legend: n=36 SNPs. Each dot represents a SNP, with the slope of each line corresponding to the estimated MR effect per method.

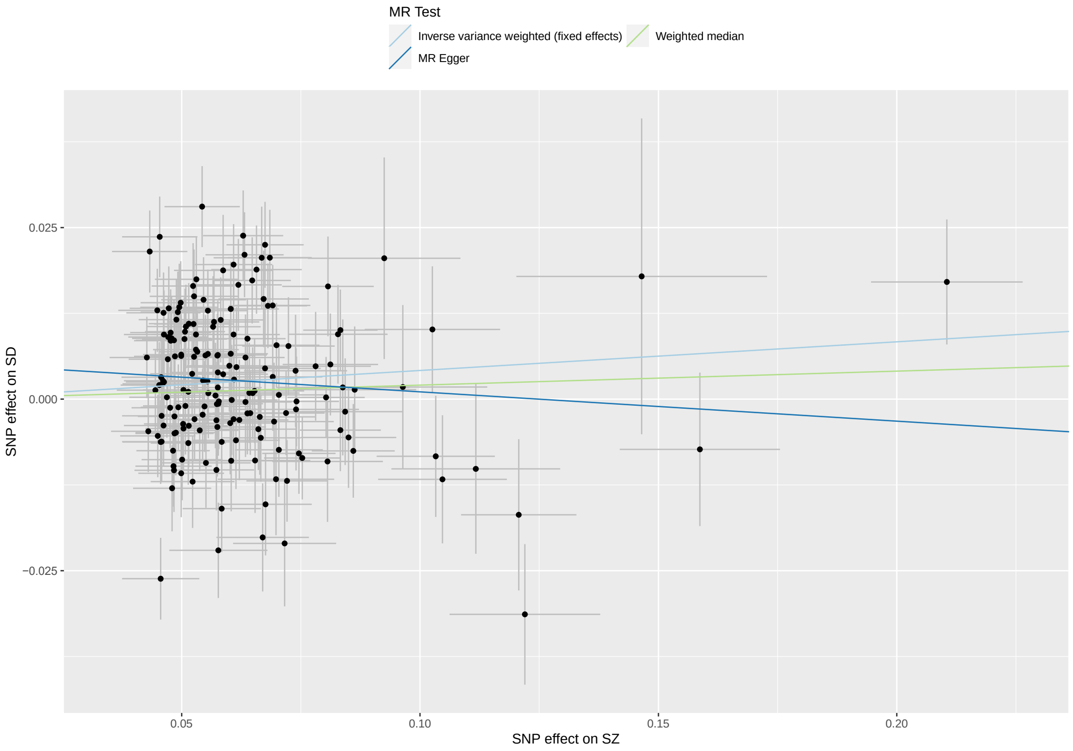

Supplementary Figure 103: scatterplot of SZ against social deprivation
Abbreviations: MR: Mendelian randomization; SD: social deprivation; SZ: schizophrenia.
Legend: n=176 SNPs. Each dot represents a SNP, with the slope of each line corresponding to the estimated MR effect per method.

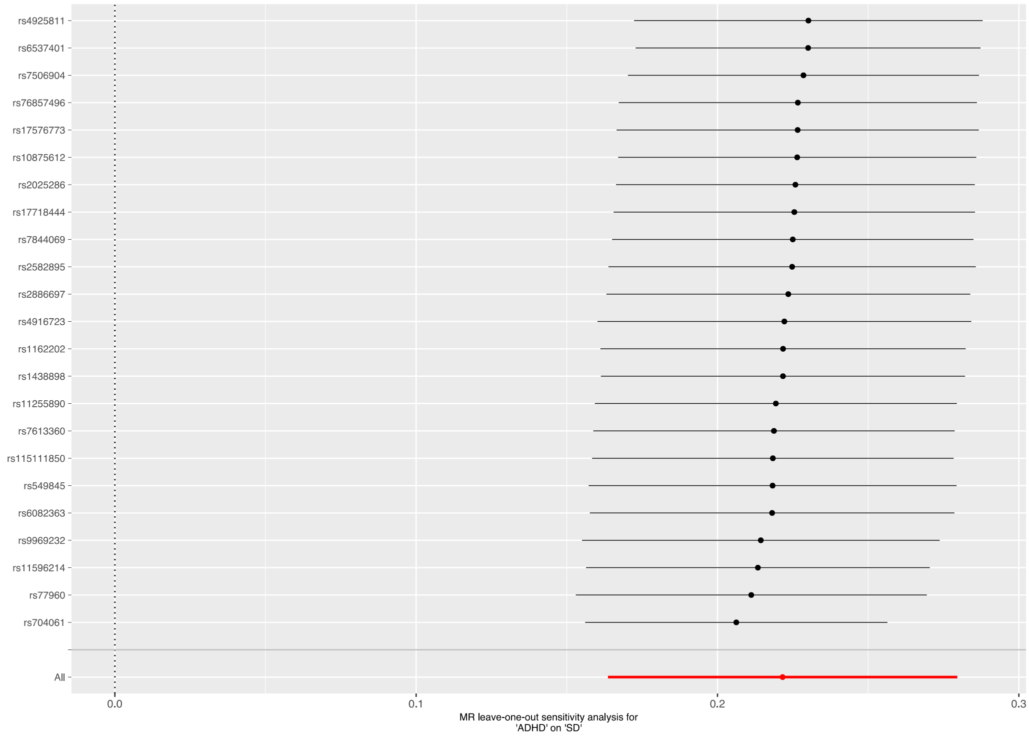

Supplementary Figure 104: leave-one-out analysis of ADHD against social deprivation
Abbreviations: MR: Mendelian randomization; SD: social deprivation; ADHD: attention deficit hyperactivity disorder.
Legend: n=23 SNPs. Forest plot of individual and combined SNP MR estimated effect size. The effect estimates represent the linear effect for the mean Towsend deprivation index per ADHD status, and the error bars represent 95% CIs.

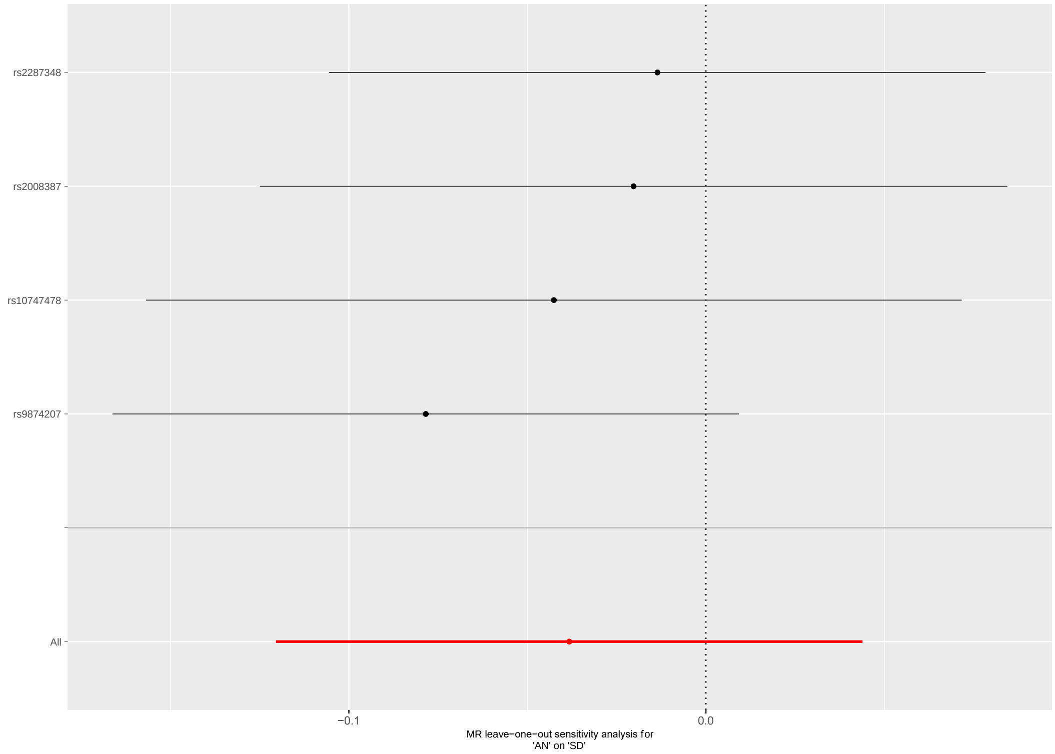

Supplementary Figure 105: leave-one-out analysis of AN against social deprivation

Abbreviations: MR: Mendelian randomization; SD: social deprivation; AN: anorexia nervosa.
Legend: n=4 SNPs. Forest plot of individual and combined SNP MR estimated effect size. The effect estimates represent the linear effect for the mean Towsend deprivation index per AN status, and the error bars represent 95% CIs.

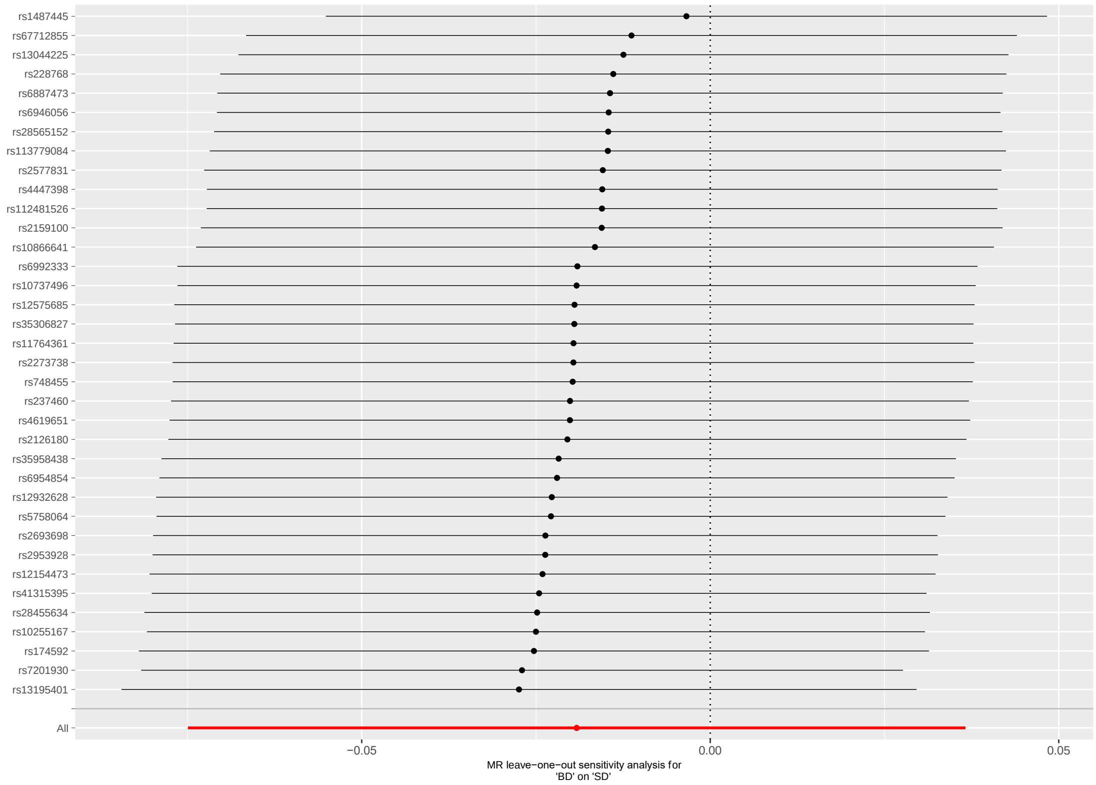

Supplementary Figure 106: leave-one-out analysis of BD against social deprivation
Abbreviations: MR: Mendelian randomization; SD: social deprivation; BD: bipolar disorder.
Legend: n=36 SNPs. Forest plot of individual and combined SNP MR estimated effect size. The effect estimates represent the linear effect for the mean Towsend deprivation index per BD status, and the error bars represent 95% CIs.

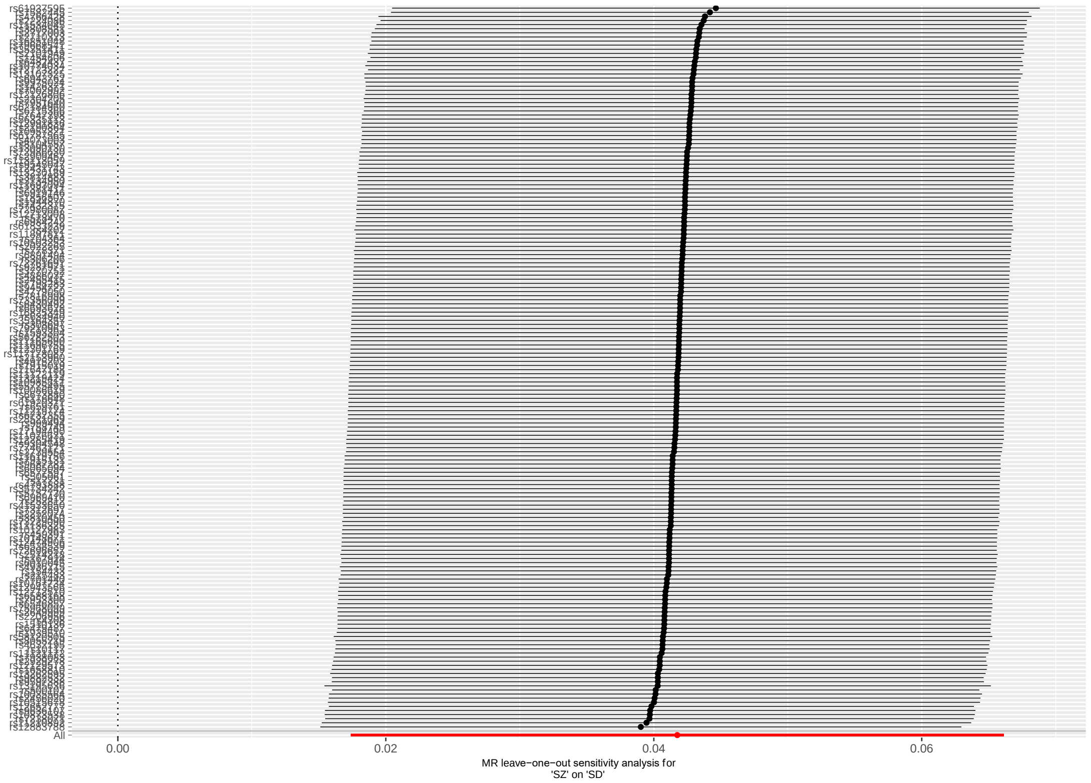

Supplementary Figure 107: leave-one-out analysis of SZ against social deprivation
Abbreviations: MR: Mendelian randomization; SD: social deprivation; SZ: schizophrenia.
Legend: n=176 SNPs. Forest plot of individual and combined SNP MR estimated effect size. The effect estimates represent the linear effect for the mean Towsend deprivation index per SZ status, and the error bars represent 95% CIs.

Supplementary Table 19: CAUSE results of the relations between social deprivation and mental illness

**Model 1**	**Model 2**	**Δ ELPD**	**SE Δ ELPD**	**z-score**	**p-value†**
		Fw: SD on ADHD			
Null	Sharing	-19.58	5.15	-3.80	**1.45×10^{-4}**
Null	Causal	-24.85	6.50	-3.82	**1.33×10^{-4}**
Sharing	Causal	-5.27	1.54	-3.43	**6.04×10^{-4}**
		Bw: ADHD on SD			
Null	Sharing	-40.14	7.96	-5.05	**4.42×10^{-7}**
Null	Causal	-45.66	9.11	-5.01	**5.44×10^{-7}**
Sharing	Causal	-5.52	1.56	-3.54	**4.00×10^{-4}**
		Fw: SD on AN			
Null	Sharing	0.36	0.09	3.98	**6.89×10^{-5}**
Null	Causal	0.94	0.57	1.64	0.101
Sharing	Causal	0.57	0.49	1.18	0.238
		Bw: AN on SD			
Null	Sharing	0.53	0.07	7.61	**2.74×10^{-14}**
Null	Causal	1.34	0.15	9.09	**9.90×10^{-20}**
Sharing	Causal	0.82	0.11	7.40	**1.36×10^{-13}**
		Fw: SD on ANX			
Null	Sharing	0.35	0.33	1.09	0.276
Null	Causal	1.00	0.82	1.23	0.219

Sharing	Causal	0.65	0.51	1.27	0.204
Bw: ANX on SD					
Null	Sharing	0.14	0.07	2.12	**0.034**
Null	Causal	0.61	0.76	0.80	0.424
Sharing	Causal	0.46	0.70	0.67	0.503
Fw: SD on ASD					
Null	Sharing	0.45	0.08	5.84	**5.22×10^{-9}**
Null	Causal	1.26	0.22	5.67	**1.43×10^{-8}**
Sharing	Causal	0.81	0.19	4.32	**1.56×10^{-5}**
Bw: ASD on SD					
Null	Sharing	0.38	0.09	4.41	**1.03×10^{-5}**
Null	Causal	1.09	0.51	2.14	**0.032**
Sharing	Causal	0.72	0.46	1.56	0.119
Fw: SD on BD					
Null	Sharing	-1.00	0.76	-1.30	0.192
Null	Causal	-4.61	2.73	-1.69	0.091
Sharing	Causal	-3.61	1.97	-1.83	0.067
Bw: BD on SD					
Null	Sharing	0.01	0.50	0.01	0.499
Null	Causal	-1.01	2.03	-0.50	0.309
Sharing	Causal	-1.01	1.54	-0.66	0.256
Fw: SD on MDD					
Null	Sharing	-2.55	1.65	-1.55	0.121
Null	Causal	5.44	3.40	-1.60	0.110
Sharing	Causal	-2.89	1.80	-1.61	0.107
Bw: MDD on SD					
Null	Sharing	-0.86	0.93	-0.92	0.358
Null	Causal	-3.39	2.77	-1.23	0.219
Sharing	Causal	-2.53	1.87	-1.36	0.174
Fw: SD on OCD					
Null	Sharing	0.48	0.18	2.67	**0.008**
Null	Causal	1.40	0.37	3.84	**1.23×10^{-4}**
Sharing	Causal	0.93	0.20	4.60	**4.22×10^{-6}**
Bw: OCD on SD					
Null	Sharing	0.23	0.07	3.40	**0.001**
Null	Causal	0.99	0.20	4.90	**9.58×10^{-7}**
Sharing	Causal	0.76	0.15	5.03	**4.90×10^{-7}**
Fw: SD on PTSD					
Null	Sharing	-3.57	2.40	-1.49	0.136
Null	Causal	-4.38	3.39	-1.29	0.197
Sharing	Causal	-0.81	1.17	-0.69	0.490
Bw: PTSD on SD					
Null	Sharing	0.16	0.07	2.15	**0.032**
Null	Causal	0.74	0.65	1.14	0.254
Sharing	Causal	0.59	0.59	1.00	0.317
Fw: SD on SZ					
Null	Sharing	-0.77	0.64	-1.19	0.234
Null	Causal	-4.27	2.66	-1.61	0.107
Sharing	Causal	-3.50	2.02	-1.74	0.082

Bw: SZ on SD					
Null	Sharing	-4.63	2.41	-1.92	0.055
Null	Causal	-7.90	3.99	-1.98	**0.048**
Sharing	Causal	-3.28	1.63	-2.01	**0.044**

Abbreviations: MR: mendelian randomization; ELPD: expected log pointwise posterior density; 95%CI: 95% confidence interval; SE: standard error; Fwd: forward MR; Bwd: backward MR; SD: social deprivation; ADHD: attention deficit hyperactivity disorder; AN: anorexia nervosa; ANX: anxiety disorder; ASD: autism spectrum disorder; BD: bipolar disorder; MDD: major depressive disorder; OCD: obsessive-compulsive disorder; PTSD: post-traumatic stress disorder; SZ: schizophrenia.
Legend: † Based on t-test. The p-values are two-sided and not adjusted for multiple testing, therefore p-value<0.05 was considered significant (and reported in bolded text).

Supplementary Table 20: Results of univariable bidirectional Mendelian Randomization of social deprivation against mental illnesses, after Steiger filtering

MR	N SNP	IVW, B (95% CI)	IVW p-value	WM, B (95% CI)	WM p-value	MR-Egger, B (95% CI)	MR-Egger p-value	Egger intercept p-value	Mean F
SD on ADHD	6	0.557 (0.331; 0.784)	**1.36× 10^{-6}**	0.526 (0.205; 0.847)	**0.001**	0.326 (-1.95; 2.60)	0.793	0.088	31.8
SD on AN	9	-0.188 (-0.470; 0.093)	0.189	-0.281 (-0.664; 0.102)	0.150	-0.761 (-3.10; 1.57)	0.543	0.299	33.0
SD on ANX	9	0.165 (-0.399; 0.730)	0.566	0.261 (-0.486; 1.01)	0.494	0.904 (-3.63; 5.44)	0.708	0.355	33.0
SD on ASD	7	0.028 (-0.276; 0.332)	0.855	0.169 (-0.240; 0.579)	0.418	-1.19 (-4.27; 1.89)	0.483	**0.027**	33.3
SD on BD	5	-0.124 (-0.374; 0.127)	0.334	-0.152 (-0.462; 0.158)	0.337	-0.418 (-1.90; 1.07)	0.620	0.880	32.3
SD on MDD	8	0.141 (-0.046; 0.328)	0.138	0.200 (-0.060; 0.460)	0.132	0.370 (-1.80; 2.54)	0.749	0.888	33.4
SD on OCD	2	-0.422 (1.84; 0.995)	0.559	NR	NR	NR	NR	NR	33.0
SD on PTSD	9	0.165 (-0.127; 0.458)	0.268	0.190 (-0.232; 0.612)	0.377	1.38 (-1.37; 4.12)	0.358	0.424	33.1
SD on SZ	7	0.084 (-0.091; 0.259)	0.345	0.247 (-0.059; 0.553)	0.114	1.18 (-1.76; 4.12)	0.468	0.686	32.1

Abbreviations: SD: social deprivation; ADHD: attention deficit hyperactivity disorder; AN: anorexia nervosa; ANX: anxiety disorder; ASD: autism spectrum disorders; BD: bipolar disorder; MDD: major depressive disorder; OCD: obsessive-compulsive disorder; PTSD: post-traumatic stress disorder; SZ: schizophrenia; MR: mendelian randomization; SNP: single nucleotide polymorphism; IVW: inverse variance weighted (fixed effect); B: effect estimates are log-odds; 95% CI: 95% confidence interval; WM: weighted median; NR: not reported because not enough SNP to perform MR.

Legend: All statistical tests were two-sided. The p-values were not adjusted for multiple testing, therefore p-value<0.05 was considered significant (and reported in bolded text).

Univariable Mendelian randomization of household income levels and mental illnesses

Supplementary Table 21: results of univariable bidirectional Mendelian randomization of Low Household income (LHI) against mental illnesses

MR	N SNP	IVW, B (95% CI)	IVW p-value	IVW Q(df)	Q p-value†	WM, B (95% CI)	WM p-value	MR-Egger, B (95% CI)	MR-Egger p-value	Egger intercept p-value	Steiger Test p-value‡	MR-PRESSO	Mean F
Fw: LHI on ADHD	8	0.610 (0.415; 0.805)	**8.25× 10^{-10}**	8 (7)	0.338	0.569 (0.286; 0.853)	**1.98× 10^{-4}**	0.695 (-0.288; 1.68)	0.215	0.867	**1.41×10^{-9}**	GT; p=0.364	33.5

Bw: ADHD on LHI	23	0.210 (0.170; 0.250)	**8.16×10^{-25}**	44 (22)	**0.004**	0.203 (0.136; 0.270)	**2.96×10^{-9}**	-0.042 (-0.353; 0.268)	0.792	0.120	**2.24×10^{-64}**	GT; p=0.444	39.2
Fw: LHI on AN	11	-0.092 (-0.333; 0.148)	0.452	16 (10)	0.106	0.018 (-0.329; 0.365)	0.919	-0.094 (-1.58; 1.39)	0.904	0.998	NR [b]	GT; p=0.102	33.7
Bw: AN on LHI	4	0.026 (-0.049; 0.100)	0.500	7 (3)	0.067	0.016 (-0.073; 0.106)	0.725	-0.519 (-1.52; 0.484)	0.417	0.396	NR [b]	GT; p=0.133	31.9
Fw: LHI on ANX	10	0.628 (0.095; 1.16)	**0.021**	6 (9)	0.734	0.700 (0.016; 1.38)	**0.045**	0.300 (-3.08; 3.68)	0.866	0.852	**0.012**	GT; p=0.767	33.6
Bw: ANX on LHI	0	NR [c]	NR [c]	NR [c]	NR [c]	NR [c]	NR [c]	NR [c]	NR [c]	NR [c]	NR [c]	NR [c]	NR [c]
Fw: LHI on ASD	12	0.177 (-0.053; 0.408)	0.132	45 (11)	**5.29×10^{-6}**	0.228 (-0.145; 0.602)	0.231	0.407 (-1.82; 2.63)	0.727	0.840	NR [b]	DT; p=0.575	33.5
Bw: ASD on LHI	0	NR [c]	NR [c]	NR [c]	NR [c]	NR [c]	NR [c]	NR [c]	NR [c]	NR [c]	NR [c]	NR [c]	NR [c]
Fw: LHI on BD	9	0.306 (0.125; 0.487)	**0.001**	13 (8)	0.106	0.159 (-0.108; 0.427)	0.243	-0.545 (-1.42; 0.332)	0.263	0.092	0.462	GT; p=0.141	33.7
Bw: BD on LHI	36	-0.001 (-0.032; 0.032)	0.983	91 (35)	**7.10×10^{-7}**	0.029 (-0.023; 0.081)	0.275	0.125 (-0.158; 0.407)	0.394	0.384	NR [b]	DT; p=0.215	39.2
Fw: LHI on MDD	10	0.351 (0.189; 0.513)	**2.23×10^{-5}**	13 (9)	0.175	0.398 (0.058; 0.558)	**0.016**	0.880 (0.082; 1.68)	**0.006**	0.218	**3.57×10^{-8}**	GT; p=0.207	33.5
Bw: MDD on LHI	0	NR [c]	NR [c]	NR [c]	NR [c]	NR [c]	NR [c]	NR [c]	NR [c]	NR [c]	NR [c]	NR [c]	NR [c]
Fw: LHI on OCD	10	-0.108 (-0.738; 0.522)	0.737	11 (9)	0.306	0.293 (-0.536; 1.12)	0.488	0.593 (-2.60; 3.78)	0.725	0.670	NR [b]	GT; p=0.309	33.5
Bw: OCD on LHI	0	NR [c]	NR [c]	NR [c]	NR [c]	NR [c]	NR [c]	NR [c]	NR [c]	NR [c]	NR [c]	NR [c]	NR [c]
Fw: LHI on PTSD	12	0.506 (0.253; 0.759)	**8.99×10^{-5}**	8 (11)	0.672	0.511 (0.167; 0.855)	**0.004**	0.412 (-0.769; 1.59)	0.510	0.877	**2.51×10^{-15}**	GT; p=0.716	33.5

Bw: PTSD on LHI	0	NR [c]	NR [c]	NR [c]	NR [c]	NR [c]	NR [c]	NR [c]	NR [c]	NR [c]	NR [c]	NR [c]	NR [c]
Fw: LHI on SZ	9	0.648 (0.488; 0.808)	**2.13× 10^{-15}**	58 (8)	**9.92× 10^{-8}**	0.400 (0.102; 0.699)	**0.009**	-0.151 (-2.16; 1.86)	0.887	0.452	0.421	DT; p=0.783	33.7
Bw: SZ on LHI	176	0.082 (0.066; 0.098)	**6.18× 10^{-24}**	439 (175)	**6.98× 10^{-24}**	0.071 (0.045; 0.098)	**3.31× 10^{-8}**	0.115 (0.016; 0.214)	**0.024**	0.511	**$<1^{-1000}$**	DT; p=0.543	45.6

Abbreviations: Fw: forward analysis; Bw: backward analysis; LHI: low household income (<£18,000); ADHD: attention deficit hyperactivity disorder; AN: anorexia nervosa; ANX: anxiety disorder; ASD: autism spectrum disorder; BD: bipolar disorder; MDD: major depressive disorder; OCD: obsessive-compulsive disorder; PTSD: post-traumatic stress disorder; SZ: schizophrenia; MR: mendelian randomization; SNP: single nucleotide polymorphism; IVW: inverse variance weighted (fixed effect); B: effect estimates are log-odds; 95% CI: 95% confidence interval; Q: Cochran's Q measure of heterogeneity; df: degree of freedom; WM: weighted median; DT: distortion test; GT: global test.

Legend:

P-value threshold for SNP selection <5e-8.

The phenotypes are analyzed as binary cases (coded as 1) and controls (coded as 0). Do note that this leads to change in the direction of effect when comparing across some of the traits. LHI = class 1 (i.e., HI<£18,000, cases) vs classes 2,3,4,5 (controls)

[a] The Mendelian randomization pleiotropy residual sum and outlier (MR-PRESSO) test identifies possible bias from horizontal pleiotropy. The test consists of three parts, (1) the MR-PRESSO global test which detects horizontal pleiotropy, (2) the outlier corrected causal estimate which corrects for the detected horizontal pleiotropy and (3) the MR-PRESSO distortion test which estimates if the causal estimate is significantly different (at p<0.05) after adjustment for outliers. We conduct all three stages (with the argument NbDistribution=1000, namely using1000 simulation form the null distribution to compute empirical p-values) and present the outlier adjusted causal estimates (OACE) when both global and distortion tests are significant.

[b] We did not run Steiger Test if none of the MR analysis resulted significant (NR: not reported in the cell).

[c] Not enough SNP to perform MR (NR: not reported in the cell).

[†] Based on chi^2 test.

[‡] Based on t-test.

All statistical tests were two-sided. The p-values were not adjusted for multiple testing, therefore p-value<0.05 was considered significant (and reported in bolded text).

Supplementary Table 22: results of univariable bidirectional Mendelian randomization of Low-Mid Household income (LMHI) against mental illnesses

MR	N SNP	IVW, B (95% CI)	IVW p-value	IVW Q(df)	Q p-value[†]	WM, B (95% CI)	WM p-value	MR-Egger, B (95% CI)	MR-Egger p-value	Egger intercept p-value	Steiger Test p-value[‡]	MR-PRESSO	Mean F
Fw: LMHI on ADHD	15	0.356 (0.198; 0.513)	**9.85×10^{-6}**	57 (14)	**3.45×10^{-7}**	0.161 (-0.075; 0.398)	0.181	-1.00 (-3.51; 1.51)	0.448	0.304	**3.23×10^{-16}**	DT; p=0.115	35.8
Bw: ADHD on LMHI	23	0.174 (0.140; 0.208)	**3.56×10^{-23}**	74 (22)	**1.66×10^{-7}**	0.166 (0.107; 0.226)	**3.90×10^{-8}**	-0.015 (-0.373; 0.343)	0.936	0.304	**1.84×10^{-60}**	DT; p=0.852	39.2
Fw: LMHI on AN	17	-0.309 (-0.526; -0.091)	**0.005**	40 (16)	**0.001**	-0.222 (-0.542; 0.099)	0.176	-1.35 (-4.23; 1.53)	0.373	0.486	**0.001**	DT; p=0.160	35.3
Bw: AN on LMHI	4	0.026 (-0.038; 0.090)	0.426	8 (3)	**0.047**	0.023 (-0.056; 0.102)	0.564	-0.652 (-1.29; -0.014)	0.183	0.171	NR [b]	GT; p=0.103	31.9
Fw: LMHI on ANX	17	0.376 (-0.078; 0.831)	0.105	9 (16)	0.930	0.189 (-0.387; 0.765)	0.520	-0.884 (-4.80; 3.03)	0.664	0.535	NR [b]	GT; p=0.952	35.3
Bw: ANX on LMHI	0	NR [c]	NR [c]	NR [c]	NR [c]	NR [c]	NR [c]	NR [c]	NR [c]	NR [c]	NR [c]	NR [c]	NR [c]
Fw: LMHI on ASD	18	-0.268 (-0.488; -0.047)	**0.017**	55 (17)	**7.54×10^{-6}**	-0.374 (-0.753; 0.005)	0.053	-2.15 (-5.04; 0.746)	0.165	0.217	0.994	DT; p=0.275	33.4
Bw: ASD on LMHI	0	NR [c]	NR [c]	NR [c]	NR [c]	NR [c]	NR [c]	NR [c]	NR [c]	NR [c]	NR [c]	NR [c]	NR [c]
Fw: LMHI on BD	16	-0.099 (-0.251; 0.053)	0.201	95 (15)	**1.84×10^{-13}**	0.067 (-0.352; 0.217)	0.643	-2.40 (-5.36; 0.553)	0.133	0.146	NR [b]	DT; p=0.255	35.6
Bw: BD on LMHI	36	-0.030 (-0.058; -0.003)	**0.032**	138 (35)	**4.45×10^{-14}**	-0.030 (-0.076; 0.017)	0.208	-0.001 (-0.302; 0.301)	0.999	0.844	**1.19×10^{-269}**	DT; p=0.226	39.2
Fw: LMHI on MDD	17	0.224 (0.077; 0.371)	**0.003**	19 (16)	0.281	0.204 (-0.004; 0.411)	0.054	-0.159 (-1.39; 1.07)	0.803	0.547	**4.83×10^{-19}**	GT; p=0.278	33.5
Bw: MDD on LMHI	0	NR [c]	NR [c]	NR [c]	NR [c]	NR [c]	NR [c]	NR [c]	NR [c]	NR [c]	NR [c]	NR [c]	NR [c]

Fw: LMHI on OCD	17	-0.774 (-1.34; -0.212)	**0.007**	11 (16)	0.795	-0.833 (-1.57; -0.097)	**0.027**	-0.350 (-4.75; 4.05)	0.878	0.852	0.249	GT; p=0.781	33.5
Bw: OCD on LMHI	0	NR [c]	NR [c]	NR [c]	NR [c]	NR [c]	NR [c]	NR [c]	NR [c]	NR [c]	NR [c]	NR [c]	NR [c]
Fw: LMHI on PTSD	17	0.283 (0.045; 0.521)	**0.020**	24 (16)	0.087	0.285 (-0.071; 0.640)	0.117	-0.485 (-2.89; 1.92)	0.698	0.537	**2.10×10^{-24}**	GT; p=0.096	35.3
Bw: PTSD on LMHI	0	NR [c]	NR [c]	NR [c]	NR [c]	NR [c]	NR [c]	NR [c]	NR [c]	NR [c]	NR [c]	NR [c]	NR [c]
Fw: LMHI on SZ	16	0.270 (0.138; 0.401)	**5.84×10^{-5}**	122 (15)	**2.81×10^{-18}**	0.117 (-0.132; 0.366)	0.357	-0.273 (-3.42; 2.87)	0.867	0.738	**2.58×10^{-16}**	DT; p=0.361	35.6
Bw: SZ on LMHI	176	0.045 (0.031; 0.059)	**1.84×10^{-10}**	436 (175)	**5.35×10^{-24}**	0.041 (0.018; 0.063)	**6.01×10^{-4}**	0.032 (-0.052; 0.117)	0.455	0.768	**$<1^{-1000}$**	DT; p=0.775	45.6

Abbreviations: Fw: forward analysis; Bw: backward analysis; LMHI: low-mid household income (≤£30,999); ADHD: attention deficit hyperactivity disorder; AN: anorexia nervosa; ANX: anxiety disorder; ASD: autism spectrum disorder; BD: bipolar disorder; MDD: major depressive disorder; OCD: obsessive-compulsive disorder; PTSD: post-traumatic stress disorder; SZ: schizophrenia; MR: mendelian randomization; SNP: single nucleotide polymorphism; IVW: inverse variance weighted (fixed effect); B: effect estimates are log-odds; 95% CI: 95% confidence interval; Q: Cochran's Q measure of heterogeneity; df: degree of freedom; WM: weighted median; DT: distortion test; GT: global test.

Legend:

P-value threshold for SNP selection <5e-8.

The phenotypes are analyzed as binary cases (coded as 1) and controls (coded as 0). Do note that this leads to change in the direction of effect when comparing across some of the traits. LMHI = cases: classes 1 (i.e., HI<£18,000) and 2 (i.e., £18,000≤HI≤£29,000) vs controls: classes 3,4,5.

[a] The Mendelian randomization pleiotropy residual sum and outlier (MR-PRESSO) test identifies possible bias from horizontal pleiotropy. The test consists of three parts, (1) the MR-PRESSO global test which detects horizontal pleiotropy, (2) the outlier corrected causal estimate which corrects for the detected horizontal pleiotropy and (3) the MR-PRESSO distortion test which estimates if the causal estimate is significantly different (at p<0.05) after adjustment for outliers. We conduct all three stages (with the argument NbDistribution=1000, namely using1000 simulation form the null distribution to compute empirical p-values) and present the outlier adjusted causal estimates (OACE) when both global and distortion tests are significant.

[b] We did not run Steiger Test if none of the MR analysis resulted significant (NR: not reported in the cell).

[c] Not enough SNP to perform MR (NR: not reported in the cell).

† Based on chi^2 test.
‡ Based on t-test.
All statistical tests were two-sided. The p-values were not adjusted for multiple testing, therefore p-value<0.05 was considered significant (and reported in bolded text).

Supplementary Table 23: results of univariable bidirectional Mendelian randomization of Mid-High Household income (MHHI) against mental illnesses

MR	N SNP	IVW, B (95% CI)	IVW p-value	IVW Q(df)	Q p-value†	WM, B (95% CI)	WM p-value	MR-Egger, B (95% CI)	MR-Egger p-value	Egger intercept p-value	Steiger Test p-value‡	MR-PRESSO	Mean F
Fw: MHHI on ADHD	20	-0.298 (-0.422; -0.174)	**2.65×10^{-6}**	49 (19)	**1.91×10^{-4}**	-0.112 (-0.312; 0.088)	0.272	-0.198 (-1.11; 0.709)	0.674	0.827	**1.27×10^{-26}**	OACE: -0.182 (-0.342; -0.023); p=0.038	36.4
Bw: ADHD on MHHI	23	-0.160 (-0.199; -0.122)	**3.68×10^{-16}**	45 (22)	**0.002**	-0.155 (-0.216; -0.093)	**3.63×10^{-7}**	0.027 (-0.280; 0.333)	0.867	0.238	**3.20×10^{-70}**	DT; p=0.549	39.2
Fw: MHHI on AN	20	0.371 (0.193; 0.550)	**4.59×10^{-5}**	49 (19)	**2.98×10^{-4}**	0.281 (-0.018; 0.581)	0.066	1.05 (-0.224; 2.33)	0.123	0.297	**2.95×10^{-4}**	OACE: 0.181 (-0.058; 0.420); p=0.156	36.6
Bw: AN on MHHI	4	-0.009 (-0.081; 0.062)	0.803	7 (3)	0.066	0.036 (-0.052; 0.124)	0.425	0.314 (-0.811; 1.44)	0.639	0.628	NR [b]	GT; p=0.130	31.9
Fw: MHHI on ANX	20	-0.320 (-0.694; 0.053)	0.093	15 (19)	0.691	-0.098 (-0.583; 0.389)	0.694	-0.951 (-3.27; 1.36)	0.431	0.595	NR [b]	GT; p=0.722	36.4
Bw: ANX on MHHI	0	NR [c]	NR [c]	NR [c]	NR [c]	NR [c]	NR [c]	NR [c]	NR [c]	NR [c]	NR [c]	NR [c]	NR [c]
Fw: MHHI on ASD	21	0.480 (0.303; 0.658)	**1.11×10^{-7}**	36 (20)	**0.015**	0.393 (0.093; 0.692)	**0.010**	0.345 (-0.763; 1.45)	0.549	0.809	0.057	DT; p=0.208	36.2
Bw: ASD on MHHI	0	NR [c]	NR [c]	NR [c]	NR [c]	NR [c]	NR [c]	NR [c]	NR [c]	NR [c]	NR [c]	NR [c]	NR [c]
Fw: MHHI on BD	19	0.179 (0.053; 0.304)	**0.005**	125 (18)	**9.94×10^{-18}**	0.028 (-0.208; 0.264)	0.816	1.69 (0.376; 3.00)	**0.022**	**0.033**	0.187	DT; p=0.092	36.8

Bw: BD on MHHI	36	0.040 (0.009; 0.071)	**0.011**	133 (35)	**2.56×10^{-13}**	0.030 (-0.020; 0.081)	0.239	0.025 (-0.308; 0.357)	0.885	0.927	**2.14×10^{-267}**	OACE: 0.002 (-0.039; 0.042); p=0.943	39.2
Fw: MHHI on MDD	21	-0.123 (-0.238; -0.008)	**0.036**	34 (20)	**0.025**	-0.070 (-0.245; 0.105)	0.433	0.474 (-0.178; 1.13)	0.171	0.082	**3.29×10^{-23}**	DT; p=0.256	36.2
Bw: MDD on MHHI	0	NR [c]	NR [c]	NR [c]	NR [c]	NR [c]	NR [c]	NR [c]	NR [c]	NR [c]	NR [c]	NR [c]	NR [c]
Fw: MHHI on OCD	21	-0.079 (-0.511; 0.353)	0.720	23 (20)	0.265	-0.065 (-0.679; 0.549)	0.836	-0.399 (-2.63; 1.83)	0.729	0.776	NR [b]	GT; p=0.271	36.2
Bw: OCD on MHHI	0	NR [c]	NR [c]	NR [c]	NR [c]	NR [c]	NR [c]	NR [c]	NR [c]	NR [c]	NR [c]	NR [c]	NR [c]
Fw: MHHI on PTSD	21	-0.233 (-0.421; -0.044)	**0.016**	25 (20)	0.184	-0.195 (-0.471; 0.080)	0.165	-0.080 (-1.06; 0.900)	0.874	0.758	**1.75×10^{-30}**	GT; p=0.197	36.2
Bw: PTSD on MHHI	0	NR [c]	NR [c]	NR [c]	NR [c]	NR [c]	NR [c]	NR [c]	NR [c]	NR [c]	NR [c]	NR [c]	NR [c]
Fw: MHHI on SZ	20	-0.098 (-0.201; 0.005)	0.063	143 (19)	**7.85×10^{-21}**	-0.171 (-0.376; 0.033)	0.100	-0.236 (-1.60; 1.12)	0.738	0.841	NR [b]	DT; p=0.574	36.4
Bw: SZ on MHHI	176	-0.046 (-0.061; -0.030)	**6.73×10^{-9}**	432 (175)	**1.53×10^{-23}**	-0.039 (-0.066; -0.012)	**0.005**	0.018 (-0.077; 0.112)	0.707	0.172	**$<1^{-1000}$**	DT; p=0.431	45.6

Abbreviations: Fw: forward analysis; Bw: backward analysis; MHHI: mid-high household income (≥ £52,000); ADHD: attention deficit hyperactivity disorder; AN: anorexia nervosa; ANX: anxiety disorder; ASD: autism spectrum disorder; BD: bipolar disorder; MDD: major depressive disorder; OCD: obsessive-compulsive disorder; PTSD: post-traumatic stress disorder; SZ: schizophrenia; MR: mendelian randomization; SNP: single nucleotide polymorphism; IVW: inverse variance weighted (fixed effect); B: effect estimates are log-odds; 95% CI: 95% confidence interval; Q: Cochran's Q measure of heterogeneity; df: degree of freedom; WM: weighted median; DT: distortion test; GT: global test.

Legend:

P-value threshold for SNP selection <5e-8.

The phenotypes are analyzed as binary cases (coded as 1) and controls (coded as 0). Do note that this leads to change in the direction of effect when comparing across some of the traits. MHHI = controls: classes 1, 2, and 3 vs cases: classes 4 (i.e., £52,000 ≤HI≤£100,000) and 5 (i.e., HI>£100,000)

[a] The Mendelian randomization pleiotropy residual sum and outlier (MR-PRESSO) test identifies possible bias from horizontal pleiotropy. The test consists of three parts, (1) the MR-PRESSO global test which detects horizontal pleiotropy, (2) the outlier corrected causal estimate which corrects for the detected horizontal pleiotropy and (3) the MR-PRESSO distortion test which estimates if the causal estimate is significantly different (at $p<0.05$) after adjustment for outliers. We conduct all three stages (with the argument NbDistribution=1000, namely using1000 simulation form the null distribution to compute empirical p-values) and present the outlier adjusted causal estimates (OACE) when both global and distortion tests are significant.
[b] We did not run Steiger Test if none of the MR analysis resulted significant (NR: not reported in the cell).
[c] Not enough SNP to perform MR (NR: not reported in the cell).
† Based on chi^2 test.
‡ Based on t-test.
All statistical tests were two-sided. The p-values were not adjusted for multiple testing, therefore p-value<0.05 was considered significant (and reported in bolded text).

Supplementary Table 24: results of univariable bidirectional Mendelian randomization of High Household income (HHI) against mental illness

MR	N SNP	IVW, B (95% CI)	IVW p-value	IVW Q(df)	Q p-value†	WM, B (95% CI)	WM p-value	MR-Egger, B (95% CI)	MR-Egger p-value	Egger intercept p-value	Steiger Test p-value‡	MR-PRESSO	Mean F
Fw: HHI on ADHD	2	-0.033 (-0.216; 0.149)	0.720	0.4 (2)	0.513	NR [c]	NR [c]	NR [c]	NR [c]	NR [c]	NR [b]	NR [c]	45.1
Bw: ADHD on HHI	23	-0.193 (-0.266; -0.120)	**1.87×10^{-7}**	42 (22)	**0.007**	-0.211 (-0.322; -0.100)	**2.26×10^{-4}**	0.085 (-0.464; 0.633)	0.765	0.324	**1.81×10^{-85}**	DT; p=0.982	39.2
Fw: HHI on AN	2	0.333 (0.065; 0.600)	**0.015**	2 (1)	0.195	NR [c]	NR [c]	NR [c]	NR [c]	NR [c]	0.183	NR [c]	45.1
Bw: AN on HHI	4	-0.043 (-0.177; 0.092)	0.535	2 (3)	0.544	-0.036 (-0.196; 0.125)	0.663	-0.113 (-1.36; 1.13)	0.875	0.921	NR [b]	GT; p=0.581	31.9
Fw: HHI on ANX	2	-0.101 (-0.641; 0.440)	0.715	0.004 (1)	0.949	NR [c]	NR [c]	NR [c]	NR [c]	NR [c]	NR [b]	NR [c]	45.1
Bw: ANX on HHI	0	NR [c]	NR [c]	NR [c]	NR [c]	NR [c]	NR [c]	NR [c]	NR [c]	NR [c]	NR [c]	NR [c]	NR [c]

Fw: HHI on ASD	3	0.094 (-0.121; 0.308)	0.392	40 (2)	**1.85×10^{-9}**	0.509 (0.163; 0.855)	**0.004**	-4.07 (-6.29; 1.85)	0.173	0.167	**0.031**	NR [c]	46.8
Bw: ASD on HHI	0	NR [c]	NR [c]	NR [c]	NR [c]	NR [c]	NR [c]	NR [c]	NR [c]	NR [c]	NR [c]	NR [c]	NR [c]
Fw: HHI on BD	2	0.608 (0.423; 0.792)	**1.01×10^{-10}**	1 (1)	0.283	NR [c]	NR [c]	NR [c]	NR [c]	NR [c]	**0.015**	NR [c]	45.1
Bw: BD on HHI	36	0.076 (0.017; 0.134)	**0.011**	87 (35)	**2.65×10^{-6}**	0.066 (-0.026; 0.159)	0.158	0.065 (-0.443; 0.573)	0.803	0.968	**1.53×10^{-282}**	DT; p=0.117	39.2
Fw: HHI on MDD	2	0.013 (-0.163; 0.188)	0.889	2 (1)	0.219	NR [c]	NR [c]	NR [c]	NR [c]	NR [c]	NR [c]	NR [c]	45.1
Bw: MDD on HHI	0	NR [c]	NR [c]	NR [c]	NR [c]	NR [c]	NR [c]	NR [c]	NR [c]	NR [c]	NR [c]	NR [c]	NR [c]
Fw: HHI on OCD	2	0.199 (-0.469; 0.866)	0.560	2 (1)	0.180	NR [c]	NR [c]	NR [c]	NR [c]	NR [c]	NR [c]	NR [c]	45.1
Bw: OCD on HHI	0	NR [c]	NR [c]	NR [c]	NR [c]	NR [c]	NR [c]	NR [c]	NR [c]	NR [c]	NR [c]	NR [c]	NR [c]
Fw: HHI on PTSD	3	-0.190 (-0.425; 0.046)	0.114	1 (2)	0.569	-0.157 (-0.453; 0.139)	0.300	-0.887 (-2.40; 0.626)	0.456	0.528	NR [b]	NR [c]	46.8
Bw: PTSD on HHI	0	NR [c]	NR [c]	NR [c]	NR [c]	NR [c]	NR [c]	NR [c]	NR [c]	NR [c]	NR [c]	NR [c]	NR [c]
Fw: HHI on SZ	2	0.030 (-0.121; 0.182)	0.693	14 (1)	**2.18×10^{-4}**	NR [c]	NR [c]	NR [c]	NR [c]	NR [c]	NR [c]	NR [c]	45.1
Bw: SZ on HHI	176	-0.013 (-0.042; 0.016)	0.395	295 (175)	**3.49×10^{-8}**	0.002 (-0.049; 0.052)	0.950	0.095 (-0.051; 0.242)	0.205	0.138	NR [b]	DT; p=0.195	45.6

Abbreviations: Fw: forward analysis; Bw: backward analysis; HHI: high household income (> £100,000); ADHD: attention deficit hyperactivity disorder; AN: anorexia nervosa; ANX: anxiety disorder; ASD: autism spectrum disorder; BD: bipolar disorder; MDD: major depressive disorder; OCD: obsessive-compulsive disorder; PTSD: post-traumatic stress disorder; SZ: schizophrenia; MR: mendelian randomization; SNP: single

nucleotide polymorphism; IVW: inverse variance weighted (fixed effect); B: effect estimates are log-odds; 95% CI: 95% confidence interval; Q: Cochran's Q measure of heterogeneity; df: degree of freedom; WM: weighted median; DT: distortion test; GT: global test.
Legend:
P-value threshold for SNP selection <5e-8.
The phenotypes are analyzed as binary cases (coded as 1) and controls (coded as 0). Do note that this leads to change in the direction of effect when comparing across some of the traits. HHI = cases: class 5 (i.e., HI>£100,000) vs controls: classes 1,2,3,4.
[a] The Mendelian randomization pleiotropy residual sum and outlier (MR-PRESSO) test identifies possible bias from horizontal pleiotropy. The test consists of three parts, (1) the MR-PRESSO global test which detects horizontal pleiotropy, (2) the outlier corrected causal estimate which corrects for the detected horizontal pleiotropy and (3) the MR-PRESSO distortion test which estimates if the causal estimate is significantly different (at p<0.05) after adjustment for outliers. We conduct all three stages (with the argument NbDistribution=1000, namely using1000 simulation form the null distribution to compute empirical p-values) and present the outlier adjusted causal estimates (OACE) when both global and distortion tests are significant.
[b] We did not run Steiger Test if none of the MR analysis resulted significant (NR: not reported in the cell).
[c] Not enough SNP to perform MR (NR: not reported in the cell).
[†] Based on chi^2 test.
[‡] Based on t-test.
All statistical tests were two-sided. The p-values were not adjusted for multiple testing, therefore p-value<0.05 was considered significant (and reported in bolded text).

Supplementary Table 25: Odds Ratio of univariable bidirectional Mendelian randomization analysis of household income levels against mental illnesses

Forward MR analysis			*Backward analysis*		
**MR: method**	**OR (95% CI)**	**p-value**	**MR: method**	**OR (95% CI)**	**p-value**
LHI → ADHD:			ADHD → LHI:		
IVW	1.84 (1.51; 2.24)	**8.25×10^{-10}**	IVW	1.23 (1.19; 1.28)	**8.16×10^{-25}**
WM	1.77 (1.33; 2.35)	**1.98×10^{-4}**	WM	1.23 (1.15; 1.31)	**2.96×10^{-9}**
MR-Egger	2.00 (0.750; 5.35)	0.215	MR-Egger	0.959 (0.703; 1.31)	0.792
LMHI → ADHD:			ADHD → LMHI:		
IVW	1.43 (1.22; 1.67)	**9.85×10^{-6}**	IVW	1.19 (1.15; 1.23)	**3.56×10^{-23}**
WM	1.18 (0.928; 1.49)	0.181	WM	1.18 (1.11; 1.25)	**3.90×10^{-8}**
MR-Egger	0.367 (0.030; 4.51)	0.448	MR-Egger	0.985 (0.689; 1.41)	0.936
MHHI → ADHD:			ADHD → MHHI:		
IVW	0.742 (0.656; 0.841)	**2.65×10^{-6}**	IVW	0.852 (0.820; 0.885)	**3.68×10^{-16}**
WM	0.894 (0.732; 1.09)	0.272	WM	0.856 (0.805; 0.911)	**3.63×10^{-7}**
MR-Egger	0.820 (0.331; 2.03)	0.674	MR-Egger	1.03 (0.756; 1.40)	0.867
HHI → ADHD:			ADHD → HHI:		
IVW	0.967 (0.806; 1.16)	0.720	IVW	0.824 (0.767; 0.886)	**1.87×10^{-7}**
WM	NR	NR	WM	0.810 (0.724; 0.905)	**2.26×10^{-4}**
MR-Egger	NR	NR	MR-Egger	1.09 (0.629; 1.88)	0.765
LHI → AN:			AN → LHI:		
IVW	0.912 (0.717; 1.16)	0.452	IVW	1.03 (0.952; 1.10)	0.500
WM	1.02 (0.716; 1.45)	0.919	WM	1.02 (0.925; 1.12)	0.725
MR-Egger	0.910 (0.206; 4.03)	0.904	MR-Egger	0.595 (0.218; 1.62)	0.417
LMHI → AN:			AN → LMHI:		
IVW	0.734 (0.591; 0.913)	**0.005**	IVW	1.03 (0.963; 1.09)	0.426
WM	0.801 (0.588; 1.09)	0.176	WM	1.02 (0.944; 1.11)	0.564
MR-Egger	0.259 (0.015; 4.61)	0.373	MR-Egger	0.521 (0.275; 0.986)	0.183
MHHI → AN:			AN → MHHI:		
IVW	1.45 (1.21; 1.73)	**4.59×10^{-5}**	IVW	0.991 (0.923; 1.06)	0.803
WM	1.32 (0.994; 1.77)	0.066	WM	1.04 (0.946; 1.14)	0.425
MR-Egger	2.87 (0.800; 10.3)	0.123	MR-Egger	1.37 (0.444; 4.22)	0.639
HHI → AN:			AN → HHI:		
IVW	1.39 (1.07; 1.82)	**0.015**	IVW	0.958 (0.837; 1.10)	0.535
WM	NR	NR	WM	0.965 (0.826; 1.13)	0.663
MR-Egger	NR	NR	MR-Egger	0.893 (0.258; 3.10)	0.875
LHI → ANX:			ANX → LHI:		
IVW	1.87 (1.10; 3.20)	**0.021**	IVW	NR	NR
WM	2.01 (1.01; 4.00)	**0.045**	WM	NR	NR
MR-Egger	1.35 (0.046; 39.8)	0.866	MR-Egger	NR	NR
LMHI → ANX:			ANX → LMHI:		
IVW	1.46 (0.925; 2.30)	0.105	IVW	NR	NR
WM	1.21 (0.648; 2.25)	0.520	WM	NR	NR
MR-Egger	0.413 (0.008; 20.7)	0.664	MR-Egger	NR	NR
MHHI → ANX:			ANX → MHHI:		
IVW	0.726 (0.499; 1.05)	0.093	IVW	NR	NR
WM	0.907 (0.543; 1.52)	0.694	WM	NR	NR
MR-Egger	0.386 (0.038; 3.91)	0.431	MR-Egger	NR	NR
HHI → ANX:			ANX → HHI:		
IVW	0.904 (0.527; 1.55)	0.715	IVW	NR	NR
WM	NR	NR	WM	NR	NR
MR-Egger	NR	NR	MR-Egger	NR	NR
LHI → ASD:			ASD → LHI:		
IVW	1.19 (0.948; 1.50)	0.132	IVW	NR	NR
WM	1.26 (0.856; 1.85)	0.231	WM	NR	NR
MR-Egger	1.50 (0.162; 13.9)	0.727	MR-Egger	NR	NR
LMHI → ASD:			ASD → LMHI:		
IVW	0.765 (0.614; 0.954)	**0.017**	IVW	NR	NR
WM	0.688 (0.463; 1.02)	0.053	WM	NR	NR
MR-Egger	0.117 (0.006; 2.11)	0.165	MR-Egger	NR	NR
MHHI → ASD:			ASD → MHHI:		
IVW	1.62 (1.35; 1.93)	**1.11×10^{-7}**	IVW	NR	NR
WM	1.48 (1.14; 1.93)	**0.010**	WM	NR	NR
MR-Egger	1.41 (0.466; 4.28)	0.549	MR-Egger	NR	NR
HHI → ASD:			ASD → HHI:		
IVW	1.10 (0.886; 1.36)	0.392	IVW	NR	NR
WM	1.66 (1.17; 2.36)	**0.004**	WM	NR	NR
MR-Egger	0.017 (0.002; 0.157)	0.173	MR-Egger	NR	NR
LHI → BD:			BD → LHI:		
IVW	1.36 (1.13; 1.63)	**0.001**	IVW	1.00 (0.968; 1.03)	0.983
WM	1.17 (0.877; 1.57)	0.243	WM	1.03 (0.975; 1.09)	0.275
MR-Egger	0.580 (0.241; 1.39)	0.263	MR-Egger	1.13 (0.854; 1.50)	0.394

LMHI → BD:			BD → LMHI:		
IVW	0.906 (0.778; 1.05)	0.201	IVW	0.970 (0.944; 0.997)	**0.032**
WM	0.935 (0.714; 1.22)	0.643	WM	0.971 (0.927; 1.02)	0.208
MR-Egger	0.091 (0.005; 1.74)	0.133	MR-Egger	1.00 (0.739; 1.35)	0.999
MHHI → BD:			BD → MHHI:		
IVW	1.20 (1.05; 1.36)	**0.005**	IVW	1.04 (1.01; 1.07)	**0.011**
WM	1.03 (0.818; 1.29)	0.816	WM	1.03 (0.983; 1.08)	0.239
MR-Egger	5.41 (1.46; 20.1)	0.022	MR-Egger	1.02 (0.735; 1.43)	0.885
HHI → BD:			BD → HHI:		
IVW	1.84 (1.53; 2.21)	**1.01×10^{-10}**	IVW	1.08 (1.02; 1.14)	**0.011**
WM	NR	NR	WM	1.07 (0.976; 1.17)	0.158
MR-Egger	NR	NR	MR-Egger	1.07 (0.642; 1.77)	0.803
LHI → MDD:			MDD → LHI:		
IVW	1.42 (1.21; 1.67)	**2.23×10^{-5}**	IVW	NR	NR
WM	1.36 (1.07; 1.73)	**0.016**	WM	NR	NR
MR-Egger	2.41 (1.09; 5.35)	**0.006**	MR-Egger	NR	NR
LMHI → MDD:			MDD → LMHI:		
IVW	1.25 (1.08; 1.45)	**0.003**	IVW	NR	NR
WM	1.23 (0.995; 1.51)	0.054	WM	NR	NR
MR-Egger	0.853 (0.250; 2.91)	0.803	MR-Egger	NR	NR
MHHI → MDD:			MDD → MHHI:		
IVW	0.884 (0.788; 0.992)	**0.036**	IVW	NR	NR
WM	0.932 (0.781; 1.11)	0.433	WM	NR	NR
MR-Egger	1.61 (0.837; 3.08)	0.171	MR-Egger	NR	NR
HHI → MDD:			MDD → HHI:		
IVW	1.01 (0.849; 1.21)	0.889	IVW	NR	NR
WM	NR	NR	WM	NR	NR
MR-Egger	NR	NR	MR-Egger	NR	NR
LHI → OCD:			OCD → LHI:		
IVW	0.897 (0.478; 1.69)	0.737	IVW	NR	NR
WM	1.34 (0.575; 3.13)	0.488	WM	NR	NR
MR-Egger	1.81 (0.075; 43.9)	0.725	MR-Egger	NR	NR
LMHI → OCD:			OCD → LMHI:		
IVW	0.461 (0.263; 0.809)	**0.007**	IVW	NR	NR
WM	0.435 (0.201; 0.938)	**0.027**	WM	NR	NR
MR-Egger	0.705 (0.009; 57.5)	0.878	MR-Egger	NR	NR
MHHI → OCD:			OCD → MHHI:		
IVW	0.924 (0.600; 1.42)	0.720	IVW	NR	NR
WM	0.937 (0.497; 1.77)	0.836	WM	NR	NR
MR-Egger	0.671 (0.072; 6.23)	0.729	MR-Egger	NR	NR
HHI → OCD:			OCD → HHI:		
IVW	1.22 (0.626; 2.38)	0.560	IVW	NR	NR
WM	NR	NR	WM	NR	NR
MR-Egger	NR	NR	MR-Egger	NR	NR
LHI → PTSD:			PTSD → LHI:		
IVW	1.66 (1.29; 2.14)	**8.99×10^{-5}**	IVW	NR	NR
WM	1.67 (1.19; 2.34)	**0.004**	WM	NR	NR
MR-Egger	1.51 (0.463; 4.92)	0.510	MR-Egger	NR	NR
LMHI → PTSD:			PTSD → LMHI:		
IVW	1.33 (1.05; 1.68)	**0.020**	IVW	NR	NR
WM	1.33 (0.934; 1.89)	0.117	WM	NR	NR
MR-Egger	0.616 (0.056; 6.79)	0.537	MR-Egger	NR	NR
MHHI → PTSD:			PTSD → MHHI:		
IVW	0.793 (0.656; 0.957)	**0.016**	IVW	NR	NR
WM	0.823 (0.619; 1.09)	0.165	WM	NR	NR
MR-Egger	0.923 (0.346; 2.46)	0.874	MR-Egger	NR	NR
HHI → PTSD:			PTSD → HHI:		
IVW	0.827 (0.654; 1.05)	0.114	IVW	NR	NR
WM	0.855 (0.642; 1.14)	0.300	WM	NR	NR
MR-Egger	0.412 (0.091; 1.87)	0.456	MR-Egger	NR	NR
LHI → SZ:			SZ → LHI:		
IVW	1.91 (1.63; 2.24)	**2.13×10^{-15}**	IVW	1.09 (1.07; 1.10)	**6.18×10^{-24}**
WM	1.49 (1.11; 2.00)	**0.009**	WM	1.07 (1.05; 1.10)	**3.31×10^{-8}**
MR-Egger	0.860 (0.115; 6.44)	0.887	MR-Egger	1.12 (1.02; 1.24)	**0.024**
LMHI → SZ:			SZ → LMHI:		
IVW	1.31 (1.15; 1.49)	**5.84×10^{-5}**	IVW	1.05 (1.03; 1.06)	**1.84×10^{-10}**
WM	1.12 (0.877; 1.44)	0.357	WM	1.04 (1.02; 1.07)	**6.01×10^{-4}**
MR-Egger	0.761 (0.033; 17.6)	0.867	MR-Egger	1.03 (0.949; 1.12)	0.455
MHHI → SZ:			SZ → MHHI:		
IVW	0.907 (0.818; 1.01)	0.063	IVW	0.955 (0.941; 0.970)	**6.73×10^{-9}**
WM	0.843 (0.687; 1.03)	0.100	WM	0.962 (0.936; 0.989)	**0.005**
MR-Egger	0.790 (0.202; 3.08)	0.738	MR-Egger	1.02 (0.927; 1.12)	0.707
HHI → SZ:			SZ → HHI:		

IVW	1.03 (0.886; 1.20)	0.693	IVW	0.987 (0.959; 1.02)	0.395
WM	NR	NR	WM	1.00 (0.956; 1.05)	0.950
MR-Egger	NR	NR	MR-Egger	1.10 (0.950; 1.27)	0.205

Abbreviations: MR: Mendelian randomization; OR: Odds Ratio; 95% CI: 95% confidence intervals; HI: household income; ADHD: attention deficit hyperactivity disorder; AN: anorexia nervosa; ANX: anxiety disorder; ASD: autism spectrum disorders; BD: bipolar disorder; MDD: major depressive disorder; OCD: obsessive-compulsive disorder; PTSD: post-traumatic stress disorder; SZ: schizophrenia; IVW: inverse variance weighted (fixed effect); WM: weighted median; NR: not reported because not enough SNP to perform MR.
Legend: LHI: low household income, cases were those less than £18,000; LMHI: low-mid HI, cases were those less than £29,999; MHHI: mid-high HI, cases were those more than £52,000; HHI: high HI, cases were those more than £100,000.
All statistical tests were two-sided. The p-values were not adjusted for multiple testing, therefore p-value<0.05 was considered significant (and reported in bolded text).

Supplementary Table 26: Results of univariable bidirectional Mendelian randomization analysis of household income levels against mental illnesses, after Steiger filtering

MR	N SNP	IVW, B (95% CI)	IVW p-value	WM, B (95% CI)	WM p-value	MR-Egger, B (95% CI)	MR-Egger p-value	Egger intercept p-value	Mean F
LHI on ADHD	8	0.610 (0.415; 0.805)	**8.25×10^{-10}**	0.569 (0.278; 0.861)	**1.17×10^{-4}**	0.695 (-0.288; 1.68)	0.215	0.744	33.5
LMHI on ADHD	14	0.238 (0.075; 0.401)	**0.004**	0.156 (-0.088; 0.399)	0.209	-1.62 (-3.18; 0.057)	0.065	0.170	36.0
MHHI on ADHD	19	-0.246 (-0.373; -0.118)	**1.57×10^{-4}**	-0.111 (-0.311; 0.090)	0.279	0.375 (-0.476; 1.23)	0.400	0.428	36.5
HHI on ADHD	2	-0.033 (-0.216; 0.149)	0.720	NR	NR	NR	NR	NR	45.1
LHI on AN	10	0.005 (-0.247; 0.256)	0.972	0.028 (-0.310; 0.365)	0.871	0.217 (-0.970; 1.40)	0.729	0.807	34.0
LMHI on AN	15	-0.096 (-0.330; 0.138)	0.421	-0.078 (-0.401; 0.244)	0.634	-1.34 (-3.15; 0.468)	0.170	0.130	34.9
MHHI on AN	17	0.137 (-0.062; 0.336)	0.178	0.212 (-0.072; 0.496)	0.143	0.429 (-0.624; 1.48)	0.437	0.921	34.8
HHI on AN	2	0.333 (0.065; 0.600)	**0.015**	NR	NR	NR	NR	NR	45.1
LHI on ANX	9	0.509 (-0.061; 1.08)	0.080	0.677 (-0.070; 1.42)	0.076	0.437 (-2.96; 3.83)	0.808	0.377	33.5
LMHI on ANX	16	0.285 (-0.184; 0.754)	0.233	0.174 (-0.421; 0.769)	0.566	-0.608 (-4.54; 3.32)	0.766	0.022	35.5
MHHI on ANX	19	-0.286 (-0.662; 0.089)	0.135	-0.096 (-0.604; 0.411)	0.710	-0.400 (-2.79; 1.99)	0.747	0.097	36.8
HHI on ANX	2	-0.101 (-0.641; 0.440)	0.715	NR	NR	NR	NR	NR	45.1
LHI on ASD	8	0.213 (-0.066; 0.492)	0.135	0.274 (-0.127; 0.675)	0.180	-0.739 (-1.90; 0.423)	0.259	0.459	33.8
LMHI on ASD	9	0.003 (-0.311; 0.318)	0.980	-0.110 (-0.522; 0.302)	0.602	-1.10 (-3.49; 1.29)	0.396	0.287	32.7
MHHI on ASD	16	0.240 (0.034; 0.445)	**0.023**	0.136 (-0.160; 0.430)	0.367	0.230 (-0.618; 1.08)	0.603	0.004	35.6
HHI on ASD	0	NR	NR	NR	NR	NR	NR	NR	NR
LHI on BD	6	0.073 (-0.154; 0.300)	0.528	0.068 (-0.210; 0.345)	0.633	-0.146 (-0.995; 0.702)	0.752	0.554	32.5
LMHI on BD	11	-0.001 (-0.190; 0.190)	0.998	-0.004 (-0.286; 0.279)	0.980	0.876 (-1.17; 2.92)	0.424	0.988	32.5
MHHI on BD	14	-0.042 (-0.197; 0.114)	0.598	-0.140 (-0.375; 0.094)	0.241	0.211 (-0.746; 1.17)	0.673	0.247	33.0
HHI on BD	0	NR	NR	NR	NR	NR	NR	NR	NR
LHI on MDD	10	0.351 (0.189; 0.513)	**2.23×10^{-5}**	0.308 (0.072; 0.544)	**0.011**	0.880 (0.082; 1.68)	0.063	0.942	33.5
LMHI on MDD	16	0.224 (0.077; 0.371)	**0.003**	0.204 (-0.005; 0.412)	0.056	-0.026 (-1.35; 1.30)	0.970	0.650	35.6
MHHI on MDD	21	-0.123 (-0.238; -0.008)	**0.036**	-0.070 (-0.240; 0.100)	0.420	0.474 (-0.178; 1.13)	0.171	0.526	36.2
HHI on MDD	2	0.013 (-0.163; 0.188)	0.889	NR	NR	NR	NR	NR	45.1
LHI on OCD	8	0.119 (-0.592; 0.830)	0.743	0.272 (-0.590; 1.13)	0.536	-0.417 (-3.55; 2.72)	0.803	0.064	33.5
LMHI on OCD	9	-0.036 (-0.793; 0.722)	0.927	0.095 (-0.863; 1.05)	0.846	1.60 (-3.87; 7.07)	0.584	0.781	35.9
MHHI on OCD	12	0.072 (-0.478; 0.621)	0.798	0.231 (-0.514; 0.976)	0.544	0.753 (-1.82; 3.33)	0.579	0.726	39.0
HHI on OCD	0	NR	NR	NR	NR	NR	NR	NR	NR

LHI on PTSD	12	0.506 (0.253; 0.759)	**8.99× 10^{-5}**	0.511 (0.167; 0.854)	**0.004**	0.412 (-0.769; 1.59)	0.510	0.949	33.5
LMHI on PTSD	17	0.283 (0.045; 0.521)	**0.002**	0.285 (-0.064; 0.633)	0.109	-0.485 (-2.89; 1.92)	0.698	0.508	35.3
MHHI on PTSD	21	-0.233 (-0.421; -0.044)	**0.016**	-0.195 (-0.475; 0.084)	0.171	-0.080 (-1.06; 0.900)	0.874	0.506	36.2
HHI on PTSD	3	-0.189 (-0.425; 0.046)	0.114	-0.157 (-0.452; 0.139)	0.298	-0.887 (-2.40; 0.626)	0.456	0.695	46.8
LHI on SZ	8	0.491 (0.320; 0.662)	**1.87× 10^{-8}**	0.382 (0.088; 0.675)	**0.011**	0.300 (-1.45; 2.05)	0.749	0.447	33.5
LMHI on SZ	15	0.145 (0.009; 0.282)	**0.037**	0.110 (-0.141; 0.360)	0.391	-0.212 (-2.84; 2.42)	0.877	0.325	35.2
MHHI on SZ	19	-0.023 (-0.129; 0.083)	0.665	-0.170 (-0.380; 0.040)	0.112	-0.331 (-1.53; 0.868)	0.596	0.454	36.3
HHI on SZ	2	0.030 (-0.121; 0.182)	0.693	NR	NR	NR	NR	NR	45.1

Abbreviations: MR: Mendelian randomization; B: effect estimates are log-odds; 95% CI: 95% confidence intervals; HI: household income; ADHD: attention deficit hyperactivity disorder; AN: anorexia nervosa; ANX: anxiety disorder; ASD: autism spectrum disorders; BD: bipolar disorder; MDD: major depressive disorder; OCD: obsessive-compulsive disorder; PTSD: post-traumatic stress disorder; SZ: schizophrenia; IVW: inverse variance weighted (fixed effect); WM: weighted median; NR: not reported because not enough SNP to perform MR.

Legend: LHI: low household income, cases were those less than £18,000; LMHI: low-mid HI, cases were those less than £29,999; MHHI: mid-high HI, cases were those more than £52,000; HHI: high HI, cases were those more than £100,000.

All statistical tests were two-sided. The p-values were not adjusted for multiple testing, therefore p-value<0.05 was considered significant (and reported in bolded text).

Univariable Mendelian randomization of cognitive abilities and mental illnesses

Supplementary Table 27: Odds Ratio of univariable forward Mendelian randomization analysis of cognitive abilities against mental illnesses

MR: method	OR (95% CI)	p-value
CA → ADHD:		
IVW	0.528 (0.487; 0.573)	**1.37×10^{-52}**
WM	0.611 (0.532; 0.702)	**1.78×10^{-11}**
MR-Egger	0.530 (0.282; 0.995)	0.050
CA→ AN:		
IVW	1.36 (1.21; 1.53)	**2.28×10^{-7}**
WM	1.47 (1.22; 1.76)	**4.02×10^{-5}**
MR-Egger	2.65 (1.25; 5.62)	**0.012**
CA → ANX:		
IVW	0.709 (0.561; 0.896)	**0.004**
WM	0.853 (0.604; 1.20)	0.358
MR-Egger	0.765 (0.246; 2.37)	0.643
CA → ASD:		
IVW	1.36 (1.21; 1.53)	**2.09×10^{-7}**
WM	1.32 (1.10; 1.60)	**0.004**
MR-Egger	1.74 (0.760; 3.97)	0.193
CA → BD:		
IVW	1.00 (0.925; 1.09)	0.934
WM	0.887 (0.767; 1.02)	0.102
MR-Egger	1.16 (0.564; 2.38)	0.689
CA → MDD:		
IVW	0.870 (0.807; 0.937)	**2.66×10^{-4}**
WM	0.902 (0.799; 1.02)	0.099
MR-Egger	0.838 (0.520; 1.35)	0.470
CA → OCD:		
IVW	1.31 (0.988; 1.75)	0.061
WM	1.20 (0.775; 1.87)	0.414
MR-Egger	0.903 (0.198; 4.12)	0.896
CA → PTSD:		
IVW	0.870 (0.768; 0.987)	**0.030**
WM	0.813 (0.671; 0.984)	**0.032**
MR-Egger	1.25 (0.633; 2.46)	0.525
CA → SZ:		
IVW	0.743 (0.694; 0.796)	**2.92×10^{-17}**
WM	0.862 (0.748; 0.993)	**0.038**
MR-Egger	0.945 (0.393; 2.27)	0.899

Abbreviations: MR: Mendelian randomization; OR: Odds Ratio; 95% CI: 95% confidence intervals; CA: cognitive abilities; ADHD: attention deficit hyperactivity disorder; AN: anorexia nervosa; ANX: anxiety disorder; ASD: autism spectrum disorders; BD: bipolar disorder; MDD: major depressive disorder; OCD: obsessive-compulsive disorder; PTSD: post-traumatic stress disorder; SZ: schizophrenia; IVW: inverse variance weighted (fixed effect); WM: weighted median; NR: not reported because not enough SNP to perform MR.
Legend: All statistical tests were two-sided. The p-values were not adjusted for multiple testing, therefore p-value<0.05 was considered significant (and reported in bolded text).

Supplementary Table 28: results of bidirectional MR of cognitive abilities against mental illness

**MR**	**N SNP**	**IVW, B (95% CI)**	**IVW p-value**	**IVW Q(df)**	**Q p-value**[†]	**WM, B (95% CI)**	**WM p-value**	**MR-Egger, B (95% CI)**	**MR-Egger p-value**	**Egger intercept p-value**	**Steiger Test p-value**[‡]	**MR-PRESSO**	**Mean F**
Fw: CA on ADHD	131	-0.638 (-0.720; -0.556)	**1.37×10^{-52}**	341 (130)	**7.02×10^{-21}**	-0.493 (-0.632; -0.354)	**1.78×10^{-11}**	-0.635 (-1.27; -0.005)	0.050	0.993	**$<1^{-1000}$**	DT; p=0.714	44.0
Bw: ADHD on CA	23	-0.151 (-0.172; -0.131)	**6.21×10^{-48}**	91 (22)	**2.16×10^{-10}**	-0.106 (-0.142; -0.071)	**1.33×10^{-8}**	-0.010 (-0.236; 0.256)	0.938	0.206	**2.61×10^{-25}**	OACE: -0.122 (-0.155; -0.090); **$p=5.93\times10^{-7}$**	39.2
Fw: CA on AN	137	0.306 (0.190; 0.422)	**2.28×10^{-7}**	291 (136)	**3.76×10^{-13}**	0.384 (0.197; 0.571)	**4.02×10^{-5}**	0.974 (0.223; 1.73)	**0.012**	0.076	**2.21×10^{-114}**	DT; p=0.874	43.9
Bw: AN on CA	3	-0.005 (-0.047; 0.038)	0.826	6 (2)	0.053	-0.008 (-0.072; 0.055)	0.795	-0.007 (-0.931; 0.917)	0.991	0.997	NR [b]	NR [c]	32.3
Fw: CA on ANX	137	-0.344 (-0.578; -0.110)	**0.004**	149 (136)	0.204	-0.159 (-0.499; 0.180)	0.358	-0.268 (-1.40; 0.865)	0.643	0.894	**6.32×10^{-66}**	GT; p=0.210	43.9
Bw: ANX on CA	0	NR [c]	NR [c]	NR [c]	NR [c]	NR [c]	NR [c]	NR [c]	NR [c]	NR [c]	NR [c]	NR [c]	NR [c]
Fw: CA on ASD	138	0.310 (0.193; 0.427)	**2.09×10^{-7}**	338 (137)	**9.51×10^{-19}**	0.280 (0.087; 0.473)	**0.004**	0.552 (-0.274; 1.38)	0.193	0.556	**2.76×10^{-43}**	DT; p=0.553	43.8
Bw: ASD on CA	0	NR [c]	NR [c]	NR [c]	NR [c]	NR [c]	NR [c]	NR [c]	NR [c]	NR [c]	NR [c]	NR [c]	NR [c]
Fw: CA on BD	134	0.003 (-0.078; 0.085)	0.934	491 (133)	**2.06×10^{-42}**	-0.120 (-0.265; 0.024)	0.102	0.147 (-0.573; 0.867)	0.689	0.689	NR [b]	DT; p=0.420	44.0
Bw: BD on CA	36	0.015 (-0.001; 0.032)	0.069	370 (35)	**2.04×10^{-57}**	0.024 (-0.006; 0.054)	0.119	-0.149 (-0.445; 0.147)	0.330	0.276	NR [b]	OACE: -0.003 (-0.030; 0.024); p=0.805	39.2
Fw: CA on MDD	138	-0.140 (-0.215; -0.065)	**2.66×10^{-4}**	279 (137)	**1.06×10^{-11}**	-0.103 (-0.225; 0.019)	0.099	-0.177 (-0.654; 0.301)	0.470	0.876	**2.64×10^{-283}**	DT; p=0.842	43.8

Bw: MDD on CA	0	NR [c]	NR [c]	NR [c]	NR [c]	NR [c]	NR [c]	NR [c]	NR [c]	NR [c]	NR [c]	NR [c]	NR [c]
Fw: CA on OCD	138	0.272 (-0.012; 0.557)	0.061	192 (137)	**0.001**	0.186 (-0.259; 0.630)	0.414	-0.102 (-1.62; 1.41)	0.896	0.621	NR [b]	DT; p=0.848	43.8
Bw: OCD on CA	0	NR [c]	NR [c]	NR [c]	NR [c]	NR [c]	NR [c]	NR [c]	NR [c]	NR [c]	NR [c]	NR [c]	NR [c]
Fw: CA on PTSD	136	-0.139 (-0.264; -0.013)	**0.030**	192 (135)	**0.001**	-0.207 (-0.396; -0.018)	**0.032**	0.220 (-0.458; 0.898)	0.525	0.289	**$<1^{-1000}$**	DT; p=0.172	43.9
Bw: PTSD on CA	0	NR [c]	NR [c]	NR [c]	NR [c]	NR [c]	NR [c]	NR [c]	NR [c]	NR [c]	NR [c]	NR [c]	NR [c]
Fw: CA on SZ	134	-0.297 (-0.365; -0.228)	**2.92×10^{-17}**	986 (133)	**4.05×10^{-128}**	-0.149 (-0.289; -0.008)	**0.038**	-0.057 (-0.933; 0.819)	0.899	0.584	**$<1^{-1000}$**	DT; p=0.863	44.0
Bw: SZ on CA	175	-0.055 (-0.063; -0.047)	**2.23×10^{-39}**	1010 (174)	**6.49×10^{-114}**	-0.046 (-0.062; -0.030)	**9.05×10^{-9}**	-0.092 (-0.170; 0.014)	**0.023**	0.345	**1.86×10^{-286}**	OACE: -0.036 (-0.051; -0.022); **$p=4.87\times 10^{-6}$**	45.6

Abbreviations: Fw: forward analysis; Bw: backward analysis; CA: cognitive abilities; ADHD: attention deficit hyperactivity disorder; AN: anorexia nervosa; ANX: anxiety disorder; ASD: autism spectrum disorder; BD: bipolar disorder; MDD: major depressive disorder; OCD: obsessive-compulsive disorder; PTSD: post-traumatic stress disorder; SZ: schizophrenia; MR: mendelian randomization; SNP: single nucleotide polymorphism; IVW: inverse variance weighted (fixed effect); B: effect estimates are log-odds for binary traits (i.e., for mental illnesses) and unstandardized regression coefficient for continuous traits (i.e., for cognitive abilities); 95% CI: 95% confidence interval; Q: Cochran's Q measure of heterogeneity; df: degree of freedom; WM: weighted median; DT: distortion test; GT: global test.

Legend:

P-value threshold for SNP selection <5e-8.

[a] The Mendelian randomization pleiotropy residual sum and outlier (MR-PRESSO) test identifies possible bias from horizontal pleiotropy. The test consists of three parts, (1) the MR-PRESSO global test which detects horizontal pleiotropy, (2) the outlier corrected causal estimate which corrects for the detected horizontal pleiotropy and (3) the MR-PRESSO distortion test which estimates if the causal estimate is significantly different (at p<0.05) after adjustment for outliers. We conduct all three stages (with the argument NbDistribution=1000, namely using1000 simulation form the null distribution to compute empirical p-values) and present the outlier adjusted causal estimates (OACE) when both global and distortion tests are significant.

[b] We did not run Steiger Test if none of the MR analysis resulted significant (NR: not reported in the cell).
[c] Not enough SNP to perform MR (NR: not reported in the cell).
† Based on chi^2 test.
‡ Based on t-test.
All statistical tests were two-sided. The p-values were not adjusted for multiple testing, therefore p-value<0.05 was considered significant (and reported in bolded text).

<u>Plots - Forward analyses</u>

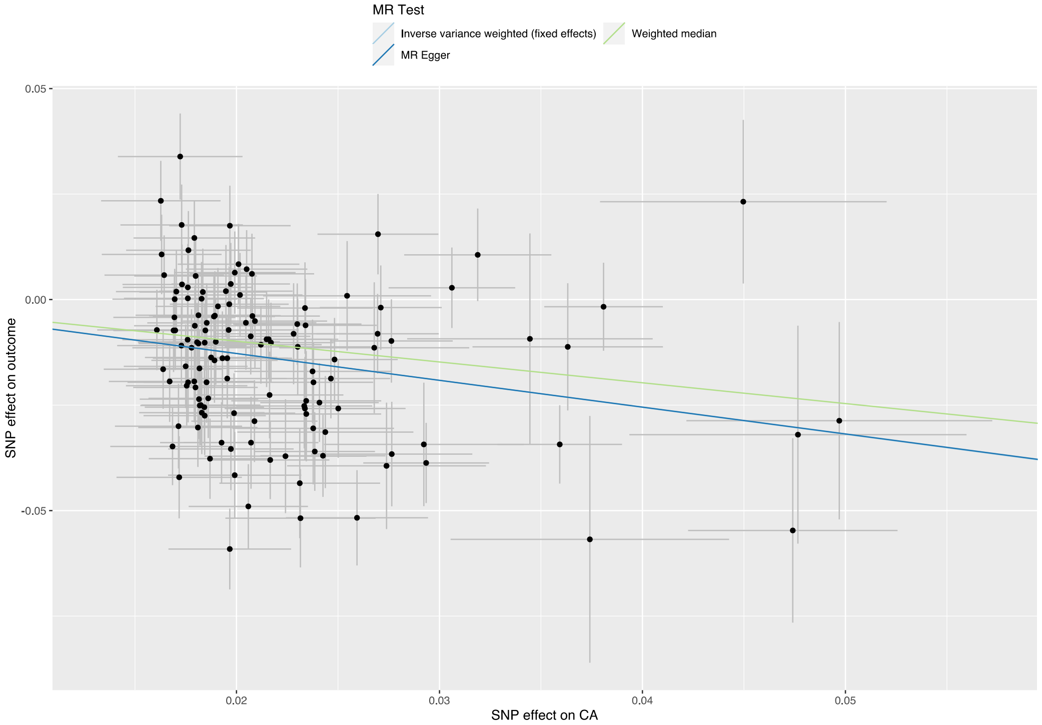

Supplementary Figure 108: scatterplot of cognitive abilities against ADHD
Abbreviations: MR: Mendelian randomization; SNP: single nucleotide polymorphism; CA: cognitive abilities; ADHD: attention deficit hyperactivity disorder.
Legend: n=131 SNPs. Each dot represents a SNP, with the slope of each line corresponding to the estimated MR effect per method.

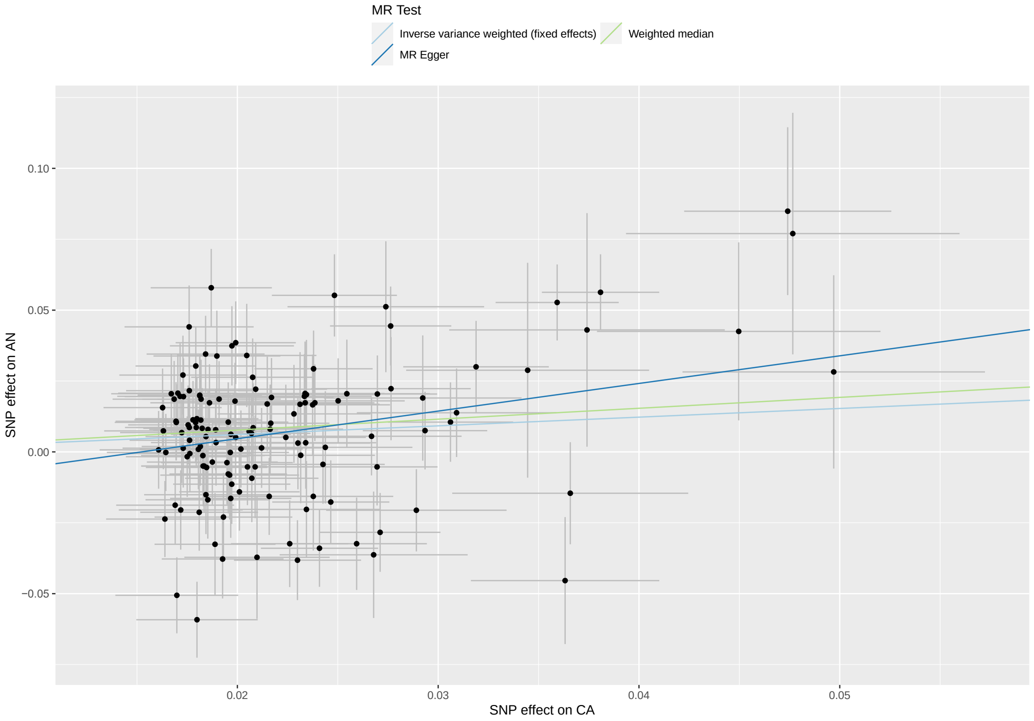

Supplementary Figure 109: scatterplot of cognitive abilities against AN
Abbreviations: MR: Mendelian randomization; SNP: single nucleotide polymorphism; CA: cognitive abilities; AN: anorexia nervosa.
Legend: n=137 SNPs. Each dot represents a SNP, with the slope of each line corresponding to the estimated MR effect per method.

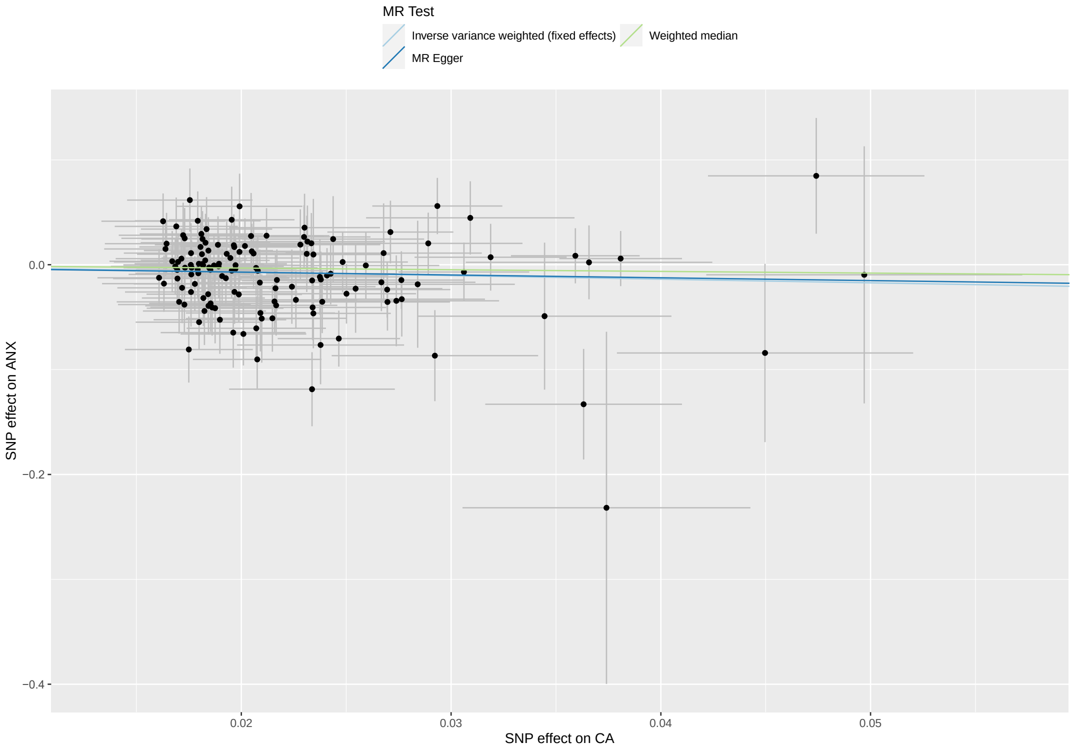

Supplementary Figure 110: scatterplot of cognitive abilities against ANX
Abbreviations: MR: Mendelian randomization; SNP: single nucleotide polymorphism; CA: cognitive abilities; ANX: anxiety disorders.
Legend: n=137 SNPs. Each dot represents a SNP, with the slope of each line corresponding to the estimated MR effect per method.

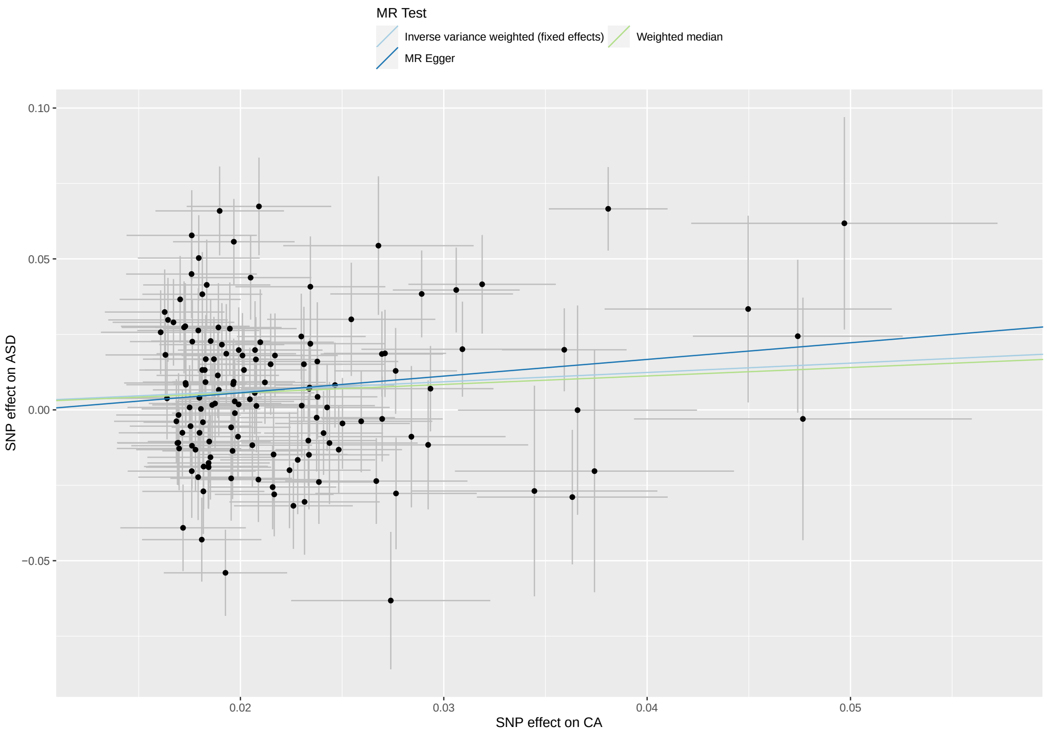

Supplementary Figure 111: scatterplot of cognitive abilities against ASD
Abbreviations: MR: Mendelian randomization; SNP: single nucleotide polymorphism; CA: cognitive abilities; ASD: autism spectrum disorders.
Legend: n=138 SNPs. Each dot represents a SNP, with the slope of each line corresponding to the estimated MR effect per method.

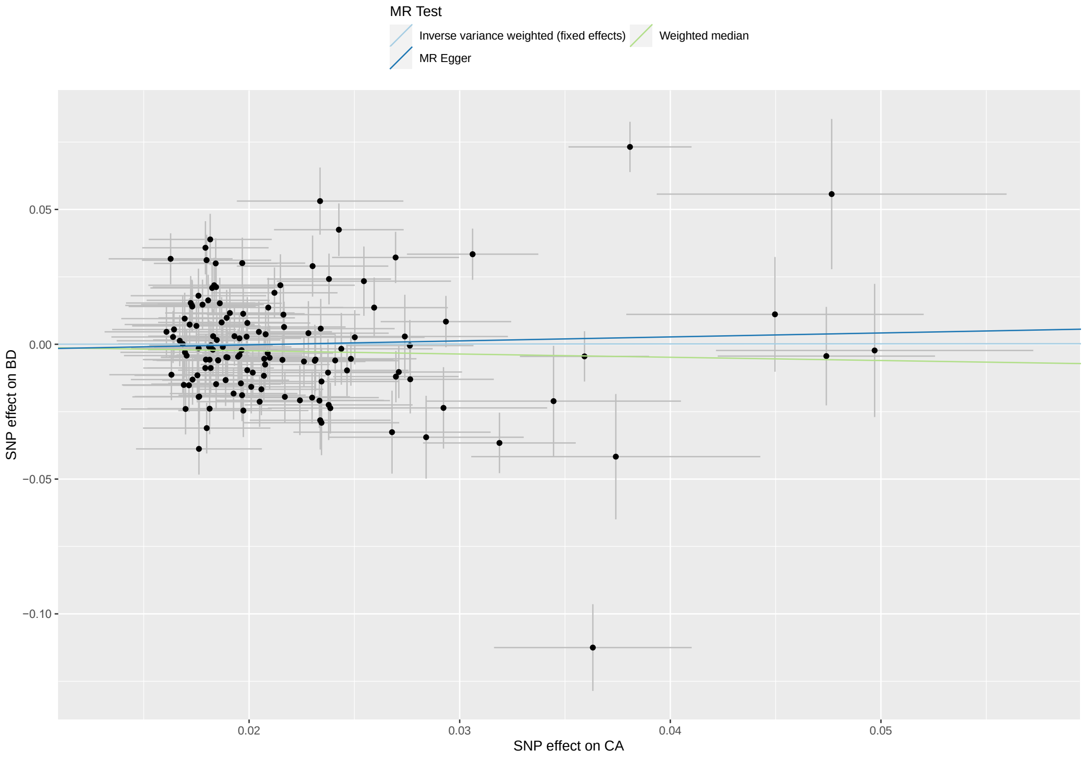

Supplementary Figure 112: scatterplot of cognitive abilities against BD
Abbreviations: MR: Mendelian randomization; SNP: single nucleotide polymorphism; CA: cognitive abilities; BD: bipolar disorder.
Legend: n=134 SNPs. Each dot represents a SNP, with the slope of each line corresponding to the estimated MR effect per method.

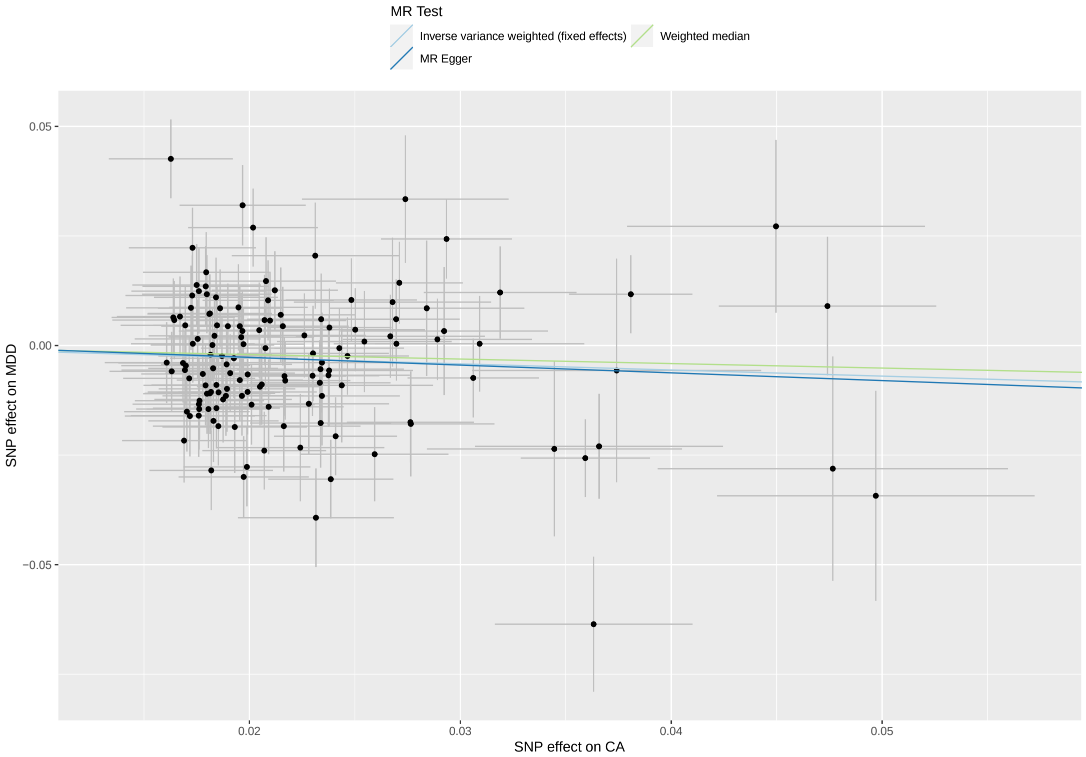

Supplementary Figure 113: scatterplot of cognitive abilities against MDD
Abbreviations: MR: Mendelian randomization; SNP: single nucleotide polymorphism; CA: cognitive abilities; MDD: major depressive disorder.
Legend: n=138 SNPs. Each dot represents a SNP, with the slope of each line corresponding to the estimated MR effect per method.

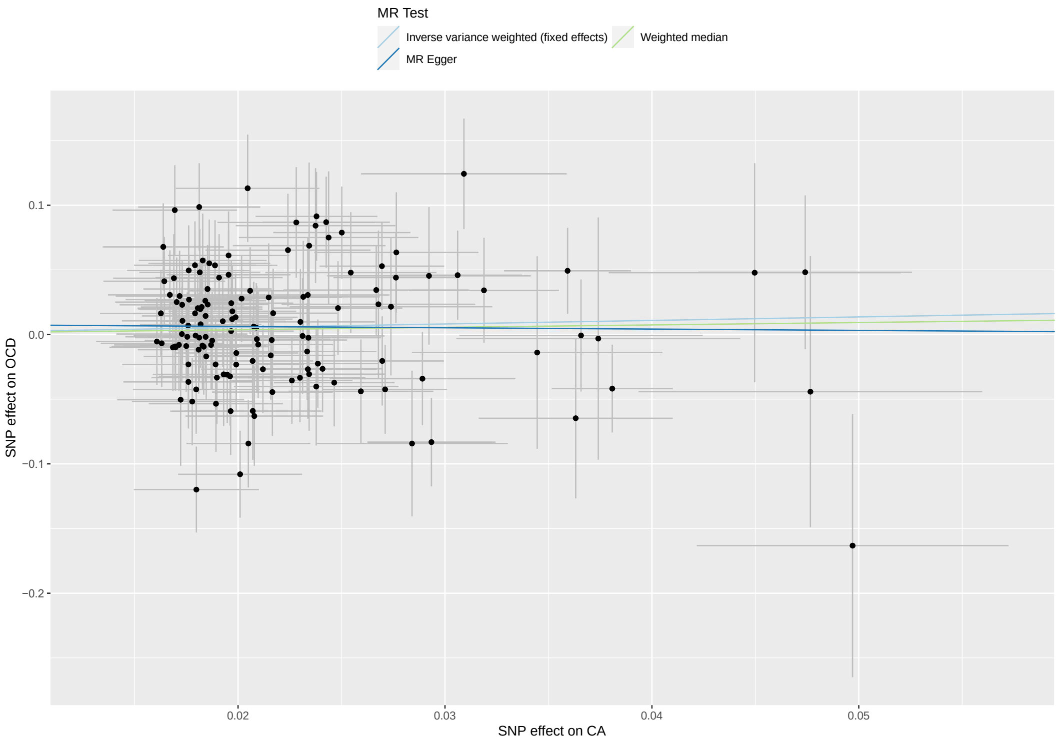

Supplementary Figure 114: scatterplot of cognitive abilities against OCD
Abbreviations: MR: Mendelian randomization; SNP: single nucleotide polymorphism; CA: cognitive abilities; OCD: obsessive-compulsive disorder.
Legend: n=138 SNPs. Each dot represents a SNP, with the slope of each line corresponding to the estimated MR effect per method.

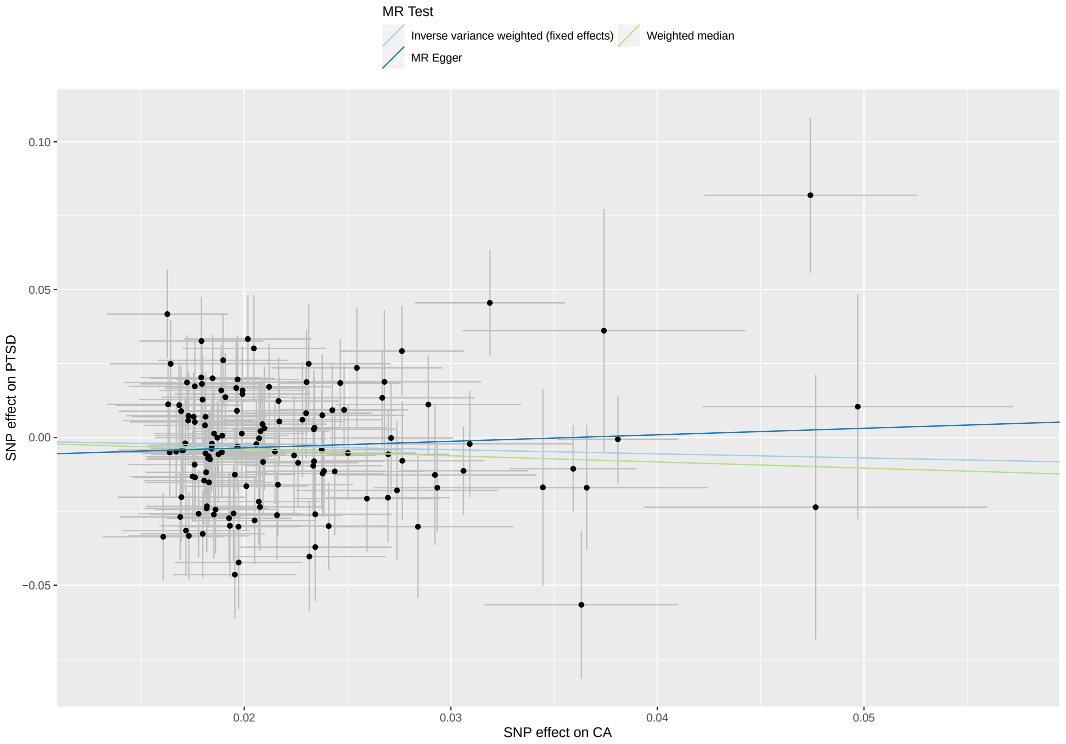

Supplementary Figure 115: scatterplot of cognitive abilities against PTSD
Abbreviations: MR: Mendelian randomization; SNP: single nucleotide polymorphism; CA: cognitive abilities; PTSD: post-traumatic stress disorder.

Legend: n=136 SNPs. Each dot represents a SNP, with the slope of each line corresponding to the estimated MR effect per method.

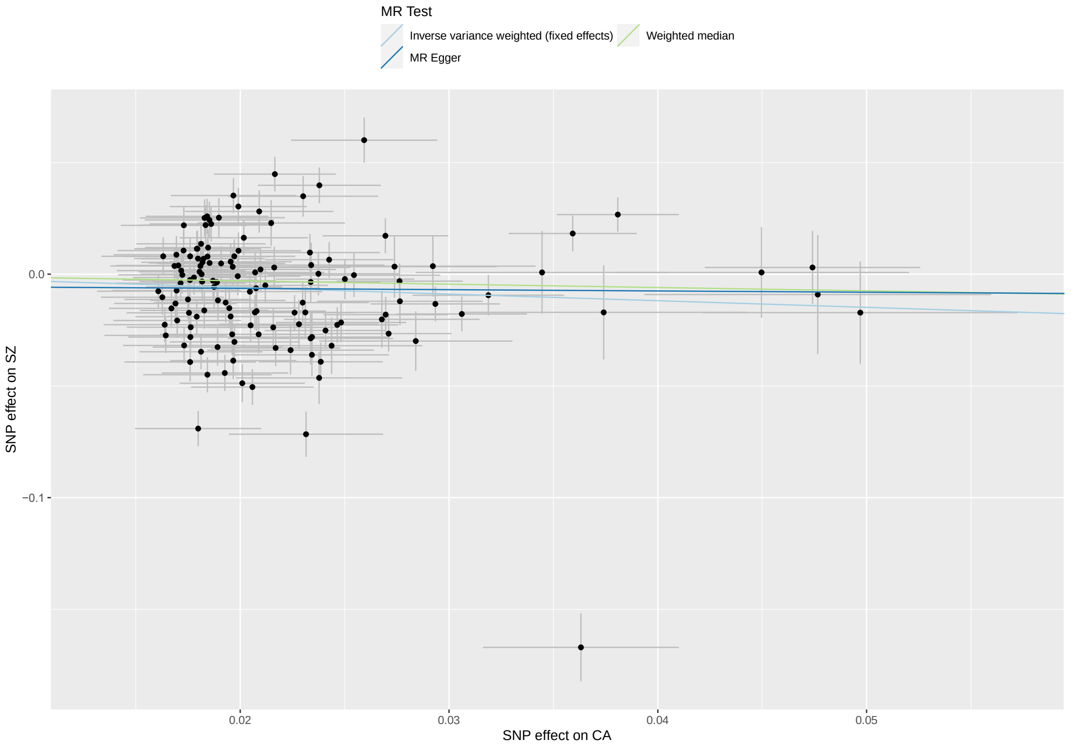

Supplementary Figure 116: scatterplot of cognitive abilities against SZ
Abbreviations: MR: Mendelian randomization; SNP: single nucleotide polymorphism; CA: cognitive abilities; SZ: schizophrenia.
Legend: n=134 SNPs. Each dot represents a SNP, with the slope of each line corresponding to the estimated MR effect per method.

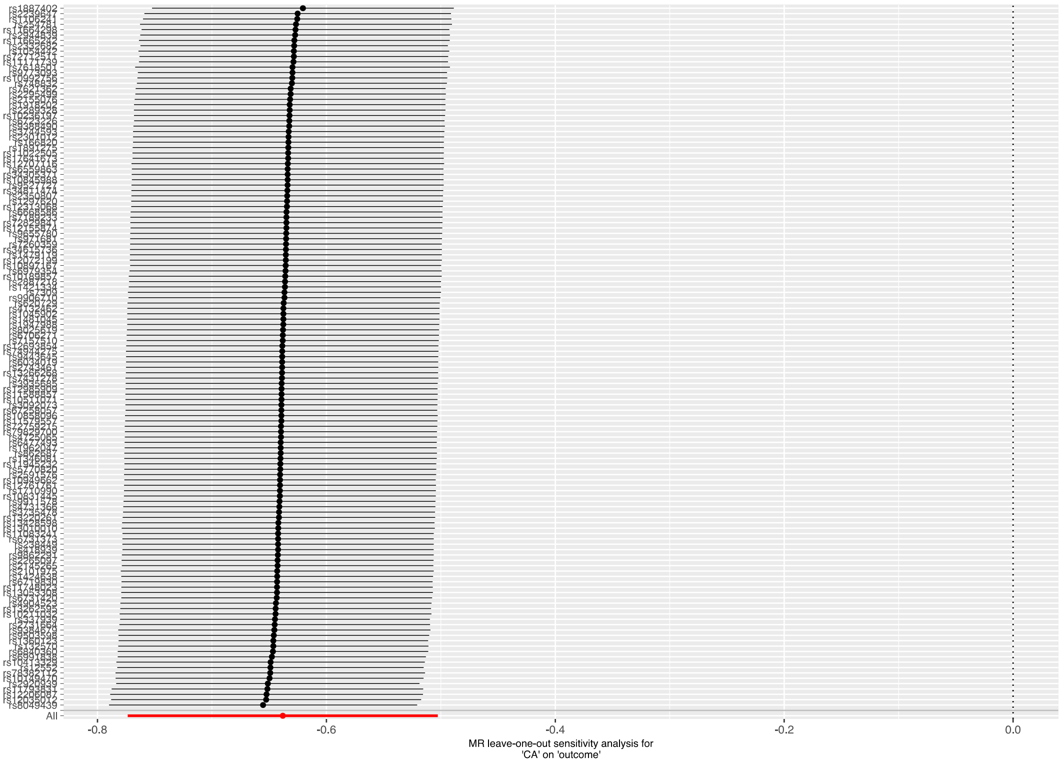

Supplementary Figure 117: leave-one out analysis of cognitive abilities against ADHD
Abbreviations: MR: Mendelian randomization; CA: cognitive abilities; ADHD: attention deficit hyperactivity disorder.
Legend: n=131 SNPs. Forest plot of individual and combined SNP MR estimated effect size. The effect estimates represent the log-odds for ADHD per one standard deviation (1 SD) increase in the mean cognitive ability, and the error bars represent 95% CIs.

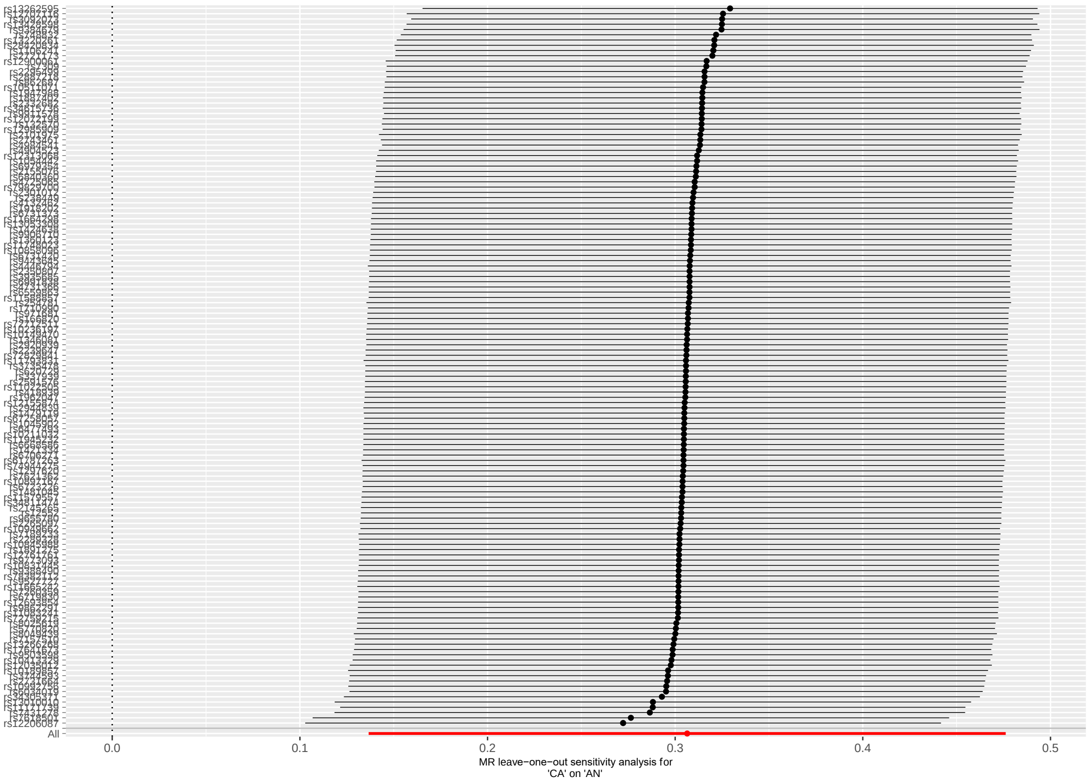

Supplementary Figure 118: leave-one out analysis of cognitive abilities against AN
Abbreviations: MR: Mendelian randomization; CA: cognitive abilities; AN: anorexia nervosa.
Legend: n=137 SNPs. Forest plot of individual and combined SNP MR estimated effect size. The effect estimates represent the log-odds for AN per one standard deviation (1 SD) increase in the mean cognitive ability, and the error bars represent 95% CIs.

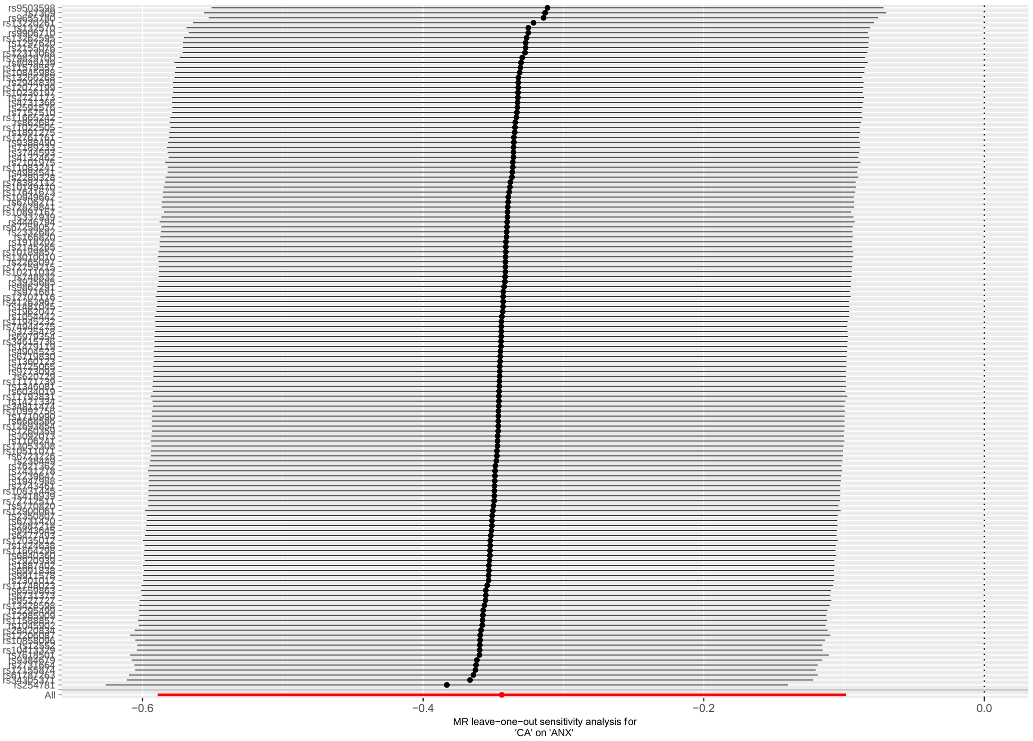

Supplementary Figure 119: leave-one out analysis of cognitive abilities against ANX
Abbreviations: MR: Mendelian randomization; CA: cognitive abilities; ANX: anxiety disorders.
Legend: n=137 SNPs. Forest plot of individual and combined SNP MR estimated effect size. The effect estimates represent the log-odds for ANX per one standard deviation (1 SD) increase in the mean cognitive ability, and the error bars represent 95% CIs.

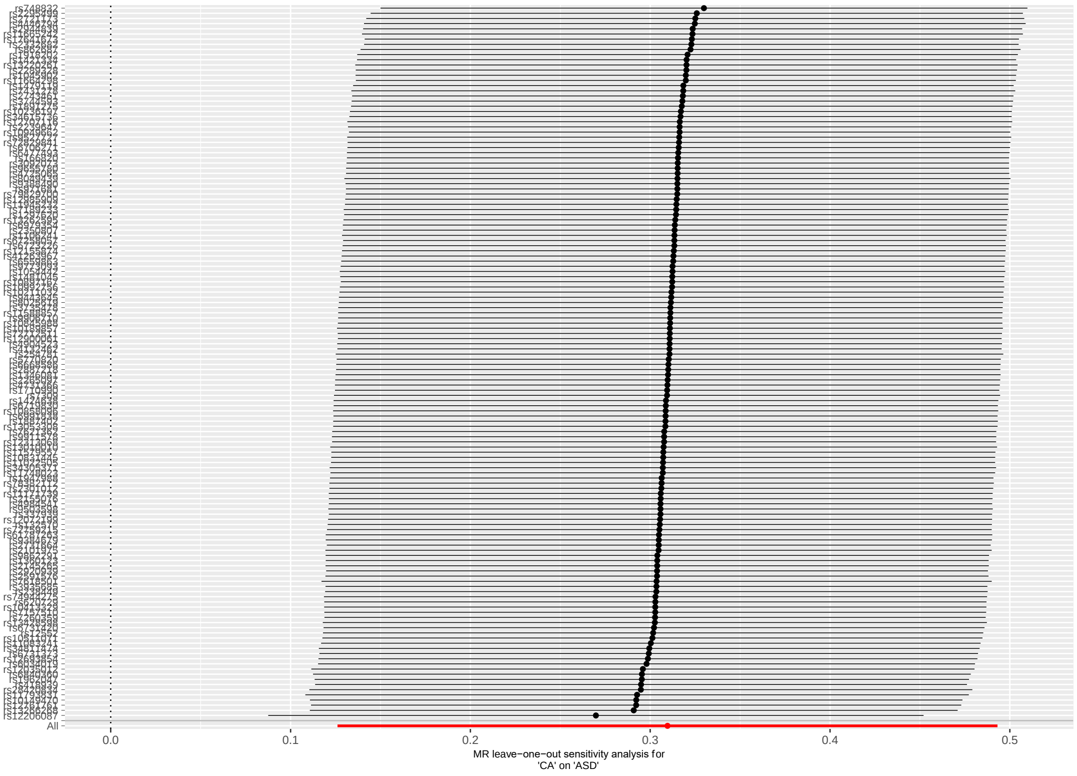

Supplementary Figure 120: leave-one out analysis of cognitive abilities against ASD
Abbreviations: MR: Mendelian randomization; CA: cognitive abilities; ASD: autism spectrum disorders.
Legend: n=138 SNPs. Forest plot of individual and combined SNP MR estimated effect size. The effect estimates represent the log-odds for ASD per one standard deviation (1 SD) increase in the mean cognitive ability, and the error bars represent 95% CIs.

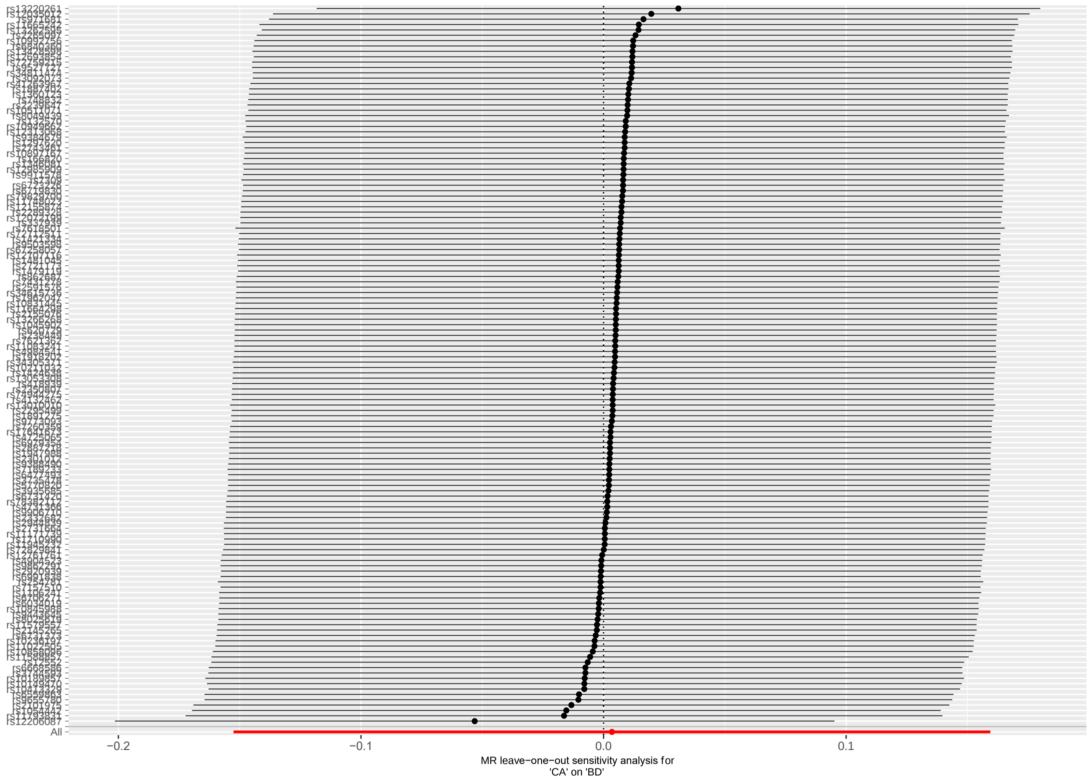

Supplementary Figure 121: leave-one out analysis of cognitive abilities against BD
Abbreviations: MR: Mendelian randomization; CA: cognitive abilities; BD: bipolar disorder.
Legend: n=134 SNPs. Forest plot of individual and combined SNP MR estimated effect size. The effect estimates represent the log-odds for BD per one standard deviation (1 SD) increase in the mean cognitive ability, and the error bars represent 95% CIs.

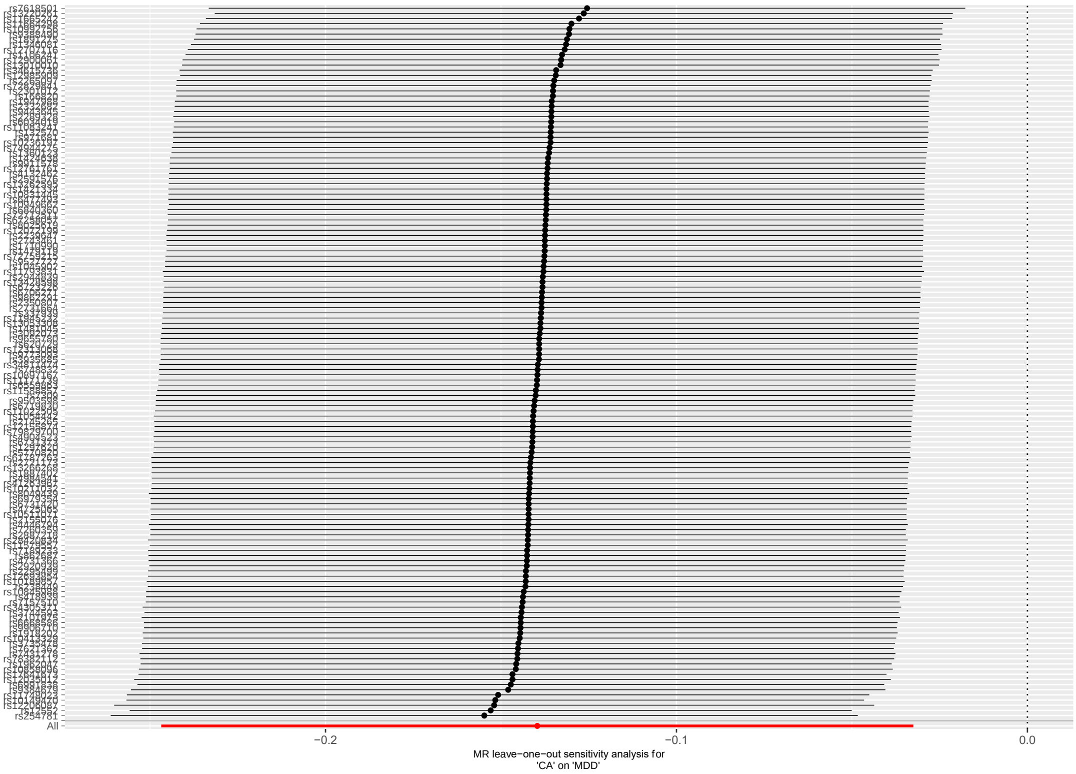

Supplementary Figure 122: leave-one-out analysis of cognitive abilities against MDD
Abbreviations: MR: Mendelian randomization; CA: cognitive abilities; MDD: major depressive disorder.
Legend: n=138 SNPs. Forest plot of individual and combined SNP MR estimated effect size. The effect estimates represent the log-odds for MDD per one standard deviation (1 SD) increase in the mean cognitive ability, and the error bars represent 95% CIs.

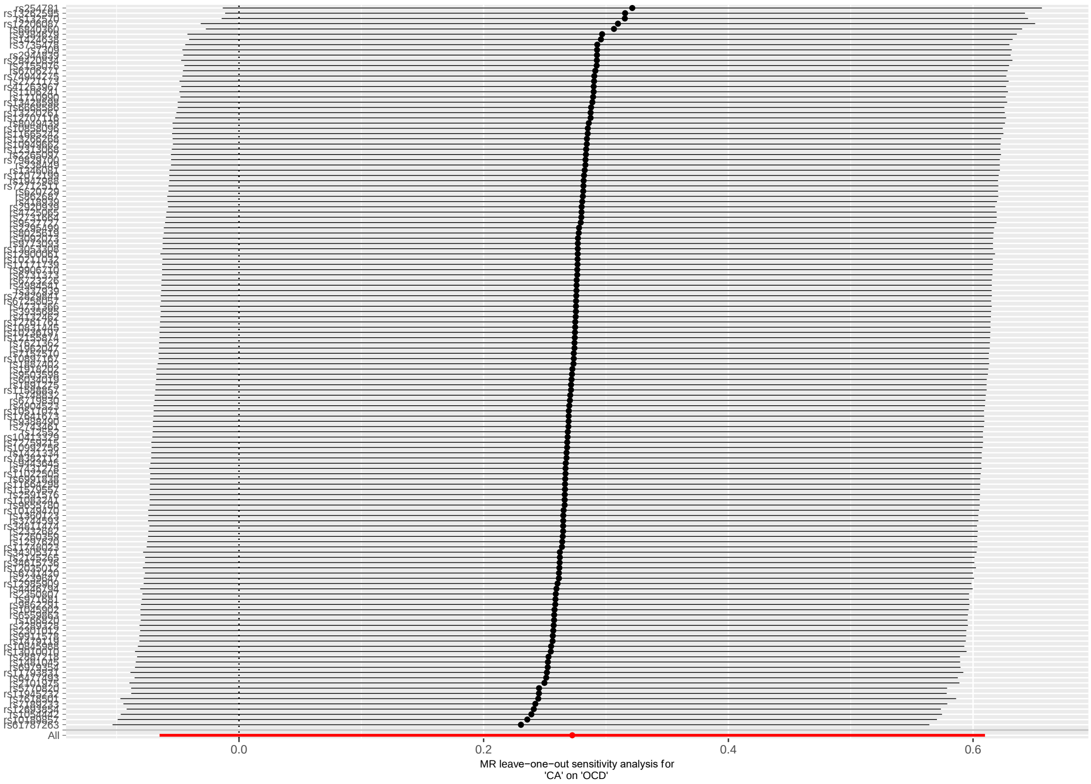

Supplementary Figure 123: leave-one out analysis of cognitive abilities against OCD
Abbreviations: MR: Mendelian randomization; CA: cognitive abilities; OCD: obsessive-compulsive disorder.
Legend: n=138 SNPs. Forest plot of individual and combined SNP MR estimated effect size. The effect estimates represent the log-odds for OCD per one standard deviation (1 SD) increase in the mean cognitive ability, and the error bars represent 95% CIs.

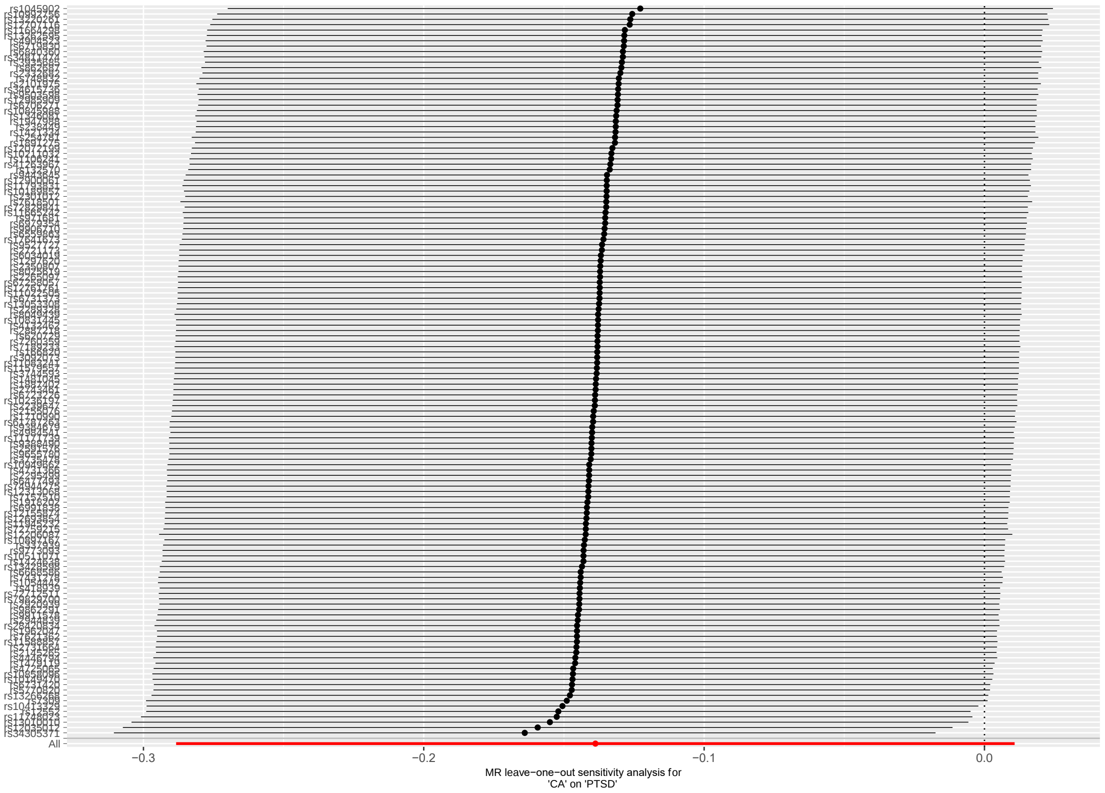

Supplementary Figure 124: leave-one out analysis of cognitive abilities against PTSD
Abbreviations: MR: Mendelian randomization; CA: cognitive abilities; PTSD: post-traumatic stress disorder.
Legend: n=136 SNPs. Forest plot of individual and combined SNP MR estimated effect size. The effect estimates represent the log-odds for PTSD per one standard deviation (1 SD) increase in the mean cognitive ability, and the error bars represent 95% CIs.

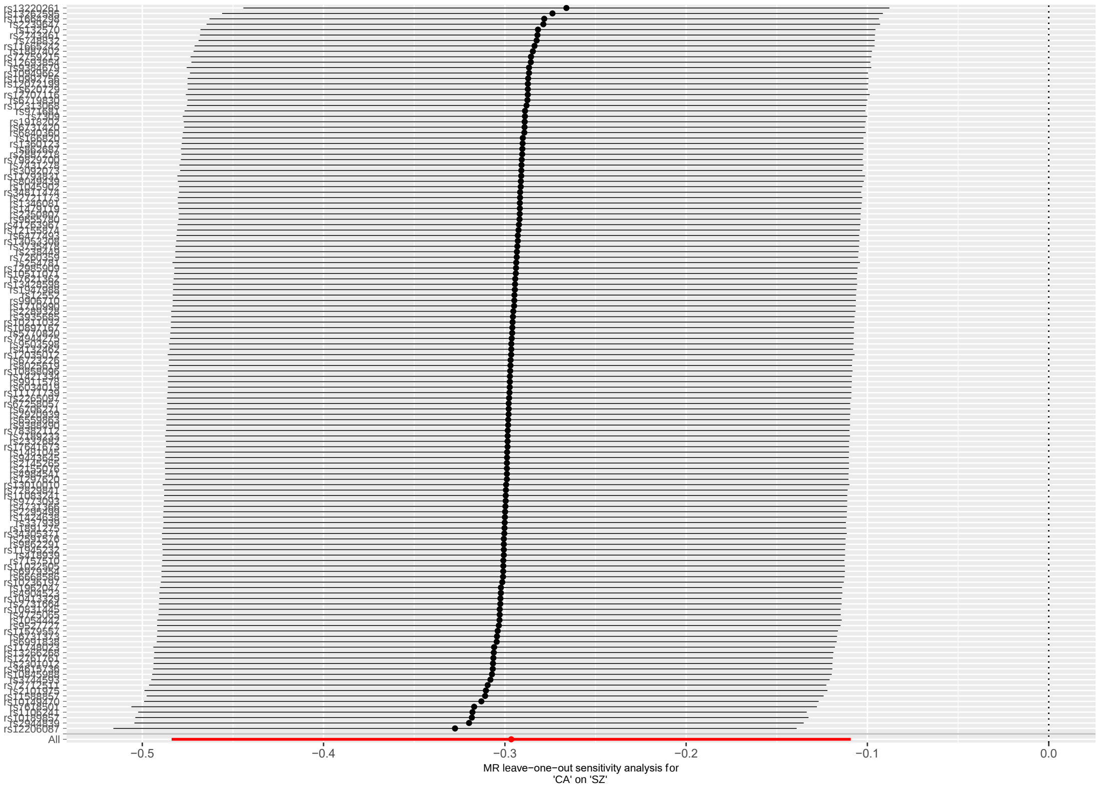

Supplementary Figure 125: leave-one-out analysis of cognitive abilities against SZ
Abbreviations: MR: Mendelian randomization; CA: cognitive abilities; SZ: schizophrenia.
Legend: n=134 SNPs. Forest plot of individual and combined SNP MR estimated effect size. The effect estimates represent the log-odds for SZ per one standard deviation (1 SD) increase in the mean cognitive ability, and the error bars represent 95% CIs.

<u>Plots - Backward analyses</u>

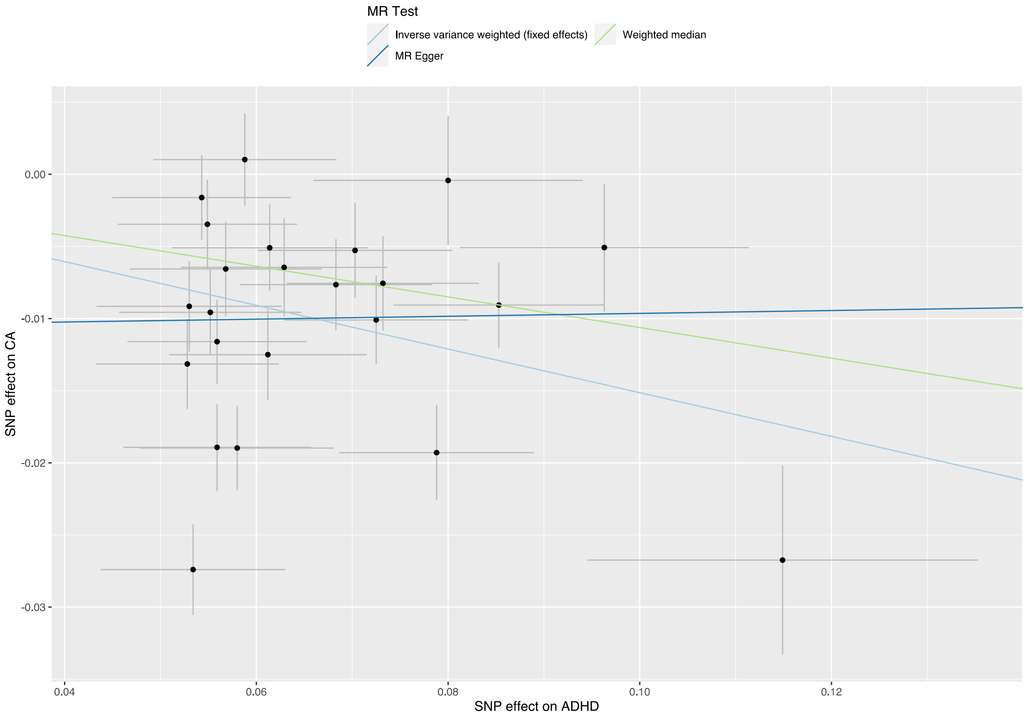

Supplementary Figure 126: scatterplot of ADHD against cognitive abilities
Abbreviations: MR: Mendelian randomization; SNP: single nucleotide polymorphism; CA: cognitive abilities; ADHD: attention deficit hyperactivity disorder.
Legend: n=23 SNPs. Each dot represents a SNP, with the slope of each line corresponding to the estimated MR effect per method.

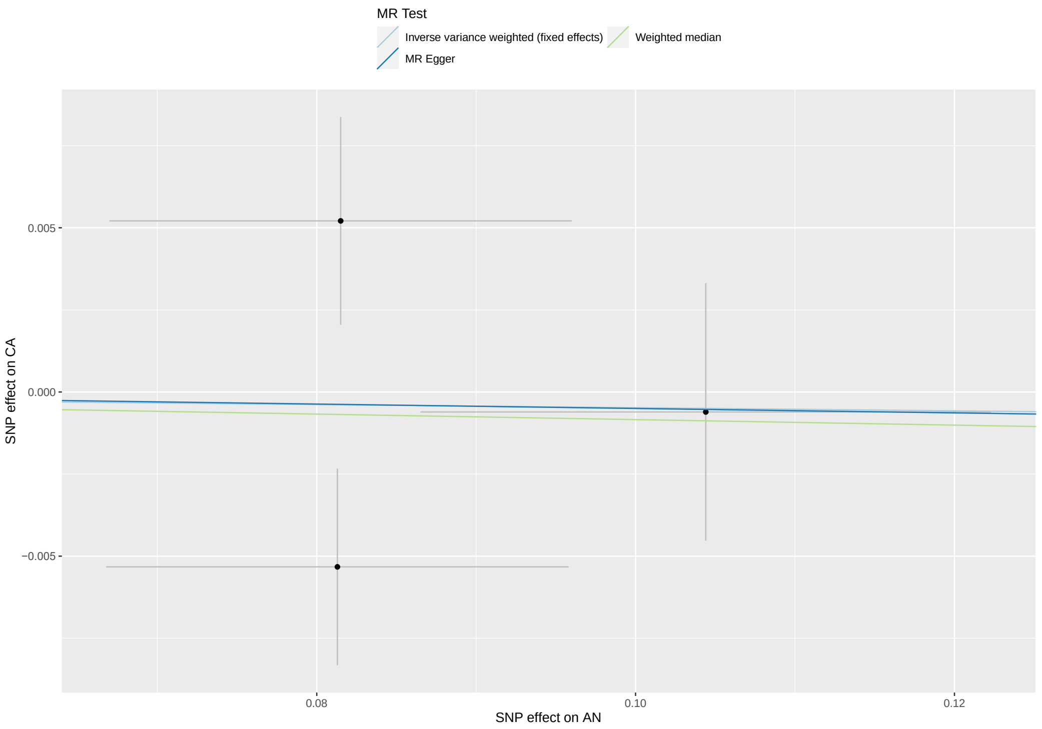

Supplementary Figure 127: scatterplot of AN against cognitive abilities
Abbreviations: MR: Mendelian randomization; SNP: single nucleotide polymorphism; CA: cognitive abilities; AN: anorexia nervosa.
Legend: n=3 SNPs. Each dot represents a SNP, with the slope of each line corresponding to the estimated MR effect per method.

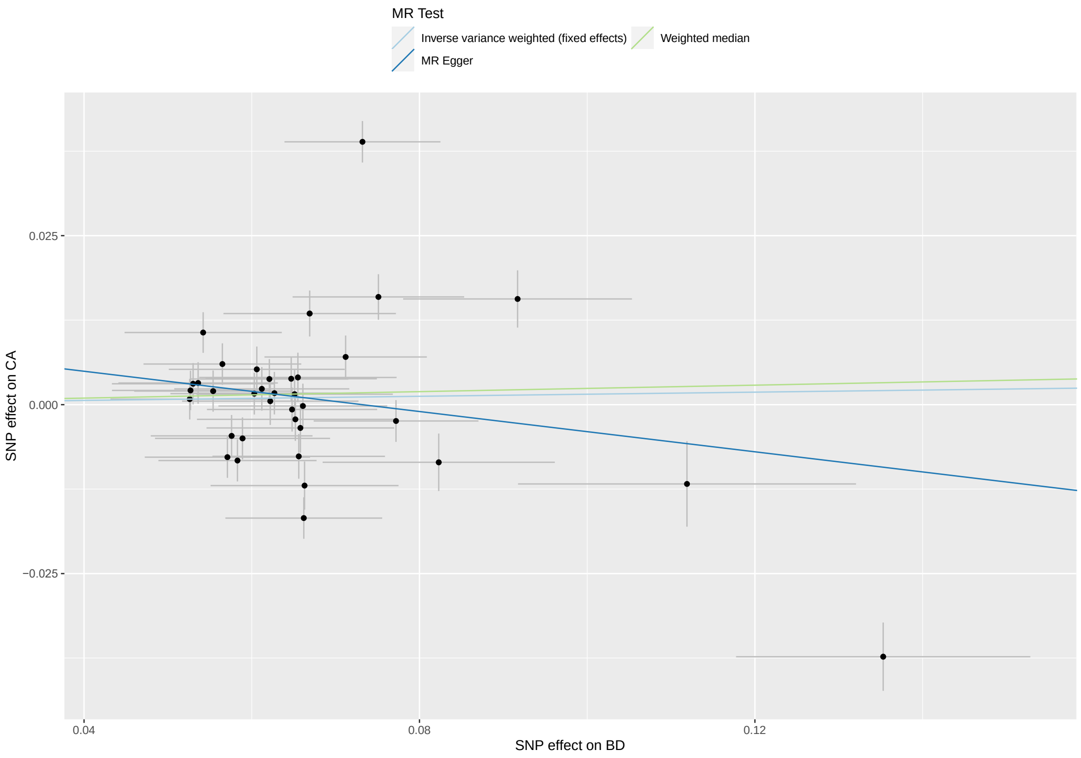

Supplementary Figure 128: scatterplot of BD against cognitive abilities
Abbreviations: MR: Mendelian randomization; SNP: single nucleotide polymorphism; CA: cognitive abilities; BD: bipolar disorder.
Legend: n=36 SNPs. Each dot represents a SNP, with the slope of each line corresponding to the estimated MR effect per method.

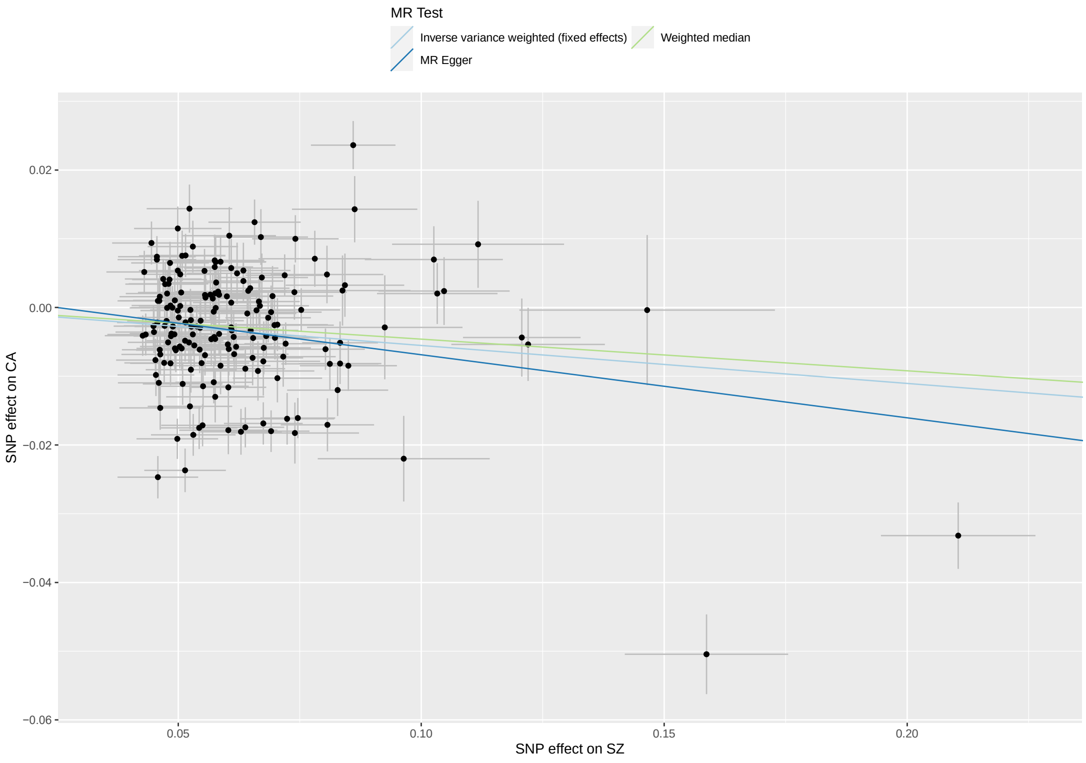

Supplementary Figure 129: scatterplot of SZ against cognitive abilities
Abbreviations: MR: Mendelian randomization; SNP: single nucleotide polymorphism; CA: cognitive abilities; SZ: schizophrenia.
Legend: n=175 SNPs. Each dot represents a SNP, with the slope of each line corresponding to the estimated MR effect per method.

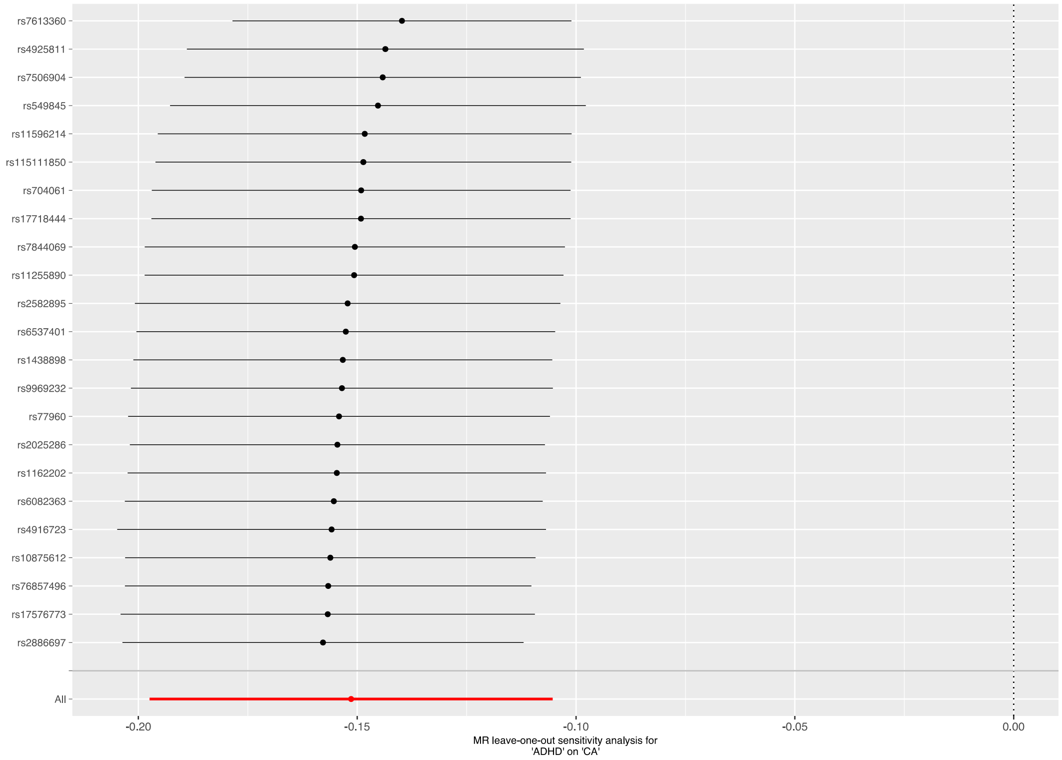

Supplementary Figure 130: leave-one-out analysis of ADHD against cognitive abilities
Abbreviations: MR: Mendelian randomization; CA: cognitive abilities; ADHD: attention deficit hyperactivity disorder.
Legend: n=23 SNPs. Forest plot of individual and combined SNP MR estimated effect size. The effect estimates represent the linear effect for the mean cognitive ability per ADHD status, and the error bars represent 95% CIs.

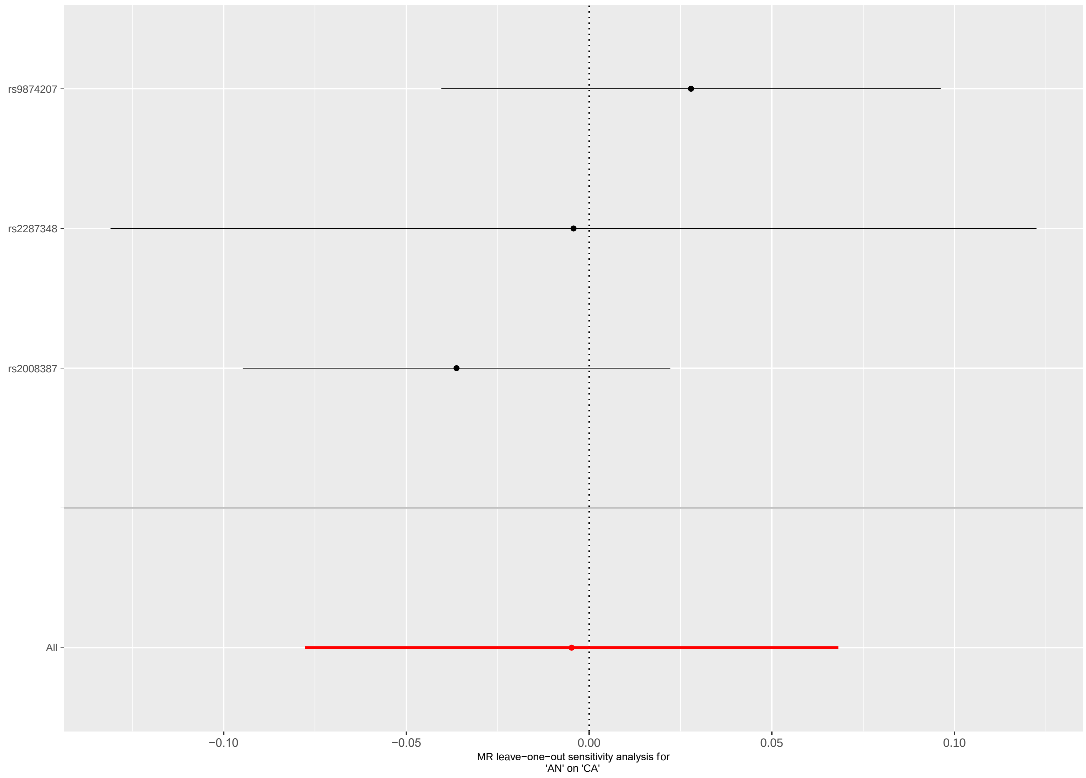

Supplementary Figure 131: leave-one-out analysis of AN against cognitive abilities
Abbreviations: MR: Mendelian randomization; CA: cognitive abilities; AN: anorexia nervosa.
Legend: n=3 SNPs. Forest plot of individual and combined SNP MR estimated effect size.
The effect estimates represent the linear effect for the mean cognitive ability per AN status,
and the error bars represent 95% CIs.

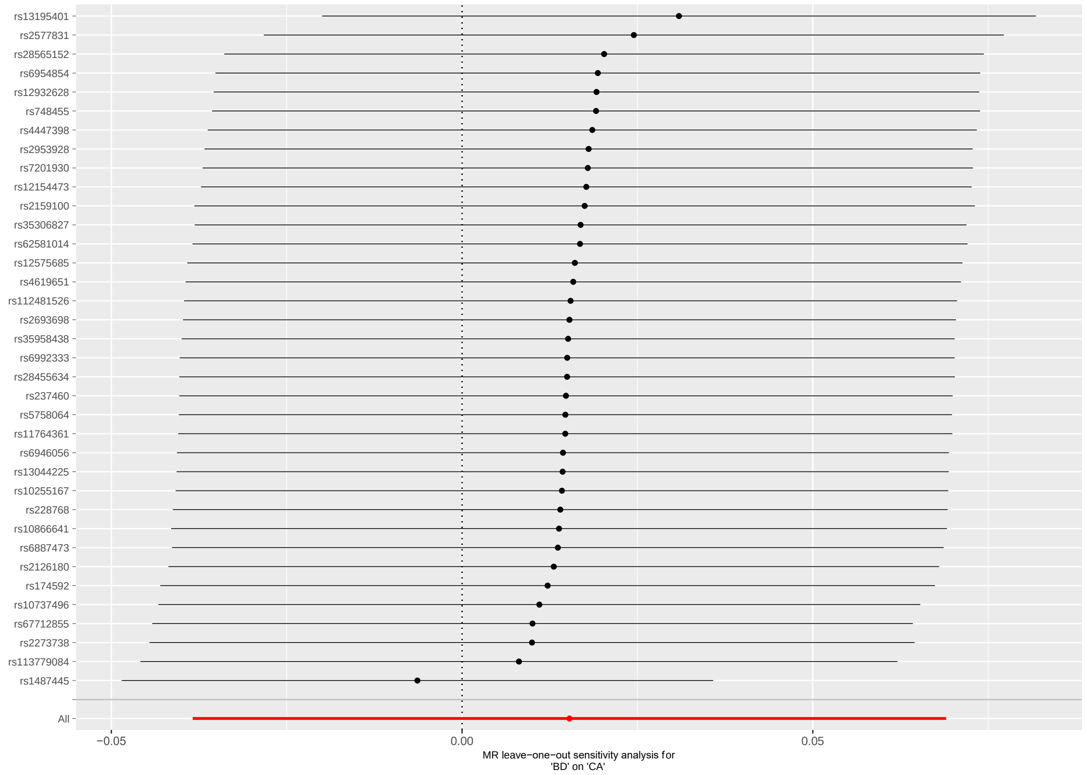

Supplementary Figure 132: leave-one-out analysis of BD against cognitive abilities
Abbreviations: MR: Mendelian randomization; CA: cognitive abilities; BD: bipolar disorder.

Legend: n=36 SNPs. Forest plot of individual and combined SNP MR estimated effect size. The effect estimates represent the linear effect for the mean cognitive ability per BD status, and the error bars represent 95% CIs.

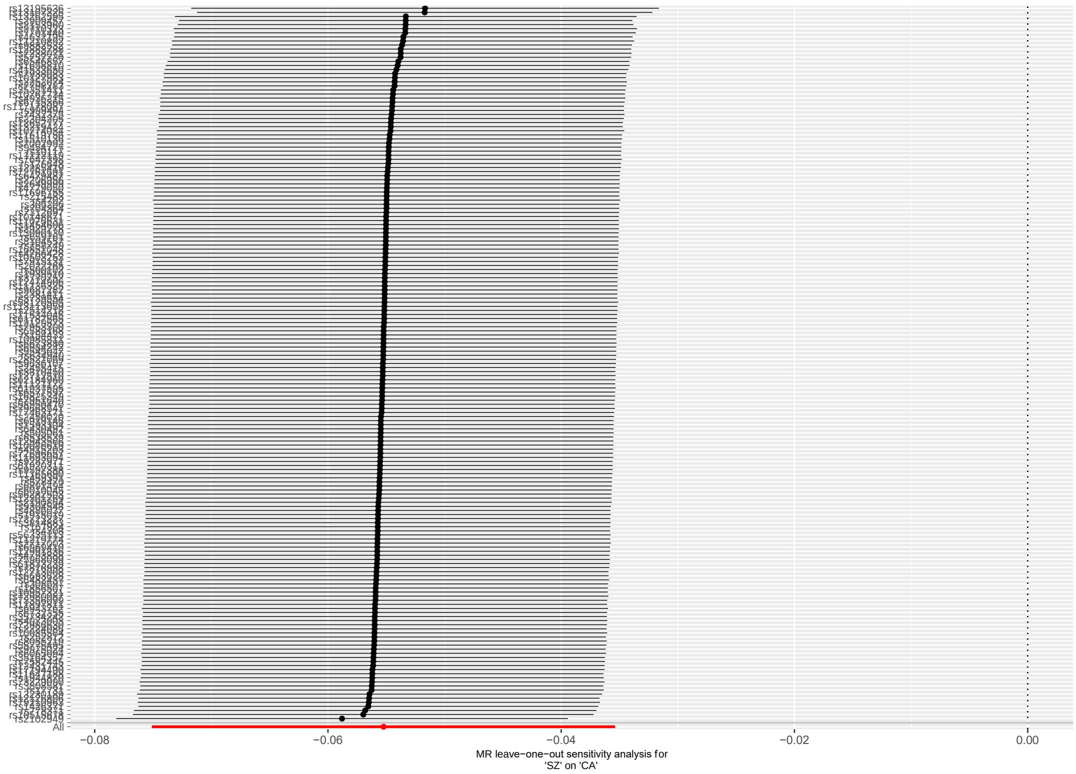

Supplementary Figure 133: leave-one-out analysis of SZ against cognitive abilities
Abbreviations: MR: Mendelian randomization; CA: cognitive abilities; SZ: schizophrenia.
Legend: n=175 SNPs. Forest plot of individual and combined SNP MR estimated effect size. The effect estimates represent the linear effect for the mean cognitive ability per SZ status, and the error bars represent 95% CIs.

Supplementary Table 29: CAUSE results of the relations between cognitive abilities and mental illnesses

**Model 1**	**Model 2**	**Δ ELPD**	**SE Δ ELPD**	**z-score**	**p-value[†]**
		Fw: CA on ADHD			
Null	Sharing	-69.24	9.79	-7.07	**1.55×10^{-12}**
Null	Causal	-76.54	10.84	-7.06	**1.67×10^{-12}**
Sharing	Causal	-7.30	1.21	-6.04	**1.54×10^{-9}**
		Bw: ADHD on CA			
Null	Sharing	-22.00	4.75	-4.64	**3.48×10^{-6}**
Null	Causal	-28.89	6.27	-4.61	**4.03×10^{-6}**
Sharing	Causal	-6.89	1.57	-4.39	**1.13×10^{-5}**
		Fw: CA on AN			
Null	Sharing	-0.01	0.53	-0.02	0.984
Null	Causal	-1.01	2.02	-0.50	0.617
Sharing	Causal	-1.00	1.50	-0.66	0.509
		Bw: AN on CA			
Null	Sharing	0.46	0.07	6.83	**8.49×10^{-12}**
Null	Causal	1.35	0.07	18.32	**5.73×10^{-75}**

Sharing	Causal	0.89	0.02	40.41	**<1^{-1000}**
Fw: CA on ANX					
Null	Sharing	-7.02	3.68	-1.91	0.056
Null	Causal	-7.17	4.27	-1.68	0.093
Sharing	Causal	-0.14	1.38	-0.11	0.912
Bw: ANX on CA					
Null	Sharing	0.25	0.09	2.88	**0.004**
Null	Causal	1.08	0.30	3.61	**3.06×10^{-4}**
Sharing	Causal	0.83	0.25	3.35	**0.001**
Fw: CA on ASD					
Null	Sharing	-0.40	0.88	-0.46	0.646
Null	Causal	-1.88	2.43	-0.78	0.435
Sharing	Causal	-1.48	1.57	-0.94	0.347
Bw: ASD on CA					
Null	Sharing	0.31	0.17	1.88	0.060
Null	Causal	0.39	1.01	0.39	0.697
Sharing	Causal	0.08	0.85	0.09	0.928
Fw: CA on BD					
Null	Sharing	0.42	0.15	2.80	**0.003**
Null	Causal	0.79	0.98	0.81	0.210
Sharing	Causal	0.37	0.84	0.84	0.328
Bw: BD on CA					
Null	Sharing	0.45	0.07	5.98	**2.23×10^{-9}**
Null	Causal	1.09	0.52	2.08	**0.038**
Sharing	Causal	0.64	0.45	1.42	0.156
Fw: CA on MDD					
Null	Sharing	-7.83	2.96	-2.64	**0.008**
Null	Causal	-12.20	4.61	-2.65	**0.008**
Sharing	Causal	-4.38	1.68	-2.60	**0.009**
Bw: MDD on CA					
Null	Sharing	0.36	0.08	4.74	**2.14×10^{-6}**
Null	Causal	0.78	0.73	1.07	0.285
Sharing	Causal	0.42	0.66	0.64	0.522
Fw: CA on OCD					
Null	Sharing	-1.11	1.55	-0.72	0.471
Null	Causal	-1.66	2.54	-0.65	0.516
Sharing	Causal	-0.54	1.10	-0.50	0.617
Bw: OCD on CA					
Null	Sharing	0.28	0.03	8.40	**4.64×10^{-17}**
Null	Causal	1.05	0.11	9.63	**5.97×10^{-22}**
Sharing	Causal	0.77	0.10	8.08	**6.48×10^{-16}**
Fw: CA on PTSD					
Null	Sharing	-10.48	3.93	-2.67	**0.008**
Null	Causal	-13.58	5.18	-2.62	**0.009**
Sharing	Causal	-3.10	1.48	-2.09	**0.037**
Bw: PTSD on CA					
Null	Sharing	0.27	0.09	2.86	**0.004**
Null	Causal	0.77	0.63	1.23	0.219
Sharing	Causal	0.50	0.55	0.92	0.358

Fw: CA on SZ					
Null	Sharing	-6.54	2.36	-2.77	**0.006**
Null	Causal	-12.02	4.25	-2.83	**0.005**
Sharing	Causal	-5.48	1.90	-2.88	**0.004**
Bw: SZ on CA					
Null	Sharing	-5.95	2.35	-2.53	**0.011**
Null	Causal	-10.84	4.20	-2.58	**0.010**
Sharing	Causal	-4.89	1.86	-2.62	**0.009**

Abbreviations: MR: mendelian randomization; ELPD: expected log pointwise posterior density; 95%CI: 95% confidence interval; SE: standard error; Fwd: forward MR; Bwd: backward MR; CA: cognitive abilities; ADHD: attention deficit hyperactivity disorder; AN: anorexia nervosa; ANX: anxiety disorder; ASD: autism spectrum disorder; BD: bipolar disorder; MDD: major depressive disorder; OCD: obsessive-compulsive disorder; PTSD: post-traumatic stress disorder; SZ: schizophrenia.
Legend: † Based on t-test. The p-values are two-sided and not adjusted for multiple testing, therefore p-value<0.05 was considered significant (and reported in bolded text).

Supplementary Table 30: Results of univariable bidirectional Mendelian Randomization of cognitive abilities against mental illnesses, after Steiger filtering

**MR**	**N SNP**	**IVW, B (95% CI)**	**IVW p-value**	**WM, B (95% CI)**	**WM p-value**	**MR-Egger, B (95% CI)**	**MR-Egger p-value**	**Egger intercept p-value**	**Mean F**
CA on ADHD	131	-0.638 (-0.720; -0.556)	**1.37×10^{-52}**	-0.493 (-0.632; -0.354)	**4.05×10^{-11}**	-0.635 (-1.27; -0.005)	0.050	0.993	44.0
CA on AN	132	0.298 (0.180; 0.416)	**1.06×10^{-7}**	0.366 (0.170; 0.561)	**9.07×10^{-5}**	0.854 (0.183; 1.52)	**0.014**	0.147	44.0
CA on ANX	125	-0.109 (-0.353; 0.135)	0.380	-0.068 (-0.425; 0.288)	0.706	0.143 (-0.967; 1.25)	0.801	0.138	44.1
CA on ASD	116	0.102 (-0.026; 0.229)	0.117	0.172 (-0.021; 0.366)	0.081	0.557 (-0.096; 1.21)	0.097	0.076	44.3
CA on BD	112	-0.083 (-0.173; 0.006)	0.069	-0.138 (-0.283; 0.006)	0.061	-0.082 (-0.623; 0.460)	0.768	0.632	42.9
CA on MDD	137	-0.153 (-0.228; -0.078)	**6.79×10^{-5}**	-0.106 (-0.228; 0.016)	0.089	-0.098 (-0.557; 0.362)	0.677	0.282	43.9
CA on OCD	94	-0.013 (-0.354; 0.327)	0.940	-0.097 (-0.583; 0.389)	0.695	-0.125 (-1.57; 1.32)	0.866	0.855	44.8
CA on PTSD	136	-0.139 (-0.264; -0.013)	**0.030**	-0.207 (-0.406; -0.009)	**0.041**	0.220 (-0.458; 0.898)	0.525	0.498	43.9
CA on SZ	130	-0.209 (-0.279; -0.139)	**6.16×10^{-12}**	-0.140 (-0.276; -0.005)	**0.043**	0.161 (-0.624; 0.945)	0.689	**0.001**	44

Abbreviations: CA: cognitive abilities; ADHD: attention deficit hyperactivity disorder; AN: anorexia nervosa; ANX: anxiety disorder; ASD: autism spectrum disorders; BD: bipolar disorder; MDD: major depressive disorder; OCD: obsessive-compulsive disorder; PTSD: post-traumatic stress disorder; SZ: schizophrenia; MR: mendelian randomization; SNP: single nucleotide polymorphism; IVW: inverse variance weighted (fixed effect); B: effect estimates are log-odds; 95% CI: 95% confidence interval; WM: weighted median; NR: not reported because not enough SNP to perform MR.

Legend: All statistical tests were two-sided. The p-values were not adjusted for multiple testing, therefore p-value<0.05 was considered significant (and reported in bolded text).

Multivariable Mendelian Randomization of poverty indicators and cognitive abilities against mental illness

Supplementary Table 31: Multivariable Mendelian Randomization results of household income and cognitive abilities on mental illness

Regression	N SNP	IVW, B (95% CI)	IVW p-value
Outcome: ADHD	192		
Exposure 1: HI		-0.124 (-0.326; 0.078)	0.230
Exposure 2: CA		-0.508 (-0.639; -0.376)	**4.10×10^{-14}**
Outcome: AN	201		
Exposure 1: HI		0.010 (-0.229; 0.250)	0.933
Exposure 2: CA		0.350 (0.202; 0.498)	**3.57×10^{-6}**
Outcome: ANX	199		
Exposure 1: HI		0.010 (-0.350; 0.370)	0.956
Exposure 2: CA		-0.260 (-0.475; -0.045)	**0.018**
Outcome: ASD	204		
Exposure 1: HI		0.196 (-0.047; 0.440)	0.114
Exposure 2: CA		0.273 (0.115; 0.432)	**0.001**
Outcome: BD	195		
Exposure 1: HI		0.049 (-0.164; 0.261)	0.654
Exposure 2: CA		-0.021 (-0.159; 0.117)	0.766
Outcome: MDD	204		
Exposure 1: HI		-0.029 (-0.177; 0.119)	0.701
Exposure 2: CA		-0.118 (-0.211; -0.025)	**0.013**
Outcome: OCD	204		
Exposure 1: HI		0.108 (-0.341; 0.558)	0.636
Exposure 2: CA		0.296 (0.013; 0.580)	**0.041**
Outcome: PTSD	204		
Exposure 1: HI		0.143 (-0.052; 0.338)	0.150
Exposure 2: CA		-0.077 (-0.206; 0.051)	0.236
Outcome: SZ	195		
Exposure 1: HI		0.085 (-0.167; 0.336)	0.510
Exposure 2: CA		-0.163 (-0.328; 0.002)	0.052

Abbreviations: SNP: single nucleotide polymorphism; IVW: multivariable mendelian randomization via inverse variance weighted method (random effects); B: effect estimates are log-odds; 95% CI: 95% confidence intervals; ADHD: attention deficit hyperactivity disorder; HI: household income; CA: cognitive abilities; AN: anorexia nervosa; ANX: anxiety disorder; ASD: autism spectrum disorder; BD: bipolar disorder; MDD: major depressive disorder; OCD: obsessive-compulsive disorder; PTSD: post-traumatic stress disorder; SZ: schizophrenia.
Legend: All statistical tests were two-sided. The p-values were not adjusted for multiple testing, therefore p-value<0.05 was considered significant (and reported in bolded text).

Supplementary Table 32: Multivariable Mendelian Randomization results of occupational income and cognitive abilities on mental illness

Regression	N SNP	IVW, B (95% CI)	IVW p-value

Outcome: ADHD<br>Exposure 1: OI<br>Exposure 2: CA	163	 -0.189 (-0.427; 0.048)<br>-0.511 (-0.654; -0.368)	 0.118<br>**2.36×10^{-12}**
Outcome: AN<br>Exposure 1: OI<br>Exposure 2: CA	165	 0.248 (-0.021; 0.517)<br>0.329 (0.166; 0.491)	 0.071<br>**7.29×10^{-5}**
Outcome: ANX<br>Exposure 1: OI<br>Exposure 2: CA	164	 -0.032 (-0.425; 0.360)<br>0.121 (-0.600; -0.126)	 0.872<br>**0.003**
Outcome: ASD<br>Exposure 1: OI<br>Exposure 2: CA	166	 0.393 (0.103; 0.682)<br>0.267 (0.094; 0.441)	 **0.008**<br>**0.003**
Outcome: BD<br>Exposure 1: OI<br>Exposure 2: CA	166	 0.204 (-0.039; 0.447)<br>0.050 (-0.097; 0.196)	 0.100<br>0.506
Outcome: MDD<br>Exposure 1: OI<br>Exposure 2: CA	166	 -0.079 (-0.258; 0.101)<br>-0.121 (-0.229; -0.014)	 0.389<br>**0.027**
Outcome: OCD<br>Exposure 1: OI<br>Exposure 2: CA	166	 -0.133 (-0.657; 0.391)<br>0.201 (-0.114; 0.516)	 0.618<br>0.210
Outcome: PTSD<br>Exposure 1: OI<br>Exposure 2: CA	166	 0.143 (-0.092; 0.378)<br>-0.183 (-0.326; -0.041)	 0.234<br>**0.011**
Outcome: SZ<br>Exposure 1: OI<br>Exposure 2: CA	166	 -0.003 (-0.298; 0.291)<br>-0.229 (-0.406; -0.052)	 0.981<br>**0.011**

Abbreviations: SNP: single nucleotide polymorphism; IVW: multivariable mendelian randomization via inverse variance weighted method (random effects); B: effect estimates are log-odds; 95% CI: 95% confidence intervals; ADHD: attention deficit hyperactivity disorder; OI: occupational income; CA: cognitive abilities; AN: anorexia nervosa; ANX: anxiety disorder; ASD: autism spectrum disorder; BD: bipolar disorder; MDD: major depressive disorder; OCD: obsessive-compulsive disorder; PTSD: post-traumatic stress disorder; SZ: schizophrenia.
Legend: All statistical tests were two-sided. The p-values were not adjusted for multiple testing, therefore p-value<0.05 was considered significant (and reported in bolded text).

Supplementary Table 33: Multivariable Mendelian Randomization results of social deprivation and cognitive abilities on mental illness

**Regression**	**N SNP**	**IVW, B (95% CI)**	**IVW p-value**
Outcome: ADHD<br>Exposure 1: SD<br>Exposure 2: CA	152	 -0.135 (-0.317; 0.048)<br>-0.656 (-0.785; -0.528)	 0.147<br>**1.80×10^{-23}**
Outcome: AN<br>Exposure 1: SD<br>Exposure 2: CA	161	 -0.240 (-0.448; -0.033)<br>0.342 (0.185; 0.499)	 **0.023**<br>**1.90×10^{-5}**
Outcome: ANX<br>Exposure 1: SD	161	 0.083 (-0.223; 0.389)	 0.593

Exposure 2: CA		-0.267 (-0.490; -0.44)	**0.019**
Outcome: ASD<br>Exposure 1: SD<br>Exposure 2: CA	162	 -0.354 (-0.587; -0.120)<br>0.235 (0.066; 0.404)	 **0.003**<br>**0.007**
Outcome: BD<br>Exposure 1: SD<br>Exposure 2: CA	155	 0.088 (-0.124; 0.299)<br>-0.007 (-0.159; 0.145)	 0.416<br>0.925
Outcome: MDD<br>Exposure 1: SD<br>Exposure 2: CA	162	 0.005 (-0.142; 0.152)<br>-0.129 (-0.229; -0.028)	 0.946<br>**0.012**
Outcome: OCD<br>Exposure 1: SD<br>Exposure 2: CA	162	 -0.055 (-0.519; 0.409)<br>0.335 (0.023; 0.647)	 0.817<br>**0.035**
Outcome: PTSD<br>Exposure 1: SD<br>Exposure 2: CA	162	 -0.053 (-0.244; 0.139)<br>-0.140 (-0.279; -0.002)	 0.588<br>**0.047**
Outcome: SZ<br>Exposure 1: SD<br>Exposure 2: CA	155	 0.142 (-0.104; 0.387)<br>-0.218 (-0.395; -0.040)	 0.259<br>**0.016**

Abbreviations: SNP: single nucleotide polymorphism; IVW: multivariable mendelian randomization via inverse variance weighted method (random effects); B: effect estimates are log-odds; 95% CI: 95% confidence intervals; ADHD: attention deficit hyperactivity disorder; SD: social deprivation measured with Towsend deprivation index; CA: cognitive abilities; AN: anorexia nervosa; ANX: anxiety disorder; ASD: autism spectrum disorder; BD: bipolar disorder; MDD: major depressive disorder; OCD: obsessive-compulsive disorder; PTSD: post-traumatic stress disorder; SZ: schizophrenia.
Legend: All statistical tests were two-sided. The p-values were not adjusted for multiple testing, therefore p-value<0.05 was considered significant (and reported in bolded text).

Frequently Asked Questions (FAQ)

What was your goal with this study?

Our goal was to examine whether there is evidence for a causal relationship between poverty and mental illness. A causal role of poverty on mental illness (and vice versa) would be of significant importance for mental illness prevention and for our understanding of mental health.

Is the relation between poverty and mental health not already obvious?

Previous research has identified strong correlations between poverty and mental illness, but disentangling cause-and-effect is more difficult. A relation between poverty and mental illness may be due to the effects of mental illness on someone's financial situation (less income or more spending on health care) or may be caused a third factor (which are called confounding factors). For instance, education may play a role in the poverty-mental health relationship.

How do you find evidence of causality?

Scientists usually rely on randomization to infer causality. For example, to determine whether a specific factor, such as a medical treatment, causes a particular outcome, such as depression

reduction, the gold standard is to perform Randomized Controlled Trials (RCTs). These studies involve a random assignment of participants into different groups. Randomization enables the casual distribution of potential confounders across the treatment groups, mitigating their effects, and allowing scientists to attribute observed changes to the effect of the intervention. However, conducting such experiments is not always ethical or feasible, such as when it comes to investigating poverty. Clearly, it would not be ethical to deliberately induce poverty in a subset of individuals to observe whether this leads to the development of mental illnesses.

Fortunately, we are all recruited in an experiment without knowing it, at the point at which we were conceived. Our genes, which have passed on randomly from generation to generation, influence our behaviors but are also markers for our social circumstances. Therefore, we can use genetic information as a proxy for an exposure of interest (in our case poverty), which may be otherwise difficult to manipulate. Since an individual's genetic code is determined at birth and remains stable throughout live, the inherited genetic variants are not affected by anything one may or may not choose to do during life, meaning these are not related to confounding factors. We can use this knowledge to learn about cause-and-effect, by grouping people according to their genetic code. This method is called Mendelian randomization (MR). In this study we used MR to investigate if poverty is causally associated with mental illness, and vice-versa.

The Figure below represents the distinctions and similarities between MR and RCTs.

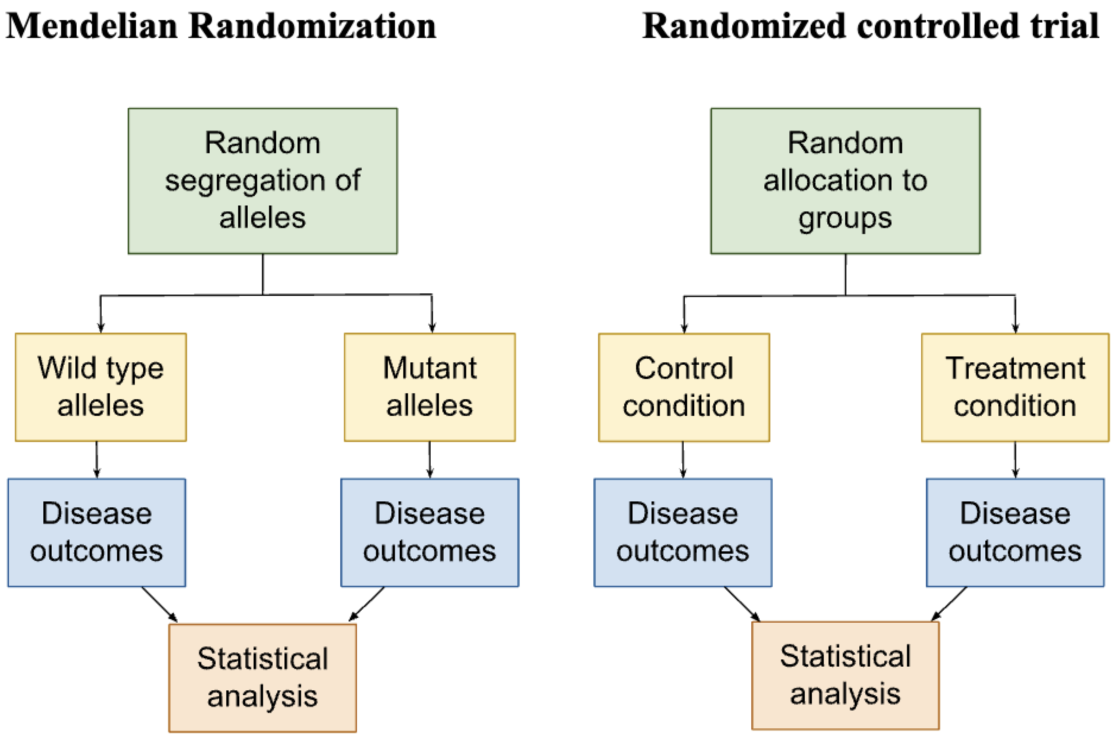

What do you mean when you talk about 'poverty'?

Poverty can be generally described as a situation where people do not have enough (financial) resources to meet their basic needs and face disadvantages in society. But there are several aspects of poverty that can be distinguished. We used the following poverty indicators: household income, occupational income, and social deprivation.

Household income refers to the total amount of money earned by everyone in a family across one-year span. When household income is low, it means there is not enough money to cover essential things like food, housing, and healthcare. This makes it difficult for people to have a decent quality of life.

Occupational income is the money individuals earn from their jobs. If someone's occupational income is limited, it means they might have low-paying jobs or struggle to find stable employment. This makes it hard to make ends meet and can contribute to poverty.

Social deprivation means not having access to things that are necessary for a good life. This includes things like education, healthcare, housing, transportation, and support from friends and community. When people experience high levels of social deprivation, it can be even more challenging to escape poverty and improve their circumstances.

What did you do in this study?

Our study aimed to investigate the relationship between poverty and various mental illnesses using the method described above, called Mendelian randomization. We utilized genetic variations associated with both poverty and mental illnesses to explore causality. The mental illnesses we focused on included attention deficit and hyperactivity disorder (ADHD), anorexia nervosa, anxiety disorders, autism spectrum disorders, bipolar disorder, major depressive disorder, obsessive-compulsive disorder, post-traumatic stress disorder (PTSD), and schizophrenia. We also considered the impact of cognitive ability on this relationship, recognizing that education levels may influence both income and health-related choices. To conduct our statistical analyses, we incorporated cognitive ability as a potential confounding factor.

What is meant by the genetic architecture of poverty, and how did you identify genetic variations related to poverty?

Our study employed genome-wide association studies (GWAS) to identify genetic variations linked to poverty in a white British adult sample. A GWAS is a type of study that searches for genetic variants that are linked to some outcome: they have been instrumental in understanding associations of genetic variants with various health conditions, such as schizophrenia or cardiovascular disease, and traits, like height and weight. The genetic variants we just mentioned are called Single-Nucleotide Polymorphisms, or “SNPs”. These are points on the DNA—many millions of them—where people tend to differ in which nucleotide (which one of the four chemical “letters” of DNA: A, C, T, and G) is present. For example, 25% of people might have an “A” at that point on their DNA, whereas the remaining 75% have a “G”. This is the most common, though far from the only, way in which people differ in their DNA. A GWAS study lines up all the SNPs a person has and tests the extent to which each one is linked to the outcome of interest. In the case of poverty each SNP might coincide with the odd of being poor without any inference why this relation exists. Our GWAS estimated a common measure of poverty that captured the genetic overlap between our three indices of poverty: household income, occupational income, and social deprivation. Using genetic data to identify poverty risk may appear unconventional since poverty is not traditionally viewed as a biological condition or trait. However, with increasing GWAS sample sizes, there is growing power to detect SNPs associated with multifactorial phenotypes like diabetes, mental illness, or social conditions such as income and poverty. Clearly, the more complex or multifactorial the phenotype is, the less likely is to observe a direct genetic effect. For example, in type II diabetes mellitus, a multifactorial disease, GWAS could identify genetic variants associated not only with the disease itself but also with

related individual characteristics like a preference for consuming sweets or maintaining a sedentary lifestyle. In such cases, the genetic variants associated with upstream or downstream factors exhibit pleiotropic effects. This understanding extends to the genetic architecture of poverty, where GWAS aids in unraveling its complex genetic underpinnings, even if direct effects are improbable.

What did you find?

We found that our common factor of poverty captured the majority of the genetic variance, with household income, occupational income, and social deprivation accounting for 100%, 88%, and 74%, respectively. In addition, the summary statistics used to evaluate the genotype-phenotype association (i.e., the Mean Chi2, the LDSC intercept, and the narrow sense heritability) supported a stronger relationship for the poverty common factor than the single indicators. Through our investigations using Mendelian randomization, we found compelling evidence that mental illness can contribute to poverty, and in turn, poverty can play a causal role in the development of ADHD, major depressive disorder, and schizophrenia. Interestingly, we observed that poverty was inversely associated with the risk of anorexia nervosa. Additionally, we found that cognitive ability accounted for some of the variance in the poverty-mental illness relationship.

How may poverty and mental illness be related?

Although the relationships we found between poverty and some mental illnesses converged across a series of analyses, it is very unlikely there are direct genetic effects. Rather, the genetic relationship between poverty and mental illness is likely to involve psychosocial mechanisms that eventually are related to poverty, this process is called vertical pleiotropy. Whilst the investigation of each pathway from poverty to mental illness separately is beyond the scope of this study, it is important to acknowledge that some psychopathological features (such as perfectionism in the context of anorexia nervosa, or attention deficit in the context of ADHD, or overall individual's health status) may facilitate something else that is linked to income (such as education attainment). In that context, Mendelian randomization can best be viewed as a way to approximate randomly assign heritable traits that give rise to income differences. In the current study, we particularly investigated the role of cognitive ability using multivariable Mendelian randomization. Future research should a use similar approach to investigate the role of other traits that link poverty to mental health outcomes.

Why is this relevant?

These findings underscore the potential benefits of income-sensitive mental health policies as they may promote better mental health outcomes for all. It suggests that targeted interventions aimed at addressing poverty as a cause of mental illness will advance health equity. Our research provides robust evidence supporting the need to address poverty as a significant contributing factor to the development of mental illness.
In summary, our study highlights the critical role poverty plays in mental illness risk and emphasizes the urgency of implementing effective strategies that address both poverty and mental health concerns. This is particularly relevant in an era where inequities and mental illness are growing worldwide.

To whom do the results apply?

Our findings were made in the context of the western societies and in particular Great Britain (that is, the participants were from England, Scotland, Wales Western Europe and the USA), in approximately the last ten years. In addition, our analysis was restricted to people who described their ethnic background as White. It will be important to investigate the extent to

which the poverty-linked genetic variants and the associations with mental illness we have found apply to people of different backgrounds, and those from different countries, cultures, and time periods.

Are the associations not just due to “dynastic effects”?

A dynastic effect refers to the transmission of socioeconomic or health-related outcomes across generations within a family. This means that the circumstances, advantages, or disadvantages experienced by one generation may influence the outcomes of the following generations. In the context of income and poverty, if one grows up in a family with a history of important income losses or economic debts, it could be that their income levels in adulthood are not related to their skills and abilities tied to their earning capacity, but rather to the transmission of disadvantaged economic conditions from their parents. Importantly, the dynastic effect can be a potential source of bias in Mendelian randomization (MR) studies. MR relies on the random assortment of genetic variants during meiosis, ensuring that the genetic instrument (SNPs used as proxies for exposure) is not associated with confounding factors. However, if there is a dynastic effect, meaning that the genetic variants not only influence the exposure of interest in the individual but also the exposures of their ancestors or descendants, it could violate the assumptions of MR. Addressing dynastic effects in MR studies may require additional methodological considerations, such as the use of family-based MR designs, or exploring alternative genetic instruments that are less likely to be influenced by dynastic transmission. It is important to note that our primary analysis was conducted on a general factor of poverty. This poverty factor was derived using the variance that is common across each indicator of poverty meaning if dynastic effects were the sole or main signal captured by social deprivation they would not be included in this general factor.

Does this study imply that poverty and mental illnesses are determined at birth?

No, our results do not suggest that an individual's income or likelihood of developing mental illnesses is predetermined at birth. The associations we observed between genetic variations, poverty, and mental health were small and based on extensive sample sizes. For example, the estimated heritability (commonly denoted as h2 in genetic studies) of our poverty factor was 8.38%, indicating that only around 8% of the variation in poverty levels among individuals is attributable to the effect of genetics. For schizophrenia, which is likely the most powerful GWAS included in our research, the estimated h2 was around 20%. These numbers indicate that even individuals with similar genes vary with respect to poverty and mental health outcomes. However, there is a slightly elevated likelihood that individuals with specific combinations of genetic variants may have higher incomes or be more susceptible to mental illnesses.

Do genes determine whether you become poor?

Discovering genetic associations with poverty does not mean that other environmental and genetic factors do not influence them – and as we noted, the effects we observed are small. The concept of "genetic determinism" is false. Another example is educational achievement. Although intelligence and cognitive abilities have a genetic component, environmental factors such as access to quality education, supportive learning environments, and parental involvement play crucial roles in determining educational outcomes. Individuals with the same genetic make-up can achieve different educational levels based on their environment and opportunities.

Isn’t research like this going to lead to discrimination against those with certain genes?

Unfortunately, a lot of scientific research has the potential to be misused. It is crucial to approach genetic research with caution and recognize that it is just one piece of the puzzle

when exploring the relationship between poverty and mental illness. While genetic research provides valuable insights, it is important to acknowledge the limitations that include significant influence of many other environmental and genetic factors in the development of mental illness and socioeconomic disparities.

To prevent discrimination, it is crucial to emphasize the importance of creating inclusive and supportive environments that foster equal opportunities and access to resources for all individuals, regardless of their genetic or environmental backgrounds. By addressing the broader social determinants of mental health and socioeconomic disparities, we can work towards a more equitable society that promotes mental well-being and socioeconomic mobility for all.

Does this study imply that mentally ill people are not able to earn the money they need to live?

While the research indicates that there may be causal relations between mental illness and poverty, it does not imply that individuals with mental illness are unable to earn the money they need to live. Mental illness can impact individuals in different ways. Factors such as access to resources, supportive environments, and individual strengths and abilities also play significant roles in determining socioeconomic outcomes.

It is crucial to avoid generalizations and stereotypes about the capabilities and potential of individuals with mental illness. Many individuals with mental health conditions lead fulfilling lives, hold jobs, and contribute to society. However, it is true that mental health challenges can present additional obstacles and may require appropriate support systems to ensure individuals can access equal opportunities. The research highlights the importance of addressing the systemic barriers and stigma that individuals with mental illness may face in employment and socioeconomic domains. By promoting inclusive workplaces, providing reasonable accommodations, and fostering supportive environments, we can create conditions that enable individuals with mental illness to thrive professionally and achieve financial stability.

What are the practical applications of this research?

It is important to note that while our research provides valuable insights, any practical implications may vary depending on the specific context and population under study. The goal of this study was to better understand the way poverty and mental illness are related, building a picture of how genes relate to different phenotypes. To move from such “basic” understanding to practical applications, more work is required, and new questions arise, about interventions and policies. Foremost replication of our findings in different populations is essential. Subsequently other research will then need to be performed to identify practical challenges and provide guidelines to maximize efficacy of health-promoting interventions. The multifaceted nature of poverty asks for a carefully designed intervention, considerate of broader social determinants of mental health, such as access to healthcare, education and social support. Implementation of interventions and policies should be done in a thoughtful and context-specific manner, taking into account the unique needs and resources of different communities and individuals.
