# Supplementary material for: Investigating the impact of poverty on mental illness in the UK Biobank using Mendelian randomization
Source: Nat Hum Behav. 2024 Jul 10;8(9):1771–83. doi: 10.1038/s41562-024-01919-3 (PMC11420075; doi:10.1038/s41562-024-01919-3)
Supplement: Supplementary file 2 — Reporting Summary [file 41562_2024_1919_MOESM2_ESM.pdf]

nature portfolio

Corresponding author(s): Mattia Marchi

Last updated by author(s): May 24, 2024

Reporting Summary

Nature Portfolio wishes to improve the reproducibility of the work that we publish. This form provides structure for consistency and transparency in reporting. For further information on Nature Portfolio policies, see our Editorial Policies and the Editorial Policy Checklist.

Statistics

For all statistical analyses, confirm that the following items are present in the figure legend, table legend, main text, or Methods section.

n/a	Confirmed	
☐	☒	The exact sample size (*n*) for each experimental group/condition, given as a discrete number and unit of measurement
☐	☒	A statement on whether measurements were taken from distinct samples or whether the same sample was measured repeatedly
☐	☒	The statistical test(s) used AND whether they are one- or two-sided<br>*Only common tests should be described solely by name; describe more complex techniques in the Methods section.*
☐	☒	A description of all covariates tested
☐	☒	A description of any assumptions or corrections, such as tests of normality and adjustment for multiple comparisons
☐	☒	A full description of the statistical parameters including central tendency (e.g. means) or other basic estimates (e.g. regression coefficient) AND variation (e.g. standard deviation) or associated estimates of uncertainty (e.g. confidence intervals)
☐	☒	For null hypothesis testing, the test statistic (e.g. *F*, *t*, *r*) with confidence intervals, effect sizes, degrees of freedom and *P* value noted<br>*Give P values as exact values whenever suitable.*
☒	☐	For Bayesian analysis, information on the choice of priors and Markov chain Monte Carlo settings
☐	☒	For hierarchical and complex designs, identification of the appropriate level for tests and full reporting of outcomes
☐	☒	Estimates of effect sizes (e.g. Cohen's *d*, Pearson's *r*), indicating how they were calculated

Our web collection on statistics for biologists contains articles on many of the points above.

Software and code

Policy information about availability of computer code

Data collection	No software was used.
Data analysis	The GWAS of household income and social deprivation were performed in REGENIE version 3.1.3 (1). All the other analyses were conducted in R version 4.4.0 (2), using the packages TwosampleMR version 0.6.2 (3), CAUSE version 1.2.0 (4), and GenomicSEM version 0.0.5 (5).<br><br>1: Mbatchou, J. et al. Computationally efficient whole-genome regression for quantitative and binary traits. Nature Genetics 2021 53:7 53, 1097–1103 (2021).<br>2: RStudio Team. RStudio: Integrated Development Environment for R. Preprint at http://www.rstudio.com/ (2022).<br>3: Hemani, G. et al. The MR-Base platform supports systematic causal inference across the human phenome. Elife 7, (2018).<br>4: Morrison, J., Knoblauch, N., Marcus, J. H., Stephens, M. & He, X. Mendelian randomization accounting for correlated and uncorrelated pleiotropic effects using genome-wide summary statistics. Nature Genetics 2020 52:7 52, 740–747 (2020).<br>5: Grotzinger, A. D. et al. Genomic SEM Provides Insights into the Multivariate Genetic Architecture of Complex Traits. Nat Hum Behav 3, 513 (2019).<br><br>The custom codes used to produce the results reported in the manuscript are available at: https://github.com/MattiaMarchi/Common-factor-GWAS---MR

For manuscripts utilizing custom algorithms or software that are central to the research but not yet described in published literature, software must be made available to editors and reviewers. We strongly encourage code deposition in a community repository (e.g. GitHub). See the Nature Portfolio guidelines for submitting code & software for further information.

Data

Policy information about availability of data

All manuscripts must include a data availability statement. This statement should provide the following information, where applicable:

- Accession codes, unique identifiers, or web links for publicly available datasets
- A description of any restrictions on data availability
- For clinical datasets or third party data, please ensure that the statement adheres to our policy

UK Biobank data used in this study are available via the UK Biobank data access process (see http://www.ukbiobank.ac.uk/register-apply/). PGC data can be publicly accessed from https://pgc.unc.edu/for-researchers/download-results/. OI data are publicly available and can be downloaded from https://osf.io/rg8sh/. CA data were obtained from the author of the relevant publication accessible at https://www.nature.com/articles/s41380-017-0001-5. The full GWAS summary statistics of HI, SD, and poverty are uploaded on GWAS catalog (https://www.ebi.ac.uk/gwas/deposition/submission/6572ff9a53c4ef000109d322; Study accession IDs: GCST90302879; GCST90302880; GCST90302881; GCST90302882; GCST90302883; GCST90302884; GCST90302885).

Research involving human participants, their data, or biological material

Policy information about studies with human participants or human data. See also policy information about sex, gender (identity/presentation), and sexual orientation and race, ethnicity and racism.

Reporting on sex and gender	Our study employs Mendelian randomization, utilizing summary statistics from previously conducted GWAS to examine the causal effect of an instrumental variable on a potential outcome. It is important to note that no participants were directly enrolled, and human data was not handled in this research. Our findings have broad applicability across all sexes and genders. Although sex and gender were not directly collected or utilized in this specific Mendelian randomization study, it is worth mentioning that the majority of the GWAS studies we utilized did consider sex as a covariate in their analyses. However, disaggregated sex and gender information was not collected in our source data. As a result, obtaining consent for sharing individual-level data specific to sex and gender is not applicable to our study.
Reporting on race, ethnicity, or other socially relevant groupings	Our study did not use race or ethnic categorisation as conceptual frameworks. Ancestry is reported in the GWAS methods, since this holds significance for the validity of the GWAS. To address population stratification, most of the GWAS included in our study included the principal components as covariate in their analyses. The socially relevant variables in our study were occupational income, household income, and social deprivation. It is important to note that the data used in this study were previously collected within the context of other research, and individual-level data were managed within their respective centers. Household income is the only variable that is dichotomized into different categories, defining UK biobank participants in the following way: (1) low HI: being less than £18,000; (2) low-mid HI: being less than £29,999; (3) mid-high HI: being more than £52,000; (4) high HI: being more than £100,000. This categorisation allowed us to investigate if the effect of income on mental illness is particularly strong at specific income levels.
Population characteristics	Not applicable, since no participants were directly enrolled in our study.
Recruitment	Not applicable, since no participants were directly enrolled in our study.
Ethics oversight	Since our study used summary statistics from previously conducted GWAS, there was no additional ethical approval needed: ethical approval was obtained in all original GWAS studies.

Note that full information on the approval of the study protocol must also be provided in the manuscript.

Field-specific reporting

Please select the one below that is the best fit for your research. If you are not sure, read the appropriate sections before making your selection.

☒ Life sciences ☐ Behavioural & social sciences ☐ Ecological, evolutionary & environmental sciences

For a reference copy of the document with all sections, see nature.com/documents/nr-reporting-summary-flat.pdf

Life sciences study design

All studies must disclose on these points even when the disclosure is negative.

Sample size	To enhance statistical power, the largest available genome-wide association studies were used for both the exposures and the outcomes to perform Mendelian Randomization. The sample sizes ranged from 9,725 to 440,350. Sample size details are reported in Table 2.
Data exclusions	No data were excluded from the analyses.
Replication	To verify the reproducibility of our findings, we have made the lead SNPs list and summary statistics of the common factor poverty GWAS available, the full GWAS summary statistics available upon reasonable request, and the codes for replicating the analyses accessible.
Randomization	Participants were randomly segregated into groups: wild type alleles versus mutant alleles.

Blinding	In Mendelian Randomization, estimation of the genetic instrument effect relies on randomization of genotype during meiosis, which is similar to blinding to allocation.

Reporting for specific materials, systems and methods

We require information from authors about some types of materials, experimental systems and methods used in many studies. Here, indicate whether each material, system or method listed is relevant to your study. If you are not sure if a list item applies to your research, read the appropriate section before selecting a response.

Materials & experimental systems

n/a	Involved in the study
☒	☐ Antibodies
☒	☐ Eukaryotic cell lines
☒	☐ Palaeontology and archaeology
☒	☐ Animals and other organisms
☒	☐ Clinical data
☒	☐ Dual use research of concern
☒	☐ Plants

Methods

n/a	Involved in the study
☒	☐ ChIP-seq
☒	☐ Flow cytometry
☒	☐ MRI-based neuroimaging
